# Supplementary material for: Acetate and glycerol are not uniquely suited for the evolution of cross-feeding in E. coli
Source: PLoS Comput Biol. 2020 Nov 30;16(11):e1008433. doi: 10.1371/journal.pcbi.1008433 (PMC7728234; doi:10.1371/journal.pcbi.1008433)
Supplement: S1 Text — The flux through each reaction is given for the ancestral and evolved strain. (DOCX) [file pcbi.1008433.s001.docx]

**S1_text**

PRODUCERS

##############################

12ppd__S

.......................................................

12PPDStex,0.0,-1.0

12PPDStpp,0.0,-1.0

AACTOOR,0.0,0.022343818251225667

ACONTa,1.2398589150000043,1.498830498820737

ACONTb,1.2398589150000041,1.498830498820737

AKGDH,1.024820515000004,1.28379880011819

AOBUTDs,0.0,0.022343818251225667

ATPS4rpp,13.370320970000053,13.80753408699773

CO2tex,-4.632830737500008,-5.8200493145056305

CO2tpp,-4.632830737500008,-5.819536160327867

CS,1.2398589150000043,1.4988315092245077

CYTBO3_4pp,8.410403675000031,8.784754496820648

DHAPT,0.32252132541666795,3.840539704924595e-06

ENO,3.220598515000006,3.50191734208122

F6PA,0.32252132541666795,7.181497009208098e-07

FBA3,0.19573216416666564,1.0897866598675663

FUM,1.2333893150000121,1.4923860642708837

G6PDH2r,0.7056018925000015,1.0815495573879834

GAPD,3.5642330149999975,3.8569702496477083

GHMT2r,0.22309610000000604,0.23451624960899498

GLCptspp,2.1404179895833364,2.744289209747344

GLCt2pp,0.0,0.09397938625371349

GLCtex_copy2,0.0,0.696851886597623

GLUDy,-1.7103415000000157,-1.7217356384735405

GLYAT,0.0,0.022343312917211033

GLYCL,0.010920900000001992,0.0

GND,0.7056018925000014,1.0815495105521333

H2Otex,-9.914783537500057,-10.101912936428288

H2Otpp,-9.914783537500059,-10.10194547527692

HEX1,0.0,0.09398258670346757

ICDHyr,1.2398589150000043,1.4988112961222124

LALDO3,0.0,1.0

LCARS,0.0,1.0

MDH,1.233197915000013,1.5146001085788776

MGSA,0.0,0.9776561817487743

MOX,0.00032519999999967104,-0.022063234526524655

NADH16pp,7.319156560000027,7.434121124166176

O2tex,4.206049537500015,4.3932681012504355

O2tpp,4.206049537500015,4.3932681012504355

PDH,1.8733075150000043,2.1546768296647247

PFK_3,0.19573216416666564,1.0897866598675663

PGCD,0.34363450000000595,0.35505347235173895

PGI,1.434816097083335,1.7567193586584633

PGK,-3.564233014999997,-3.8569702496477083

PGL,0.7056018925000015,1.0815495573879834

PGM,-3.2205985149999905,-3.50191734208122

PSERT,0.343634500000006,0.35505347235173895

PSP_L,0.343634500000006,0.35505347235173895

RPE,0.31519592833333127,0.5658329490071141

RPI,-0.38244516416667035,-0.5077557615454777

SUCDi,1.024954315000004,1.2853355323151576

SUCOAS,-0.9198485150000041,-1.1788297984363874

TALA,0.0,-0.7687359371880058

TKT1,0.1957321641666656,0.32105072267956025

TKT2,0.11946376416666554,0.24478222632755386

TPI,1.7028532254166657,1.2977281984432154

56 reacctions require a flux change for the evolution of the 12ppd__S producer

.......................................................

.......................................................

4abut

.......................................................

ABUTtex,0.0,-0.9999999999999999

ACONTa,1.2398589150000043,1.6158953505314124

ACONTb,1.2398589150000041,1.6158953505314124

AKGDH,1.024820515000004,0.40085695053141274

ATPS4rpp,13.370320970000053,13.61824729893132

CO2tex,-4.632830737500008,-5.072921826316462

CO2tpp,-4.632830737500008,-5.072921826316462

CS,1.2398589150000043,1.6158953505314124

CYTBO3_4pp,8.410403675000031,8.29058585264692

DHAPT,0.32252132541666795,3.200688048821121e-06

ENO,3.220598515000006,4.596634950519966

F6PA,0.32252132541666795,3.200688048821121e-06

FBA3,0.19573216416666564,1.1532715429497238

FUM,1.2333893150000121,0.6094257505306506

G6PDH2r,0.7056018925000015,1.017583674731151

GAPD,3.5642330149999975,4.940269450519691

GLCptspp,2.1404179895833364,2.838972549834054

GLCt2pp,0.0,0.041460621213090576

GLCtex_copy1,2.1404179895833364,2.8804331710471445

GLUABUTt7pp,0.0,1.001

GLUDC,0.0,1.000000000000567

GLUDy,-1.7103415000000157,-2.710341499997606

GLUt2rpp,0.0,-1.0019999999999998

GND,0.7056018925000014,1.017583674731151

H2Otex,-9.914783537500057,-11.354874626308272

H2Otpp,-9.914783537500059,-11.354874626307792

HEX1,0.0,0.040460621213090686

Htex,-1.8376463999999728,-2.8376463999969594

ICDHyr,1.2398589150000043,1.6158953505314124

MDH,1.233197915000013,0.6092343505306501

NADH16pp,7.319156560000027,7.821302302115555

NH4tex,2.160164800000022,3.1601647999964793

NH4tpp,2.160164800000022,3.1601647999964793

O2tex,4.206049537500015,4.14614062632346

O2tpp,4.206049537500015,4.14614062632346

PDH,1.8733075150000043,2.2493439505220034

PFK_3,0.19573216416666564,1.1532715429497238

PGI,1.434816097083335,1.8618494963159935

PGK,-3.564233014999997,-4.940269450519691

PGL,0.7056018925000015,1.017583674731151

PGM,-3.2205985149999905,-4.596634950519966

PPC,0.5918716000000012,1.591871599999228

RPE,0.31519592833333127,0.5231837831546022

RPI,-0.38244516416667035,-0.4864390915770067

SUCDi,1.024954315000004,0.40412455053136553

SUCOAS,-0.9198485150000041,-0.2958849505314127

TALA,0.0,-0.8535454513725291

TKT1,0.1957321641666656,0.29972609157719465

TKT2,0.11946376416666554,0.22345769157740758

TPI,1.7028532254166657,2.3388744794712295

50 reacctions require a flux change for the evolution of the 4abut producer

.......................................................

.......................................................

5dglcn

.......................................................

5DGLCNR,0.0,-1.0

5DGLCNt2rpp,0.0,-1.0

5DGLCNtex,0.0,-1.0

ACONTa,1.2398589150000043,1.1398591550139152

ACONTb,1.2398589150000041,1.1398591550139152

AKGDH,1.024820515000004,0.9248207550144639

ATPS4rpp,13.370320970000053,13.570319889964594

CO2tex,-4.632830737500008,-3.8828307375270015

CO2tpp,-4.632830737500008,-3.8828307375270015

CS,1.2398589150000043,1.1398591550139152

CYTBO3_4pp,8.410403675000031,8.910403675064067

DHAPT,0.32252132541666795,0.3455215654207878

ENO,3.220598515000006,3.1205987550050875

F6PA,0.32252132541666795,0.3455215654207877

FUM,1.2333893150000121,1.1333895550138946

G6PDH2r,0.7056018925000015,0.2556011724896599

GAPD,3.5642330149999975,3.4642332550050856

GLCDpp,0.0,1.0

GLCNt2rpp,0.0,1.0

GLCptspp,2.1404179895833364,2.0154179895831703

GLCtex_copy1,2.1404179895833364,3.01541798958336

GND,0.7056018925000014,0.2556011724896599

H2Otex,-9.914783537500057,-10.165283537519372

H2Otpp,-9.914783537500059,-11.164783537519156

Htex,-1.8376463999999728,-2.8366463999967664

ICDHyr,1.2398589150000043,1.139859155013915

MDH,1.233197915000013,1.1331969548304164

NADH16pp,7.319156560000027,6.917288919866173

O2tex,4.206049537500015,4.456299537532034

O2tpp,4.206049537500015,4.456049537532033

PDH,1.8733075150000043,1.7733077550074987

PGI,1.434816097083335,1.7598168170935105

PGK,-3.564233014999997,-3.4642332550050856

PGL,0.7056018925000015,0.2556011724896599

PGM,-3.2205985149999905,-3.1205987550050875

RPE,0.31519592833333127,0.015195448326817074

RPI,-0.38244516416667035,-0.23244492416330126

SUCDi,1.024954315000004,0.9279557551978955

SUCOAS,-0.9198485150000041,-0.8198487550145046

TALA,0.0,-0.15100024000336365

TKT1,0.1957321641666656,0.04573192416330207

TKT2,0.11946376416666554,-0.030536475836485

TPI,1.7028532254166657,1.727853465420785

43 reacctions require a flux change for the evolution of the 5dglcn producer

.......................................................

.......................................................

LalaDgluMdap

.......................................................

3PEPTtex,0.0,-1.0

ACGAMK,0.0,1.0

ACM6PH,0.0,1.0

ACONTa,1.2398589150000043,2.182793703457264

ACONTb,1.2398589150000041,2.182793703457264

ACS,0.0,2.0009992000000003

ADK1,0.524992600000009,2.527121999999963

AGDC,0.0,2.0

AGM3PApp,0.0,1.0

AGMH,0.0,1.0

AGMt2pp,0.0,1.0

AKGDH,1.024820515000004,0.9677553034572477

ALAALAabcpp,0.0,1.0

ALATA_L,-0.11627119999999999,-1.115271200000052

ANHMK,0.0,1.0

ASAD,-0.21381520000000004,-1.2138148000000228

ASPK,0.2138152,1.2138148000000228

ASPTA,-0.5855358000000094,-1.58553459999996

ATPS4rpp,13.370320970000053,23.48475115309975

CO2tex,-4.632830737500008,-7.489774008625091

CO2tpp,-4.632830737500008,-7.489774008625091

CS,1.2398589150000043,2.1827937034572473

CYTBO3_4pp,8.410403675000031,15.124292617250125

DAPE,0.07418980000000001,1.0741897999999992

DHAPT,0.32252132541666795,0.0

DHDPRy,0.07418980000000004,1.0741897999999999

DHDPS,0.07418980000000004,1.0741897999999999

ENO,3.220598515000006,8.163532103457214

F6PA,0.32252132541666795,0.0

FBA,1.2128609358333324,3.5010023578458913

FUM,1.2333893150000121,1.1763233034572522

G1PACT,0.018897600000000004,2.0188976

G6PDH2r,0.7056018925000015,3.7342084382750045

GAPD,3.5642330149999975,8.507009123449961

GLCptspp,2.1404179895833364,4.405873303457247

GLCt2pp,0.0,0.7106994979802383

GLCtex_copy2,0.0,2.9771548118541484

GLUDy,-1.7103415000000157,-5.710182819992724

GLUR,-0.005557600000000003,-1.0055575999999746

GND,0.7056018925000014,3.734208438274966

H2Otex,-9.914783537500057,-20.772219208624847

H2Otpp,-9.914783537500059,-22.77171920862486

HEX1,0.0,0.7106994979802383

Htex,-1.8376463999999728,-6.836644799999931

ICDHyr,1.2398589150000043,2.182793703457264

LDH_D,0.0,1.0

MCTP1Bpp,0.0,0.5

MDH,1.233197915000013,1.1761765034572136

MLDCP3App,0.0,0.5

MLDEP1pp,0.0,0.5

MLTGY3pp,0.0,0.5

MPTG,0.002778800000000001,0.5027788

NADH16pp,7.319156560000027,14.089110713792877

NDPK2,0.08505659999999997,2.08505660000003

NH4tex,2.160164800000022,6.1601619999999

NH4tpp,2.160164800000022,6.1601619999999

O2tex,4.206049537500015,7.56324400862502

O2tpp,4.206049537500015,7.562994008625083

PAPPT3,0.005557600000000001,1.0055576

PDH,1.8733075150000043,2.8159297434428376

PFK,1.2128609358333324,3.501002357845844

PGAMT,-0.018897600000000004,-2.0188975999999457

PGI,1.434816097083335,1.3823643631625373

PGK,-3.564233014999997,-8.507009123449961

PGL,0.7056018925000015,3.734208438274966

PGM,-3.2205985149999905,-8.163532103457214

PPC,0.5918716000000012,2.5918711999999973

PPK,-0.7007176000000049,-4.7028481999999485

RPE,0.31519592833333127,2.3342677588499328

RPI,-0.38244516416667035,-1.39197987942498

SDPDS,0.07418980000000003,1.0741897999999999

SDPTA,-0.07418980000000003,-1.0741897999999992

SUCDi,1.024954315000004,0.9700229034572481

SUCOAS,-0.9198485150000041,0.13721629654276057

TALA,0.0,1.008535915258335

THDPS,0.07418980000000003,1.0741897999999999

TKT1,0.1957321641666656,1.2052680794249682

TKT2,0.11946376416666554,1.1289996794249646

TPI,1.7028532254166657,3.66947332201255

UAAGDS,0.005557600000000004,1.0055576

UAGCVT,0.005557600000000004,1.0055576

UAGDP,0.018897600000000004,2.0188976

UAGPT3,0.005557600000000001,1.0055576

UAMAGS,0.005557600000000003,1.0055576

UAMAS,0.005557600000000003,1.0055576

UAPGR,0.005557600000000003,1.0055576

UDCPDP,0.005557600000000001,1.0055576

UGMDDS,0.005557600000000003,1.0055576

UMPK,0.07560779999999996,1.0756077999999434

89 reacctions require a flux change for the evolution of the LalaDgluMdap producer

.......................................................

.......................................................

LalaDgluMdapDala

.......................................................

4PEPTtex,0.0,-1.0

ACGAMK,0.0,1.0

ACM6PH,0.0,1.0

ACONTa,1.2398589150000043,1.950605169239907

ACONTb,1.2398589150000041,1.950605169239907

ACS,0.0,2.0009992

ADK1,0.524992600000009,2.5249893999999813

AGDC,0.0,2.0

AGM4PApp,0.0,1.0

AGMH,0.0,1.0

AGMt2pp,0.0,1.0

AKGDH,1.024820515000004,0.7355667692398651

ALAALAr,0.005557600000000003,1.0055575999999746

ALAR,0.008336400000000004,1.008336399999962

ALATA_L,-0.11627119999999999,-2.115271200000052

ANHMK,0.0,1.0

ASAD,-0.21381520000000004,-1.2138148000000228

ASPK,0.2138152,1.2138148000000228

ASPTA,-0.5855358000000094,-1.58553459999996

ATPS4rpp,13.370320970000053,22.948816861520186

CO2tex,-4.632830737500008,-7.409691873099632

CO2tpp,-4.632830737500008,-7.409691873099632

CS,1.2398589150000043,1.950605169239866

CYTBO3_4pp,8.410403675000031,14.964128346199288

DALAt2pp,0.0027788000000000005,1.0027788

DAPE,0.07418980000000001,1.0741897999999992

DHAPT,0.32252132541666795,0.0

DHDPRy,0.07418980000000004,1.0741897999999999

DHDPS,0.07418980000000004,1.0741897999999999

ENO,3.220598515000006,8.931343569239857

F6PA,0.32252132541666795,0.0

FBA3,0.19573216416666564,2.7671877545566304

FUM,1.2333893150000121,0.9441347692398949

G1PACT,0.018897600000000004,2.0188976

G6PDH2r,0.7056018925000015,4.350224865380028

GAPD,3.5642330149999975,9.2749762692398

GLCptspp,2.1404179895833364,5.173684769239864

GLCt2pp,0.0,0.4295410096100678

GLCtex_copy1,2.1404179895833364,5.60222577884997

GLUDy,-1.7103415000000157,-6.710338499999921

GLUR,-0.005557600000000003,-1.0055575999999746

GND,0.7056018925000014,4.350224865380061

H2Otex,-9.914783537500057,-22.692137073099616

H2Otpp,-9.914783537500059,-24.691637073099514

HEX1,0.0,0.42854100961006747

Htex,-1.8376463999999728,-7.836644799999931

ICDHyr,1.2398589150000043,1.950605169239907

LDH_D,0.0,1.0

MDDCP3pp,0.0,0.499

MDDCP4pp,0.0,0.5

MDH,1.233197915000013,0.9439433692398325

MLTGY1pp,0.0,0.5

MPTG,0.002778800000000001,0.5027788

NADH16pp,7.319156560000027,14.162134976959424

NDPK2,0.08505659999999997,2.08505660000003

NH4tex,2.160164800000022,7.160162000000014

NH4tpp,2.160164800000022,7.160162000000014

O2tex,4.206049537500015,7.483161873099675

O2tpp,4.206049537500015,7.482911873099624

PAPPT3,0.005557600000000001,1.0055576

PDH,1.8733075150000043,2.584052569239867

PFK_3,0.19573216416666564,2.7671877545565833

PGAMT,-0.018897600000000004,-2.0188975999999457

PGI,1.434816097083335,1.2520009134698284

PGK,-3.564233014999997,-9.2749762692398

PGL,0.7056018925000015,4.350224865380061

PGM,-3.2205985149999905,-8.931343569239857

PPC,0.5918716000000012,2.5918712000000026

PPK,-0.7007176000000049,-4.702714399999991

RPE,0.31519592833333127,2.7449453769201

RPI,-0.38244516416667035,-1.5973186884600636

SDPDS,0.07418980000000003,1.0741897999999999

SDPTA,-0.07418980000000003,-1.0741897999999992

SUCDi,1.024954315000004,0.7367005692398654

SUCOAS,-0.9198485150000041,0.3694048307601179

TALA,0.0,-1.3565808660965786

THDPS,0.07418980000000003,1.0741897999999999

TKT1,0.1957321641666656,1.4106068884600518

TKT2,0.11946376416666554,1.3343384884600482

TPI,1.7028532254166657,3.950787490389871

UAAGDS,0.005557600000000004,1.0055576

UAGCVT,0.005557600000000004,1.0055576

UAGDP,0.018897600000000004,2.0188976

UAGPT3,0.005557600000000001,1.0055576

UAMAGS,0.005557600000000003,1.0055576

UAMAS,0.005557600000000003,1.0055576

UAPGR,0.005557600000000003,1.0055576

UDCPDP,0.005557600000000001,1.0055575999999997

UGMDDS,0.005557600000000003,1.0055576

UMPK,0.07560779999999996,1.0756077999999434

90 reacctions require a flux change for the evolution of the LalaDgluMdapDala producer

.......................................................

.......................................................

ac

.......................................................

ACKr,0.11651519999999993,-0.8834848000019856

ACONTa,1.2398589150000043,0.689858914995106

ACONTb,1.2398589150000041,0.689858914995106

ACt2rpp,0.0,-1.0

ACtex,0.0,-1.0

AKGDH,1.024820515000004,0.474820514995106

ATPS4rpp,13.370320970000053,12.47032097000328

CO2tex,-4.632830737500008,-4.257830737533411

CO2tpp,-4.632830737500008,-4.257830737533411

CS,1.2398589150000043,0.689858914995106

CYTBO3_4pp,8.410403675000031,7.660403675082237

DHAPT,0.32252132541666795,0.500687992649509

ENO,3.220598515000006,3.6705985148684106

F6PA,0.32252132541666795,0.500687992649509

FUM,1.2333893150000121,0.6833893149943656

G6PDH2r,0.7056018925000015,0.980601892557277

GAPD,3.5642330149999975,4.014233014983714

GLCptspp,2.1404179895833364,2.4112513222210623

GLCtex_copy1,2.1404179895833364,2.411251322916845

GND,0.7056018925000014,0.980601892557277

H2Otex,-9.914783537500057,-9.540283537527

H2Otpp,-9.914783537500059,-9.53978353752652

Htex,-1.8376463999999728,-2.8366463999981217

ICDHyr,1.2398589150000043,0.689858914995106

MDH,1.233197915000013,0.6831979149943651

NADH16pp,7.319156560000027,7.119156560087131

O2tex,4.206049537500015,3.8312995375411183

O2tpp,4.206049537500015,3.8310495375411184

PDH,1.8733075150000043,2.3233075148700673

PGK,-3.564233014999997,-4.014233014983714

PGL,0.7056018925000015,0.980601892557277

PGM,-3.2205985149999905,-3.6705985148684106

PTAr,-0.11651519999999993,0.8834848000019856

RPE,0.31519592833333127,0.49852926170534606

RPI,-0.38244516416667035,-0.4741118308523893

SUCDi,1.024954315000004,0.474954314995106

SUCOAS,-0.9198485150000041,-0.3708485149956733

TALA,0.0,0.09066666668590093

TKT1,0.1957321641666656,0.28739883085256657

TKT2,0.11946376416666554,0.2111304308527795

TPI,1.7028532254166657,1.8820198920655622

41 reacctions require a flux change for the evolution of the ac producer

.......................................................

.......................................................

acald

.......................................................

ACALD,0.0,-1.0

ACALDtex,0.0,-1.0

ACALDtpp,0.0,-1.0

ACONTa,1.2398589150000043,0.9648589150166148

ACONTb,1.2398589150000041,0.9648589150166148

AKGDH,1.024820515000004,0.7498205150165962

ATPS4rpp,13.370320970000053,12.92032096996461

CO2tex,-4.632830737500008,-4.945330737529754

CO2tpp,-4.632830737500008,-4.945330737529754

CS,1.2398589150000043,0.9648589150166148

CYTBO3_4pp,8.410403675000031,8.035403675074814

DHAPT,0.32252132541666795,0.6621046587543219

ENO,3.220598515000006,3.945598515005972

F6PA,0.32252132541666795,0.6621046587543219

FUM,1.2333893150000121,0.9583893150164021

G6PDH2r,0.7056018925000015,0.8431018924899994

GAPD,3.5642330149999975,4.2892330150056965

GLCptspp,2.1404179895833364,2.5258346556321647

GLCtex_copy1,2.1404179895833364,2.5258346562500975

GND,0.7056018925000014,0.8431018924899994

H2Otex,-9.914783537500057,-10.227283537524372

H2Otpp,-9.914783537500059,-10.227283537523892

ICDHyr,1.2398589150000043,0.9648589150166148

MDH,1.233197915000013,0.9581979150164017

NADH16pp,7.319156560000027,7.218156560058241

O2tex,4.206049537500015,4.018549537537407

O2tpp,4.206049537500015,4.018549537537407

PDH,1.8733075150000043,2.598307515007206

PGI,1.434816097083335,1.6827327631421651

PGK,-3.564233014999997,-4.2892330150056965

PGL,0.7056018925000015,0.8431018924899994

PGM,-3.2205985149999905,-3.945598515005972

RPE,0.31519592833333127,0.4068625949938678

RPI,-0.38244516416667035,-0.4282784974966489

SUCDi,1.024954315000004,0.7520881150165726

SUCOAS,-0.9198485150000041,-0.6448485150165962

TALA,0.0,0.0468333333302553

TKT1,0.1957321641666656,0.24156549749693568

TKT2,0.11946376416666554,0.1652970974969321

TPI,1.7028532254166657,2.0424365587544955

40 reacctions require a flux change for the evolution of the acald producer

.......................................................

.......................................................

ade

.......................................................

ACONTa,1.2398589150000043,1.2904200716764354

ACONTb,1.2398589150000041,1.2904200716764354

ADEt2rpp,0.0,-1.0

ADEtex,0.0,-1.0

ADK3,0.0,1.0009999999990675

ADSL1r,0.059807400000004014,1.0598073999999968

ADSL2r,0.08960300000000408,1.0896029999999968

ADSS,0.059807400000004014,1.0598073999999968

AICART,0.10855060000000409,1.1085505999999967

AIRC2,0.08960300000000408,1.0896029999999968

AIRC3,-0.08960300000000408,-1.0896029999999968

AKGDH,1.024820515000004,1.0753816716769842

AMPN,0.0,0.99899999999982

ASPTA,-0.5855358000000094,-2.585535799999812

ATPS4rpp,13.370320970000053,22.26906325664188

CO2tex,-4.632830737500008,-5.009233629183072

CO2tpp,-4.632830737500008,-5.009233629183072

CS,1.2398589150000043,1.2904200716764354

CYTBO3_4pp,8.410403675000031,14.163209458377299

DHAPT,0.32252132541666795,3.2005222587727644e-06

ENO,3.220598515000006,3.2711596716682223

F6PA,0.32252132541666795,3.2005222587727644e-06

FBA3,0.19573216416666564,1.1717442971184433

FUM,1.2333893150000121,3.2839504716769783

G6PDH2r,0.7056018925000015,1.4303213141598632

GAPD,3.5642330149999975,5.114794171668218

GARFT,0.08915700000000398,1.089156999999997

GHMT2r,0.22309610000000604,1.7230960999999954

GLCptspp,2.1404179895833364,2.5134972711480588

GLCt2pp,0.0,0.5229878670450416

GLCtex_copy2,0.0,0.8950671486097641

GLNS,0.36212300000000835,2.3621229999995355

GLUDy,-1.7103415000000157,-5.210341499998864

GLUPRT,0.08964760000000407,1.0896475999999968

GLYCL,0.010920900000001992,0.5109208999999986

GND,0.7056018925000014,1.4303213141598632

H2Otex,-9.914783537500057,-20.291186429178307

H2Otpp,-9.914783537500059,-20.291186429178065

HCO3E,0.10516900000000409,1.1051681999990801

HEX1,0.0,0.5229878670450416

Htex,-1.8376463999999728,-6.837646399998527

ICDHyr,1.2398589150000043,1.2904200716764354

IMPC,-0.10855060000000409,-1.1085505999999967

MDH,1.233197915000013,3.283803671676978

MTHFC,0.1977522000000081,2.197752199999994

MTHFD,0.1977522000000081,2.197752199999994

NADH16pp,7.319156560000027,13.021401186700313

NDPK1,0.158125000000004,2.1591249999990643

NH4tex,2.160164800000022,7.160164799998607

NH4tpp,2.160164800000022,7.160164799998607

O2tex,4.206049537500015,7.082452429188649

O2tpp,4.206049537500015,7.082452429188649

PDH,1.8733075150000043,1.9238686716700186

PFK_3,0.19573216416666564,1.1717442971184433

PGCD,0.34363450000000595,1.8436344999999956

PGI,1.434816097083335,1.6061638240332372

PGK,-3.564233014999997,-5.114794171668218

PGL,0.7056018925000015,1.4303213141598632

PGM,-3.2205985149999905,-3.2711596716682223

PPK,-0.7007176000000049,-1.7007175999972028

PRAGSr,0.08964760000000409,1.0896475999999968

PRAIS,0.08964760000000409,1.0896475999999968

PRASCSi,0.0896030000000041,1.0896029999999968

PRFGS,0.08964760000000407,1.0896475999999968

PRPPS,0.1865784000000047,1.1865783999999964

PSERT,0.343634500000006,1.8436344999999956

PSP_L,0.343634500000006,1.8436344999999956

RPE,0.31519592833333127,0.7983422094402878

RPI,-0.38244516416667035,-0.6240183047200338

SUCDi,1.024954315000004,1.0755154716769844

SUCOAS,-0.9198485150000041,-0.9704096716769597

TALA,0.0,-0.7344389923984058

TKT1,0.1957321641666656,0.4373053047200374

TKT2,0.11946376416666554,0.36103690472025035

TPI,1.7028532254166657,2.357347233473984

75 reacctions require a flux change for the evolution of the ade producer

.......................................................

.......................................................

adn

.......................................................

ACONTa,1.2398589150000043,1.409524140903045

ACONTb,1.2398589150000041,1.409524140903045

ADK1,0.524992600000009,1.5260673399959614

ADNt2pp_copy2,0.0,-1.0

ADNtex,0.0,-1.0

ADSL1r,0.059807400000004014,1.059806999999978

ADSL2r,0.08960300000000408,1.0896026000000347

ADSS,0.059807400000004014,1.0598069999999995

AICART,0.10855060000000409,1.1085501999999678

AIRC2,0.08960300000000408,1.0896025999999996

AIRC3,-0.08960300000000408,-1.0896026000000347

AKGDH,1.024820515000004,1.1944857409030891

ASPTA,-0.5855358000000094,-2.58553459999996

ATPS4rpp,13.370320970000053,23.030922258189776

CO2tex,-4.632830737500008,-5.306989302257762

CO2tpp,-4.632830737500008,-5.306989302257762

CS,1.2398589150000043,1.4095241409030894

CYTBO3_4pp,8.410403675000031,14.758723204515444

DHAPT,0.32252132541666795,0.0

ENO,3.220598515000006,3.390262540903109

F6PA,0.32252132541666795,0.0

FBA,1.2128609358333324,2.427019259693452

FUM,1.2333893150000121,3.403053740903033

G6PDH2r,0.7056018925000015,1.370765379548402

GAPD,3.5642330149999975,5.233895240903053

GARFT,0.08915700000000398,1.0896471999999449

GHMT2r,0.22309610000000604,1.7230951000000232

GLCptspp,2.1404179895833364,2.6326037409030896

GLCt2pp,0.0,1.2868382761398642

GLCtex_copy2,0.0,1.7800240274596173

GLNS,0.36212300000000835,2.3621222

GLUDy,-1.7103415000000157,-5.210338500000034

GLUPRT,0.08964760000000407,1.0896471999999997

GLYCL,0.010920900000001992,0.5109202999999999

GND,0.7056018925000014,1.3707653795484556

H2Otex,-9.914783537500057,-21.589434502257745

H2Otpp,-9.914783537500059,-21.588934502257757

HCO3E,0.10516900000000409,1.1051674000000276

HEX1,0.0,1.2858382761398643

Htex,-1.8376463999999728,-6.836644799999931

ICDHyr,1.2398589150000043,1.409524140903045

IMPC,-0.10855060000000409,-1.1085501999999678

MDH,1.233197915000013,3.4028623409030843

MTHFC,0.1977522000000081,2.1977514000000156

MTHFD,0.1977522000000081,2.1977514000000156

NADH16pp,7.319156560000027,13.497810863612356

NDPK1,0.158125000000004,1.1571238000000221

NH4tex,2.160164800000022,7.160162000000014

NH4tpp,2.160164800000022,7.160162000000014

NTD7,0.0,0.9999433399959206

O2tex,4.206049537500015,7.380459302257691

O2tpp,4.206049537500015,7.380209302257754

PDH,1.8733075150000043,2.0429715409030895

PFK,1.2128609358333324,2.4270192596934694

PGCD,0.34363450000000595,1.8436326999999997

PGI,1.434816097083335,2.5476766374945328

PGK,-3.564233014999997,-5.233895240903053

PGL,0.7056018925000015,1.3707653795484556

PGM,-3.2205985149999905,-3.390262540903109

PPK,-0.7007176000000049,-1.7007923399959282

PRAGSr,0.08964760000000409,1.0896471999999449

PRAIS,0.08964760000000409,1.0896471999999997

PRASCSi,0.0896030000000041,1.0896025999999996

PRFGS,0.08964760000000407,1.0896471999999997

PRPPS,0.1865784000000047,1.186655139995878

PSERT,0.343634500000006,1.8436326999999997

PSP_L,0.343634500000006,1.8436326999999997

RPE,0.31519592833333127,0.09197238636568272

RPI,-0.38244516416667035,-1.270832193182855

SUCDi,1.024954315000004,1.1957533409030892

SUCOAS,-0.9198485150000041,-1.089514140903134

TALA,0.0,-0.11061177098383723

TKT1,0.1957321641666656,0.08412039318284315

TKT2,0.11946376416666554,0.007851993182839578

TPI,1.7028532254166657,2.593490223860158

75 reacctions require a flux change for the evolution of the adn producer

.......................................................

.......................................................

agm

.......................................................

ACGK,0.05915840000000002,1.0591584

ACGS,0.05915840000000002,1.0591584

ACODA,0.059158400000000014,1.0591584

ACONTa,1.2398589150000043,1.917743399811346

ACONTb,1.2398589150000041,1.917743399811346

ACOTA,-0.05915840000000002,-1.0591584

ACS,0.0,1.0009999999985815

ADK1,0.524992600000009,2.527127199997298

AGMtex,0.0,-1.0

AGPR,-0.05915840000000003,-1.0591584

AKGDH,1.024820515000004,0.702704999811327

ARGAGMt7pp,0.0,1.0

ARGDC,0.0,1.0

ARGSL,0.05915840000000001,1.0591584

ARGSS,0.05915840000000001,1.0591584

ARGt3pp,0.0,1.0

ASPTA,-0.5855358000000094,-1.5855357999998023

ATPS4rpp,13.370320970000053,17.014552000375623

CBMKr,0.12531739999999997,1.1253174000000001

CO2tex,-4.632830737500008,-6.327541949517193

CO2tpp,-4.632830737500008,-6.327541949517193

CS,1.2398589150000043,1.917743399811346

CYTBO3_4pp,8.410403675000031,10.799826099049852

DHAPT,0.32252132541666795,6.356231097015552e-10

ENO,3.220598515000006,4.8984829998008985

F6PA,0.32252132541666795,6.356231097015552e-10

FBA,1.2128609358333324,2.0984815434163657

FUM,1.2333893150000121,1.9112737998113123

G6PDH2r,0.7056018925000015,2.3666596500927954

GAPD,3.5642330149999975,5.242117499800711

GLCptspp,2.1404179895833364,3.140823799166803

GLCt2pp,0.0,0.11537939241460782

GLCtex_copy1,2.1404179895833364,3.256203191581411

GLUDy,-1.7103415000000157,-4.71034149999924

GND,0.7056018925000014,2.3666596500927954

H2Otex,-9.914783537500057,-15.609494749512043

H2Otpp,-9.914783537500059,-15.609494749511564

HEX1,0.0,0.11537939241460782

Htex,-1.8376463999999728,-3.837646399999262

ICDHyr,1.2398589150000043,1.917743399811346

MDH,1.233197915000013,1.9111269998113118

NADH16pp,7.319156560000027,10.028828299238526

NH4tex,2.160164800000022,6.160164799998976

NH4tpp,2.160164800000022,6.160164799998976

O2tex,4.206049537500015,5.400760749524926

O2tpp,4.206049537500015,5.400760749524926

OCBT,0.05915840000000002,1.0591584

PDH,1.8733075150000043,2.551191999802128

PFK,1.2128609358333324,2.0984815434163657

PGI,1.434816097083335,0.8895435414886155

PGK,-3.564233014999997,-5.242117499800711

PGL,0.7056018925000015,2.3666596500927954

PGM,-3.2205985149999905,-4.8984829998008985

PPC,0.5918716000000012,1.5918715999998363

PPK,-0.7007176000000049,-2.7018521999952534

RPE,0.31519592833333127,1.4225677667289125

RPI,-0.38244516416667035,-0.9361310833643415

SUCDi,1.024954315000004,0.7058387998113259

SUCOAS,-0.9198485150000041,-0.5977329998113023

TALA,0.0,0.5546859191983516

TKT1,0.1957321641666656,0.7494180833643498

TKT2,0.11946376416666554,0.6731496833645627

TPI,1.7028532254166657,2.2649525082181627

63 reacctions require a flux change for the evolution of the agm producer

.......................................................

.......................................................

akg

.......................................................

ACONTa,1.2398589150000043,1.6398591550227313

ACONTb,1.2398589150000041,1.6398591550227313

AKGDH,1.024820515000004,0.42482075502271316

AKGt2rpp,0.0,-1.0

AKGtex,0.0,-1.0

ATPS4rpp,13.370320970000053,13.570319889953407

CO2tex,-4.632830737500008,-3.6328307375414743

CO2tpp,-4.632830737500008,-3.6328307375414743

CS,1.2398589150000043,1.6398591550227313

DHAPT,0.32252132541666795,0.05585849960672418

ENO,3.220598515000006,4.620598755008102

F6PA,0.32252132541666795,0.05585849960672418

FBA,1.2128609358333324,2.211857334982553

FRD3,0.0,0.5969997599772909

FUM,1.2333893150000121,0.6333895550221555

G6PDH2r,0.7056018925000015,0.5056011724855994

GAPD,3.5642330149999975,4.964233255007743

GLCptspp,2.1404179895833364,2.8070810554034704

GLCtex_copy2,0.0,0.6656666666669744

GND,0.7056018925000014,0.5056011724855994

H2Otex,-9.914783537500057,-10.915283537534044

H2Otpp,-9.914783537500059,-10.914783537533804

Htex,-1.8376463999999728,-3.8366463999978078

ICDHyr,1.2398589150000043,1.6398591550227313

MDH,1.233197915000013,0.633242755022156

NADH18pp,0.0,0.5969997599772909

PDH,1.8733075150000043,2.273307755010278

PFK,1.2128609358333324,2.211857334982553

PGI,1.434816097083335,2.301479882917871

PGK,-3.564233014999997,-4.964233255007743

PGL,0.7056018925000015,0.5056011724855994

PGM,-3.2205985149999905,-4.620598755008102

PPC,0.5918716000000012,1.591871599999271

RPE,0.31519592833333127,0.1818621149907727

RPI,-0.38244516416667035,-0.315778257495285

SUCOAS,-0.9198485150000041,-0.3208487550226886

TALA,0.0,-0.06766690667138575

TKT1,0.1957321641666656,0.1290652574952799

TKT2,0.11946376416666554,0.05279685749549282

TPI,1.7028532254166657,2.4361867987563075

40 reacctions require a flux change for the evolution of the akg producer

.......................................................

.......................................................

ala__D

.......................................................

ACGAMK,0.0,0.49861060002000207

ACKr,0.11651519999999993,1.1137364000395458

ACM6PH,0.0,0.49861060002000207

ACONTa,1.2398589150000043,1.3637473950179138

ACONTb,1.2398589150000041,1.3637473950179138

AGDC,0.0,0.9972212000400041

AGM3PH,0.0,0.49861060002000207

AGM3Pt2pp,0.0,0.49861060002000207

AGM4PCPpp,0.0,0.4976106000200257

AKGDH,1.024820515000004,1.1487089950178955

ALAALAr,0.005557600000000003,0.5041682000200021

ALAR,0.008336400000000004,1.0083364000000001

ALATA_L,-0.11627119999999999,-1.1152712

AM3PA,0.0,0.49861060002000207

ANHMK,0.0,0.49861060002000207

ATPS4rpp,13.370320970000053,16.61003941014732

CO2tex,-4.632830737500008,-5.69185723753967

CO2tpp,-4.632830737500008,-5.69185723753967

CS,1.2398589150000043,1.3637473950179138

CYTBO3_4pp,8.410403675000031,10.528456675099987

DALAt2pp,0.0027788000000000005,4.000411113480595e-11

DALAtex,0.0,-1.0

DHAPT,0.32252132541666795,0.27129478873489454

ENO,3.220598515000006,4.344486995003284

F6PA,0.32252132541666795,0.27129478873489454

FBA3,0.19573216416666564,0.6933427641832453

FUM,1.2333893150000121,1.3572777950173267

G1PACT,0.018897600000000004,1.0161188000395458

G6PDH2r,0.7056018925000015,1.3929629524982452

GAPD,3.5642330149999975,4.6881214950029175

GLCptspp,2.1404179895833364,2.8169224062500007

GLCtex_copy2,0.0,0.6755044166664086

GLUDy,-1.7103415000000157,-2.71034149999775

GND,0.7056018925000014,1.3929629524982452

H2Otex,-9.914783537500057,-11.973810037532168

H2Otpp,-9.914783537500059,-12.971031237572172

Htex,-1.8376463999999728,-2.8376463999977615

ICDHyr,1.2398589150000043,1.3637473950179138

LDH_D,0.0,0.49861060002000207

MDDCP3pp,0.0,0.24930530001000104

MDDCP4pp,0.0,0.24930530001000104

MDH,1.233197915000013,1.3571309950173271

MLTGY1pp,0.0,0.24830530001002468

MPTG,0.002778800000000001,0.25208410001000103

NADH16pp,7.319156560000027,9.31332108008209

NDPK2,0.08505659999999997,1.0822778000395459

NH4tex,2.160164800000022,3.1601647999969296

NH4tpp,2.160164800000022,3.1601647999969296

O2tex,4.206049537500015,5.265076037549993

O2tpp,4.206049537500015,5.265076037549993

PAPPT3,0.005557600000000001,0.5041682000200021

PDH,1.8733075150000043,1.9971959950054603

PFK_3,0.19573216416666564,0.6933427641832453

PGAMT,-0.018897600000000004,-1.0161188000395458

PGK,-3.564233014999997,-4.6881214950029175

PGL,0.7056018925000015,1.3929629524982452

PGM,-3.2205985149999905,-4.344486995003284

PPK,-0.7007176000000049,-1.698073400036508

PTAr,-0.11651519999999993,-1.1137364000395458

RPE,0.31519592833333127,0.7734366349992071

RPI,-0.38244516416667035,-0.6115655174994965

SUCDi,1.024954315000004,1.1498427950178955

SUCOAS,-0.9198485150000041,-1.043736995017871

TALA,0.0,-0.2684902466837481

TKT1,0.1957321641666656,0.4248525174994971

TKT2,0.11946376416666554,0.34858411749971

TPI,1.7028532254166657,2.150237288751786

UAGCVT,0.005557600000000004,0.5041682000200021

UAGDP,0.018897600000000004,1.0161188000395458

UAGPT3,0.005557600000000001,0.5041682000200021

UAPGR,0.005557600000000003,0.5041682000200021

UDCPDP,0.005557600000000001,0.5041682000200021

UGMDDS,0.005557600000000003,0.5041682000200021

UM3PL,0.0,0.49861060002000207

UMPK,0.07560779999999996,0.5742184000197729

75 reacctions require a flux change for the evolution of the ala__D producer

.......................................................

.......................................................

ala__L

.......................................................

ACONTa,1.2398589150000043,0.9648589150149423

ACONTb,1.2398589150000041,0.9648589150149423

AKGDH,1.024820515000004,0.7498205150154914

ALATA_L,-0.11627119999999999,-1.1152711999999998

ALAt2pp_copy2,-0.001041,-1.000041

ALAtex,0.0,-1.0

ATPS4rpp,13.370320970000053,12.920320969963239

CO2tex,-4.632830737500008,-4.445330737528298

CO2tpp,-4.632830737500008,-4.445330737528298

CS,1.2398589150000043,0.9648589150149423

CYTBO3_4pp,8.410403675000031,8.035403675067952

DHAPT,0.32252132541666795,0.57877132600455

ENO,3.220598515000006,3.945598515005541

F6PA,0.32252132541666795,0.57877132600455

FUM,1.2333893150000121,0.9583893150152814

G6PDH2r,0.7056018925000015,1.3431018924890925

GAPD,3.5642330149999975,4.289233015005428

GLCptspp,2.1404179895833364,2.6091679890036765

GLCtex_copy1,2.1404179895833364,2.609167989583085

GLUDy,-1.7103415000000157,-2.7103414999969977

GND,0.7056018925000014,1.3431018924890925

H2Otex,-9.914783537500057,-10.727283537520908

H2Otpp,-9.914783537500059,-10.727283537520668

Htex,-1.8376463999999728,-2.8376464000005446

ICDHyr,1.2398589150000043,0.9648589150149423

MDH,1.233197915000013,0.9581979150152813

NADH16pp,7.319156560000027,7.219156560052461

NH4tex,2.160164800000022,3.160164799996633

NH4tpp,2.160164800000022,3.160164799996633

O2tex,4.206049537500015,4.018549537533976

O2tpp,4.206049537500015,4.018549537533976

PDH,1.8733075150000043,1.5983075150083348

PGI,1.434816097083335,1.2660660970939925

PGK,-3.564233014999997,-4.289233015005428

PGL,0.7056018925000015,1.3431018924890925

PGM,-3.2205985149999905,-3.945598515005541

RPE,0.31519592833333127,0.7401959283265687

RPI,-0.38244516416667035,-0.5949451641629824

SUCDi,1.024954315000004,0.7500881150154914

SUCOAS,-0.9198485150000041,-0.6448485150154915

TALA,0.0,0.2114999999965122

TKT1,0.1957321641666656,0.40823216416317787

TKT2,0.11946376416666554,0.33196376416339074

TPI,1.7028532254166657,1.9591032254210194

44 reacctions require a flux change for the evolution of the ala__L producer

.......................................................

.......................................................

alaala

.......................................................

ACGAMK,0.0,1.0

ACM6PH,0.0,1.0

ACONTa,1.2398589150000043,1.4896833007924215

ACONTb,1.2398589150000041,1.4896833007924215

ACS,0.0,1.9989992

ADK1,0.524992600000009,2.523124400000029

AGDC,0.0,2.0

AGM3PA,0.0,1.0

AGM3Pt2pp,0.0,1.0

AGMH,0.0,1.0

AKGDH,1.024820515000004,1.2746449007923704

ALAALAr,0.005557600000000003,1.0055575999999746

ALAALAtex,0.0,-1.0

ALAR,0.008336400000000004,2.008336399999962

ALATA_L,-0.11627119999999999,-2.115271200000052

ANHMK,0.0,1.0

ATPS4rpp,13.370320970000053,19.870655242911994

CO2tex,-4.632830737500008,-6.7578751835582125

CO2tpp,-4.632830737500008,-6.7578751835582125

CS,1.2398589150000043,1.4896833007923713

CYTBO3_4pp,8.410403675000031,12.660519003961857

DHAPT,0.32252132541666795,0.21817223533077512

ENO,3.220598515000006,5.4704156915810245

F6PA,0.32252132541666795,0.21817223533082597

FBA,1.2128609358333324,2.211851526622013

FUM,1.2333893150000121,1.4832129007924095

G1PACT,0.018897600000000004,2.0188976

G6PDH2r,0.7056018925000015,2.0811797903924116

GAPD,3.5642330149999975,5.814048391580968

GLCptspp,2.1404179895833364,3.494584656250211

GLCtex_copy1,2.1404179895833364,3.493584656250164

GLUDy,-1.7103415000000157,-3.7103385000000344

GND,0.7056018925000014,2.081179790392433

H2Otex,-9.914783537500057,-15.039814374346747

H2Otpp,-9.914783537500059,-16.039814374346747

Htex,-1.8376463999999728,-3.8376453462919926

ICDHyr,1.2398589150000043,1.4896833007924215

LDH_D,0.0,1.0

MCTP1Bpp,0.0,0.5

MDH,1.233197915000013,1.483066100792371

MLDCP3App,0.0,0.5

MLDEP1pp,0.0,0.5

MLTGY3pp,0.0,0.5

MPTG,0.002778800000000001,0.5027788

NADH16pp,7.319156560000027,11.319447503169487

NDPK2,0.08505659999999997,2.0840614092113583

NH4tex,2.160164800000022,4.160162000000014

NH4tpp,2.160164800000022,4.160162000000014

O2tex,4.206049537500015,6.331107201980899

O2tpp,4.206049537500015,6.331107201980899

PAPPT3,0.005557600000000001,1.0055576

PDH,1.8733075150000043,2.1241307007923704

PFK,1.2128609358333324,2.2118515266219574

PGAMT,-0.018897600000000004,-2.0188975999999457

PGI,1.434816097083335,1.4134048658577285

PGK,-3.564233014999997,-5.814048391580968

PGL,0.7056018925000015,2.081179790392433

PGM,-3.2205985149999905,-5.4704156915810245

PPK,-0.7007176000000049,-4.700837927869202

RPE,0.31519592833333127,1.2322486602615754

RPI,-0.38244516416667035,-0.8409703301308582

SUCDi,1.024954315000004,1.2759125007923708

SUCOAS,-0.9198485150000041,-1.1696733007923967

TALA,0.0,0.45752636596409957

TKT1,0.1957321641666656,0.6542585301308463

TKT2,0.11946376416666554,0.5779901301308428

TPI,1.7028532254166657,2.598494726119384

UAGCVT,0.005557600000000004,1.0055576

UAGDP,0.018897600000000004,2.0188976

UAGPT3,0.005557600000000001,1.0055576

UAPGR,0.005557600000000003,1.0055576

UDCPDP,0.005557600000000001,1.005557053708056

UGMDDS,0.005557600000000003,1.0055576

UM3PL,0.0,1.0

UMPK,0.07560779999999996,1.0756066000000146

75 reacctions require a flux change for the evolution of the alaala producer

.......................................................

.......................................................

alltn

.......................................................

ACONTa,1.2398589150000043,0.8202074655229016

ACONTb,1.2398589150000041,0.8202074655229016

ADSL2r,0.08960300000000408,1.0896029999999843

AICART,0.10855060000000409,1.1085505999998038

AIRC2,0.08960300000000408,1.0896029999999843

AIRC3,-0.08960300000000408,-1.0896029999999843

AKGDH,1.024820515000004,0.6051690655228831

ALLTNt2rpp,0.0,-1.0

ALLTNtex,0.0,-1.0

ASPTA,-0.5855358000000094,-1.5855357999984732

ATPS4rpp,13.370320970000053,22.2096238689483

CO2tex,-4.632830737500008,-4.3337021137939695

CO2tpp,-4.632830737500008,-4.3337021137939695

CS,1.2398589150000043,0.8202074655229016

CYTBO3_4pp,8.410403675000031,13.812146427604384

DHAPT,0.32252132541666795,7.581629768438347e-10

ENO,3.220598515000006,2.800947065511073

F6PA,0.32252132541666795,7.581629768438347e-10

FBA3,0.19573216416666564,0.9807901432911219

FUM,1.2333893150000121,1.813737865522104

G6PDH2r,0.7056018925000015,1.1654276172352547

GAPD,3.5642330149999975,4.6445815655107685

GARFT,0.08915700000000398,1.0891569999999842

GHMT2r,0.22309610000000604,1.723096099999886

GLCptspp,2.1404179895833364,2.0432878647550035

GLCt2pp,0.0,0.713942020871682

GLCtex_copy1,2.1404179895833364,2.7572298856266855

GLNS,0.36212300000000835,2.36212299999933

GLUDy,-1.7103415000000157,-4.210341499997422

GLUPRT,0.08964760000000407,1.0896475999999844

GLYCL,0.010920900000001992,0.5109208999999018

GND,0.7056018925000014,1.1654276172352547

H2Otex,-9.914783537500057,-16.61615491378718

H2Otpp,-9.914783537500059,-16.615654913786702

HCO3E,0.10516900000000409,1.1051689999990677

HEX1,0.0,0.713942020871682

Htex,-1.8376463999999728,-5.836646399998325

ICDHyr,1.2398589150000043,0.8202074655229016

IMPC,-0.10855060000000409,-1.1085505999998038

IMPD,0.048743200000000084,1.0487432

MDH,1.233197915000013,2.8135910655221035

MOX,0.00032519999999967104,-0.9997193999999996

MTHFC,0.1977522000000081,2.1977521999997878

MTHFD,0.1977522000000081,2.1977521999997878

NADH16pp,7.319156560000027,13.1405507620815

NH4tex,2.160164800000022,6.160164799997623

NH4tpp,2.160164800000022,6.160164799997623

NTD10,0.0,1.0

O2tex,4.206049537500015,6.907170913802192

O2tpp,4.206049537500015,6.906920913802192

PDH,1.8733075150000043,1.4536560655132493

PFK_3,0.19573216416666564,0.9807901432911219

PGCD,0.34363450000000595,1.8436344999996959

PGI,1.434816097083335,1.5918022683914308

PGK,-3.564233014999997,-4.6445815655107685

PGL,0.7056018925000015,1.1654276172352547

PGM,-3.2205985149999905,-2.800947065511073

PPK,-0.7007176000000049,-1.7007175999956903

PRAGSr,0.08964760000000409,1.0896475999999844

PRAIS,0.08964760000000409,1.0896475999999844

PRASCSi,0.0896030000000041,1.0896029999999843

PRFGS,0.08964760000000407,1.0896475999999844

PSERT,0.343634500000006,1.8436344999996959

PSP_L,0.343634500000006,1.8436344999996959

PUNP7,0.0,1.0

R15BPK,0.0,0.999

R1PK,0.0,0.999

RPE,0.31519592833333127,0.6217464114906774

RPI,-0.38244516416667035,-0.5357204057450355

SUCDi,1.024954315000004,0.6054366655228831

SUCOAS,-0.9198485150000041,-0.5001970655234258

TALA,0.0,-0.6317827375458896

TKT1,0.1957321641666656,0.34900740574523226

TKT2,0.11946376416666554,0.2727390057454452

TPI,1.7028532254166657,2.166389879882756

URIC,0.0,1.0

XAND,0.0,1.0

77 reacctions require a flux change for the evolution of the alltn producer

.......................................................

.......................................................

anhgm

.......................................................

3PEPTabcpp,0.0,1.0

ACONTa,1.2398589150000043,1.172210695639592

ACONTb,1.2398589150000041,1.172210695639592

AGM3PApp,0.0,1.0

AKGDH,1.024820515000004,0.9571722956396093

ALAALAabcpp,0.0,1.0

ANHGMtex,0.0,-1.0

ATPS4rpp,13.370320970000053,20.505594579971444

CO2tex,-4.632830737500008,-6.9641650664212875

CO2tpp,-4.632830737500008,-6.9641650664212875

CS,1.2398589150000043,1.1722106956396094

CYTBO3_4pp,8.410403675000031,13.073117566054862

DHAPT,0.32252132541666795,0.0

ENO,3.220598515000006,6.1529371873365335

F6PA,0.32252132541666795,0.0

FBA3,0.19573216416666564,1.8963672268021128

FUM,1.2333893150000121,1.16574029563958

G1PACT,0.018897600000000004,2.0188976

G6PDH2r,0.7056018925000015,1.239894361287611

GAPD,3.5642330149999975,6.496569887336477

GF6PTA,0.018897600000000007,2.0188976

GLCptspp,2.1404179895833364,4.395278387336516

GLCt2pp,0.0,1.3003498555793713

GLCtex_copy2,0.0,3.556210253332551

GLNS,0.36212300000000835,2.362122200000008

GND,0.7056018925000014,1.2398943612876017

H2Otex,-9.914783537500057,-19.246097611154028

H2Otpp,-9.914783537500059,-21.246097611154028

HEX1,0.0,1.2993498555793714

Htex,-1.8376463999999728,-4.837646746964197

ICDHyr,1.2398589150000043,1.172210695639592

MCTP1Bpp,0.0,0.5

MDH,1.233197915000013,1.1665488956396075

MLDCP3App,0.0,0.5

MLDEP1pp,0.0,0.5

MLTGY3pp,0.0,0.5

MPTG,0.002778800000000001,0.5027788

NADH16pp,7.319156560000027,12.049518670415253

NDPK2,0.08505659999999997,2.085055626517942

NH4tex,2.160164800000022,4.160161026517926

NH4tpp,2.160164800000022,4.160161026517926

O2tex,4.206049537500015,6.537406483027439

O2tpp,4.206049537500015,6.537406483027439

PAPPT3,0.005557600000000001,1.0055576

PDH,1.8733075150000043,3.805667203942701

PFK_3,0.19573216416666564,1.896367226802117

PGAMT,-0.018897600000000004,-2.0188975999999457

PGI,1.434816097083335,4.45473485511036

PGK,-3.564233014999997,-6.496569887336477

PGL,0.7056018925000015,1.2398943612876017

PGM,-3.2205985149999905,-6.1529371873365335

PPK,-0.7007176000000049,-2.7026961833938685

RPE,0.31519592833333127,0.6713909075250513

RPI,-0.38244516416667035,-0.5605426537625817

SUCDi,1.024954315000004,0.9584398956396092

SUCOAS,-0.9198485150000041,-0.8522006956395671

TALA,0.0,-1.5225375730395854

TKT1,0.1957321641666656,0.3738296537625274

TKT2,0.11946376416666554,0.29756125376252385

TPI,1.7028532254166657,3.079966962635467

UAGCVT,0.005557600000000004,1.0055576

UAGDP,0.018897600000000004,2.0188976

UAGPT3,0.005557600000000001,1.0055576

UAPGR,0.005557600000000003,1.0055576

UDCPDP,0.005557600000000001,1.0055566265179008

UGMDDS,0.005557600000000003,1.0055576

UM3PL,0.0,1.0

UMPK,0.07560779999999996,1.0756077999999434

68 reacctions require a flux change for the evolution of the anhgm producer

.......................................................

.......................................................

arg__L

.......................................................

ACGK,0.05915840000000002,1.0591584

ACGS,0.05915840000000002,1.0591584

ACODA,0.059158400000000014,1.0591584

ACONTa,1.2398589150000043,1.9421916168789721

ACONTb,1.2398589150000041,1.9421916168789721

ACOTA,-0.05915840000000002,-1.0591584000000012

ACS,0.0,0.9989992000000001

ADK1,0.524992600000009,2.5251511400016398

AGPR,-0.05915840000000003,-1.0591584000000012

AKGDH,1.024820515000004,0.7261532168790157

ARGSL,0.05915840000000001,1.0591584000000012

ARGSS,0.05915840000000001,1.0591584

ARGt3pp,0.0,1.0

ARGtex,0.0,-1.0

ASPTA,-0.5855358000000094,-1.58553459999996

ATPS4rpp,13.370320970000053,16.96567190624353

CBMKr,0.12531739999999997,1.1253173999999717

CO2tex,-4.632830737500008,-5.389157992197511

CO2tpp,-4.632830737500008,-5.389157992197511

CS,1.2398589150000043,1.942191616879016

CYTBO3_4pp,8.410403675000031,10.923060584395028

DHAPT,0.32252132541666795,0.0

ENO,3.220598515000006,4.922930016879036

F6PA,0.32252132541666795,0.0

FBA3,0.19573216416666564,1.0955298490127916

FUM,1.2333893150000121,1.9357212168789601

G6PDH2r,0.7056018925000015,2.3549316415604835

GAPD,3.5642330149999975,5.26656271687898

GLCptspp,2.1404179895833364,3.166271216879016

GLCt2pp,0.0,0.10019891515389112

GLCtex_copy1,2.1404179895833364,3.265470132032874

GLUDy,-1.7103415000000157,-4.710338499999921

GND,0.7056018925000014,2.354931641560485

H2Otex,-9.914783537500057,-15.671103192197393

H2Otpp,-9.914783537500059,-15.671103192197393

HEX1,0.0,0.09919891515389123

Htex,-1.8376463999999728,-4.837644799999907

ICDHyr,1.2398589150000043,1.9411916168789958

MDH,1.233197915000013,1.9365298168789877

NADH16pp,7.319156560000027,10.129480767516013

NH4tex,2.160164800000022,6.1601619999999

NH4tpp,2.160164800000022,6.1601619999999

O2tex,4.206049537500015,5.462377992197503

O2tpp,4.206049537500015,5.462377992197503

OCBT,0.05915840000000002,1.0591584000000012

PDH,1.8733075150000043,2.576639016879016

PFK_3,0.19573216416666564,1.0955298490127527

PGI,1.434816097083335,0.9105384904723905

PGK,-3.564233014999997,-5.26656271687898

PGL,0.7056018925000015,2.354931641560485

PGM,-3.2205985149999905,-4.922930016879036

PPC,0.5918716000000012,1.5908711999999996

PPK,-0.7007176000000049,-2.7008761400016965

RPE,0.31519592833333127,1.4147498943736991

RPI,-0.38244516416667035,-0.9322209471868064

SUCDi,1.024954315000004,0.7282870168790156

SUCOAS,-0.9198485150000041,-0.621181616878971

TALA,0.0,-0.3500207018258834

TKT1,0.1957321641666656,0.7455091471867945

TKT2,0.11946376416666554,0.6692407471867909

TPI,1.7028532254166657,2.2791295848460322

61 reacctions require a flux change for the evolution of the arg__L producer

.......................................................

.......................................................

asn__L

.......................................................

ACONTa,1.2398589150000043,1.1965986612930237

ACONTb,1.2398589150000041,1.1965986612930237

ADK1,0.524992600000009,1.5241231999999627

AKGDH,1.024820515000004,0.9815602612930632

ASNS2,0.04821100000000001,1.048211

ASNt2rpp,0.0,-1.0

ASNtex,0.0,-1.0

ASPTA,-0.5855358000000094,-1.58553459999996

ATPS4rpp,13.370320970000053,14.456327072409977

CO2tex,-4.632830737500008,-4.02577257989401

CO2tpp,-4.632830737500008,-4.025281979893975

CS,1.2398589150000043,1.1965986612930632

CYTBO3_4pp,8.410403675000031,9.196324747466154

DHAPT,0.32252132541666795,0.0

ENO,3.220598515000006,4.177325669955962

F6PA,0.32252132541666795,0.0

FBA3,0.19573216416666564,0.9107343594179156

FUM,1.2333893150000121,1.1901282612931254

G6PDH2r,0.7056018925000015,1.2276005873518443

GAPD,3.5642330149999975,4.52120366995598

GLCptspp,2.1404179895833364,2.419666869956002

GLCt2pp,0.0,0.2862283134117276

GLCtex_copy2,0.0,0.5664771937843933

GLUDy,-1.7103415000000157,-2.7105829863330655

GND,0.7056018925000014,1.2276005873518763

H2Otex,-9.914783537500057,-12.307699879221559

H2Otpp,-9.914783537500059,-12.307699879221559

HEX1,0.0,0.2862283134117276

Htex,-1.8376463999999728,-3.837643172666162

ICDHyr,1.2398589150000043,1.1965986612930237

MDH,1.233197915000013,1.1899814612930868

NADH16pp,7.319156560000027,8.147847286173091

NH4tex,2.160164800000022,4.160159558999226

NH4tpp,2.160164800000022,4.160159558999226

O2tex,4.206049537500015,4.599010073733098

O2tpp,4.206049537500015,4.599010073733098

PDH,1.8733075150000043,1.8300525706285264

PFK_3,0.19573216416666564,0.9107343594178987

PGI,1.434816097083335,1.4782954096828007

PGK,-3.564233014999997,-4.52120366995598

PGL,0.7056018925000015,1.2276005873518763

PGM,-3.2205985149999905,-4.177325669955962

PPC,0.5918716000000012,1.5918712000000002

PPK,-0.7007176000000049,-1.7028286719936432

RPE,0.31519592833333127,0.6631958582345305

RPI,-0.38244516416667035,-0.5564439291173358

SUCDi,1.024954315000004,0.9826940612930631

SUCOAS,-0.9198485150000041,-0.8765886612931126

TALA,0.0,-0.5410022303005917

TKT1,0.1957321641666656,0.3697321291173239

TKT2,0.11946376416666554,0.2934637291173203

TPI,1.7028532254166657,2.09433409525127

52 reacctions require a flux change for the evolution of the asn__L producer

.......................................................

.......................................................

asp__L

.......................................................

ACONTa,1.2398589150000043,1.0982570946008536

ACONTb,1.2398589150000041,1.0982570946008536

AKGDH,1.024820515000004,0.8822186946008057

ASPTA,-0.5855358000000094,-1.58553459999996

ASPt2pp_copy2,0.0,-1.0009999999999764

ASPtex,0.0,-1.0

ATPS4rpp,13.370320970000053,13.653552925077747

CO2tex,-4.632830737500008,-3.7793017293623734

CO2tpp,-4.632830737500008,-3.7793017293623734

CS,1.2398589150000043,1.098257094600806

CYTBO3_4pp,8.410403675000031,8.703348058724682

DHAPT,0.32252132541666795,0.0

ENO,3.220598515000006,4.078995494600804

F6PA,0.32252132541666795,0.0

FBA,1.2128609358333324,1.8683668088737022

FUM,1.2333893150000121,1.0917866946008417

G6PDH2r,0.7056018925000015,1.276878945559929

GAPD,3.5642330149999975,4.4226281946007475

GLCptspp,2.1404179895833364,2.3223366946008066

GLCt2pp,0.0,0.3424907269595825

GLCtex_copy2,0.0,0.5254094319770526

GLUDy,-1.7103415000000157,-2.7103385000000344

GND,0.7056018925000014,1.276878945559929

H2Otex,-9.914783537500057,-11.061246929362369

H2Otpp,-9.914783537500059,-11.061246929362369

HEX1,0.0,0.3424907269595825

Htex,-1.8376463999999728,-3.837644800000021

ICDHyr,1.2398589150000043,1.0972570946007636

MDH,1.233197915000013,1.0926398946007794

NADH16pp,7.319156560000027,7.753702764123877

NH4tex,2.160164800000022,3.160162000000014

NH4tpp,2.160164800000022,3.160162000000014

O2tex,4.206049537500015,4.3525217293623655

O2tpp,4.206049537500015,4.3525217293623655

PDH,1.8733075150000043,1.7327044946008061

PFK,1.2128609358333324,1.8683668088737484

PGI,1.434816097083335,1.3879484760004743

PGK,-3.564233014999997,-4.4226281946007475

PGL,0.7056018925000015,1.276878945559929

PGM,-3.2205985149999905,-4.078995494600804

PPC,0.5918716000000012,1.5908712000000003

RPE,0.31519592833333127,0.6960480970399203

RPI,-0.38244516416667035,-0.5728700485200307

SUCDi,1.024954315000004,0.8844862946008059

SUCOAS,-0.9198485150000041,-0.7772470946008525

TALA,0.0,0.18942608435327202

TKT1,0.1957321641666656,0.3861582485200188

TKT2,0.11946376416666554,0.3098898485200152

TPI,1.7028532254166657,2.036837773040361

49 reacctions require a flux change for the evolution of the asp__L producer

.......................................................

.......................................................

cgly

.......................................................

ACONTa,1.2398589150000043,1.2010789675113092

ACONTb,1.2398589150000041,1.2010789675113092

ACS,0.0,0.9989999999985815

ADK1,0.524992600000009,2.5251271999967235

ADSK,0.04957439999999991,1.0495744

AKGDH,1.024820515000004,0.9860405675113088

ATPS4rpp,13.370320970000053,20.447390264974416

BPNT,0.049574399999999914,1.0495744

CGLYtex,0.0,-1.0

CO2tex,-4.632830737500008,-8.0085786189204

CO2tpp,-4.632830737500008,-7.030420418797999

CS,1.2398589150000043,1.2010789675113092

CYSS,0.04957439999999994,1.0495744

CYTBO3_4pp,8.410403675000031,13.161899437855158

DHAPT,0.32252132541666795,0.0

ENO,3.220598515000006,3.1818185675008444

F6PA,0.32252132541666795,0.0

FBA,1.2128609358333324,2.0907234335791554

FDH4pp,0.0,0.9771582001223995

FORtppi,0.0,0.9781582001223995

FTHFD,0.0,0.9776676001223992

FUM,1.2333893150000121,1.1946093675107408

G6PDH2r,0.7056018925000015,3.23045231621191

GAPD,3.5642330149999975,5.514532167561869

GHMT2r,0.22309610000000604,1.2121752000611998

GLCptspp,2.1404179895833364,2.42415936750239

GLCt2pp,0.0,1.1122166023127926

GLCtex_copy2,0.0,1.3949579802318461

GLUCYS,0.0,1.0

GLUDy,-1.7103415000000157,-3.699420600059881

GLUt2rpp,0.0,1.0

GLYCL,0.010920900000001992,6.119948992022728e-11

GND,0.7056018925000014,3.23045231621191

GTHRDHpp,0.0,1.0

GTHRDabc2pp,0.0,1.0

GTHS,0.0,1.0

H2Otex,-9.914783537500057,-17.290531418914163

H2Otpp,-9.914783537500059,-18.290531418913922

HEX1,0.0,1.1112166023127927

ICDHyr,1.2398589150000043,1.2010789675113092

MDH,1.233197915000013,1.1944625675107405

MTHFC,0.1977522000000081,1.1759104001223992

MTHFD,0.1977522000000081,1.1759104001223992

NADH16pp,7.319156560000027,11.130407870221495

NDPK1,0.158125000000004,1.1571250000000237

NH4tex,2.160164800000022,4.160164799997862

NH4tpp,2.160164800000022,4.160164799997862

O2tex,4.206049537500015,6.581797418927579

O2tpp,4.206049537500015,6.581797418927579

PAPSR,0.049574399999999914,1.0495744

PDH,1.8733075150000043,1.8345275675020916

PFK,1.2128609358333324,2.0907234335791554

PGCD,0.34363450000000595,2.332713600061025

PGI,1.434816097083335,0.30492365360327245

PGK,-3.564233014999997,-5.514532167561869

PGL,0.7056018925000015,3.23045231621191

PGM,-3.2205985149999905,-3.1818185675008444

PPK,-0.7007176000000049,-2.699852199994489

PSERT,0.343634500000006,2.332713600061025

PSP_L,0.343634500000006,2.332713600061025

RPE,0.31519592833333127,1.9984295441416504

RPI,-0.38244516416667035,-1.2240619720707184

SADT2,0.04957439999999991,1.0495744

SERAT,0.04957439999999993,1.0495744

SO4t2pp,0.05044199999999991,1.050442

SO4tex,0.05044199999999991,1.050442

SUCDi,1.024954315000004,0.9891743675112639

SUCOAS,-0.9198485150000041,-0.8810685675112842

SULR,0.049574399999999914,1.0495744

TALA,0.0,0.8426168079044933

TKT1,0.1957321641666656,1.037348972070719

TKT2,0.11946376416666554,0.9610805720709314

TPI,1.7028532254166657,2.2571943977455566

TRDR,0.049574399999999914,1.0495744

74 reacctions require a flux change for the evolution of the cgly producer

.......................................................

.......................................................

cit

.......................................................

ACONTa,1.2398589150000043,0.714858914246588

ACONTb,1.2398589150000041,0.714858914246588

AKGDH,1.024820515000004,0.4998205410881609

ATPS4rpp,13.370320970000053,13.420319587567562

CITt3pp,0.0,1.0

CITtex,0.0,-1.0

CO2tex,-4.632830737500008,-3.320330772928422

CO2tpp,-4.632830737500008,-3.320330772928422

CS,1.2398589150000043,1.7148589145287487

CYTBO3_4pp,8.410403675000031,8.785402917082413

DHAPT,0.32252132541666795,0.0162712833790769

ENO,3.220598515000006,4.695598443819671

F6PA,0.32252132541666795,0.0162712833790769

FBA,1.2128609358333324,2.2118609459247605

FUM,1.2333893150000121,0.7083893154002681

G6PDH2r,0.7056018925000015,0.9681018914349209

GAPD,3.5642330149999975,5.039232944050907

GLCptspp,2.1404179895833364,2.9216679895837387

GLCtex_copy2,0.0,0.7802500000001324

GND,0.7056018925000014,0.9681018914349209

H2Otex,-9.914783537500057,-10.602283537075525

H2Otpp,-9.914783537500059,-10.602283537034737

Htex,-1.8376463999999728,-4.837646372832587

ICDHyr,1.2398589150000043,0.7148589137628841

MDH,1.233197915000013,0.7081979152777367

NADH16pp,7.319156560000027,8.219155776236313

O2tex,4.206049537500015,4.393549524868232

O2tpp,4.206049537500015,4.393549524868232

PDH,1.8733075150000043,2.348307514425642

PFK,1.2128609358333324,2.2118609459247605

PGI,1.434816097083335,1.953566098027892

PGK,-3.564233014999997,-5.039232944050907

PGL,0.7056018925000015,0.9681018914349209

PGM,-3.2205985149999905,-4.695598443819671

PPC,0.5918716000000012,1.5918715708579776

RPE,0.31519592833333127,0.49019589544167286

RPI,-0.38244516416667035,-0.4699451959937064

SUCDi,1.024954315000004,0.5010881408461008

SUCOAS,-0.9198485150000041,-0.3948485445652252

TALA,0.0,0.08649998355406432

TKT1,0.1957321641666656,0.28323214772072997

TKT2,0.11946376416666554,0.20696374772094286

TPI,1.7028532254166657,2.3966031935930343

43 reacctions require a flux change for the evolution of the cit producer

.......................................................

.......................................................

cys__L

.......................................................

ACONTa,1.2398589150000043,1.303780360798499

ACONTb,1.2398589150000041,1.303780360798499

ACS,0.0,0.9989999999981726

ADK1,0.524992600000009,3.5251271999968266

ADK3,0.0,-1.0010000000000483

ADSK,0.04957439999999991,1.0495744

AKGDH,1.024820515000004,1.0887419607990663

ATPS4rpp,13.370320970000053,17.242477278398837

BPNT,0.049574399999999914,1.0495744

CO2tex,-4.632830737500008,-7.292634351984131

CO2tpp,-4.632830737500008,-7.292634351984131

CS,1.2398589150000043,1.303780360798499

CYSS,0.04957439999999994,1.0495744

CYStex,0.0,-1.0

CYStpp,0.0,1.0

CYTBO3_4pp,8.410403675000031,10.73001090398516

DHAPT,0.32252132541666795,3.2007041494974686e-06

ENO,3.220598515000006,3.284519960786243

F6PA,0.32252132541666795,3.2007041494974686e-06

FBA3,0.19573216416666564,0.6378711322556789

FUM,1.2333893150000121,1.2973107607990648

G6PDH2r,0.7056018925000015,3.1736411695977322

GAPD,3.5642330149999975,4.628154460786051

GLCptspp,2.1404179895833364,2.526857560084796

GLCt2pp,0.0,0.5568610319067737

GLCtex_copy2,0.0,0.9423006024082334

GLUDy,-1.7103415000000157,-2.710341499998065

GND,0.7056018925000014,3.1736411695977322

H2Otex,-9.914783537500057,-14.575087151977982

H2Otpp,-9.914783537500059,-14.574587151977502

HEX1,0.0,0.5558610319067738

Htex,-1.8376463999999728,-0.8366463999979354

ICDHyr,1.2398589150000043,1.303780360798499

MDH,1.233197915000013,1.2971639607990653

NADH16pp,7.319156560000027,9.574842343186093

NH4tex,2.160164800000022,3.1601647999976064

NH4tpp,2.160164800000022,3.1601647999976064

O2tex,4.206049537500015,5.36610315199258

O2tpp,4.206049537500015,5.36585315199258

PAPSR,0.049574399999999914,1.0495744

PDH,1.8733075150000043,1.937228960788847

PFK_3,0.19573216416666564,0.6378711322556789

PGCD,0.34363450000000595,1.3436344999998087

PGI,1.434816097083335,-0.0909225776061624

PGK,-3.564233014999997,-4.628154460786051

PGL,0.7056018925000015,3.1736411695977322

PGM,-3.2205985149999905,-3.284519960786243

PPK,-0.7007176000000049,-2.699852199994602

PSERT,0.343634500000006,1.3436344999998087

PSP_L,0.343634500000006,1.3436344999998087

RPE,0.31519592833333127,1.960555446398865

RPI,-0.38244516416667035,-1.2051249231993257

SADT2,0.04957439999999991,1.0495744

SERAT,0.04957439999999993,1.0495744

SO4t2pp,0.05044199999999991,1.050442

SO4tex,0.05044199999999991,1.050442

SUCDi,1.024954315000004,1.0888757607990662

SUCOAS,-0.9198485150000041,-0.9837699607990662

SULR,0.049574399999999914,1.0495744

TALA,0.0,0.3805407909436471

TKT1,0.1957321641666656,1.018411923199326

TKT2,0.11946376416666554,0.942143523199539

TPI,1.7028532254166657,1.8234740687933506

TRDR,0.049574399999999914,1.0495744

64 reacctions require a flux change for the evolution of the cys__L producer

.......................................................

.......................................................

cytd

.......................................................

ACONTa,1.2398589150000043,1.5378666031035195

ACONTb,1.2398589150000041,1.5378666031035195

ADK1,0.524992600000009,1.5229925999972163

AKGDH,1.024820515000004,1.322828203103501

ASPCT,0.06615899999999995,1.066159

ASPTA,-0.5855358000000094,-1.5855357999984645

ATPS4rpp,13.370320970000053,17.774305593790768

CBMKr,0.12531739999999997,1.1253173999994328

CO2tex,-4.632830737500008,-4.877849957743167

CO2tpp,-4.632830737500008,-4.877849957743167

CS,1.2398589150000043,1.5378666031035195

CTPS2,0.03210499999999995,1.032105

CYTBO3_4pp,8.410403675000031,11.900442115506632

CYTDt2pp_copy2,0.0,-1.0

CYTDtex,0.0,-1.0

CYTK1,0.036180999999999956,-0.9638190000008238

DHAPT,0.32252132541666795,8.840321807923601e-10

DHORDfum,0.0,0.9980000000000001

DHORTS,-0.06615899999999995,-1.066159

ENO,3.220598515000006,4.518606203088841

F6PA,0.32252132541666795,8.840321807923601e-10

FBA3,0.19573216416666564,1.2744246400856256

FUM,1.2333893150000121,1.5313970031027377

G6PDH2r,0.7056018925000015,1.0565980484450113

GAPD,3.5642330149999975,4.862240703088373

GLCptspp,2.1404179895833364,2.760947002206926

GLCt2pp,0.0,0.9203075240767737

GLCtex_copy1,2.1404179895833364,3.6812545262836998

GLNS,0.36212300000000835,1.3621229999993456

GLUDy,-1.7103415000000157,-2.710341499997225

GND,0.7056018925000014,1.0565980484450113

H2Otex,-9.914783537500057,-17.159802757734443

H2Otpp,-9.914783537500059,-17.159802757733964

HEX1,0.0,0.9193075240767739

Htex,-1.8376463999999728,-4.837646399997375

ICDHyr,1.2398589150000043,1.5378666031035195

MDH,1.233197915000013,1.5312502031027377

NADH16pp,7.319156560000027,9.51118731240313

NDPK2,0.08505659999999997,1.0850565999985815

NDPK3,0.03618099999999997,-0.9638190000008238

NH4tex,2.160164800000022,5.160164799996305

NH4tpp,2.160164800000022,5.160164799996305

NTD4,0.0,1.0

O2tex,4.206049537500015,5.9510687577533155

O2tpp,4.206049537500015,5.9510687577533155

OMPDC,0.06615899999999997,1.066159

ORPT,-0.06615899999999995,-1.066159

PDH,1.8733075150000043,2.171315203091066

PFK_3,0.19573216416666564,1.2744246400856256

PGI,1.434816097083335,2.6236564778386886

PGK,-3.564233014999997,-4.862240703088373

PGL,0.7056018925000015,1.0565980484450113

PGM,-3.2205985149999905,-4.518606203088841

PPC,0.5918716000000012,1.5918715999992465

PPK,-0.7007176000000049,-1.7007175999951745

PRPPS,0.1865784000000047,1.1855783999998117

RPE,0.31519592833333127,-0.11747330103615652

RPI,-0.38244516416667035,-1.166110549481627

SUCDi,1.024954315000004,2.321962003103501

SUCOAS,-0.9198485150000041,-1.217856203103501

TALA,0.0,-1.2950270906038104

TKT1,0.1957321641666656,-0.020602450518184767

TKT2,0.11946376416666554,-0.09687085051797176

TPI,1.7028532254166657,2.4600243768033554

UMPK,0.07560779999999996,1.0756077999992908

65 reacctions require a flux change for the evolution of the cytd producer

.......................................................

.......................................................

dha

.......................................................

CYTBO3_4pp,8.410403675000031,8.432269630891714

DHAPT,0.32252132541666795,0.0

DHAtex,0.0,-1.0

DHAtpp,0.0,-1.0

F6PA,0.32252132541666795,1.0

FBA,1.2128609358333324,1.0389332886597995

G6PDH2r,0.7056018925000015,0.7034152992242546

GLCptspp,2.1404179895833364,2.4673125092614563

GLCt2pp,0.0,0.17492764330547095

GLCtex_copy1,2.1404179895833364,2.6422401525669272

GND,0.7056018925000014,0.7034152992242546

H2Otex,-9.914783537500057,-9.925716515429059

H2Otpp,-9.914783537500059,-9.92571651542882

HEX1,0.0,0.1739276433053619

NADH16pp,7.319156560000027,7.3346493254838165

PFK,1.2128609358333324,1.0389332886597995

PGI,1.434816097083335,1.9378248533425635

PGL,0.7056018925000015,0.7034152992242546

TPI,1.7028532254166657,1.2054042528266407

19 reacctions require a flux change for the evolution of the dha producer

.......................................................

.......................................................

etha

.......................................................

AACPS1,0.0,1.9940000000000713

ACONTa,1.2398589150000043,1.4926920651063083

ACONTb,1.2398589150000041,1.4926920651063083

ADK1,0.524992600000009,4.525992599998494

AGPAT140,0.0,0.9970000000000356

AKGDH,1.024820515000004,1.2776536651068575

ATPS4rpp,13.370320970000053,18.86465466978109

CO2tex,-4.632830737500008,-6.764913612757526

CO2tpp,-4.632830737500008,-6.764913612757526

CS,1.2398589150000043,1.4926920651063083

CYTBO3_4pp,8.410403675000031,11.674569425525785

CYTK1,0.036180999999999956,1.0361809999995422

DASYN140,0.0,0.9970000000000356

DHAPT,0.32252132541666795,5.499620758797619e-10

ENO,3.220598515000006,3.4734316650980954

ETHAtex,0.0,-1.0

F6PA,0.32252132541666795,5.499620758797619e-10

FACOAE140,0.0,1.9940000000000713

FACOAL140t2pp,0.0,1.9940000000000713

FBA3,0.19573216416666564,1.0814061599247708

FUM,1.2333893150000121,1.4862224651066465

G3PAT140,0.0,0.9970000000000356

G6PDH2r,0.7056018925000015,1.0791853174447825

GAPD,3.5642330149999975,4.817066165097984

GLCptspp,2.1404179895833364,2.7157724645502284

GLCt2pp,0.0,0.11332600423841521

GLCtex_copy1,2.1404179895833364,2.8290984687886436

GLUDy,-1.7103415000000157,-2.7103414999983726

GLYC3Pt6pp,1.6967721175485333e-34,1.0

GND,0.7056018925000014,1.0791853174447825

GPDDA2pp,5.655907058495111e-35,1.0

H2Otex,-9.914783537500057,-12.046866412751513

H2Otpp,-9.914783537500059,-15.046866412751273

HEX1,0.0,0.11332600423841525

ICDHyr,1.2398589150000043,1.4926920651063083

LPLIPAL1E140pp,0.0,0.9970000000000356

MDH,1.233197915000013,1.486031065106646

NADH16pp,7.319156560000027,10.330489160418928

NDPK3,0.03618099999999997,1.0361809999995422

NH4tex,2.160164800000022,3.1601647999980065

NH4tpp,2.160164800000022,3.1601647999980065

O2tex,4.206049537500015,5.838132412762892

O2tpp,4.206049537500015,5.838132412762892

PDH,1.8733075150000043,2.126140665099891

PE140abcpp,0.0,0.9970000000000356

PFK_3,0.19573216416666564,1.0814061599247708

PGCD,0.34363450000000595,1.3436344999998884

PGI,1.434816097083335,1.749913151343861

PGK,-3.564233014999997,-4.817066165097984

PGL,0.7056018925000015,1.0791853174447825

PGM,-3.2205985149999905,-3.4734316650980954

PIt2rpp,0.19292620000000565,1.1929261999995053

PLIPA2E140pp,0.0,0.9970000000000356

PPK,-0.7007176000000049,-5.70071759999683

PSD140,0.0,0.9970000000000356

PSERT,0.343634500000006,1.3436344999998884

PSP_L,0.343634500000006,1.3436344999998884

PSSA140,0.0,0.9970000000000356

RPE,0.31519592833333127,0.5642515449636953

RPI,-0.38244516416667035,-0.5069729724815455

SUCDi,1.024954315000004,1.2777874651068575

SUCOAS,-0.9198485150000041,-1.172681665106833

TALA,0.0,-0.7611461874430299

TKT1,0.1957321641666656,0.32025997248174093

TKT2,0.11946376416666554,0.24399157248195436

TPI,1.7028532254166657,2.2670058963080155

66 reacctions require a flux change for the evolution of the etha producer

.......................................................

.......................................................

etoh

.......................................................

ACALD,0.0,-1.0000000002789875

ACONTa,1.2398589150000043,1.1398594234365582

ACONTb,1.2398589150000041,1.1398594234365582

AKGDH,1.024820515000004,0.9248210572267231

ALCD2x,0.0,-1.0

ATPS4rpp,13.370320970000053,12.570389510243672

CO2tex,-4.632830737500008,-5.3828308665593125

CO2tpp,-4.632830737500008,-5.382830866081863

CS,1.2398589150000043,1.1398594234365582

CYTBO3_4pp,8.410403675000031,7.910442672728591

DHAPT,0.32252132541666795,0.7641890825869383

ENO,3.220598515000006,4.12059961544603

ETOHtex,0.0,-1.0

ETOHtrpp,0.0,-1.0

F6PA,0.32252132541666795,0.7641890825869383

FUM,1.2333893150000121,1.133389863921871

G6PDH2r,0.7056018925000015,0.7556004374482024

GAPD,3.5642330149999975,4.464233545473478

GLCptspp,2.1404179895833364,2.598751322916769

GLCtex_copy2,0.0,0.4573333333331626

GND,0.7056018925000014,0.7556004374482024

H2Otex,-9.914783537500057,-9.66528258600479

H2Otpp,-9.914783537500059,-9.664783702414033

ICDHyr,1.2398589150000043,1.1398594234365582

MDH,1.233197915000013,1.1331984732009541

NADH16pp,7.319156560000027,6.919195016815073

O2tex,4.206049537500015,3.956300337513587

O2tpp,4.206049537500015,3.9560508954198017

PDH,1.8733075150000043,2.7733080106169314

PGI,1.434816097083335,1.8431508853492042

PGK,-3.564233014999997,-4.464233545473478

PGL,0.7056018925000015,0.7556004374482024

PGM,-3.2205985149999905,-4.12059961544603

RPE,0.31519592833333127,0.34852829412943626

RPI,-0.38244516416667035,-0.3991113433192244

SUCDi,1.024954315000004,0.9249548572267231

SUCOAS,-0.9198485150000041,-0.8198490733330323

TALA,0.0,0.017666182897946026

TKT1,0.1957321641666656,0.21239834706461166

TKT2,0.11946376416666554,0.1361299470648246

TPI,1.7028532254166657,2.144520391129212

41 reacctions require a flux change for the evolution of the etoh producer

.......................................................

.......................................................

for

.......................................................

ACONTa,1.2398589150000043,1.439541777506065

ACONTb,1.2398589150000041,1.439541777506065

AKGDH,1.024820515000004,1.2245033775061198

ATPS4rpp,13.370320970000053,12.970453044987721

CO2tex,-4.632830737500008,-4.132799143747775

CO2tpp,-4.632830737500008,-4.132799143747775

CS,1.2398589150000043,1.43954177750612

DHAPT,0.32252132541666795,0.4378777202148254

ENO,3.220598515000006,3.420280177506129

F6PA,0.32252132541666795,0.4378777202148285

FORtex,0.0,-1.0

FORtppi,0.0,1.0

FUM,1.2333893150000121,1.4330713775061668

G6PDH2r,0.7056018925000015,0.6057864112294737

GAPD,3.5642330149999975,3.764158177506147

GLCptspp,2.1404179895833364,2.223743657291294

GLCtex_copy2,0.0,0.08432566770795757

GND,0.7056018925000014,0.6057864112294472

H2Otex,-9.914783537500057,-9.41474434374777

H2Otpp,-9.914783537500059,-9.41474434374777

Htex,-1.8376463999999728,-2.837644800000021

ICDHyr,1.2398589150000043,1.439541777506065

MDH,1.233197915000013,1.4328799775061043

NADH16pp,7.319156560000027,7.1185467099894755

PDH,1.8733075150000043,1.0734797775061198

PFL,0.0,0.9995094

PGI,1.434816097083335,1.617957246061792

PGK,-3.564233014999997,-3.764158177506147

PGL,0.7056018925000015,0.6057864112294472

PGM,-3.2205985149999905,-3.420280177506129

RPE,0.31519592833333127,0.24865307415291227

RPI,-0.38244516416667035,-0.3491725370764698

SUCDi,1.024954315000004,1.2256371775061197

SUCOAS,-0.9198485150000041,-1.119531777506154

TALA,0.0,-0.033513327090190614

TKT1,0.1957321641666656,0.16246073707645792

TKT2,0.11946376416666554,0.08619233707645435

TPI,1.7028532254166657,1.819451520214784

38 reacctions require a flux change for the evolution of the for producer

.......................................................

.......................................................

g3pe

.......................................................

AACPS1,0.0,2.0

ACONTa,1.2398589150000043,1.6056284100751752

ACONTb,1.2398589150000041,1.6056284100751752

ADK1,0.524992600000009,4.525992599999073

AGPAT140,0.0,1.0

AKGDH,1.024820515000004,1.3905900100757425

ATPS4rpp,13.370320970000053,19.638781979844374

CO2tex,-4.632830737500008,-7.547254475179647

CO2tpp,-4.632830737500008,-7.547254475179647

CS,1.2398589150000043,1.6056284100751752

CYTBO3_4pp,8.410403675000031,12.2392511503716

CYTK1,0.036180999999999956,1.036180999999542

DASYN140,0.0,1.0

DHAPT,0.32252132541666795,5.415592418955839e-10

ENO,3.220598515000006,3.586368010066944

F6PA,0.32252132541666795,5.415592418955839e-10

FACOAE140,0.0,2.0

FACOAL140t2pp,0.0,2.0

FBA3,0.19573216416666564,1.5639523611651185

FUM,1.2333893150000121,1.5991588100757377

G3PAT140,0.0,1.0

G3PD2,-0.027805600000000014,-1.0278056

G3PEtex,0.0,-1.0

G6PDH2r,0.7056018925000015,1.5227171449601826

GAPD,3.5642330149999975,4.930002510066941

GLCptspp,2.1404179895833364,2.828708809527498

GLCt2pp,0.0,0.6307798029983522

GLCtex_copy1,2.1404179895833364,3.4594886125258504

GLUDy,-1.7103415000000157,-2.7103414999982816

GND,0.7056018925000014,1.5227171449601826

H2Otex,-9.914783537500057,-13.82920727517485

H2Otpp,-9.914783537500059,-15.82920727517437

HEX1,0.0,0.6297798029983763

Htex,-1.8376463999999728,-0.8376463999990721

ICDHyr,1.2398589150000043,1.6056284100751752

LPLIPAL1E140pp,0.0,1.0

MDH,1.233197915000013,1.5990120100757381

NADH16pp,7.319156560000027,10.78223454029586

NDPK3,0.03618099999999997,1.036180999999542

NH4tex,2.160164800000022,3.1601647999980256

NH4tpp,2.160164800000022,3.1601647999980256

O2tex,4.206049537500015,6.120473275185801

O2tpp,4.206049537500015,6.120473275185801

PDH,1.8733075150000043,2.2390770100687587

PE140abcpp,0.0,1.0

PFK_3,0.19573216416666564,1.5639523611651185

PGCD,0.34363450000000595,1.3436344999999965

PGI,1.434816097083335,1.9357714675656916

PGK,-3.564233014999997,-4.930002510066941

PGL,0.7056018925000015,1.5227171449601826

PGM,-3.2205985149999905,-3.586368010066944

PIt2rpp,0.19292620000000565,1.1929261999995417

PItex,0.19292620000000563,1.1929261999995417

PLIPA2E140pp,0.0,1.0

PPK,-0.7007176000000049,-5.700717599997206

PSD140,0.0,1.0

PSERT,0.343634500000006,1.3436344999999965

PSP_L,0.343634500000006,1.3436344999999965

PSSA140,0.0,1.0

RPE,0.31519592833333127,0.859939429973833

RPI,-0.38244516416667035,-0.6548169149868078

SUCDi,1.024954315000004,1.3907238100757424

SUCOAS,-0.9198485150000041,-1.285618010075718

TALA,0.0,-1.0958484461783085

TKT1,0.1957321641666656,0.46810391498681003

TKT2,0.11946376416666554,0.39183551498702296

TPI,1.7028532254166657,1.7495520975399588

67 reacctions require a flux change for the evolution of the g3pe producer

.......................................................

.......................................................

g3pg

.......................................................

AACPS2,0.0,1.999

ACONTa,1.2398589150000043,2.0491677975772973

ACONTb,1.2398589150000041,2.0491677975772973

ADK1,0.524992600000009,4.525122799999963

AGPAT141,0.0,0.9995

AKGDH,1.024820515000004,1.8341293975773254

ATPS4rpp,13.370320970000053,19.75176954484857

CO2tex,-4.632830737500008,-7.656098443943279

CO2tpp,-4.632830737500008,-7.656098443943279

CS,1.2398589150000043,2.049245737580619

CYTBO3_4pp,8.410403675000031,12.456941487886613

CYTK1,0.036180999999999956,1.036181000000056

DASYN141,0.0,0.9995

DHAPT,0.32252132541666795,0.0

ENO,3.220598515000006,4.029906197577361

F6PA,0.32252132541666795,0.0

FACOAE141,0.0,1.999

FACOAL141t2pp,0.0,1.999

FBA,1.2128609358333324,2.839811392753404

FUM,1.2333893150000121,2.0426973975772853

G3PAT141,0.0,0.9995

G3PD2,-0.027805600000000014,-2.027805599999965

G3PGtex,0.0,-1.0

G6PDH2r,0.7056018925000015,1.3009435512113896

GAPD,3.5642330149999975,4.373538897577305

GLCptspp,2.1404179895833364,3.2722473975773263

GLCt2pp,0.0,0.3720461430798898

GLCtex_copy2,0.0,1.5048755510738796

GND,0.7056018925000014,1.300943551211336

H2Otex,-9.914783537500057,-12.938043643943274

H2Otpp,-9.914783537500059,-14.938043643943274

HEX1,0.0,0.3710461430798899

Htex,-1.8376463999999728,-0.837644800000021

ICDHyr,1.2398589150000043,2.0491677975772973

LPLIPAL1G141pp,0.0,0.9995

MDH,1.233197915000013,2.0425059975773365

NADH16pp,7.319156560000027,10.555385490309288

NDPK3,0.03618099999999997,1.036181000000056

O2tex,4.206049537500015,6.229318443943271

O2tpp,4.206049537500015,6.229318443943271

PDH,1.8733075150000043,2.6826151975773262

PFK,1.2128609358333324,2.839811392753441

PG141abcpp,0.0,0.9995

PGI,1.434816097083335,2.342349989445893

PGK,-3.564233014999997,-4.373538897577305

PGL,0.7056018925000015,1.300943551211336

PGM,-3.2205985149999905,-4.029906197577361

PGPP141,0.0,0.9995

PGSA141,0.0,0.9995

PIt2rpp,0.19292620000000565,1.192926199999988

PItex,0.19292620000000563,1.192926199999988

PLIPA2G141pp,0.0,0.9995

PPK,-0.7007176000000049,-5.701849000000038

RPE,0.31519592833333127,0.7120911674742274

RPI,-0.38244516416667035,-0.5808915837370705

SUCDi,1.024954315000004,1.8362631975773254

SUCOAS,-0.9198485150000041,-1.7291577975772725

TALA,0.0,0.19744761957042556

TKT1,0.1957321641666656,0.39417978373705864

TKT2,0.11946376416666554,0.31791138373716876

TPI,1.7028532254166657,1.0082823569200627

61 reacctions require a flux change for the evolution of the g3pg producer

.......................................................

.......................................................

glcn

.......................................................

ACONTa,1.2398589150000043,1.1398588589993324

ACONTb,1.2398589150000041,1.1398588589993324

AKGDH,1.024820515000004,0.924820458999313

ATPS4rpp,13.370320970000053,13.57032266199451

CO2tex,-4.632830737500008,-4.382830857498334

CO2tpp,-4.632830737500008,-4.382830857498334

CS,1.2398589150000043,1.139858858999313

CYTBO3_4pp,8.410403675000031,8.910404954996567

DHAPT,0.32252132541666795,0.2641884960829375

ENO,3.220598515000006,3.120599298999309

F6PA,0.32252132541666795,0.2641884960829657

FUM,1.2333893150000121,1.1333898189992624

G6PDH2r,0.7056018925000015,0.7556014205003976

GAPD,3.5642330149999975,3.4642340389992796

GLCDpp,0.0,1.0

GLCNtex,0.0,-1.0

GLCptspp,2.1404179895833364,2.098751322916385

GLCtex_copy2,0.0,0.9593333333330487

GND,0.7056018925000014,0.7556014205003599

H2Otex,-9.914783537500057,-9.664788977498233

H2Otpp,-9.914783537500059,-10.664788977498233

Htex,-1.8376463999999728,-2.837646839999934

ICDHyr,1.2398589150000043,1.1398588589993324

MDH,1.233197915000013,1.1331984189993136

NADH16pp,7.319156560000027,6.9191578959972535

O2tex,4.206049537500015,4.456050177498241

O2tpp,4.206049537500015,4.456050177498241

PDH,1.8733075150000043,1.7733062589993134

PGI,1.434816097083335,1.3431499024160303

PGK,-3.564233014999997,-3.4642340389992796

PGL,0.7056018925000015,0.7556014205003599

PGM,-3.2205985149999905,-3.120599298999309

RPE,0.31519592833333127,0.3485292936669566

RPI,-0.38244516416667035,-0.39911132683346295

SUCDi,1.024954315000004,0.925088058999313

SUCOAS,-0.9198485150000041,-0.8198488589993076

TALA,0.0,0.017666682666799716

TKT1,0.1957321641666656,0.2123988468334801

TKT2,0.11946376416666554,0.13613044683347653

TPI,1.7028532254166657,1.644520396082953

40 reacctions require a flux change for the evolution of the glcn producer

.......................................................

.......................................................

glu__L

.......................................................

ACONTa,1.2398589150000043,1.640536007615484

ACONTb,1.2398589150000041,1.640536007615484

AKGDH,1.024820515000004,0.4254976076154841

ATPS4rpp,13.370320970000053,13.568966784764303

CO2tex,-4.632830737500008,-4.134523469027052

CO2tpp,-4.632830737500008,-4.134523469027052

CS,1.2398589150000043,1.640536007615484

DHAPT,0.32252132541666795,6.896592186222961e-10

ENO,3.220598515000006,4.6212756076042085

F6PA,0.32252132541666795,6.896592186222961e-10

FBA3,0.19573216416666564,1.1676484595805066

FRD2,0.0,0.5963229073845201

FUM,1.2333893150000121,0.6340664076147196

G6PDH2r,0.7056018925000015,1.0052633461894156

GAPD,3.5642330149999975,4.964910107603915

GLCptspp,2.1404179895833364,2.863616406916662

GLCt2pp,0.0,0.027083704582296253

GLCtex_copy2,0.0,0.749282121915622

GLUDy,-1.7103415000000157,-2.710341499997618

GLUt2rpp,0.0,-1.0019999999999998

GLUtex,0.0,-1.0

GND,0.7056018925000014,1.0052633461894156

H2Otex,-9.914783537500057,-11.416476269019391

H2Otpp,-9.914783537500059,-11.416476269019137

HEX7,0.0,0.026083704582296363

Htex,-1.8376463999999728,-3.8376463999971597

ICDHyr,1.2398589150000043,1.640536007615484

MDH,1.233197915000013,0.6339196076147195

NADH17pp,0.0,0.5963229073845201

NH4tex,2.160164800000022,3.1601647999966946

NH4tpp,2.160164800000022,3.1601647999966946

PDH,1.8733075150000043,2.273984607606023

PFK_3,0.19573216416666564,1.1676484595805066

PGI,1.434816097083335,1.8593530607272464

PGK,-3.564233014999997,-4.964910107603915

PGL,0.7056018925000015,1.0052633461894156

PGM,-3.2205985149999905,-4.6212756076042085

PPC,0.5918716000000012,1.5918715999992519

RPE,0.31519592833333127,0.5149702307934467

RPI,-0.38244516416667035,-0.48233231539642746

SUCOAS,-0.9198485150000041,-0.32152560761545956

TALA,0.0,-0.8720291441838897

TKT1,0.1957321641666656,0.29561931539661684

TKT2,0.11946376416666554,0.21935091539682983

TPI,1.7028532254166657,2.353248196103638

XYLI2,0.0,0.026083704582296363

46 reacctions require a flux change for the evolution of the glu__L producer

.......................................................

.......................................................

gly

.......................................................

ACONTa,1.2398589150000043,0.7842411851637605

ACONTb,1.2398589150000041,0.7842411851637605

AKGDH,1.024820515000004,0.569202785163742

ATPS4rpp,13.370320970000053,14.281065829669458

CO2tex,-4.632830737500008,-4.466484163072725

CO2tpp,-4.632830737500008,-3.4883259629318553

CS,1.2398589150000043,0.7842411851637605

CYTBO3_4pp,8.410403675000031,9.077710526161967

DHAPT,0.32252132541666795,0.0

ENO,3.220598515000006,2.7649807851518857

F6PA,0.32252132541666795,0.0

FBA,1.2128609358333324,1.762234727214052

FDH4pp,0.0,0.9781582001408698

FORtppi,0.0,0.9781582001408698

FTHFD,0.0,0.97766760014087

FUM,1.2333893150000121,0.77777158516354

G6PDH2r,0.7056018925000015,0.9388712073801313

GAPD,3.5642330149999975,4.097694385222024

GHMT2r,0.22309610000000604,1.2121752000703283

GLCptspp,2.1404179895833364,2.007321585154001

GLCt2pp,0.0,0.43870530868579927

GLCtex_copy1,2.1404179895833364,2.4460268938398

GLUDy,-1.7103415000000157,-2.6994206000683945

GLYCL,0.010920900000001992,7.033928994815142e-11

GLYt2pp_copy2,0.0,-1.0

GLYtex,0.0,-1.0

GND,0.7056018925000014,0.9388712073801313

H2Otex,-9.914783537500057,-10.748936963065969

H2Otpp,-9.914783537500059,-10.748436963065497

HEX1,0.0,0.43870530868579927

Htex,-1.8376463999999728,-2.8366463999983407

ICDHyr,1.2398589150000043,0.7842411851637605

MDH,1.233197915000013,0.7775801851635427

MTHFC,0.1977522000000081,1.1759104001406677

MTHFD,0.1977522000000081,1.1759104001406677

NADH16pp,7.319156560000027,7.462056740857405

NH4tex,2.160164800000022,3.1601647999976032

NH4tpp,2.160164800000022,3.1601647999976032

O2tex,4.206049537500015,4.539952963080979

O2tpp,4.206049537500015,4.539702963080983

PDH,1.8733075150000043,1.4176897851541086

PFK,1.2128609358333324,1.762234727214052

PGCD,0.34363450000000595,1.332713600070138

PGI,1.434816097083335,1.507155686459669

PGK,-3.564233014999997,-4.097694385222024

PGL,0.7056018925000015,0.9388712073801313

PGM,-3.2205985149999905,-2.7649807851518857

PSERT,0.343634500000006,1.332713600070138

PSP_L,0.343634500000006,1.332713600070138

RPE,0.31519592833333127,0.4707088049205898

RPI,-0.38244516416667035,-0.4602016024599998

SUCDi,1.024954315000004,0.572336585163693

SUCOAS,-0.9198485150000041,-0.46423078516374205

TALA,0.0,0.07675643829352279

TKT1,0.1957321641666656,0.2734886024601884

TKT2,0.11946376416666554,0.1972202024604014

TPI,1.7028532254166657,1.930705691380905

57 reacctions require a flux change for the evolution of the gly producer

.......................................................

.......................................................

glyald

.......................................................

ACONTa,1.2398589150000043,1.172363683011309

ACONTb,1.2398589150000041,1.172363683011309

AKGDH,1.024820515000004,0.9573252830118765

ALCD19,0.0,-1.0

ATPS4rpp,13.370320970000053,14.505311033970887

CO2tex,-4.632830737500008,-4.964092257520215

CO2tpp,-4.632830737500008,-4.964092257520215

CS,1.2398589150000043,1.172363683011309

CYTBO3_4pp,8.410403675000031,9.072926715051631

DHAPT,0.32252132541666795,2.5605346228863013e-06

ENO,3.220598515000006,3.1531032830030283

F6PA,0.32252132541666795,2.5605346228863013e-06

FBA3,0.19573216416666564,0.8945454437181084

FUM,1.2333893150000121,1.1658940830112954

G3PD2,-0.027805600000000014,-1.0278056

G3PT,0.0,1.0

G6PDH2r,0.7056018925000015,1.2393491084917787

GAPD,3.5642330149999975,3.496737783003018

GLCptspp,2.1404179895833364,2.3954415224705436

GLCt2pp,0.0,0.30018672044527295

GLCtex_copy2,0.0,0.5542102533324802

GLYALDtex,0.0,-1.0

GLYALDtpp,0.0,-1.0

GND,0.7056018925000014,1.2393491084917787

H2Otex,-9.914783537500057,-10.24604505751417

H2Otpp,-9.914783537500059,-10.24604505751393

HEX1,0.0,0.30018672044527295

ICDHyr,1.2398589150000043,1.172363683011309

MDH,1.233197915000013,1.1657026830112949

NADH16pp,7.319156560000027,8.047308632039755

O2tex,4.206049537500015,4.537311057525815

O2tpp,4.206049537500015,4.537311057525815

PDH,1.8733075150000043,1.8058122830048924

PFK_3,0.19573216416666564,0.8945454437181084

PGI,1.434816097083335,1.456279134424038

PGK,-3.564233014999997,-3.496737783003018

PGL,0.7056018925000015,1.2393491084917787

PGM,-3.2205985149999905,-3.1531032830030283

RPE,0.31519592833333127,0.6710274056615677

RPI,-0.38244516416667035,-0.56036090283067

SUCDi,1.024954315000004,0.9604590830118764

SUCOAS,-0.9198485150000041,-0.8523532830118765

TALA,0.0,-0.520897540887431

TKT1,0.1957321641666656,0.3736479028306774

TKT2,0.11946376416666554,0.29737950283089026

TPI,1.7028532254166657,1.080147740086064

46 reacctions require a flux change for the evolution of the glyald producer

.......................................................

.......................................................

glyc

.......................................................

ACONTa,1.2398589150000043,1.344848438515818

ACONTb,1.2398589150000041,1.344848438515818

AKGDH,1.024820515000004,1.1298102220286745

ATPS4rpp,13.370320970000053,14.160342341191978

CO2tex,-4.632830737500008,-5.395305308517815

CO2tpp,-4.632830737500008,-5.3953052986009125

CS,1.2398589150000043,1.344848438515818

CYTBO3_4pp,8.410403675000031,8.935348683446254

DHAPT,0.32252132541666795,0.0

ENO,3.220598515000006,3.325587504394816

F6PA,0.32252132541666795,-4.958451427228283e-10

FBA3,0.19573216416666564,0.9951638213773555

FUM,1.2333893150000121,1.3383789865639792

G3PD2,-0.027805600000000014,-1.0278055993800033

G3PT,0.0,0.9999999993801936

G6PDH2r,0.7056018925000015,1.1531075727403932

GAPD,3.5642330149999975,3.669222320021196

GLCptspp,2.1404179895833364,2.567928524576978

GLCt2pp,0.0,0.19956830150100968

GLCtex_copy1,2.1404179895833364,2.767496826078362

GLYCtex,0.0,-1.0

GLYCtpp,1.9304563662165405e-18,0.9999999993801936

GND,0.7056018925000014,1.1531075727403932

H2Otex,-9.914783537500057,-9.677257372923128

H2Otpp,-9.914783537500059,-9.677257373542934

HEX1,0.0,0.19856830274062254

ICDHyr,1.2398589150000043,1.344848438515818

MDH,1.233197915000013,1.3381875856700554

NADH16pp,7.319156560000027,7.739114993120695

O2tex,4.206049537500015,4.468523604264249

O2tpp,4.206049537500015,4.468523604264249

PDH,1.8733075150000043,1.9782972461690465

PFK_3,0.19573216416666564,0.9951638213773555

PGI,1.434816097083335,1.6133892545772075

PGK,-3.564233014999997,-3.669222320021196

PGL,0.7056018925000015,1.1531075727403932

PGM,-3.2205985149999905,-3.32558750398111

RPE,0.31519592833333127,0.6135330902358639

RPI,-0.38244516416667035,-0.5316136825049876

SUCDi,1.024954315000004,1.1299408969464295

SUCOAS,-0.9198485150000041,-1.0248382534937135

TALA,0.0,-0.65026307625953

TKT1,0.1957321641666656,0.34490074511782554

TKT2,0.11946376416666554,0.26863234511803835

TPI,1.7028532254166657,1.1807635586118639

45 reacctions require a flux change for the evolution of the glyc producer

.......................................................

.......................................................

glyc__R

.......................................................

ACONTa,1.2398589150000043,2.2149046000000183

ACONTb,1.2398589150000041,2.2149046000000183

AKGDH,1.024820515000004,0.0

ATPS4rpp,13.370320970000053,13.442071399174182

CO2tex,-4.632830737500008,-4.513559572258254

CO2tpp,-4.632830737500008,-4.513559572258254

CS,1.2398589150000043,2.2149046000000183

CYTBO3_4pp,8.410403675000031,9.17186134453299

DHAPT,0.32252132541666795,0.8085246383611289

ENO,3.220598515000006,4.195644199988191

F6PA,0.32252132541666795,0.8085246383611289

FTHFLi,0.0,0.020841799175699505

FUM,1.2333893150000121,2.2084349999992368

G6PDH2r,0.7056018925000015,0.6939563710317419

GAPD,3.5642330149999975,4.528357800400049

GLCptspp,2.1404179895833364,2.6205394620374136

GLCtex_copy2,0.0,0.47912147245386416

GLXCL,0.0,1.0

GLYCAt2rpp,1.4997824761187406e-14,-1.0

GLYCAtex,1.4997824761187406e-14,-1.0

GLYCL,0.010920900000001992,4.1205605594285544e-10

GND,0.7056018925000014,0.6939563710317419

H2Otex,-9.914783537500057,-9.796012372251583

H2Otpp,-9.914783537500059,-9.795512372251583

Htex,-1.8376463999999728,-2.8366463999983527

ICDHyr,1.2398589150000043,0.2150384000000184

ICL,0.0,1.9998662

MDH,1.233197915000013,2.208109799999237

MTHFC,0.1977522000000081,0.17591040082410417

MTHFD,0.1977522000000081,0.17591040082410417

NADH16pp,7.319156560000027,7.105568544532991

O2tex,4.206049537500015,4.587028372266495

O2tpp,4.206049537500015,4.586778372266495

PDH,1.8733075150000043,2.826377600814667

PFL,0.0,0.021841799175699617

PGCD,0.34363450000000595,0.33271360041185805

PGI,1.434816097083335,1.9265830910056718

PGK,-3.564233014999997,-4.528357800400049

PGL,0.7056018925000015,0.6939563710317419

PGM,-3.2205985149999905,-4.195644199988191

PSERT,0.343634500000006,0.33271360041185805

PSP_L,0.343634500000006,0.33271360041185805

SUCDi,1.024954315000004,2.0

SUCOAS,-0.9198485150000041,0.10497199999945729

THD2pp,0.0,1.059032457518252

TPI,1.7028532254166657,2.1868565383613174

TRSARr,0.0,1.0

47 reacctions require a flux change for the evolution of the glyc__R producer

.......................................................

.......................................................

glyc3p

.......................................................

AACPS6,0.0,1.9940000000000007

ACONTa,1.2398589150000043,1.8648589150137223

ACONTb,1.2398589150000041,1.8648589150137223

ADK1,0.524992600000009,4.524127199998488

AGPAT180,0.0,0.9970000000000003

AKGDH,1.024820515000004,1.6498205150142708

ATPS4rpp,13.370320970000053,18.120320969964897

CO2tex,-4.632830737500008,-6.69533073752338

CO2tpp,-4.632830737500008,-6.69533073752338

CS,1.2398589150000043,1.8648589150137223

CYTBO3_4pp,8.410403675000031,11.535403675063097

DHAPT,0.32252132541666795,0.10377132596821648

ENO,3.220598515000006,3.845598515004945

F6PA,0.32252132541666795,0.10377132596821648

FACOAE180,0.0,1.9940000000000007

FACOAL180t2pp,0.0,1.9940000000000007

FBA,1.2128609358333324,2.211860935285507

FUM,1.2333893150000121,1.8583893150136717

G3PAT180,0.0,0.9970000000000003

G3PD2,-0.027805600000000014,-1.0278056000000002

G6PDH2r,0.7056018925000015,0.8931018924897949

GAPD,3.5642330149999975,4.1892330150049215

GLCptspp,2.1404179895833364,2.984167989039388

GLCtex_copy2,0.0,0.842749999999763

GLYC3Ptex,0.0,-1.0

GND,0.7056018925000014,0.8931018924897949

H2Otex,-9.914783537500057,-11.977783537518807

H2Otpp,-9.914783537500059,-13.977283537518327

ICDHyr,1.2398589150000043,1.8648589150137223

LPLIPAL1A180pp,0.0,0.9970000000000003

MDH,1.233197915000013,1.858197915013672

NADH16pp,7.319156560000027,9.819156560048826

O2tex,4.206049537500015,5.768799537531549

O2tpp,4.206049537500015,5.768549537531548

PA180abcpp,0.0,0.9970000000000003

PDH,1.8733075150000043,2.4983075150073053

PFK,1.2128609358333324,2.211860935285507

PGI,1.434816097083335,2.091066096549593

PGK,-3.564233014999997,-4.1892330150049215

PGL,0.7056018925000015,0.8931018924897949

PGM,-3.2205985149999905,-3.845598515004945

PIt2rpp,0.19292620000000565,1.192926199999501

PItex,0.19292620000000563,1.192926199999501

PLIPA2A180pp,0.0,0.9970000000000003

PPK,-0.7007176000000049,-4.7008521999966275

RPE,0.31519592833333127,0.44019592832691684

RPI,-0.38244516416667035,-0.4449451641633365

SUCDi,1.024954315000004,1.6499543150142708

SUCOAS,-0.9198485150000041,-1.5448485150142464

TALA,0.0,0.061499999996307224

TKT1,0.1957321641666656,0.258232164163352

TKT2,0.11946376416666554,0.18196376416356486

TPI,1.7028532254166657,1.4841032254206783

53 reacctions require a flux change for the evolution of the glyc3p producer

.......................................................

.......................................................

glyclt

.......................................................

ACONTa,1.2398589150000043,1.689872295013927

ACONTb,1.2398589150000041,1.689872295013927

AKGDH,1.024820515000004,0.4749676950144757

ATPS4rpp,13.370320970000053,13.47029420996635

CO2tex,-4.632830737500008,-4.257797287526322

CO2tpp,-4.632830737500008,-4.257797287526322

CS,1.2398589150000043,1.689872295013927

CYTBO3_4pp,8.410403675000031,8.660336775063241

DHAPT,0.32252132541666795,0.5005731476282201

ENO,3.220598515000006,3.670611895005143

F6PA,0.32252132541666795,0.5005731476282201

FUM,1.2333893150000121,1.6834026950139043

G6PDH2r,0.7056018925000015,0.9805283024906437

GAPD,3.5642330149999975,4.01424639500514

GLCptspp,2.1404179895833364,2.4112457473790396

GLCtex_copy2,0.0,0.2698277583333586

GLYCLTDx,0.0,0.9998662

GLYCLTt2rpp,0.0,-1.0

GLYCLTtex,0.0,-1.0

GND,0.7056018925000014,0.9805283024906437

H2Otex,-9.914783537500057,-9.53975008752026

H2Otpp,-9.914783537500059,-9.539750087519765

Htex,-1.8376463999999728,-2.8376463999973622

ICDHyr,1.2398589150000043,0.6900060950139271

ICL,0.0,0.9998662

MDH,1.233197915000013,1.6831220950139052

NADH16pp,7.319156560000027,7.119210080048765

O2tex,4.206049537500015,4.33101608753162

O2tpp,4.206049537500015,4.33101608753162

PDH,1.8733075150000043,2.3231870950073197

PGK,-3.564233014999997,-4.01424639500514

PGL,0.7056018925000015,0.9805283024906437

PGM,-3.2205985149999905,-3.670611895005143

RPE,0.31519592833333127,0.49848020166080653

RPI,-0.38244516416667035,-0.4740873008302956

SUCDi,1.024954315000004,1.4749676950144757

SUCOAS,-0.9198485150000041,-0.3699956950144512

TALA,0.0,0.09064213666363115

TKT1,0.1957321641666656,0.2873743008302968

TKT2,0.11946376416666554,0.21110590083050976

TPI,1.7028532254166657,1.8820388470873142

41 reacctions require a flux change for the evolution of the glyclt producer

.......................................................

.......................................................

gthrd

.......................................................

ACONTa,1.2398589150000043,1.609703197478062

ACONTb,1.2398589150000041,1.609703197478062

ACS,0.0,0.9989999999980143

ADK1,0.524992600000009,2.5219925999954524

ADSK,0.04957439999999991,1.0495744

AKGDH,1.024820515000004,0.394664797478043

ATPS4rpp,13.370320970000053,20.63014180504239

BPNT,0.049574399999999914,1.0495744

CO2tex,-4.632830737500008,-7.5301391938772415

CO2tpp,-4.632830737500008,-6.551980993719295

CS,1.2398589150000043,1.609703197478062

CYSS,0.04957439999999994,1.0495744

CYTBO3_4pp,8.410403675000031,13.205020587774825

DHAPT,0.32252132541666795,0.0

ENO,3.220598515000006,4.590442797463951

F6PA,0.32252132541666795,0.0

FBA,1.2128609358333324,2.745754234401696

FDH4pp,0.0,0.9781582001579465

FORtppi,0.0,0.9781582001579465

FTHFD,0.0,0.9776676001579461

FUM,1.2333893150000121,0.6032335974773041

G6PDH2r,0.7056018925000015,3.5261402012191123

GAPD,3.5642330149999975,6.923156397542477

GHMT2r,0.22309610000000604,1.212175200078891

GLCptspp,2.1404179895833364,2.8327835974655007

GLCt2pp,0.0,1.4571858015076127

GLCtex_copy2,0.0,2.148551409389777

GLUCYS,0.0,1.0

GLUDy,-1.7103415000000157,-4.699420600076811

GLYCL,0.010920900000001992,7.888534270250602e-11

GND,0.7056018925000014,3.5261402012191123

GTHRDabc2pp,0.0,1.0

GTHRDtex,0.0,-1.0

GTHS,0.0,1.0

H2Otex,-9.914783537500057,-19.812091993869235

H2Otpp,-9.914783537500059,-19.812091993868755

HEX1,0.0,1.4571858015076127

Htex,-1.8376463999999728,-3.83764639999794

ICDHyr,1.2398589150000043,1.609703197478062

MDH,1.233197915000013,0.6030867974773058

MTHFC,0.1977522000000081,1.1759104001577763

MTHFD,0.1977522000000081,1.1759104001577763

NADH16pp,7.319156560000027,11.76577099013886

NDPK1,0.158125000000004,1.1591249999998248

NH4tex,2.160164800000022,5.160164799996939

NH4tpp,2.160164800000022,5.160164799996939

O2tex,4.206049537500015,6.603357993887412

O2tpp,4.206049537500015,6.603357993887412

PAPSR,0.049574399999999914,1.0485744000000237

PDH,1.8733075150000043,2.243151797465609

PFK,1.2128609358333324,2.745754234401696

PGCD,0.34363450000000595,2.3327136000785256

PGI,1.434816097083335,0.763829197754001

PGK,-3.564233014999997,-6.923156397542477

PGL,0.7056018925000015,3.5261402012191123

PGM,-3.2205985149999905,-4.590442797463951

PPC,0.5918716000000012,1.5918715999998145

PPK,-0.7007176000000049,-2.7007175999932067

PSERT,0.343634500000006,2.3327136000785256

PSP_L,0.343634500000006,2.3327136000785256

RPE,0.31519592833333127,2.195554800813234

RPI,-0.38244516416667035,-1.3226246004063367

SADT2,0.04957439999999991,1.0495744

SERAT,0.04957439999999993,1.0495744

SO4t2pp,0.05044199999999991,1.050442

SO4tex,0.05044199999999991,1.050442

SUCDi,1.024954315000004,0.39579859747801927

SUCOAS,-0.9198485150000041,-0.289692797478043

SULR,0.049574399999999914,1.0495744

TALA,0.0,0.9411794362405124

TKT1,0.1957321641666656,1.1359116004065106

TKT2,0.11946376416666554,1.059643200406723

TPI,1.7028532254166657,2.9122251985680583

TRDR,0.049574399999999914,1.0485744000000237

74 reacctions require a flux change for the evolution of the gthrd producer

.......................................................

.......................................................

gua

.......................................................

ACONTa,1.2398589150000043,1.4115850163838344

ACONTb,1.2398589150000041,1.4115850163838344

ADK1,0.524992600000009,1.5279919072201897

ADSL2r,0.08960300000000408,1.0896040485398273

AICART,0.10855060000000409,1.1085516485396467

AIRC2,0.08960300000000408,1.0896040485398273

AIRC3,-0.08960300000000408,-1.0896040485398273

AKGDH,1.024820515000004,1.1965461073391337

ASPTA,-0.5855358000000094,-1.5855376364945697

ATPS4rpp,13.370320970000053,23.02696620536947

CO2tex,-4.632830737500008,-4.812141537335918

CO2tpp,-4.632830737500008,-4.812141537335918

CS,1.2398589150000043,1.4115850163838344

CYTBO3_4pp,8.410403675000031,14.769080643150653

DHAPT,0.32252132541666795,0.0

ENO,3.220598515000006,3.3923228350582804

F6PA,0.32252132541666795,0.0

FBA,1.2128609358333324,2.3428887527409845

FUM,1.2333893150000121,2.4051170039477525

G6PDH2r,0.7056018925000015,0.8697367265696634

GAPD,3.5642330149999975,5.235958690258728

GARFT,0.08915700000000398,1.0891580485398276

GHMT2r,0.22309610000000604,1.7230973042952373

GLCptspp,2.1404179895833364,2.6346633861289934

GLCt2pp,0.0,0.36897307033916427

GLCtex_copy1,2.1404179895833364,3.0036364564685605

GLNS,0.36212300000000835,3.3621250947265984

GLUDy,-1.7103415000000157,-4.210342344389484

GLUPRT,0.08964760000000407,1.0896486485398273

GLYCL,0.010920900000001992,0.5109210561473164

GMPS2,0.048743200000000084,1.0487432004702875

GND,0.7056018925000014,0.8697367265696634

GUAtex,0.0,-1.0000000004702876

GUAtpp,0.0,-1.0000000004702876

H2Otex,-9.914783537500057,-20.094103915455925

H2Otpp,-9.914783537500059,-20.0941039155735

HCO3E,0.10516900000000409,1.1051688485389106

HEX1,0.0,0.36897307033916427

Htex,-1.8376463999999728,-6.837652277529983

ICDHyr,1.2398589150000043,1.4115850163838344

IMPC,-0.10855060000000409,-1.1085516485396467

IMPD,0.048743200000000084,1.0487432004702875

MDH,1.233197915000013,2.404925595241643

MTHFC,0.1977522000000081,2.197754297079474

MTHFD,0.1977522000000081,2.197754297079474

NADH16pp,7.319156560000027,13.50610793581152

NH4tex,2.160164800000022,7.160168792303485

NH4tpp,2.160164800000022,7.160168792303485

NTD9,0.0,1.0000000007054322

O2tex,4.206049537500015,7.385365721575327

O2tpp,4.206049537500015,7.385365721575327

PDH,1.8733075150000043,2.0450330641267827

PFK,1.2128609358333324,2.3428887527409845

PGCD,0.34363450000000595,1.843635855200447

PGI,1.434816097083335,2.1338997297809223

PGK,-3.564233014999997,-5.235958690258728

PGL,0.7056018925000015,0.8697367265696634

PGM,-3.2205985149999905,-3.3923228350582804

PPK,-0.7007176000000049,-2.7017154327846162

PRAGSr,0.08964760000000409,1.0896486485398273

PRAIS,0.08964760000000409,1.0896486485398273

PRASCSi,0.0896030000000041,1.0896040485398273

PRFGS,0.08964760000000407,1.0896486485398273

PSERT,0.343634500000006,1.843635855200447

PSP_L,0.343634500000006,1.843635855200447

PUNP3,0.0,1.0000000007054322

R15BPK,0.0,0.9990004897186543

R1PK,0.0,0.9990004897186543

RPE,0.31519592833333127,0.4246187843035649

RPI,-0.38244516416667035,-0.4371571422665568

SUCDi,1.024954315000004,1.1978137073391335

SUCOAS,-0.9198485150000041,-1.0915743568444671

TALA,0.0,0.05371142798501083

TKT1,0.1957321641666656,0.2504435921516759

TKT2,0.11946376416666554,0.17417519215188904

TPI,1.7028532254166657,2.5113603227996615

76 reacctions require a flux change for the evolution of the gua producer

.......................................................

.......................................................

hxa

.......................................................

ACACT1r,0.07117599999999999,1.0711761999990834

ACACT2r,0.07117599999999998,1.0711761999990834

ACOAD1f,-0.07117599999999998,-1.0711761999990834

ACOAD2f,-0.07117599999999999,-1.0711761999990834

ACONTa,1.2398589150000043,0.5898589150127427

ACONTb,1.2398589150000041,0.5898589150127427

AKGDH,1.024820515000004,0.37482051501272423

ATPS4rpp,13.370320970000053,11.670320369971023

CO2tex,-4.632830737500008,-6.007830737523591

CO2tpp,-4.632830737500008,-6.007830737523591

CS,1.2398589150000043,0.5898589150127427

CYTBO3_4pp,8.410403675000031,7.16040367505772

DHAPT,0.32252132541666795,1.4423546587560285

ECOAH1,0.07117599999999999,1.0711761999990834

ECOAH2,0.07117599999999998,1.0711761999990834

ENO,3.220598515000006,5.570598515004938

F6PA,0.32252132541666795,1.4423546587560283

FACOAE60,0.0,1.0

FADRx,0.437178,2.437177999995416

FUM,1.2333893150000121,0.5833893150127096

G6PDH2r,0.7056018925000015,1.030601892492604

GAPD,3.5642330149999975,5.914233015004938

GLCptspp,2.1404179895833364,3.369584656250254

GLCtex_copy2,0.0,1.2281666666666762

GND,0.7056018925000014,1.030601892492604

H2Otex,-9.914783537500057,-11.289783537519384

H2Otpp,-9.914783537500059,-11.289783537518929

HACD1,0.07117599999999999,1.0711761999990834

HACD2,0.07117599999999998,1.0711761999990834

HEXt2rpp,0.0,-1.0

HXAtex,0.0,-1.0

Htex,-1.8376463999999728,-2.83764639999913

ICDHyr,1.2398589150000043,0.5898589150127427

MDH,1.233197915000013,0.5831971148514867

NADH16pp,7.319156560000027,6.717289559883789

O2tex,4.206049537500015,3.58104953752886

O2tpp,4.206049537500015,3.58104953752886

PDH,1.8733075150000043,4.223307515006136

PGI,1.434816097083335,2.338982763757599

PGK,-3.564233014999997,-5.914233015004938

PGL,0.7056018925000015,1.030601892492604

PGM,-3.2205985149999905,-5.570598515004938

RPE,0.31519592833333127,0.5318625949954594

RPI,-0.38244516416667035,-0.4907784974976721

SUCDi,1.024954315000004,0.37795511517393066

SUCOAS,-0.9198485150000041,-0.269848515012697

TALA,0.0,0.10733333333098471

TKT1,0.1957321641666656,0.3040654974976178

TKT2,0.11946376416666554,0.2277970974978416

TPI,1.7028532254166657,2.823686558753554

50 reacctions require a flux change for the evolution of the hxa producer

.......................................................

.......................................................

hxan

.......................................................

ACONTa,1.2398589150000043,1.3621075545655685

ACONTb,1.2398589150000041,1.3621075545655685

ADSL2r,0.08960300000000408,1.0896026000000347

AICART,0.10855060000000409,1.1085501999999678

AIRC2,0.08960300000000408,1.0896025999999939

AIRC3,-0.08960300000000408,-1.0896026000000347

AKGDH,1.024820515000004,1.1470691545656193

ASPTA,-0.5855358000000094,-1.5856684000000314

ATPS4rpp,13.370320970000053,21.12580241559249

CO2tex,-4.632830737500008,-4.688996998407561

CO2tpp,-4.632830737500008,-4.688996998407561

CS,1.2398589150000043,1.3621075545656192

CYTBO3_4pp,8.410403675000031,13.522774072828062

DHAPT,0.32252132541666795,0.0

ENO,3.220598515000006,3.3428370855624507

F6PA,0.32252132541666795,0.0

FBA3,0.19573216416666564,1.2967956051605825

FUM,1.2333893150000121,2.355770954565628

G6PDH2r,0.7056018925000015,0.8950317037139257

GAPD,3.5642330149999975,5.1864697855623945

GARFT,0.08915700000000398,1.0896471999999449

GHMT2r,0.22309610000000604,1.7230951000000232

GLCptspp,2.1404179895833364,2.5851782855623986

GLCt2pp,0.0,0.3979242900028419

GLCtex_copy2,0.0,0.843684585981904

GLNS,0.36212300000000835,2.362122199999988

GLUDy,-1.7103415000000157,-4.210472299999992

GLUPRT,0.08964760000000407,1.089647199999994

GLYCL,0.010920900000001992,0.5109202999999971

GND,0.7056018925000014,0.8950317037139655

H2Otex,-9.914783537500057,-18.970932523131296

H2Otpp,-9.914783537500059,-18.97093332940426

HCO3E,0.10516900000000409,1.1051674000000276

HEX1,0.0,0.396924290002842

HYXNtex,0.0,-1.0

HYXNtpp,0.0,-1.0

Htex,-1.8376463999999728,-5.837646412545951

ICDHyr,1.2398589150000043,1.3621075545655685

IMPC,-0.10855060000000409,-1.1085501999999678

MDH,1.233197915000013,2.3556241545655894

MTHFC,0.1977522000000081,2.1977514000000156

MTHFD,0.1977522000000081,2.1977514000000156

NADH16pp,7.319156560000027,12.309278318262443

NH4tex,2.160164800000022,6.160162000000014

NH4tpp,2.160164800000022,6.160162000000014

NTD11,0.0,0.9998662

O2tex,4.206049537500015,6.76223473641403

O2tpp,4.206049537500015,6.76223473641403

PDH,1.8733075150000043,1.9965549545656192

PFK_3,0.19573216416666564,1.2967956051605907

PGCD,0.34363450000000595,1.8436326999999912

PGI,1.434816097083335,2.0870708718513242

PGK,-3.564233014999997,-5.1864697855623945

PGL,0.7056018925000015,0.8950317037139655

PGM,-3.2205985149999905,-3.3428370855624507

PPK,-0.7007176000000049,-1.701567668266648

PRAGSr,0.08964760000000409,1.0896471999999449

PRAIS,0.08964760000000409,1.089647199999994

PRASCSi,0.0896030000000041,1.0896025999999939

PRFGS,0.08964760000000407,1.089647199999994

PSERT,0.343634500000006,1.8436326999999912

PSP_L,0.343634500000006,1.8436326999999912

PUNP5,0.0,1.0

R15BPK,0.0,1.000999599999989

R1PK,0.0,1.000999599999989

RPE,0.31519592833333127,0.4414832691426227

RPI,-0.38244516416667035,-0.445587634571325

SUCDi,1.024954315000004,1.1482029545656194

SUCOAS,-0.9198485150000041,-1.0420975545656574

TALA,0.0,-1.0379197705892693

TKT1,0.1957321641666656,0.25887583457131313

TKT2,0.11946376416666554,0.18260743457130957

TPI,1.7028532254166657,2.4823953409938895

73 reacctions require a flux change for the evolution of the hxan producer

.......................................................

.......................................................

idon__L

.......................................................

5DGLCNR,0.0,-1.0

ACONTa,1.2398589150000043,1.3148597131548803

ACONTb,1.2398589150000041,1.3148597131548803

AKGDH,1.024820515000004,1.0998213131559091

ATPS4rpp,13.370320970000053,13.220321369539016

CO2tex,-4.632830737500008,-4.320330737556968

CO2tpp,-4.632830737500008,-4.320332733410912

CS,1.2398589150000043,1.3148597131548803

CYTBO3_4pp,8.410403675000031,8.785403675129528

DHAPT,0.32252132541666795,0.4476044589582219

ENO,3.220598515000006,3.2955993131381502

F6PA,0.32252132541666795,0.4476044589582219

FUM,1.2333893150000121,1.3083901131557096

G6PDH2r,0.7056018925000015,0.16810249188125959

GAPD,3.5642330149999975,3.6392328152107947

GLCDpp,0.0,1.0

GLCNt2rpp,0.0,1.0

GLCptspp,2.1404179895833364,2.0883346562499128

GLCtex_copy2,0.0,0.9469166666663207

GND,0.7056018925000014,0.16810249188125959

H2Otex,-9.914783537500057,-9.602783537537311

H2Otpp,-9.914783537500059,-10.60228353753731

Htex,-1.8376463999999728,-2.8366463999926914

ICDHyr,1.2398589150000043,1.3148597131548803

IDOND,0.0,0.9999990009980017

IDONt2rpp,0.0,-1.0

IDONtex,0.0,-1.0

MDH,1.233197915000013,1.3081987131557096

NADH16pp,7.319156560000027,6.619157757827771

O2tex,4.206049537500015,4.393799537564765

O2tpp,4.206049537500015,4.393549537564764

PDH,1.8733075150000043,1.9483083131447179

PGI,1.434816097083335,1.9202321643686533

PGK,-3.564233014999997,-3.6392328152107947

PGL,0.7056018925000015,0.16810249188125959

PGM,-3.2205985149999905,-3.2955993131381502

RPE,0.31519592833333127,-0.04313700541185212

RPI,-0.38244516416667035,-0.2032786972935701

SUCDi,1.024954315000004,1.0999551131559093

SUCOAS,-0.9198485150000041,-0.9948493131563894

TALA,0.0,-0.18016646687269816

TKT1,0.1957321641666656,0.01656569729396748

TKT2,0.11946376416666554,-0.0597027027058196

TPI,1.7028532254166657,1.829936358958395

44 reacctions require a flux change for the evolution of the idon__L producer

.......................................................

.......................................................

ins

.......................................................

ACONTa,1.2398589150000043,1.4567579335371066

ACONTb,1.2398589150000041,1.4567579335371066

ADK1,0.524992600000009,1.5229929999986778

ADSL2r,0.08960300000000408,1.0896029999999886

AICART,0.10855060000000409,1.1085505999999885

AIRC2,0.08960300000000408,1.0896029999999886

AIRC3,-0.08960300000000408,-1.0896029999999886

AKGDH,1.024820515000004,1.2417195335375681

ASPTA,-0.5855358000000094,-1.585535799998724

ATPS4rpp,13.370320970000053,21.93652253292403

CO2tex,-4.632830737500008,-4.92507788382955

CO2tpp,-4.632830737500008,-4.92507788382955

CS,1.2398589150000043,1.4567579335371066

CYTBO3_4pp,8.410403675000031,13.994897967674925

DHAPT,0.32252132541666795,2.5609838463225287e-06

ENO,3.220598515000006,3.4374975335214804

F6PA,0.32252132541666795,2.5609838463225287e-06

FBA,1.2128609358333324,2.535904194405366

FUM,1.2333893150000121,2.4502883335375456

G6PDH2r,0.7056018925000015,0.8471519832270801

GAPD,3.5642330149999975,5.281132033521273

GARFT,0.08915700000000398,1.0891569999999886

GHMT2r,0.22309610000000604,1.723096099999983

GLCptspp,2.1404179895833364,2.679835772540713

GLCt2pp,0.0,1.1759567414223873

GLCtex_copy1,2.1404179895833364,3.8557925139631

GLNS,0.36212300000000835,2.362122999998559

GLUDy,-1.7103415000000157,-4.210341499995716

GLUPRT,0.08964760000000407,1.0896475999999886

GLYCL,0.010920900000001992,0.5109208999999943

GND,0.7056018925000014,0.8471519832270801

H2Otex,-9.914783537500057,-20.20703068381444

H2Otpp,-9.914783537500059,-20.20703068381444

HCO3E,0.10516900000000409,1.105168199999072

HEX1,0.0,1.1749567414223874

Htex,-1.8376463999999728,-5.837646399993934

ICDHyr,1.2398589150000043,1.4567579335371066

IMPC,-0.10855060000000409,-1.1085505999999885

INSt2pp_copy2,0.0,-1.0

INStex,0.0,-1.0

MDH,1.233197915000013,2.4500969335375444

MTHFC,0.1977522000000081,2.1977521999999774

MTHFD,0.1977522000000081,2.1977521999999774

NADH16pp,7.319156560000027,12.684751834137355

NH4tex,2.160164800000022,6.160164799994486

NH4tpp,2.160164800000022,6.160164799994486

NTD11,0.0,1.0

O2tex,4.206049537500015,6.998296683837462

O2tpp,4.206049537500015,6.998296683837462

PDH,1.8733075150000043,2.0902065335266853

PFK,1.2128609358333324,2.535904194405366

PGCD,0.34363450000000595,1.843634499999793

PGI,1.434816097083335,3.0076405307360203

PGK,-3.564233014999997,-5.281132033521273

PGL,0.7056018925000015,0.8471519832270801

PGM,-3.2205985149999905,-3.4374975335214804

PPK,-0.7007176000000049,-1.700717999996621

PRAGSr,0.08964760000000409,1.0896475999999886

PRAIS,0.08964760000000409,1.0896475999999886

PRASCSi,0.0896030000000041,1.0896029999999886

PRFGS,0.08964760000000407,1.0896475999999886

PRPPS,0.1865784000000047,1.1855783999999874

PSERT,0.343634500000006,1.843634499999793

PSP_L,0.343634500000006,1.843634499999793

RPE,0.31519592833333127,-0.2571040111815617

RPI,-0.38244516416667035,-1.0962951944091002

SUCDi,1.024954315000004,1.2438533335375683

SUCOAS,-0.9198485150000041,-1.1367475335380484

TALA,0.0,-0.2871499697575529

TKT1,0.1957321641666656,-0.09041780559088727

TKT2,0.11946376416666554,-0.16668620559067437

TPI,1.7028532254166657,2.7043777195560694

72 reacctions require a flux change for the evolution of the ins producer

.......................................................

.......................................................

lac__D

.......................................................

ACONTa,1.2398589150000043,1.1398468237607333

ACONTb,1.2398589150000041,1.1398468237607333

AKGDH,1.024820515000004,0.9248277166660139

ATPS4rpp,13.370320970000053,12.570400736759368

CO2tex,-4.632830737500008,-4.382833137752187

CO2tpp,-4.632830737500008,-4.382833137752187

CS,1.2398589150000043,1.1398496240292415

CYTBO3_4pp,8.410403675000031,7.910464201300366

D_LACt2pp,0.0,-1.0000001091013704

D_LACtex,0.0,-1.0000001091013704

DHAPT,0.32252132541666795,0.7641913662048039

ENO,3.220598515000006,4.120593714544157

F6PA,0.32252132541666795,0.7641905061222711

FUM,1.2333893150000121,1.1333977423069772

G6PDH2r,0.7056018925000015,0.7556056736331028

GAPD,3.5642330149999975,4.4642218579026824

GLCptspp,2.1404179895833364,2.598747624031665

GLCtex_copy1,2.1404179895833364,2.598749388112839

GND,0.7056018925000014,0.7556075141135742

H2Otex,-9.914783537500057,-9.664774870903273

H2Otpp,-9.914783537500059,-9.664784737626405

Htex,-1.8376463999999728,-2.837659075246279

ICDHyr,1.2398589150000043,1.1398468237607333

LDH_D,0.0,-0.9999989661345581

MDH,1.233197915000013,1.1332429248565088

NADH16pp,7.319156560000027,6.91919106208029

O2tex,4.206049537500015,3.956082623778109

O2tpp,4.206049537500015,3.956082623778109

PDH,1.8733075150000043,1.7733581098123161

PGI,1.434816097083335,1.8431431505136369

PGK,-3.564233014999997,-4.4642218579026824

PGL,0.7056018925000015,0.7556056736331028

PGM,-3.2205985149999905,-4.120593714544157

RPE,0.31519592833333127,0.34853013073161376

RPI,-0.38244516416667035,-0.3991156832961127

SUCDi,1.024954315000004,0.9259647169730365

SUCOAS,-0.9198485150000041,-0.8198583564103972

TALA,0.0,0.017667162986654716

TKT1,0.1957321641666656,0.2123993271533252

TKT2,0.11946376416666554,0.13613080357828852

TPI,1.7028532254166657,2.144507863568916

41 reacctions require a flux change for the evolution of the lac__D producer

.......................................................

.......................................................

lac__L

.......................................................

AACTOOR,0.0,0.022332670746672875

ACONTa,1.2398589150000043,1.153856330694855

ACONTb,1.2398589150000041,1.153856330694855

AKGDH,1.024820515000004,0.9388174010766301

AOBUTDs,0.0,0.022332670746672875

ATPS4rpp,13.370320970000053,14.497167263020266

CO2tex,-4.632830737500008,-4.957517246042869

CO2tpp,-4.632830737500008,-4.957026646042869

CS,1.2398589150000043,1.153856330694855

CYTBO3_4pp,8.410403675000031,9.05977781278363

DHAPT,0.32252132541666795,0.0

ENO,3.220598515000006,3.1569294006791635

F6PA,0.32252132541666795,0.0

FBA3,0.19573216416666564,0.8885479945749614

FUM,1.2333893150000121,1.1473863406056006

G6PDH2r,0.7056018925000015,1.25406212688634

GAPD,3.5642330149999975,3.511974523299712

GHMT2r,0.22309610000000604,0.23450794051153168

GLCptspp,2.1404179895833364,2.3992696007051233

GLCt2pp,0.0,0.2952631091513229

GLCtex_copy1,2.1404179895833364,2.69453270985692

GLUDy,-1.7103415000000157,-1.7217541479961689

GLYAT,0.0,0.022332670746672875

GLYCL,0.010920900000001992,0.0

GND,0.7056018925000014,1.25406212688634

H2Otex,-9.914783537500057,-10.239969814254597

H2Otpp,-9.914783537500059,-10.239469814254596

HEX1,0.0,0.29431906598149415

Htex,-1.8376463999999728,-2.8366460747950675

ICDHyr,1.2398589150000043,1.153856330694855

L_LACt2rpp,0.0,-1.0

L_LACtex,0.0,-1.0

LALDO3,0.0,1.0

LCADi,0.0,1.0

MDH,1.233197915000013,1.1695276137272477

MGSA,0.0,0.9776673292533271

MOX,0.00032519999999967104,-0.022007473121646942

NADH16pp,7.319156560000027,8.053043211707207

O2tex,4.206049537500015,4.5309866063918145

O2tpp,4.206049537500015,4.530736606391814

PDH,1.8733075150000043,1.8096362218208593

PFK_3,0.19573216416666564,0.8885479945749614

PGCD,0.34363450000000595,0.35504512262054816

PGI,1.434816097083335,1.4395265398002774

PGK,-3.564233014999997,-3.511974523299712

PGL,0.7056018925000015,1.25406212688634

PGM,-3.2205985149999905,-3.1569294006791635

PSERT,0.343634500000006,0.35504512262054816

PSP_L,0.343634500000006,0.35504512262054816

RPE,0.31519592833333127,0.6808359474367691

RPI,-0.38244516416667035,-0.5652653794500291

SUCDi,1.024954315000004,0.940951201076424

SUCOAS,-0.9198485150000041,-0.8348453336871894

TALA,0.0,-0.5099958208566833

TKT1,0.1957321641666656,0.3785521737182781

TKT2,0.11946376416666554,0.302283773718491

TPI,1.7028532254166657,1.0964803987803597

57 reacctions require a flux change for the evolution of the lac__L producer

.......................................................

.......................................................

mal__L

.......................................................

ACONTa,1.2398589150000043,1.3181542488498934

ACONTb,1.2398589150000041,1.3181542488498934

AKGDH,1.024820515000004,1.1031158488503552

ATPS4rpp,13.370320970000053,13.21373073114411

CO2tex,-4.632830737500008,-3.8285689865357577

CO2tpp,-4.632830737500008,-3.8285689865357577

CS,1.2398589150000043,1.3181542488498934

CYTBO3_4pp,8.410403675000031,8.801879487186502

DHAPT,0.32252132541666795,0.0

ENO,3.220598515000006,4.298893848834509

F6PA,0.32252132541666795,0.0

FBA,1.2128609358333324,2.080054610742695

FUM,1.2333893150000121,1.311684648849589

G6PDH2r,0.7056018925000015,0.6664537970449188

GAPD,3.5642330149999975,4.6425283488342295

GLCptspp,2.1404179895833364,2.5412346488375883

GLCt2pp,0.0,0.13180632508482315

GLCtex_copy2,0.0,0.5316229843390752

GND,0.7056018925000014,0.6664537970449188

H2Otex,-9.914783537500057,-10.111021100619622

H2Otpp,-9.914783537500059,-10.110521100619621

HEX1,0.0,0.13080632508482326

Htex,-1.8376463999999728,-3.836645714093821

ICDHyr,1.2398589150000043,1.3181542488498934

MALt3pp,0.0,1.0

MALtex,0.0,-1.0

MDH,1.233197915000013,0.3115378488495877

NADH16pp,7.319156560000027,7.630337038336145

O2tex,4.206049537500015,4.402037443593251

O2tpp,4.206049537500015,4.401787443593251

PDH,1.8733075150000043,1.951602848839715

PFK,1.2128609358333324,2.080054610742695

PGI,1.434816097083335,2.005587176877493

PGK,-3.564233014999997,-4.6425283488342295

PGL,0.7056018925000015,0.6664537970449188

PGM,-3.2205985149999905,-4.298893848834509

PPC,0.5918716000000012,1.5918715999982846

RPE,0.31519592833333127,0.28909719803044986

RPI,-0.38244516416667035,-0.3693957990149273

SUCDi,1.024954315000004,1.1052496488503554

SUCOAS,-0.9198485150000041,-0.998143505901562

TALA,0.0,-0.014049365151547172

TKT1,0.1957321641666656,0.18268279901511847

TKT2,0.11946376416666554,0.10641439901533138

TPI,1.7028532254166657,2.248525574909537

45 reacctions require a flux change for the evolution of the mal__L producer

.......................................................

.......................................................

orn

.......................................................

ACGK,0.05915840000000002,1.0591583999994327

ACGS,0.05915840000000002,1.0591583999994327

ACKr,0.11651519999999993,1.1155151999946433

ACODA,0.059158400000000014,1.0591583999994327

ACONTa,1.2398589150000043,1.8664086975515217

ACONTb,1.2398589150000041,1.8664086975515217

ACOTA,-0.05915840000000002,-1.0591583999994327

AGPR,-0.05915840000000003,-1.0591583999994327

AKGDH,1.024820515000004,0.6513702975520707

ARGORNt7pp,0.0,1.0

ARGt3pp,0.0,1.0

ATPS4rpp,13.370320970000053,15.117221404891138

CO2tex,-4.632830737500008,-5.6992051938703066

CO2tpp,-4.632830737500008,-5.6992051938703066

CS,1.2398589150000043,1.8664086975515217

CYTBO3_4pp,8.410403675000031,9.543152587751203

DHAPT,0.32252132541666795,6.122635731742321e-10

ENO,3.220598515000006,4.847148297542735

F6PA,0.32252132541666795,6.122635731742321e-10

FBA3,0.19573216416666564,1.1327408621220314

FUM,1.2333893150000121,0.8599390975514969

G6PDH2r,0.7056018925000015,1.892327001221844

GAPD,3.5642330149999975,5.19078279754273

GLCptspp,2.1404179895833364,3.0894890969325672

GLCt2pp,0.0,0.06199130204145353

GLCtex_copy1,2.1404179895833364,3.1514803989740208

GLUDy,-1.7103415000000157,-3.710341499998105

GND,0.7056018925000014,1.892327001221844

H2Otex,-9.914783537500057,-12.981657993864204

H2Otpp,-9.914783537500059,-12.981157993863723

HEX1,0.0,0.06199130204145353

Htex,-1.8376463999999728,-2.8366463999973863

ICDHyr,1.2398589150000043,1.8664086975515217

MDH,1.233197915000013,0.8597922975514964

NADH16pp,7.319156560000027,8.823489490199131

NH4tex,2.160164800000022,4.16016479999728

NH4tpp,2.160164800000022,4.16016479999728

O2tex,4.206049537500015,4.7726739938756015

O2tpp,4.206049537500015,4.772423993875601

ORNtex,0.0,-1.0

PDH,1.8733075150000043,2.499857297544914

PFK_3,0.19573216416666564,1.1327408621220314

PGI,1.434816097083335,1.2591533977521767

PGK,-3.564233014999997,-5.19078279754273

PGL,0.7056018925000015,1.892327001221844

PGM,-3.2205985149999905,-4.847148297542735

PPC,0.5918716000000012,1.5918715999992687

PTAr,-0.11651519999999993,-1.1155151999946433

RPE,0.31519592833333127,1.1063460008149415

RPI,-0.38244516416667035,-0.7780202004073605

SUCDi,1.024954315000004,0.6545040975520714

SUCOAS,-0.9198485150000041,-0.546398297552046

TALA,0.0,-0.5414336617146671

TKT1,0.1957321641666656,0.5913072004073643

TKT2,0.11946376416666554,0.5150388004075772

TPI,1.7028532254166657,2.318340598567577

56 reacctions require a flux change for the evolution of the orn producer

.......................................................

.......................................................

pro__L

.......................................................

ACONTa,1.2398589150000043,1.7184051854120526

ACONTb,1.2398589150000041,1.7184051854120526

AKGDH,1.024820515000004,0.5033667854120982

ATPS4rpp,13.370320970000053,14.413204022533591

CO2tex,-4.632830737500008,-5.329668434686482

CO2tpp,-4.632830737500008,-5.329668434686482

CS,1.2398589150000043,1.7184051854120983

CYTBO3_4pp,8.410403675000031,8.804128427060398

DHAPT,0.32252132541666795,0.0

ENO,3.220598515000006,4.699131845990223

F6PA,0.32252132541666795,0.0

FBA3,0.19573216416666564,1.0463113144721774

FUM,1.2333893150000121,0.7119347854120406

G5SADs,0.044211,1.044211

G5SD,0.044211,1.044211

G6PDH2r,0.7056018925000015,1.9668131178719932

GAPD,3.5642330149999975,5.042764545990167

GLCptspp,2.1404179895833364,2.941473045990212

GLCt2pp,0.0,0.14840571027259397

GLCtex_copy1,2.1404179895833364,3.0888787562628295

GLU5K,0.04421099999999999,1.044211

GLUDy,-1.7103415000000157,-2.7103384999999207

GND,0.7056018925000014,1.9668131178720478

H2Otex,-9.914783537500057,-12.611600828044175

H2Otpp,-9.914783537500059,-12.611601895264357

HEX1,0.0,0.14740571027259428

Htex,-1.8376463999999728,-2.837646934440272

ICDHyr,1.2398589150000043,1.7184051854120526

MDH,1.233197915000013,0.7117879854121156

NADH16pp,7.319156560000027,8.2343350416483

NH4tex,2.160164800000022,3.1601619999999

NH4tpp,2.160164800000022,3.1601619999999

O2tex,4.206049537500015,4.402911913530147

O2tpp,4.206049537500015,4.402911913530147

P5CR,0.044211,1.044211

PDH,1.8733075150000043,2.3528525854120983

PFK_3,0.19573216416666564,1.0463113144721377

PGI,1.434816097083335,1.1220656383907226

PGK,-3.564233014999997,-5.042764545990167

PGL,0.7056018925000015,1.9668131178720478

PGM,-3.2205985149999905,-4.699131845990223

PPC,0.5918716000000012,1.591871199999996

PROt2rpp,0.0,-1.0

PROtex,0.0,-1.0

RPE,0.31519592833333127,1.1560042119147056

RPI,-0.38244516416667035,-0.8028481059573096

SUCDi,1.024954315000004,0.5046343854120982

SUCOAS,-0.9198485150000041,-0.3983951854121415

TALA,0.0,-0.430175008514766

TKT1,0.1957321641666656,0.6161363059574114

TKT2,0.11946376416666554,0.5398679059574079

TPI,1.7028532254166657,2.2319110503054844

52 reacctions require a flux change for the evolution of the pro__L producer

.......................................................

.......................................................

ptrc

.......................................................

ACGK,0.05915840000000002,1.0591584

ACGS,0.05915840000000002,1.0591584

ACKr,0.11651519999999993,1.1165151999985814

ACODA,0.059158400000000014,1.0591584

ACONTa,1.2398589150000043,1.8417682004646978

ACONTb,1.2398589150000041,1.8417682004646978

ACOTA,-0.05915840000000002,-1.0591584

AGPR,-0.05915840000000003,-1.0591584

AKGDH,1.024820515000004,0.6267298004646785

ARGORNt7pp,0.0,1.0

ARGt3pp,0.0,1.0

ATPS4rpp,13.370320970000053,15.166501999065574

CO2tex,-4.632830737500008,-6.637603551153391

CO2tpp,-4.632830737500008,-6.637603551153391

CS,1.2398589150000043,1.8417682004646978

CYTBO3_4pp,8.410403675000031,9.419949302317171

DHAPT,0.32252132541666795,2.5605048565857658e-06

ENO,3.220598515000006,4.822507800456439

F6PA,0.32252132541666795,2.5605048565857658e-06

FBA,1.2128609358333324,2.135493517262338

FUM,1.2333893150000121,0.8352986004646523

G6PDH2r,0.7056018925000015,1.904646849765394

GAPD,3.5642330149999975,5.166142300456419

GLCptspp,2.1404179895833364,3.0648460399536983

GLCt2pp,0.0,0.07636741856775053

GLCtex_copy2,0.0,0.9997954689381126

GLUDy,-1.7103415000000157,-3.7103414999987825

GND,0.7056018925000014,1.904646849765394

H2Otex,-9.914783537500057,-12.920056351146904

H2Otpp,-9.914783537500059,-12.91955635114776

HEX1,0.0,0.07536741856775064

ICDHyr,1.2398589150000043,1.8417682004646978

MDH,1.233197915000013,0.8351072004646527

NADH16pp,7.319156560000027,8.726792901852491

NH4tex,2.160164800000022,4.160164799998512

NH4tpp,2.160164800000022,4.160164799998512

O2tex,4.206049537500015,4.711072351157918

O2tpp,4.206049537500015,4.710822351158585

ORNDC,0.0,1.0

PDH,1.8733075150000043,2.475216800458281

PFK,1.2128609358333324,2.135493517262338

PGI,1.434816097083335,1.235566608756055

PGK,-3.564233014999997,-5.166142300456419

PGL,0.7056018925000015,1.904646849765394

PGM,-3.2205985149999905,-4.822507800456439

PPC,0.5918716000000012,1.5918715999992479

PTAr,-0.11651519999999993,-1.1165151999985814

PTRCORNt7pp,0.0,-1.0

PTRCtex,0.0,-1.0

RPE,0.31519592833333127,1.1145592331773142

RPI,-0.38244516416667035,-0.782126816588538

SUCDi,1.024954315000004,0.6279974004646787

SUCOAS,-0.9198485150000041,-0.5217578004646786

TALA,0.0,0.3986816524219175

TKT1,0.1957321641666656,0.5954138165885506

TKT2,0.11946376416666554,0.5191454165887636

TPI,1.7028532254166657,2.3039670419338276

57 reacctions require a flux change for the evolution of the ptrc producer

.......................................................

.......................................................

pyr

.......................................................

ACONTa,1.2398589150000043,0.9648588421349439

ACONTb,1.2398589150000041,0.9648588421349439

AKGDH,1.024820515000004,0.7498203712205448

ATPS4rpp,13.370320970000053,12.920321427044223

CO2tex,-4.632830737500008,-3.945329476143378

CO2tpp,-4.632830737500008,-3.94532947384407

CS,1.2398589150000043,0.9648588421349439

CYTBO3_4pp,8.410403675000031,8.035403563260788

DHAPT,0.32252132541666795,0.6621051206845545

ENO,3.220598515000006,3.9455990518710005

F6PA,0.32252132541666795,0.6621051206845545

FUM,1.2333893150000121,0.9583893154022007

G6PDH2r,0.7056018925000015,0.8431010240116653

GAPD,3.5642330149999975,4.289233145631732

GLCptspp,2.1404179895833364,2.525834656250254

GLCtex_copy1,2.1404179895833364,2.5258346562504435

GND,0.7056018925000014,0.8431010240116653

H2Otex,-9.914783537500057,-10.227783535770607

H2Otpp,-9.914783537500059,-10.22728353698733

Htex,-1.8376463999999728,-2.836644530714871

ICDHyr,1.2398589150000043,0.9648588421349439

MDH,1.233197915000013,0.9581979157440509

NADH16pp,7.319156560000027,7.219156593017664

O2tex,4.206049537500015,4.018799481726197

O2tpp,4.206049537500015,4.0185494816303935

PDH,1.8733075150000043,1.5983074106092772

PGI,1.434816097083335,1.6827336320948816

PGK,-3.564233014999997,-4.289233145631732

PGL,0.7056018925000015,0.8431010240116653

PGM,-3.2205985149999905,-3.9455990518710005

PYRt2rpp,0.0,-1.0

PYRtex,0.0,-1.0

RPE,0.31519592833333127,0.4068619550056554

RPI,-0.38244516416667035,-0.42827826900646826

SUCDi,1.024954315000004,0.7510879702431241

SUCOAS,-0.9198485150000041,-0.6458483669818632

TALA,0.0,0.044833013336052635

TKT1,0.1957321641666656,0.24156517750272125

TKT2,0.11946376416666554,0.16529677750293417

TPI,1.7028532254166657,2.042436796819773

40 reacctions require a flux change for the evolution of the pyr producer

.......................................................

.......................................................

ser__L

.......................................................

ACONTa,1.2398589150000043,0.9013184553630913

ACONTb,1.2398589150000041,0.9013184553630913

AKGDH,1.024820515000004,0.68628005536364

ATPS4rpp,13.370320970000053,14.047405129821897

CO2tex,-4.632830737500008,-4.286474187706176

CO2tpp,-4.632830737500008,-4.286474187706176

CS,1.2398589150000043,0.9013184553630913

CYTBO3_4pp,8.410403675000031,8.717690575423582

DHAPT,0.32252132541666795,0.0

ENO,3.220598515000006,2.882058055354264

F6PA,0.32252132541666795,0.0

FBA,1.2128609358333324,1.7535659729626314

FUM,1.2333893150000121,0.8948488553636238

G6PDH2r,0.7056018925000015,1.374872482454269

GAPD,3.5642330149999975,4.225690635076981

GLCptspp,2.1404179895833364,2.124398855356949

GLCt2pp,0.0,0.458293042589597

GLCtex_copy1,2.1404179895833364,2.582691897946546

GLUDy,-1.7103415000000157,-2.710339579720994

GND,0.7056018925000014,1.374872482454269

H2Otex,-9.914783537500057,-10.568426987700718

H2Otpp,-9.914783537500059,-10.568426987700263

HEX1,0.0,0.458293042589597

Htex,-1.8376463999999728,-2.8376463999979933

ICDHyr,1.2398589150000043,0.9013184553630913

MDH,1.233197915000013,0.8946574553636235

NADH16pp,7.319156560000027,7.964983920059941

NH4tex,2.160164800000022,3.160164799997966

NH4tpp,2.160164800000022,3.160164799997966

O2tex,4.206049537500015,4.3596929877117905

O2tpp,4.206049537500015,4.3596929877117905

PDH,1.8733075150000043,1.5347632148021337

PFK,1.2128609358333324,1.7535659729626314

PGCD,0.34363450000000595,1.3436325797227175

PGI,1.434816097083335,1.2078194154922812

PGK,-3.564233014999997,-4.225690635076981

PGL,0.7056018925000015,1.374872482454269

PGM,-3.2205985149999905,-2.882058055354264

PSERT,0.343634500000006,1.3436325797227175

PSP_L,0.343634500000006,1.3436325797227175

RPE,0.31519592833333127,0.7613763216365617

RPI,-0.38244516416667035,-0.6055353608181804

SERt2rpp,0.0,-1.0

SERtex,0.0,-1.0

SUCDi,1.024954315000004,0.68654765536364

SUCOAS,-0.9198485150000041,-0.58130805536364

TALA,0.0,0.2220901966515087

TKT1,0.1957321641666656,0.41882236081817437

TKT2,0.11946376416666554,0.3425539608183873

TPI,1.7028532254166657,1.9220369371292974

50 reacctions require a flux change for the evolution of the ser__L producer

.......................................................

.......................................................

succ

.......................................................

ACONTa,1.2398589150000043,1.4167167311634157

ACONTb,1.2398589150000041,1.4167167311634157

AKGDH,1.024820515000004,1.2016783034010015

ATPS4rpp,13.370320970000053,13.016605367534703

CO2tex,-4.632830737500008,-4.074975337077034

CO2tpp,-4.632830737500008,-4.07497533696404

CS,1.2398589150000043,1.4167167311634157

CYTBO3_4pp,8.410403675000031,8.294692539914529

DHAPT,0.32252132541666795,0.0

ENO,3.220598515000006,4.397456370402531

F6PA,0.32252132541666795,-2.37287167692537e-10

FBA3,0.19573216416666564,1.1224205480272456

FUM,1.2333893150000121,0.4102470441047149

G6PDH2r,0.7056018925000015,0.6171729984433796

GAPD,3.5642330149999975,4.741090830296927

GLCptspp,2.1404179895833364,2.6397971399831572

GLCt2pp,0.0,0.07431159574551494

GLCtex_copy2,0.0,0.5726907461453359

GND,0.7056018925000014,0.6171729984433796

H2Otex,-9.914783537500057,-10.356927832598274

H2Otpp,-9.914783537500059,-10.356927832360986

HEX1,0.0,0.07431159586415881

Htex,-1.8376463999999728,-3.8376459203727933

ICDHyr,1.2398589150000043,1.4167167311634157

MDH,1.233197915000013,0.41005564423985796

NADH16pp,7.319156560000027,8.026587636632172

O2tex,4.206049537500015,4.148193969957264

O2tpp,4.206049537500015,4.148193969957264

PDH,1.8733075150000043,2.0501653214829627

PFK_3,0.19573216416666564,1.1224205480272456

PGI,1.434816097083335,2.096935737285293

PGK,-3.564233014999997,-4.741090830296927

PGL,0.7056018925000015,0.6171729984433796

PGM,-3.2205985149999905,-4.397456370402531

PPC,0.5918716000000012,1.5918716304207379

RPE,0.31519592833333127,0.2562433463365792

RPI,-0.38244516416667035,-0.3529688521072587

SUCCt3pp,0.0,0.9990000002768354

SUCCtex,0.0,-1.0

SUCDi,1.024954315000004,0.20294590328235795

SUCOAS,-0.9198485150000041,-1.0967063019396446

TALA,0.0,-0.9561646748590624

TKT1,0.1957321641666656,0.16625587316818313

TKT2,0.11946376416666554,0.08998747316839606

TPI,1.7028532254166657,2.3060202837254344

45 reacctions require a flux change for the evolution of the succ producer

.......................................................

.......................................................

thr__L

.......................................................

ACONTa,1.2398589150000043,1.27503292752668

ACONTb,1.2398589150000041,1.27503292752668

AKGDH,1.024820515000004,1.05999452752668

ASAD,-0.21381520000000004,-1.2138152000000244

ASPK,0.2138152,1.2138152000000244

ASPTA,-0.5855358000000094,-1.5855357999994604

ATPS4rpp,13.370320970000053,15.299972944942182

CO2tex,-4.632830737500008,-5.220765768809494

CO2tpp,-4.632830737500008,-5.220765768809494

CS,1.2398589150000043,1.27503292752668

CYTBO3_4pp,8.410403675000031,9.586273737627861

DHAPT,0.32252132541666795,4.386830898539529e-10

ENO,3.220598515000006,4.255772527519429

F6PA,0.32252132541666795,4.386830898539529e-10

FBA,1.2128609358333324,1.804900434781667

FUM,1.2333893150000121,1.268563327526116

G6PDH2r,0.7056018925000015,2.188014886235516

GAPD,3.5642330149999975,4.599407027519431

GLCptspp,2.1404179895833364,2.4981133270820854

GLCt2pp,0.0,0.4069605010492361

GLCtex_copy1,2.1404179895833364,2.9050738281313215

GLUDy,-1.7103415000000157,-2.710341499999274

GND,0.7056018925000014,2.188014886235516

H2Otex,-9.914783537500057,-11.50271856880441

H2Otpp,-9.914783537500059,-11.502718568803916

HEX1,0.0,0.4069605010492361

HSDy,-0.13962539999999996,-1.1396254

HSK,0.10884319999999999,1.1088432

Htex,-1.8376463999999728,-2.837646399997928

ICDHyr,1.2398589150000043,1.27503292752668

MDH,1.233197915000013,1.2684165275261163

NADH16pp,7.319156560000027,8.45985261010118

NH4tex,2.160164800000022,3.1601647999982507

NH4tpp,2.160164800000022,3.1601647999982507

O2tex,4.206049537500015,4.79398456881393

O2tpp,4.206049537500015,4.79398456881393

PDH,1.8733075150000043,1.9084815275202636

PFK,1.2128609358333324,1.804900434781667

PGI,1.434816097083335,0.7170589418958055

PGK,-3.564233014999997,-4.599407027519431

PGL,0.7056018925000015,2.188014886235516

PGM,-3.2205985149999905,-4.255772527519429

PPC,0.5918716000000012,1.5918716000000246

RPE,0.31519592833333127,1.3034712574907197

RPI,-0.38244516416667035,-0.8765828287452552

SUCDi,1.024954315000004,1.0601283275266802

SUCOAS,-0.9198485150000041,-0.9550225275266555

TALA,0.0,0.4931376645786203

THRS,0.1088432,1.1088432

THRt2rpp,0.0,-1.001

THRtex,0.0,-1.0

TKT1,0.1957321641666656,0.6898698287452534

TKT2,0.11946376416666554,0.6136014287454663

TPI,1.7028532254166657,1.9733713993869828

54 reacctions require a flux change for the evolution of the thr__L producer

.......................................................

.......................................................

thymd

.......................................................

ACONTa,1.2398589150000043,1.3376614242860987

ACONTb,1.2398589150000041,1.3376614242860987

ADK1,0.524992600000009,1.5239929999986352

AKGDH,1.024820515000004,1.1226230242866475

ASPCT,0.06615899999999995,1.066159

ASPTA,-0.5855358000000094,-1.5855357999990256

ATPS4rpp,13.370320970000053,17.174715551420398

CBMKr,0.12531739999999997,1.1253174000000001

CO2tex,-4.632830737500008,-5.627336610706669

CO2tpp,-4.632830737500008,-5.627336610706669

CS,1.2398589150000043,1.3376614242860987

CYTBO3_4pp,8.410403675000031,11.399415421424141

DHAPT,0.32252132541666795,2.560597925027608e-06

DHFR,0.005367000000000067,1.005367

DHORD2,0.06615899999999995,1.0661590000000218

DHORTS,-0.06615899999999995,-1.066159

DUTPDP,0.0052332,1.0052332

ENO,3.220598515000006,4.318401024277271

F6PA,0.32252132541666795,2.560597925027608e-06

FBA,1.2128609358333324,2.2997645643980125

FUM,1.2333893150000121,1.331191824286445

G6PDH2r,0.7056018925000015,1.906700237853992

GAPD,3.5642330149999975,5.162035524277164

GHMT2r,0.22309610000000604,0.7230960999998935

GLCptspp,2.1404179895833364,2.5607392636820308

GLCt2pp,0.0,1.412096371431074

GLCtex_copy1,2.1404179895833364,3.9728356351131047

GLUDy,-1.7103415000000157,-3.210341499997771

GLYCL,0.010920900000001992,0.5109208999999044

GND,0.7056018925000014,1.906700237853992

H2Otex,-9.914783537500057,-16.90978941070009

H2Otpp,-9.914783537500059,-16.90928941069961

HEX1,0.0,1.412096371431074

Htex,-1.8376463999999728,-3.8366463999975804

ICDHyr,1.2398589150000043,1.3376614242860987

MDH,1.233197915000013,1.3310450242864444

NADH16pp,7.319156560000027,9.210365797137493

NDPK2,0.08505659999999997,1.0850565999985815

NH4tex,2.160164800000022,4.160164799997411

NH4tpp,2.160164800000022,4.160164799997411

NTD5,0.0,1.0

O2tex,4.206049537500015,5.7008054107120705

O2tpp,4.206049537500015,5.70055541071207

OMPDC,0.06615899999999997,1.066159

ORPT,-0.06615899999999995,-1.066159

PDH,1.8733075150000043,0.9711100242796818

PFK,1.2128609358333324,2.2997645643980125

PGCD,0.34363450000000595,0.8436344999998935

PGI,1.434816097083335,2.0661353972591128

PGK,-3.564233014999997,-5.162035524277164

PGL,0.7056018925000015,1.906700237853992

PGM,-3.2205985149999905,-4.318401024277271

POR5,0.021750999999999996,1.021751

PPC,0.5918716000000012,1.5918715999986794

PPK,-0.7007176000000049,-2.7007179999968325

PRPPS,0.1865784000000047,1.1865783999997441

PSERT,0.343634500000006,0.8436344999998935

PSP_L,0.343634500000006,0.8436344999998935

RNTR4c2,0.0052331999999999995,1.0052332

RPE,0.31519592833333127,0.44926149190316556

RPI,-0.38244516416667035,-1.4494779459512845

SUCDi,1.024954315000004,1.1227568242866257

SUCOAS,-0.9198485150000041,-1.0176510242866474

TALA,0.0,0.06603278178467675

THMDt2pp_copy2,0.0,-1.0

THMDtex,0.0,-1.0

TKT1,0.1957321641666656,0.26276494595147626

TKT2,0.11946376416666554,0.18649654595168927

TMDS,0.0052331999999999995,1.0052332

TPI,1.7028532254166657,2.468238089162739

UMPK,0.07560779999999996,1.0756077999992908

71 reacctions require a flux change for the evolution of the thymd producer

.......................................................

.......................................................

trp__L

.......................................................

ACONTa,1.2398589150000043,1.3571684511437232

ACONTb,1.2398589150000041,1.3571684511437232

ADK1,0.524992600000009,1.5229925999990641

AKGDH,1.024820515000004,1.142130051143705

ANPRT,0.011368599999999998,1.0113686

ANS,0.011368599999999998,1.0113686

ATPS4rpp,13.370320970000053,17.135701097708342

CHORS,0.07622380000000006,1.076223799999787

CO2tex,-4.632830737500008,-6.426104577850195

CO2tpp,-4.632830737500008,-6.426104577850195

CS,1.2398589150000043,1.3571684511437232

CYTBO3_4pp,8.410403675000031,10.996951355711564

DDPA,0.07622380000000009,1.076223799999787

DHAPT,0.32252132541666795,3.200551620619052e-06

DHQS,0.07622380000000006,1.076223799999787

DHQTi,0.07622380000000006,1.076223799999787

ENO,3.220598515000006,5.3379080511348915

F6PA,0.32252132541666795,3.200551620619052e-06

FBA3,0.19573216416666564,1.5023475184447717

FUM,1.2333893150000121,1.3506988511436848

G6PDH2r,0.7056018925000015,1.1469471244256932

GAPD,3.5642330149999975,5.681542551134877

GLCptspp,2.1404179895833364,2.580245650585388

GLCt2pp,0.0,1.6923846457186755

GLCtex_copy1,2.1404179895833364,4.272630296304063

GLNS,0.36212300000000835,1.362122999999522

GND,0.7056018925000014,1.1469471244256932

H2Otex,-9.914783537500057,-19.708057377845098

H2Otpp,-9.914783537500059,-19.708057377844856

HEX7,0.0,1.6898670747950353

Htex,-1.8376463999999728,-3.8376463999985155

ICDHyr,1.2398589150000043,1.3571684511437232

IGPS,0.0113686,1.0113686

MDH,1.233197915000013,1.3505520511436848

NADH16pp,7.319156560000027,9.788394704567859

NH4tex,2.160164800000022,4.160164799998336

NH4tpp,2.160164800000022,4.160164799998336

O2tex,4.206049537500015,5.499323377855782

O2tpp,4.206049537500015,5.499323377855782

PDH,1.8733075150000043,1.9906170511371157

PFK_3,0.19573216416666564,1.5023475184447717

PGK,-3.564233014999997,-5.681542551134877

PGL,0.7056018925000015,1.1469471244256932

PGM,-3.2205985149999905,-5.3379080511348915

PPK,-0.7007176000000049,-1.7007175999971975

PRAIi,0.011368599999999998,1.0113686

PRPPS,0.1865784000000047,1.18557839999999

PSCVT,0.07622380000000008,1.076223799999787

RPE,0.31519592833333127,-0.3905739170491546

RPI,-0.38244516416667035,-1.5295602414753062

SHK3Dr,0.07622380000000008,1.076223799999787

SHKK,0.07622380000000009,1.076223799999787

SUCDi,1.024954315000004,1.1422638511437049

SUCOAS,-0.9198485150000041,-1.0371580511437049

TALA,0.0,-1.1595002769694553

TKT1,0.1957321641666656,0.34284724147531614

TKT2,0.11946376416666554,-0.7334211585244708

TPI,1.7028532254166657,2.687950454829674

TRPAS2,-0.0113686,-1.0113686

TRPS3,0.0113686,1.0113686

TRPt2rpp,0.0,-1.0

TRPtex,0.0,-1.0

XYLI2,0.0,1.6898670747950353

63 reacctions require a flux change for the evolution of the trp__L producer

.......................................................

.......................................................

uri

.......................................................

ACONTa,1.2398589150000043,1.3021015500143147

ACONTb,1.2398589150000041,1.3021015500143147

ADK1,0.524992600000009,1.5232460023819598

ASPCT,0.06615899999999995,1.066159

ASPTA,-0.5855358000000094,-1.586440199997227

ATPS4rpp,13.370320970000053,16.247157085409814

CBMKr,0.12531739999999997,1.1253174000000001

CO2tex,-4.632830737500008,-4.3264079298290925

CO2tpp,-4.632830737500008,-4.3264079298290925

CS,1.2398589150000043,1.3021015500143147

CYTBO3_4pp,8.410403675000031,10.797625276607693

DHAPT,0.32252132541666795,0.0

DHORD2,0.06615899999999995,1.066159

DHORTS,-0.06615899999999995,-1.066159

ENO,3.220598515000006,4.282405704230947

F6PA,0.32252132541666795,0.0

FBA,1.2128609358333324,2.146937830866795

FUM,1.2333893150000121,1.296848950016256

G6PDH2r,0.7056018925000015,1.2123818797933492

GAPD,3.5642330149999975,4.625424349998994

GLCptspp,2.1404179895833364,2.5893017392513356

GLCt2pp,0.0,0.9985366157110794

GLCtex_copy1,2.1404179895833364,3.588838354962415

GLUDy,-1.7103415000000157,-2.711365399989689

GND,0.7056018925000014,1.213381879793349

H2Otex,-9.914783537500057,-15.606967129818052

H2Otpp,-9.914783537500059,-15.607467129817381

HEX1,0.0,0.9975366157110794

Htex,-1.8376463999999728,-3.835904183085797

ICL,0.0,0.06324263501431027

MALS,0.0001338000000000684,0.06337643501431026

MDH,1.233197915000013,1.3608985850305664

NADH16pp,7.319156560000027,8.637824526591416

NH4tex,2.160164800000022,4.159839399992392

NH4tpp,2.160164800000022,4.159839399992392

NTD2,0.0,1.0

O2tex,4.206049537500015,5.3999103383038465

O2tpp,4.206049537500015,5.399660338303846

OMPDC,0.06615899999999997,1.066159

ORPT,-0.06615899999999995,-1.066159

PDH,1.8733075150000043,1.9987915850204954

PFK,1.2128609358333324,2.146937830866795

PGI,1.434816097083335,2.3744564751690658

PGK,-3.564233014999997,-4.625424349998994

PGL,0.7056018925000015,1.2123818797933492

PGM,-3.2205985149999905,-4.282405704230947

PPC,0.5918716000000012,1.5283163649809755

PPK,-0.7007176000000049,-1.6998971566110552

PRPPS,0.1865784000000047,1.1855297999999896

RPE,0.31519592833333127,-0.012888880136063506

RPI,-0.38244516416667035,-1.218309959929871

SUCDi,1.024954315000004,1.0895079500162763

TALA,0.0,-0.16504240423480385

TKT1,0.1957321641666656,0.031689759931861794

TKT2,0.11946376416666554,-0.0445786400679253

TPI,1.7028532254166657,2.3154103950334606

URIt2pp_copy2,0.0,-1.0

URItex,0.0,-1.0

58 reacctions require a flux change for the evolution of the uri producer

.......................................................

.......................................................

xan

.......................................................

ACONTa,1.2398589150000043,1.1898173217829098

ACONTb,1.2398589150000041,1.1898173217829098

ADSL2r,0.08960300000000408,1.089603000000004

AICART,0.10855060000000409,1.1085505999998235

AIRC2,0.08960300000000408,1.089603000000004

AIRC3,-0.08960300000000408,-1.089603000000004

AKGDH,1.024820515000004,0.9747789217834772

ASPTA,-0.5855358000000094,-1.5855357999998527

ATPS4rpp,13.370320970000053,21.470403356430012

CO2tex,-4.632830737500008,-4.257726754446408

CO2tpp,-4.632830737500008,-4.257726754446408

CS,1.2398589150000043,1.1898173217829098

CYTBO3_4pp,8.410403675000031,13.660195708907946

DHAPT,0.32252132541666795,3.2006595227506372e-06

ENO,3.220598515000006,3.17055692177229

F6PA,0.32252132541666795,3.2006595227506372e-06

FBA3,0.19573216416666564,1.1963926928751452

FUM,1.2333893150000121,2.1833477217833055

G6PDH2r,0.7056018925000015,0.9806226891068094

GAPD,3.5642330149999975,5.014191421772031

GARFT,0.08915700000000398,1.0891570000000041

GHMT2r,0.22309610000000604,1.723096099999916

GLCptspp,2.1404179895833364,2.412894521114674

GLCt2pp,0.0,0.4983394712879261

GLCtex_copy2,0.0,0.769816002819264

GLNS,0.36212300000000835,2.3621229999993694

GLUDy,-1.7103415000000157,-4.210341499998657

GLUPRT,0.08964760000000407,1.0896476000000042

GLYCL,0.010920900000001992,0.5109208999999117

GND,0.7056018925000014,0.9806226891068094

H2Otex,-9.914783537500057,-18.53967955443996

H2Otpp,-9.914783537500059,-18.53967955443972

HCO3E,0.10516900000000409,1.1051681999990874

HEX1,0.0,0.49733947128794975

HXAND,0.0,0.9990000000000236

Htex,-1.8376463999999728,-5.837646399998302

ICDHyr,1.2398589150000043,1.1898173217829098

IMPC,-0.10855060000000409,-1.1085505999998235

MDH,1.233197915000013,2.1832009217833055

MTHFC,0.1977522000000081,2.1977521999998277

MTHFD,0.1977522000000081,2.1977521999998277

NADH16pp,7.319156560000027,12.617123987124515

NH4tex,2.160164800000022,6.160164799998116

NH4tpp,2.160164800000022,6.160164799998116

NTD11,0.0,0.9990000000000236

O2tex,4.206049537500015,6.830945554453972

O2tpp,4.206049537500015,6.830945554453972

PDH,1.8733075150000043,1.8232659217736917

PFK_3,0.19573216416666564,1.1963926928751452

PGCD,0.34363450000000595,1.8436344999997412

PGI,1.434816097083335,1.9296113032958147

PGK,-3.564233014999997,-5.014191421772031

PGL,0.7056018925000015,0.9806226891068094

PGM,-3.2205985149999905,-3.17055692177229

PPK,-0.7007176000000049,-1.7008521999961181

PRAGSr,0.08964760000000409,1.0896476000000042

PRAIS,0.08964760000000409,1.0896476000000042

PRASCSi,0.0896030000000041,1.089603000000004

PRFGS,0.08964760000000407,1.0896476000000042

PSERT,0.343634500000006,1.8436344999997412

PSP_L,0.343634500000006,1.8436344999997412

PUNP5,0.0,0.9990000000000236

R15BPK,0.0,1.0001345999983717

R1PK,0.0,1.0001345999983717

RPE,0.31519592833333127,0.4985431260717002

RPI,-0.38244516416667035,-0.4741187630355672

SUCDi,1.024954315000004,0.9779127217834298

SUCOAS,-0.9198485150000041,-0.8698069217834526

TALA,0.0,-0.9089869298394015

TKT1,0.1957321641666656,0.2874057630357436

TKT2,0.11946376416666554,0.2111373630359566

TPI,1.7028532254166657,2.3819956293681246

XANtex,0.0,-1.0

XANtpp,0.0,-1.0

74 reacctions require a flux change for the evolution of the xan producer

.......................................................

.......................................................

xtsn

.......................................................

ACONTa,1.2398589150000043,1.2842731739503301

ACONTb,1.2398589150000041,1.2842731739503301

ADK1,0.524992600000009,1.5229925999987153

ADSL2r,0.08960300000000408,1.0896030000000063

AICART,0.10855060000000409,1.1085506000000063

AIRC2,0.08960300000000408,1.0896030000000063

AIRC3,-0.08960300000000408,-1.0896030000000063

AKGDH,1.024820515000004,1.0692347739513592

ASPTA,-0.5855358000000094,-1.5855357999987851

ATPS4rpp,13.370320970000053,22.281492452096103

CO2tex,-4.632830737500008,-4.4938663848629465

CO2tpp,-4.632830737500008,-4.4938663848629465

CS,1.2398589150000043,1.2842731739503301

CYTBO3_4pp,8.410403675000031,14.13247496974232

DHAPT,0.32252132541666795,1.1012522005415804e-09

ENO,3.220598515000006,3.265012773934186

F6PA,0.32252132541666795,1.1012522005415804e-09

FBA,1.2128609358333324,2.4352905778622294

FUM,1.2333893150000121,2.2778035739513722

G6PDH2r,0.7056018925000015,0.9333947630196795

GAPD,3.5642330149999975,5.108647273934006

GARFT,0.08915700000000398,1.0891570000000066

GHMT2r,0.22309610000000604,1.7230961000000098

GLCptspp,2.1404179895833364,2.5073535728365552

GLCt2pp,0.0,1.2765703579650274

GLCtex_copy1,2.1404179895833364,3.7839239308015826

GLNS,0.36212300000000835,2.362122999998594

GLUDy,-1.7103415000000157,-4.210341499994693

GLUPRT,0.08964760000000407,1.0896476000000064

GLYCL,0.010920900000001992,0.5109209000000035

GND,0.7056018925000014,0.9333947630196795

H2Otex,-9.914783537500057,-19.77581918484698

H2Otpp,-9.914783537500059,-19.77581918484698

HCO3E,0.10516900000000409,1.1051689999990897

HEX1,0.0,1.2755703579650515

Htex,-1.8376463999999728,-5.837646399993364

ICDHyr,1.2398589150000043,1.2842731739503301

IMPC,-0.10855060000000409,-1.1085506000000063

IMPD,0.048743200000000084,1.0477432

MDH,1.233197915000013,2.277612173951372

MTHFC,0.1977522000000081,2.197752200000013

MTHFD,0.1977522000000081,2.197752200000013

NADH16pp,7.319156560000027,12.996813595790961

NH4tex,2.160164800000022,6.160164799993489

NH4tpp,2.160164800000022,6.160164799993489

NTD10,0.0,0.999

O2tex,4.206049537500015,7.06708518487116

O2tpp,4.206049537500015,7.06708518487116

PDH,1.8733075150000043,1.9177217739399088

PFK,1.2128609358333324,2.4352905778622294

PGCD,0.34363450000000595,1.8436344999998195

PGI,1.434816097083335,2.8495291677819274

PGK,-3.564233014999997,-5.108647273934006

PGL,0.7056018925000015,0.9333947630196795

PGM,-3.2205985149999905,-3.265012773934186

PPK,-0.7007176000000049,-1.7007175999966415

PRAGSr,0.08964760000000409,1.0896476000000064

PRAIS,0.08964760000000409,1.0896476000000064

PRASCSi,0.0896030000000041,1.0896030000000063

PRFGS,0.08964760000000407,1.0896476000000064

PRPPS,0.1865784000000047,1.1855784000000062

PSERT,0.343634500000006,1.8436344999998195

PSP_L,0.343634500000006,1.8436344999998195

RPE,0.31519592833333127,-0.19960882465317464

RPI,-0.38244516416667035,-1.1250427876733124

SUCDi,1.024954315000004,1.0693685739513592

SUCOAS,-0.9198485150000041,-0.9642627739518148

TALA,0.0,-0.25840237649335945

TKT1,0.1957321641666656,-0.06167021232669381

TKT2,0.11946376416666554,-0.13793861232648083

TPI,1.7028532254166657,2.6037615431303376

XTSNt2rpp,0.0,-1.0

XTSNtex,0.0,-1.0

73 reacctions require a flux change for the evolution of the xtsn producer

.......................................................

.......................................................

##############################

##############################

CONSUMERS

##############################

12ppd__S

.......................................................

12PPDStex,0.0,3.8708838910714394

12PPDStpp,0.0,3.8708838910714394

ACONTa,1.2398589150000043,1.0670469754304113

ACONTb,1.2398589150000041,1.0670469754304113

ADK1,0.524992600000009,1.516842637898859

AKGDH,1.024820515000004,0.2606608134500258

ATPS4rpp,13.370320970000053,18.76045794034244

CO2tex,-4.632830737500008,-3.405505516731364

CO2tpp,-4.632830737500008,-3.405505516731364

CS,1.2398589150000043,1.0670469754304113

CYTBO3_4pp,8.410403675000031,13.69781349872796

DHAPT,0.32252132541666795,0.0

ENO,3.220598515000006,-0.8244362295695467

F6PA,0.32252132541666795,0.0

FBA,1.2128609358333324,-0.17470790400026445

FBA3,0.19573216416666564,0.0

FBP,0.0,0.17470790400026445

FUM,1.2333893150000121,1.061729444974173

G6PDH2r,0.7056018925000015,0.0

GAPD,3.5642330149999975,-0.4818266283277742

GLCptspp,2.1404179895833364,0.0

GLCtex_copy1,2.1404179895833364,0.0

GND,0.7056018925000014,0.0

H2Otex,-9.914783537500057,-12.556895116262389

H2Otpp,-9.914783537500059,-12.55689528440009

ICDHyr,1.2398589150000043,0.475450368528767

ICL,0.0,0.5915966069016443

L_LACD2,0.0,3.8708838910714394

LCADi,0.0,3.8708838910714394

LCARS,0.0,-3.8708838910714394

MALS,0.0001338000000000684,0.5917304069016444

MDH,1.233197915000013,1.6531783286555852

MTHFC,0.1977522000000081,0.195876269911191

MTHFD,0.1977522000000081,0.195876269911191

NADH16pp,7.319156560000027,8.906379387304899

O2tex,4.206049537500015,6.84975456319969

O2tpp,4.206049537500015,6.84975456319969

PDH,1.8733075150000043,2.2902845525158284

PFK,1.2128609358333324,0.0

PFK_3,0.19573216416666564,0.0

PFL,0.0,0.0018067660861714185

PGCD,0.34363450000000595,0.3426096012417725

PGI,1.434816097083335,0.0

PGK,-3.564233014999997,0.4818266283277742

PGL,0.7056018925000015,0.0

PGM,-3.2205985149999905,0.8244362295695467

PPC,0.5918716000000012,0.0

PPS,0.0,0.9902238295681824

PSERT,0.343634500000006,0.3426096012417725

PSP_L,0.343634500000006,0.3426096012417725

RPE,0.31519592833333127,-0.1558103040007228

RPI,-0.38244516416667035,-0.14784950400118108

SUCDi,1.024954315000004,0.8553912203516227

SUCOAS,-0.9198485150000041,-0.155713522086952

TALA,0.0,-0.03977095200046786

THD2pp,0.0,2.178525584408383

TKT1,0.1957321641666656,-0.03977095200046786

TKT2,0.11946376416666554,-0.11603935200025493

TPI,1.7028532254166657,-0.20296806446086588

59 reacctions require a flux change for the evolution of the 12ppd__S consumer

.......................................................

.......................................................

4abut

.......................................................

ABTA,0.0,3.2516745295087284

ABUTt2pp,0.0,3.2506745295087285

ABUTtex,0.0,3.2516745295087284

ACONTa,1.2398589150000043,0.4459649961855903

ACONTb,1.2398589150000041,0.4459649961855903

AKGDH,1.024820515000004,0.23092659618558986

ATPS4rpp,13.370320970000053,17.792081570597034

CO2tex,-4.632830737500008,-4.797020918060003

CO2tpp,-4.632830737500008,-4.797020918060003

CS,1.2398589150000043,0.4459649961855903

CYTBO3_4pp,8.410403675000031,11.990458565638216

DHAPT,0.32252132541666795,0.0

ENO,3.220598515000006,-0.8249699333313667

F6PA,0.32252132541666795,-0.001

FBA,1.2128609358333324,-0.17310293346456657

FBA3,0.19573216416666564,1.3206857829572982e-10

FBP,0.0,0.17310293346456657

FUM,1.2333893150000121,3.6911699256937496

G6PDH2r,0.7056018925000015,0.0

GAPD,3.5642330149999975,-0.48140233333136767

GLCptspp,2.1404179895833364,0.0

GLCtex_copy1,2.1404179895833364,0.0

GLUDy,-1.7103415000000157,1.541333029510439

GND,0.7056018925000014,0.0

H2Otex,-9.914783537500057,-6.827299188545393

H2Otpp,-9.914783537500059,-6.82729918854488

Htex,-1.8376463999999728,1.4140281295113544

ICDHyr,1.2398589150000043,0.4459649961855903

MDH,1.233197915000013,2.0219777295142767

ME1,0.0,1.6689115961794727

NADH16pp,7.319156560000027,8.441430839943896

NADTRHD,0.0,1.0425768257039212

NH4tex,2.160164800000022,-1.0915097295112595

NH4tpp,2.160164800000022,-1.0915097295112595

O2tex,4.206049537500015,5.996076982819107

O2tpp,4.206049537500015,5.996076982819107

PDH,1.8733075150000043,1.079279796179174

PFK,1.2128609358333324,0.0

PFK_3,0.19573216416666564,1.3206857829572982e-10

PGI,1.434816097083335,0.0

PGK,-3.564233014999997,0.48140233333136767

PGL,0.7056018925000015,0.0

PGM,-3.2205985149999905,0.8249699333313667

PPC,0.5918716000000012,0.0

PPCK,0.0,0.9907575333300025

RPE,0.31519592833333127,-0.15520533333295633

RPI,-0.38244516416667035,-0.1472445333334147

SSALx,0.0,3.2516745295087284

SUCDi,1.024954315000004,3.4827349256943183

SUCOAS,-0.9198485150000041,-0.12595459618556532

TALA,0.0,-0.03946846679865319

TKT1,0.1957321641666656,-0.039468466666584615

TKT2,0.11946376416666554,-0.1157368666663717

TPI,1.7028532254166657,-0.20236413333249798

54 reacctions require a flux change for the evolution of the 4abut consumer

.......................................................

.......................................................

5dglcn

.......................................................

5DGLCNR,0.0,2.5685015875005774

5DGLCNt2rpp,0.0,2.5685015875005774

5DGLCNtex,0.0,2.5685015875005774

CO2tex,-4.632830737500008,-7.201332325057386

CO2tpp,-4.632830737500008,-7.201332325057386

FBA,1.2128609358333324,0.7867775779252388

GLCptspp,2.1404179895833364,0.0

GLCtex_copy1,2.1404179895833364,0.0

GND,0.7056018925000014,3.2741027599818198

GNK,0.0,2.5685015875005774

Htex,-1.8376463999999728,0.7308551875066589

PFK,1.2128609358333324,0.7867784781912448

PGI,1.434816097083335,-0.7056011724812421

PYK,0.0,2.1414182295985

RPE,0.31519592833333127,2.0275298399883956

RPI,-0.38244516416667035,-1.2386121199938822

TALA,0.0,0.8571669558274257

TKT1,0.1957321641666656,1.0518991199940915

TKT2,0.11946376416666554,0.9756307199943044

TPI,1.7028532254166657,1.2747698675087473

20 reacctions require a flux change for the evolution of the 5dglcn consumer

.......................................................

.......................................................

LalaDgluMdap

.......................................................

3PEPTabcpp,0.0,1.7770729672897247

3PEPTtex,0.0,1.7770729672897687

ACONTa,1.2398589150000043,0.8970361402922435

ACONTb,1.2398589150000041,0.8970361402922435

AKGDH,1.024820515000004,2.459070707581912

ALAGLUE,0.0,1.7715153672896804

ALATA_L,-0.11627119999999999,1.6608463672897642

ASAD,-0.21381520000000004,-0.13962500000002365

ASPK,0.2138152,0.13962500000002365

ASPT,0.0,2.0957036139176997

ASPTA,-0.5855358000000094,-2.6070484139177097

ATPS4rpp,13.370320970000053,17.18171107512137

CO2tex,-4.632830737500008,-8.22920105322271

CO2tpp,-4.632830737500008,-8.22920105322271

CS,1.2398589150000043,0.8970361402921873

CYTBO3_4pp,8.410403675000031,10.42036208266833

DAPDC,0.0686322,1.7715153672897248

DAPE,0.07418980000000001,0.0

DHAPT,0.32252132541666795,0.0

DHDPRy,0.07418980000000004,0.0

DHDPS,0.07418980000000004,0.0

ENO,3.220598515000006,-0.8247646638102424

F6PA,0.32252132541666795,0.0

FBA,1.2128609358333324,-0.17410213333334923

FBA3,0.19573216416666564,0.0

FBP,0.0,0.17410213333333

FUM,1.2333893150000121,4.7633423214996355

G6PDH2r,0.7056018925000015,0.0

GAPD,3.5642330149999975,-0.48138666381032635

GLCptspp,2.1404179895833364,0.0

GLCtex_copy1,2.1404179895833364,-0.0009999999999763531

GLUDy,-1.7103415000000157,-0.10421727933828606

GLUR,-0.005557600000000003,0.0

GND,0.7056018925000014,0.0

H2Otex,-9.914783537500057,-2.700313937277315

H2Otpp,-9.914783537500059,-2.700315179961308

Htex,-1.8376463999999728,5.344834383791067

ICDHyr,1.2398589150000043,0.8970361402922435

LADGMDH,0.0,1.7715153672897248

LALGP,0.0,1.7715153672897248

LYSt3pp,0.0,1.7028831672897249

LYStex,0.0,-1.7028831672897695

MDH,1.233197915000013,4.497311618020149

ME1,0.0,0.26583930347943474

NADH16pp,7.319156560000027,7.894864775086418

NH4tex,2.160164800000022,-1.542363534579522

NH4tpp,2.160164800000022,-1.542363534579522

O2tex,4.206049537500015,5.211028741334189

O2tpp,4.206049537500015,5.211028741334189

PDH,1.8733075150000043,1.5309741402921873

PFK,1.2128609358333324,0.0

PFK_3,0.19573216416666564,0.0

PGI,1.434816097083335,0.0

PGK,-3.564233014999997,0.48138666381032635

PGL,0.7056018925000015,0.0

PGM,-3.2205985149999905,0.8247646638102424

PPC,0.5918716000000012,0.0

PPCK,0.0,0.9915522638102897

RPE,0.31519592833333127,-0.15520453333328987

RPI,-0.38244516416667035,-0.14724373333331187

SDPDS,0.07418980000000003,0.0

SDPTA,-0.07418980000000003,0.0

SUCDi,1.024954315000004,2.4602045075819117

SUCOAS,-0.9198485150000041,-2.428288907581873

TALA,0.0,-0.0394680666667

THDPS,0.07418980000000003,0.0

TKT1,0.1957321641666656,-0.0394680666667

TKT2,0.11946376416666554,-0.11573646666670356

TPI,1.7028532254166657,-0.20236333333332368

UAAGDS,0.005557600000000004,0.0

UAMAGS,0.005557600000000003,0.0

UAMAS,0.005557600000000003,0.0

UM3PL,0.0,0.0055576

73 reacctions require a flux change for the evolution of the LalaDgluMdap consumer

.......................................................

.......................................................

LalaDgluMdapDala

.......................................................

4PCP,0.0,1.2656127640864505

4PEPTabcpp,0.0,1.2656127640864505

4PEPTtex,0.0,1.2656127640864505

ACONTa,1.2398589150000043,1.1411563475962687

ACONTb,1.2398589150000041,1.1411563475962687

ADK1,0.524992600000009,0.7658858943800118

AKGDH,1.024820515000004,2.192041711684681

ALAGLUE,0.0,1.2646127640864506

ALAR,0.008336400000000004,-1.2572763640864506

ALATA_L,-0.11627119999999999,2.4159545716695328

ASAD,-0.21381520000000004,-0.13962579999724767

ASPK,0.2138152,0.13962579999724767

ASPT,0.0,2.5836240854668233

ASPTA,-0.5855358000000094,-3.094872539084555

ATPS4rpp,13.370320970000053,17.188631007661147

CO2tex,-4.632830737500008,-7.426879500990209

CO2tpp,-4.632830737500008,-7.426879500990776

CS,1.2398589150000043,1.1411563475962687

CYTBO3_4pp,8.410403675000031,10.348101669822093

DAPDC,0.0686322,1.2600551640864506

DAPE,0.07418980000000001,0.0

DHAPT,0.32252132541666795,0.0

DHDPRy,0.07418980000000004,0.0

DHDPS,0.07418980000000004,0.0

ENO,3.220598515000006,-0.8237581881734506

F6PA,0.32252132541666795,0.0

FBA,1.2128609358333324,-0.17404041787146826

FBA3,0.19573216416666564,0.0

FBP,0.0,0.17404041787146826

FUM,1.2333893150000121,4.984138650771988

G6PDH2r,0.7056018925000015,0.0

GAPD,3.5642330149999975,-0.4811234446787933

GLCptspp,2.1404179895833364,0.0

GLCtex_copy1,2.1404179895833364,0.0

GLUDy,-1.7103415000000157,-0.34738494681849585

GND,0.7056018925000014,0.0

H2Otex,-9.914783537500057,-2.432645310012682

H2Otpp,-9.914783537500059,-2.4326453100118366

Htex,-1.8376463999999728,4.566354356356784

ICDHyr,1.2398589150000043,1.1411563475962687

LADGMDH,0.0,1.2646127640864506

LALGP,0.0,1.2646127640864506

LYSt3pp,0.0,1.1914229640864507

LYStex,0.0,-1.1904229640864508

MDH,1.233197915000013,4.983945650771989

MTHFC,0.1977522000000081,0.19588977319137682

MTHFD,0.1977522000000081,0.19588977319137682

NADH16pp,7.319156560000027,8.089633358137412

NH4tex,2.160164800000022,-1.785383358215506

NH4tpp,2.160164800000022,-1.785383358215506

O2tex,4.206049537500015,5.174898534911046

O2tpp,4.206049537500015,5.174898534911046

PDH,1.8733075150000043,1.7729582671570643

PFK,1.2128609358333324,0.0

PFK_3,0.19573216416666564,0.0

PFL,0.0,0.0017664804296739917

PGCD,0.34363450000000595,0.3426347434946573

PGI,1.434816097083335,0.0

PGK,-3.564233014999997,0.4811234446787933

PGL,0.7056018925000015,0.0

PGM,-3.2205985149999905,0.8237581881734506

PPC,0.5918716000000012,0.0

PPCK,0.0,0.7472435640911648

PPS,0.0,0.24230222408092145

PSERT,0.343634500000006,0.3426347434946573

PSP_L,0.343634500000006,0.3426347434946573

RPE,0.31519592833333127,-0.1551428178719266

RPI,-0.38244516416667035,-0.14718201787238494

SDPDS,0.07418980000000003,0.0

SDPTA,-0.07418980000000003,0.0

SUCDi,1.024954315000004,2.192175511684681

SUCOAS,-0.9198485150000041,-2.1602591116866616

TALA,0.0,-0.03943720893606976

THDPS,0.07418980000000003,0.0

TKT1,0.1957321641666656,-0.03943720893606976

TKT2,0.11946376416666554,-0.11570560893585684

TPI,1.7028532254166657,-0.20230001787146823

77 reacctions require a flux change for the evolution of the LalaDgluMdapDala consumer

.......................................................

.......................................................

ac

.......................................................

ACKr,0.11651519999999993,8.303444187687546

ACONTa,1.2398589150000043,5.384154560827686

ACONTb,1.2398589150000041,5.384154560827686

ACt2rpp,0.0,8.186883057334512

ACtex,0.0,8.186883057334512

AKGDH,1.024820515000004,3.5856559339573537

ASPT,0.0,0.00665796682184867

ATPS4rpp,13.370320970000053,22.62869904533511

CO2tex,-4.632830737500008,-8.167151935572543

CO2tpp,-4.632830737500008,-8.166151935572543

CS,1.2398589150000043,5.38415456082764

CYTBO3_4pp,8.410403675000031,15.479106539314403

DHAPT,0.32252132541666795,0.0

ENO,3.220598515000006,-0.8234250268756114

F6PA,0.32252132541666795,0.0

FBA,1.2128609358333324,-0.17470719860760064

FBA3,0.19573216416666564,0.0

FBP,0.0,0.17470719860760062

FLDR2,0.0,1.1355967519768992

FUM,1.2333893150000121,5.385557923482338

G6PDH2r,0.7056018925000015,0.0

GAPD,3.5642330149999975,-0.481790396519159

GLCptspp,2.1404179895833364,0.0

GLCtex_copy1,2.1404179895833364,0.0

GND,0.7056018925000014,0.0

H2Otex,-9.914783537500057,-13.447201510536502

H2Otpp,-9.914783537500059,-13.447201510535933

Htex,-1.8376463999999728,6.349982819943989

ICDHyr,1.2398589150000043,3.8003833339554376

ICL,0.0,1.5837712268722486

MALS,0.0001338000000000684,1.5819050268722485

MDH,1.233197915000013,6.965682150354586

MTHFC,0.1977522000000081,0.19588960208736006

MTHFD,0.1977522000000081,0.19588960208736006

NADH16pp,7.319156560000027,10.236824615832074

O2tex,4.206049537500015,7.740400969657173

O2tpp,4.206049537500015,7.740400969657173

PDH,1.8733075150000043,0.5464404885959232

PFK,1.2128609358333324,0.0

PFK_3,0.19573216416666564,0.0

PFL,0.0,0.0017673937397210127

PGCD,0.34363450000000595,0.34263463035645253

PGI,1.434816097083335,0.0

PGK,-3.564233014999997,0.481790396519159

PGL,0.7056018925000015,0.0

PGM,-3.2205985149999905,0.8244250268756114

POR5,0.021750999999999996,-1.1149667519769082

PPC,0.5918716000000012,0.0

PPCK,0.0,0.9892126268742125

PSERT,0.343634500000006,0.34263463035645253

PSP_L,0.343634500000006,0.34263463035645253

PTAr,-0.11651519999999993,-8.303444187687546

RPE,0.31519592833333127,-0.15580959860801613

RPI,-0.38244516416667035,-0.14784879860849287

SUCDi,1.024954315000004,5.1771229234823295

SUCOAS,-0.9198485150000041,-3.4806835339593363

TALA,0.0,-0.03977059930411997

TKT1,0.1957321641666656,-0.03977059930411997

TKT2,0.11946376416666554,-0.11603899930389616

TPI,1.7028532254166657,-0.20296679860760058

60 reacctions require a flux change for the evolution of the ac consumer

.......................................................

.......................................................

acald

.......................................................

ACALD,0.0,5.787929770175651

ACALDtex,0.0,5.787929770175651

ACALDtpp,0.0,5.787929770175651

ACONTa,1.2398589150000043,2.9821533368622566

ACONTb,1.2398589150000041,2.9821533368622566

AKGDH,1.024820515000004,1.1844189035376078

ATPS4rpp,13.370320970000053,16.838589263373926

CO2tex,-4.632830737500008,-3.3661823404036255

CO2tpp,-4.632830737500008,-3.3661823404036255

CS,1.2398589150000043,2.9821533368622566

CYTBO3_4pp,8.410403675000031,11.66503665099717

DHAPT,0.32252132541666795,0.0

ENO,3.220598515000006,-0.8250368333285134

F6PA,0.32252132541666795,0.0

FBA,1.2128609358333324,-0.17410293360046541

FBA3,0.19573216416666564,2.6911450845545914e-10

FBP,0.0,0.17410293360046541

FLDR2,0.0,0.5876317999979334

FUM,1.2333893150000121,2.9756837368625386

G6PDH2r,0.7056018925000015,0.0

GAPD,3.5642330149999975,-0.48140233332889604

GLCptspp,2.1404179895833364,0.0

GLCtex_copy1,2.1404179895833364,0.0

GND,0.7056018925000014,0.0

H2Otex,-9.914783537500057,-8.648635140381167

H2Otpp,-9.914783537500059,-8.648135140384822

ICDHyr,1.2398589150000043,1.3994573035365787

ICL,0.0,1.5826960333256779

MALS,0.0001338000000000684,1.5828298333256778

MDH,1.233197915000013,4.558188370188216

NADH16pp,7.319156560000027,8.82949511413409

O2tex,4.206049537500015,5.833616025496757

O2tpp,4.206049537500015,5.833366025498584

PDH,1.8733075150000043,0.0

PFK,1.2128609358333324,0.0

PFK_3,0.19573216416666564,2.6911450845545914e-10

PGI,1.434816097083335,0.0

PGK,-3.564233014999997,0.48140233332889604

PGL,0.7056018925000015,0.0

PGM,-3.2205985149999905,0.8250368333285134

POR5,0.021750999999999996,-0.5678807999981393

PPC,0.5918716000000012,0.0

PPCK,0.0,0.990824433327575

RPE,0.31519592833333127,-0.15520533333276948

RPI,-0.38244516416667035,-0.14724453333322787

SUCDi,1.024954315000004,2.7692487368632857

SUCOAS,-0.9198485150000041,-1.0794469035380634

TALA,0.0,-0.03946846693549925

THD2pp,0.0,1.8412371964520469

TKT1,0.1957321641666656,-0.03946846666638474

TKT2,0.11946376416666554,-0.11573686666638475

TPI,1.7028532254166657,-0.2023641333311604

52 reacctions require a flux change for the evolution of the acald consumer

.......................................................

.......................................................

ade

.......................................................

ACALD,0.0,0.33093548665422495

ACONTa,1.2398589150000043,0.21503840000001812

ACONTb,1.2398589150000041,0.21503840000001812

ADD,0.0,5.2063726934645524

ADEt2rpp,0.0,5.266180093464551

ADEtex,0.0,5.266180093464551

ADPT,0.0,0.05994199999999861

ADSL1r,0.059807400000004014,0.0

ADSL2r,0.08960300000000408,0.0

ADSS,0.059807400000004014,0.0

AICART,0.10855060000000409,0.018947600000000002

AIRC2,0.08960300000000408,0.0

AIRC3,-0.08960300000000408,0.0

AKGDH,1.024820515000004,0.0

ALLTAMH,0.0,5.1765770934645525

ALLTN,0.0,5.1765770934645525

ASAD,-0.21381520000000004,-0.5457506866536045

ASPK,0.2138152,0.5457506866536045

ASPTA,-0.5855358000000094,-0.7680608866530374

ATPS4rpp,13.370320970000053,18.84433141313907

CO2tex,-4.632830737500008,-18.121223267345815

CO2tpp,-4.632830737500008,-18.121223267345815

CS,1.2398589150000043,0.21503840000001812

CYTBO3_4pp,8.410403675000031,9.056288267380918

DHAPT,0.32252132541666795,0.0

ENO,3.220598515000006,2.1069531134009107

F6PA,0.32252132541666795,0.0

FBA,1.2128609358333324,-0.17410293333249735

FBA3,0.19573216416666564,0.0

FBP,0.0,0.17410293333249735

FTHFLi,0.0,0.26317428652787

FUM,1.2333893150000121,0.05915839999943273

G6PDH2r,0.7056018925000015,0.0

GAPD,3.5642330149999975,-0.4814023333313653

GARFT,0.08915700000000398,-0.0014460000000000835

GHMT2r,0.22309610000000604,-0.20936328659084028

GLCptspp,2.1404179895833364,0.0

GLCtex_copy1,2.1404179895833364,0.0

GLNS,0.36212300000000835,0.18291699999954172

GLUDy,-1.7103415000000157,-1.5492320866524456

GLUPRT,0.08964760000000407,4.46e-05

GLXCL,0.0,2.588355446732276

GLYCK,0.0,2.588355446732276

GLYCL,0.010920900000001992,6.297037891123125e-11

GND,0.7056018925000014,0.0

H2Otex,-9.914783537500057,29.258624867293037

H2Otpp,-9.914783537500059,29.25862486729326

HCO3E,0.10516900000000409,0.015565999999083324

HSDy,-0.13962539999999996,-0.4715608866536045

HSK,0.10884319999999999,0.4407786866536045

HXAND,0.0,5.177577093464553

HXPRT,0.0,0.028795600000000005

Htex,-1.8376463999999728,24.49325406732365

ICDHyr,1.2398589150000043,0.21503840000001812

IMPC,-0.10855060000000409,-0.018947600000000002

MDH,1.233197915000013,5.2354102934639855

MOX,0.00032519999999967104,-5.176251893464553

MTHFC,0.1977522000000081,-0.24562808652787008

MTHFD,0.1977522000000081,-0.24562808652787008

NADH16pp,7.319156560000027,8.98786166738094

NDPK1,0.158125000000004,0.0993176000000002

NH4tex,2.160164800000022,-24.17073566732381

NH4tpp,2.160164800000022,-24.17073566732381

O2tex,4.206049537500015,4.52899183369046

O2tpp,4.206049537500015,4.52899183369046

PDH,1.8733075150000043,0.25224342681131556

PFK,1.2128609358333324,0.0

PFK_3,0.19573216416666564,0.0

PFL,0.0,0.2641742865278701

PGCD,0.34363450000000595,0.0

PGI,1.434816097083335,0.0

PGK,-3.564233014999997,0.4814023333313653

PGL,0.7056018925000015,0.0

PGM,-3.2205985149999905,-2.1069531134009107

PPC,0.5918716000000012,0.9239408866536227

PRAGSr,0.08964760000000409,4.46e-05

PRAIS,0.08964760000000409,4.46e-05

PRASCSi,0.0896030000000041,0.0

PRFGS,0.08964760000000407,4.46e-05

PSERT,0.343634500000006,0.0

PSP_L,0.343634500000006,0.0

PYK,0.0,1.0172246267486527

RPE,0.31519592833333127,-0.1552053333329557

RPI,-0.38244516416667035,-0.14724453333341406

SERD_L,0.0,0.08682488659084006

SUCDi,1.024954315000004,0.002133799999977315

SUCOAS,-0.9198485150000041,0.10497199999943273

TALA,0.0,-0.039468466666584295

THD2pp,0.0,3.3821661464849706

THRA,0.0,0.3309354866538107

THRS,0.1088432,0.4407786866536045

TKT1,0.1957321641666656,-0.039468466666584295

TKT2,0.11946376416666554,-0.1157368666663714

TPI,1.7028532254166657,-0.20236413333249736

TRSARr,0.0,2.587355446732276

UGLYCH,0.0,5.1765770934645525

URIC,0.0,5.1765770934645525

XAND,0.0,5.1765770934645525

98 reacctions require a flux change for the evolution of the ade consumer

.......................................................

.......................................................

adn

.......................................................

ACONTa,1.2398589150000043,0.21503839999945074

ACONTb,1.2398589150000041,0.21503839999945074

ADD,0.0,1.5628322562050003

ADNt2pp_copy1,0.0,1.6216396562048203

ADNtex,0.0,1.6226396562048202

ADPT,0.0,0.05994199999981986

ADSL1r,0.059807400000004014,0.0

ADSL2r,0.08960300000000408,0.0

ADSS,0.059807400000004014,0.0

AICART,0.10855060000000409,0.01894759999981943

AIRC2,0.08960300000000408,0.0

AIRC3,-0.08960300000000408,0.0

AKGDH,1.024820515000004,0.0

ALLTAMH,0.0,1.5330366562048199

ALLTN,0.0,1.5330366562048199

ASPTA,-0.5855358000000094,-0.43612539999922817

CO2tex,-4.632830737500008,-8.016719362083164

CO2tpp,-4.632830737500008,-7.948958161957663

CS,1.2398589150000043,0.21503839999945074

CYTBO3_4pp,8.410403675000031,7.064982643158792

ENO,3.220598515000006,2.1957779999875786

FBA,1.2128609358333324,0.004647547898917504

FDH4pp,0.0,0.06676120012550096

FORtppi,0.0,0.06776120012550096

FTHFD,0.0,0.06727060012550103

FUM,1.2333893150000121,0.0591584

G6PDH2r,0.7056018925000015,1.1482673999658493

GAPD,3.5642330149999975,1.8402412936885384

GARFT,0.08915700000000398,4.46e-05

GHMT2r,0.22309610000000604,0.12257220006266029

GLCptspp,2.1404179895833364,0.0

GLCtex_copy1,2.1404179895833364,0.0

GLNS,0.36212300000000835,0.18291699999936106

GLUDy,-1.7103415000000157,-1.4604072000605495

GLUPRT,0.08964760000000407,4.46e-05

GLXCL,0.0,0.5986473063615103

GLYCK,0.0,0.5986473063615103

GLYCL,0.010920900000001992,6.266026586487783e-11

GND,0.7056018925000014,1.1482673999658493

H2Otex,-9.914783537500057,4.550364056176858

H2Otpp,-9.914783537500059,4.550364056177098

HCO3E,0.10516900000000409,0.015565999999083336

HXAND,0.0,1.5330366562048199

HXPRT,0.0,0.02979560000018054

Htex,-1.8376463999999728,6.27555188102626

ICDHyr,1.2398589150000043,0.21503839999945074

IMPC,-0.10855060000000409,-0.01894759999981943

MALS,0.0001338000000000684,0.3358758434817992

MDH,1.233197915000013,1.9277456996866191

MOX,0.00032519999999967104,-1.5327114562048199

MTHFC,0.1977522000000081,0.08630740012532045

MTHFD,0.1977522000000081,0.08630740012532045

NADH16pp,7.319156560000027,6.930794843033291

NDPK1,0.158125000000004,0.09931760000000006

NH4tex,2.160164800000022,-5.953033481026823

NH4tpp,2.160164800000022,-5.953033481026823

O2tex,4.206049537500015,3.533339021579396

O2tpp,4.206049537500015,3.533339021579396

PDH,1.8733075150000043,1.184229043471789

PFK,1.2128609358333324,0.004647547898917504

PGCD,0.34363450000000595,0.2431106000624701

PGI,1.434816097083335,-1.1482673999658493

PGK,-3.564233014999997,-1.8402412936885384

PGL,0.7056018925000015,1.1482673999658493

PGM,-3.2205985149999905,-2.1957779999875786

PPC,0.5918716000000012,0.2561295565168797

PPM,-0.00013460000000000268,1.6226396562048202

PRAGSr,0.08964760000000409,4.46e-05

PRAIS,0.08964760000000409,4.46e-05

PRASCSi,0.0896030000000041,0.0

PRFGS,0.08964760000000407,4.46e-05

PSERT,0.343634500000006,0.2431106000624701

PSP_L,0.343634500000006,0.2431106000624701

PUNP1,-0.00013460000000000268,1.6226396562048202

PYK,0.0,1.450339518055395

RPE,0.31519592833333127,1.6920660374476095

RPI,-0.38244516416667035,0.5517594374813021

SUCDi,1.024954315000004,0.0012675999999999937

SUCOAS,-0.9198485150000041,0.104972

TALA,0.0,0.6874350545570652

TKT1,0.1957321641666656,0.8841672187236983

TKT2,0.11946376416666554,0.8078988187239112

TPI,1.7028532254166657,0.4966398374824089

TRSARr,0.0,0.5976473063615103

UGLYCH,0.0,1.5330366562048199

URIC,0.0,1.5330366562048199

XAND,0.0,1.5330366562048199

87 reacctions require a flux change for the evolution of the adn consumer

.......................................................

.......................................................

agm

.......................................................

ABTA,0.0,2.70925911646341

ABUTD,0.0,2.70925911646341

ACALD,0.0,0.308315713190718

ACONTa,1.2398589150000043,0.21468160000000092

ACONTb,1.2398589150000041,0.21468160000000092

AGMT,0.0,2.7093049164614293

AGMtex,0.0,2.7093049164614293

AKGDH,1.024820515000004,0.0

ARGAGMt7pp,0.0,-2.7093049164614293

ARGORNt7pp,0.0,2.7093049164614293

ASAD,-0.21381520000000004,-0.5241313131885313

ASPK,0.2138152,0.5241313131885313

ASPT,0.0,5.415886080658721

ASPTA,-0.5855358000000094,-6.311776205890297

ATPS4rpp,13.370320970000053,18.64540298700773

CO2tex,-4.632830737500008,-2.6306358546355475

CO2tpp,-4.632830737500008,-2.6306358546355475

CS,1.2398589150000043,0.21468160000000092

CYTBO3_4pp,8.410403675000031,12.533965910882575

DHAPT,0.32252132541666795,0.0

ENO,3.220598515000006,-0.5153218548284593

F6PA,0.32252132541666795,-0.001

FBA,1.2128609358333324,-0.1740385074150091

FBA3,0.19573216416666564,0.0

FBP,0.0,0.1740385074150091

FTHFLi,0.0,0.3102261131887375

FUM,1.2333893150000121,8.334754209165176

G6PDH2r,0.7056018925000015,0.001

GAPD,3.5642330149999975,-0.48211866853775187

GHMT2r,0.22309610000000604,-0.08727045206507605

GLCptspp,2.1404179895833364,0.0

GLCtex_copy1,2.1404179895833364,0.0

GND,0.7056018925000014,0.0

H2Otex,-9.914783537500057,-2.4920391552112533

H2Otpp,-9.914783537500059,-2.4920391552112533

HSDy,-0.13962539999999996,-0.4499415131885314

HSK,0.10884319999999999,0.4191589131905118

ICDHyr,1.2398589150000043,0.21468160000000092

MDH,1.233197915000013,7.209242060718299

ME1,0.0,0.4674876816123157

ME2,0.0,0.658699266834561

MTHFC,0.1977522000000081,-0.11352475206309359

MTHFD,0.1977522000000081,-0.11352475206309359

NADH16pp,7.319156560000027,9.755280194419166

NH4tex,2.160164800000022,-3.2587896273012644

NH4tpp,2.160164800000022,-3.2587896273012644

O2tex,4.206049537500015,6.267830655441287

O2tpp,4.206049537500015,6.267830655441287

PDH,1.8733075150000043,0.22962307413270916

PFK,1.2128609358333324,0.0

PFK_3,0.19573216416666564,0.0

PFL,0.0,0.3101752743143735

PGCD,0.34363450000000595,0.033270086290707476

PGI,1.434816097083335,-0.001

PGK,-3.564233014999997,0.48211866853775187

PGL,0.7056018925000015,0.001

PGM,-3.2205985149999905,0.5153218548284593

PPC,0.5918716000000012,0.0

PPCK,0.0,0.6811094548280011

PSERT,0.343634500000006,0.033270086290707476

PSP_L,0.343634500000006,0.033270086290707476

PTRCORNt7pp,0.0,-2.7093049164614293

PTRCTA,0.0,2.70925911646341

PTRCt2pp,0.0,2.7093049164614293

RPE,0.31519592833333127,-0.15514090741546743

RPI,-0.38244516416667035,-0.14718010741592577

SSALy,0.0,2.70925911646341

SUCDi,1.024954315000004,2.71339291646341

SUCOAS,-0.9198485150000041,0.10511428978978452

TALA,0.0,-0.0394362537077337

THRA,0.0,0.308315713190718

THRS,0.1088432,0.4191589131905118

TKT1,0.1957321641666656,-0.0394362537077337

TKT2,0.11946376416666554,-0.11570465370773374

TPI,1.7028532254166657,-0.20329810741500903

UREAtex,0.0,-2.7093049164614293

UREAtpp,0.0,-2.7093049164614293

77 reacctions require a flux change for the evolution of the agm consumer

.......................................................

.......................................................

akg

.......................................................

ACONTa,1.2398589150000043,0.5662113392643819

ACONTb,1.2398589150000041,0.5662113392643819

AKGDH,1.024820515000004,3.7230970128108676

AKGt2rpp,0.0,3.3719240735454385

AKGtex,0.0,3.3719240735454385

ATPS4rpp,13.370320970000053,14.299916755762627

CO2tex,-4.632830737500008,-8.649943167775556

CO2tpp,-4.632830737500008,-8.649943167775556

CS,1.2398589150000043,0.5662113392643819

CYTBO3_4pp,8.410403675000031,9.700780388469495

DHAPT,0.32252132541666795,0.0

ENO,3.220598515000006,-0.8249731342942236

F6PA,0.32252132541666795,0.0

FBA,1.2128609358333324,-0.174106134296586

FBA3,0.19573216416666564,0.0

FBP,0.0,0.174106134296586

FUM,1.2333893150000121,3.931665812810119

G6PDH2r,0.7056018925000015,9.602895505861353e-06

GAPD,3.5642330149999975,-0.4814055342943149

GLCptspp,2.1404179895833364,0.0

GLCtex_copy1,2.1404179895833364,0.0

GND,0.7056018925000014,9.602895505861353e-06

H2Otex,-9.914783537500057,-7.188047820666339

H2Otpp,-9.914783537500059,-7.188047820666339

Htex,-1.8376463999999728,4.906201747099219

ICDHyr,1.2398589150000043,0.5662113392643819

MDH,1.233197915000013,2.1421826735552405

ME2,0.0,1.7891579392548786

NADH16pp,7.319156560000027,5.911256775658625

O2tex,4.206049537500015,4.851237894234747

O2tpp,4.206049537500015,4.851237894234747

PDH,1.8733075150000043,1.1995261392570051

PFK,1.2128609358333324,0.0

PFK_3,0.19573216416666564,0.0

PGI,1.434816097083335,-9.602895505861353e-06

PGK,-3.564233014999997,0.4814055342943149

PGL,0.7056018925000015,9.602895505861353e-06

PGM,-3.2205985149999905,0.8249731342942236

PPC,0.5918716000000012,0.0

PPCK,0.0,0.9907607342928594

RPE,0.31519592833333127,-0.15519893140249874

RPI,-0.38244516416667035,-0.14724773429846297

SUCDi,1.024954315000004,3.7232308128108675

SUCOAS,-0.9198485150000041,-3.6181250128113476

TALA,0.0,-0.03946526570135582

THD2pp,0.0,0.295674215678796

TKT1,0.1957321641666656,-0.03946526570135582

TKT2,0.11946376416666554,-0.11573366570114291

TPI,1.7028532254166657,-0.202367334296586

49 reacctions require a flux change for the evolution of the akg consumer

.......................................................

.......................................................

ala__D

.......................................................

ACONTa,1.2398589150000043,1.9649576366878927

ACONTb,1.2398589150000041,1.9649576366878927

ADK1,0.524992600000009,1.3706717326424231

ALAR,0.008336400000000004,-4.749478531015408

ALATA_L,-0.11627119999999999,4.640543731015889

ATPS4rpp,13.370320970000053,17.85578432327991

CO2tex,-4.632830737500008,-6.069769993076908

CO2tpp,-4.632830737500008,-6.069769993076908

CS,1.2398589150000043,1.9649576366878927

CYTBO3_4pp,8.410403675000031,11.284282186168289

DALAt2pp,0.0027788000000000005,4.762594531007763

DALAtex,0.0,4.759815731007763

DHAPT,0.32252132541666795,0.0

ENO,3.220598515000006,-0.8141184943357918

F6PA,0.32252132541666795,-0.001

FBA,1.2128609358333324,-0.17310293333153773

FBA3,0.19573216416666564,0.0

FBP,0.0,0.17310293333153773

FTHFLi,0.0,0.020836677986608265

FUM,1.2333893150000121,1.958488036688372

G6PDH2r,0.7056018925000015,0.0

GAPD,3.5642330149999975,-0.48140233332927207

GLCptspp,2.1404179895833364,0.0

GLCtex_copy1,2.1404179895833364,0.0

GLUDy,-1.7103415000000157,3.0583917700136545

GLYCL,0.010920900000001992,2.5610066954340205e-06

GND,0.7056018925000014,0.0

H2Otex,-9.914783537500057,-6.5919070620510585

H2Otpp,-9.914783537500059,-6.5919070620510585

Htex,-1.8376463999999728,2.9221693310155623

ICL,0.0,0.7240987216878885

MALS,0.0001338000000000684,0.7242325216878883

MDH,1.233197915000013,2.6833953583762606

MTHFC,0.1977522000000081,0.1759155220133902

MTHFD,0.1977522000000081,0.1759155220133902

NADH16pp,7.319156560000027,9.465936349479348

NADTRHD,0.0,3.3326944070320623

NH4tex,2.160164800000022,-2.599650931015447

NH4tpp,2.160164800000022,-2.599650931015447

O2tex,4.206049537500015,5.642988793084144

O2tpp,4.206049537500015,5.642988793084144

PDH,1.8733075150000043,3.300668280378994

PFK,1.2128609358333324,0.0

PFK_3,0.19573216416666564,0.0

PFL,0.0,0.02183667798660839

PGCD,0.34363450000000595,0.33271616100651963

PGI,1.434816097083335,0.0

PGK,-3.564233014999997,0.48140233332927207

PGL,0.7056018925000015,0.0

PGM,-3.2205985149999905,0.8141184943357918

PPC,0.5918716000000012,0.0

PPCK,0.0,0.13222712169016387

PPS,0.0,0.8476789726442634

PSERT,0.343634500000006,0.33271616100651963

PSP_L,0.343634500000006,0.33271616100651963

RPE,0.31519592833333127,-0.1552053333329563

RPI,-0.38244516416667035,-0.14724453333341467

SUCDi,1.024954315000004,1.7520530366889404

TALA,0.0,-0.03946846666658462

TKT1,0.1957321641666656,-0.03946846666658462

TKT2,0.11946376416666554,-0.11573686666637169

TPI,1.7028532254166657,-0.20236413333136263

62 reacctions require a flux change for the evolution of the ala__D consumer

.......................................................

.......................................................

ala__L

.......................................................

ACONTa,1.2398589150000043,1.96496019712743

ACONTb,1.2398589150000041,1.96496019712743

ADK1,0.524992600000009,1.3706664517575389

ALATA_L,-0.11627119999999999,4.640544531012335

ALAt2pp_copy1,0.0,4.75781573100775

ALAtex,0.0,4.75981573100775

ATPS4rpp,13.370320970000053,17.855782402848835

CO2tex,-4.632830737500008,-6.069769993049966

CO2tpp,-4.632830737500008,-6.069769993049966

CS,1.2398589150000043,1.96496019712743

CYTBO3_4pp,8.410403675000031,11.284282186110715

DHAPT,0.32252132541666795,0.0

ENO,3.220598515000006,-0.8141159338891042

F6PA,0.32252132541666795,-0.001000000000000013

FBA,1.2128609358333324,-0.17310293333237395

FBA3,0.19573216416666564,0.0

FBP,0.0,0.17310293333237395

FTHFLi,0.0,0.020841798883708407

FUM,1.2333893150000121,1.958490597126651

G6PDH2r,0.7056018925000015,0.0

GAPD,3.5642330149999975,-0.48140233333105753

GLCptspp,2.1404179895833364,0.0

GLCtex_copy1,2.1404179895833364,0.0

GLUDy,-1.7103415000000157,3.058395130456542

GLYCL,0.010920900000001992,5.580525486159615e-10

GND,0.7056018925000014,0.0

H2Otex,-9.914783537500057,-6.5919070620361335

H2Otpp,-9.914783537500059,-6.591907062035894

Htex,-1.8376463999999728,2.9221693310096093

ICL,0.0,0.7241012821274257

MALS,0.0001338000000000684,0.7242350821274257

MDH,1.233197915000013,2.683400479254077

MTHFC,0.1977522000000081,0.17591040111609882

MTHFD,0.1977522000000081,0.17591040111609882

NADH16pp,7.319156560000027,9.465933788983303

NADTRHD,0.0,3.332691046576676

NH4tex,2.160164800000022,-2.599650931009889

NH4tpp,2.160164800000022,-2.599650931009889

O2tex,4.206049537500015,5.642988793055357

O2tpp,4.206049537500015,5.642988793055357

PDH,1.8733075150000043,3.3006682803645395

PFK,1.2128609358333324,0.0

PFK_3,0.19573216416666564,0.0

PFL,0.0,0.02184179888370869

PGCD,0.34363450000000595,0.3327136005580467

PGI,1.434816097083335,0.0

PGK,-3.564233014999997,0.48140233333105753

PGL,0.7056018925000015,0.0

PGM,-3.2205985149999905,0.8141159338891042

PPC,0.5918716000000012,0.0

PPCK,0.0,0.13222968212815378

PPS,0.0,0.8476738517595861

PSERT,0.343634500000006,0.3327136005580467

PSP_L,0.343634500000006,0.3327136005580467

RPE,0.31519592833333127,-0.1552053333328323

RPI,-0.38244516416667035,-0.14724453333329077

SUCDi,1.024954315000004,1.7520555971274112

TALA,0.0,-0.03946846666652262

TKT1,0.1957321641666656,-0.03946846666652262

TKT2,0.11946376416666554,-0.11573686666630967

TPI,1.7028532254166657,-0.20236413333237396

61 reacctions require a flux change for the evolution of the ala__L consumer

.......................................................

.......................................................

alaala

.......................................................

ACONTa,1.2398589150000043,1.8567787283449286

ACONTb,1.2398589150000041,1.8567787283449286

ADK1,0.524992600000009,1.478851999033542

ALAALAabcpp,0.0,2.32581905035769

ALAALAr,0.005557600000000003,-2.32026145035817

ALAALAtex,0.0,2.32581905035769

ALAR,0.008336400000000004,-4.641300500720924

ALATA_L,-0.11627119999999999,4.532365700721404

ATPS4rpp,13.370320970000053,17.963962911622666

CO2tex,-4.632830737500008,-5.745237102195433

CO2tpp,-4.632830737500008,-5.745237102195433

CS,1.2398589150000043,1.8567787283449286

CYTBO3_4pp,8.410403675000031,10.635216404400623

DHAPT,0.32252132541666795,0.0

ENO,3.220598515000006,-0.814119772384125

F6PA,0.32252132541666795,-0.001

FBA,1.2128609358333324,-0.1731029333314162

FBA3,0.19573216416666564,0.0

FBP,0.0,0.1731029333314162

FTHFLi,0.0,0.020834121889848234

FUM,1.2333893150000121,1.8503091283452051

G6PDH2r,0.7056018925000015,0.0

GAPD,3.5642330149999975,-0.48140233332914323

GLCptspp,2.1404179895833364,0.0

GLCtex_copy1,2.1404179895833364,0.0

GLUDy,-1.7103415000000157,2.950212461671245

GLYCL,0.010920900000001992,3.839054984300372e-06

GND,0.7056018925000014,0.0

H2Otex,-9.914783537500057,-4.049732751103893

H2Otpp,-9.914783537500059,-4.049732751103893

Htex,-1.8376463999999728,2.8139917007237147

ICL,0.0,0.6159198133449244

MALS,0.0001338000000000684,0.6160536133449244

MDH,1.233197915000013,2.4670375416901296

MTHFC,0.1977522000000081,0.1759180781099661

MTHFD,0.1977522000000081,0.1759180781099661

NADH16pp,7.319156560000027,8.925049476054664

NADTRHD,0.0,3.224518454786208

NH4tex,2.160164800000022,-2.4914733007236114

NH4tpp,2.160164800000022,-2.4914733007236114

O2tex,4.206049537500015,5.318455902200311

O2tpp,4.206049537500015,5.318455902200311

PDH,1.8733075150000043,3.084313019792437

PFK,1.2128609358333324,0.0

PFK_3,0.19573216416666564,0.0

PFL,0.0,0.021834121889848356

PGCD,0.34363450000000595,0.3327174390549818

PGI,1.434816097083335,0.0

PGK,-3.564233014999997,0.48140233332914323

PGL,0.7056018925000015,0.0

PGM,-3.2205985149999905,0.814119772384125

PPC,0.5918716000000012,0.0

PPCK,0.0,0.024048213347181324

PPS,0.0,0.9558591590355794

PSERT,0.343634500000006,0.3327174390549818

PSP_L,0.343634500000006,0.3327174390549818

RPE,0.31519592833333127,-0.15520533333283476

RPI,-0.38244516416667035,-0.14724453333329313

SUCDi,1.024954315000004,1.6438741283459581

TALA,0.0,-0.03946846666652385

TKT1,0.1957321641666656,-0.03946846666652385

TKT2,0.11946376416666554,-0.1157368666663109

TPI,1.7028532254166657,-0.20236413333141617

63 reacctions require a flux change for the evolution of the alaala consumer

.......................................................

.......................................................

alltn

.......................................................

ACONTa,1.2398589150000043,2.5011197190160157

ACONTb,1.2398589150000041,2.5011197190160157

AKGDH,1.024820515000004,2.286081319015997

ALLTAMH,0.0,11.355606885185466

ALLTN,0.0,11.355606885185466

ALLTNt2rpp,0.0,11.355606885185466

ALLTNtex,0.0,11.355606885185466

ATPS4rpp,13.370320970000053,15.736926847873589

CO2tex,-4.632830737500008,-37.212750340764224

CO2tpp,-4.632830737500008,-37.212750340764224

CS,1.2398589150000043,2.5011197190160157

CYTBO3_4pp,8.410403675000031,5.436601570426297

DHAPT,0.32252132541666795,0.0

ENO,3.220598515000006,4.481859319008319

F6PA,0.32252132541666795,0.0

FBA,1.2128609358333324,-0.174102933451211

FBA3,0.19573216416666564,0.0

FBP,0.0,0.174102933451211

FUM,1.2333893150000121,2.4946501190159864

G6PDH2r,0.7056018925000015,3.561487805007235e-10

GAPD,3.5642330149999975,-0.48140233345007566

GLCptspp,2.1404179895833364,0.0

GLCtex_copy1,2.1404179895833364,0.0

GLXCL,0.0,5.306896152458387

GLYCK,0.0,4.044635348450059

GLYCK2,1.4997824761187406e-14,1.2622608040083287

GND,0.7056018925000014,3.561487805007235e-10

H2Otex,-9.914783537500057,36.994045055538294

H2Otpp,-9.914783537500059,36.99454505553877

Htex,-1.8376463999999728,43.58578114074277

ICDHyr,1.2398589150000043,2.5011197190160157

MALS,0.0001338000000000684,0.7419483802686914

MDH,1.233197915000013,3.0863303190157985

ME2,0.0,0.14994298026888034

NADH16pp,7.319156560000027,3.0840936514103

NH4tex,2.160164800000022,-43.26226274074275

NH4tpp,2.160164800000022,-43.26226274074275

O2tex,4.206049537500015,2.7193984852131443

O2tpp,4.206049537500015,2.7191484852131484

PDH,1.8733075150000043,3.8763828992782905

PFK,1.2128609358333324,0.0

PFK_3,0.19573216416666564,0.0

PGI,1.434816097083335,-3.561487805007235e-10

PGK,-3.564233014999997,0.48140233345007566

PGL,0.7056018925000015,3.561487805007235e-10

PPC,0.5918716000000012,0.0

PYK,0.0,4.316071719009684

RPE,0.31519592833333127,-0.15520533309552054

RPI,-0.38244516416667035,-0.14724453345212773

SUCDi,1.024954315000004,2.286215119015997

SUCOAS,-0.9198485150000041,-2.181109319015997

TALA,0.0,-0.03946846654786673

TKT1,0.1957321641666656,-0.03946846654786673

TKT2,0.11946376416666554,-0.11573686654765382

TPI,1.7028532254166657,-0.20236413345121057

TRSARr,0.0,5.305896152458387

UGLYCH,0.0,11.355606885185466

57 reacctions require a flux change for the evolution of the alltn consumer

.......................................................

.......................................................

anhgm

.......................................................

ACGAMK,0.0,0.7082915055176736

ACKr,0.11651519999999993,1.532098211032087

ACM6PH,0.0,0.7082915055176736

ACONTa,1.2398589150000043,2.0525043863694923

ACONTb,1.2398589150000041,2.0525043863694923

AGDC,0.0,1.4165830110353472

AGMH,0.0,0.7082915055176736

AGMt2pp,0.0,0.7082915055176736

AKGDH,1.024820515000004,1.837465986369954

ANHGMtex,0.0,0.7082915055176736

ANHMK,0.0,0.7082915055176736

ATPS4rpp,13.370320970000053,15.267589955076394

CO2tex,-4.632830737500008,-5.247861404878765

CO2tpp,-4.632830737500008,-5.247861404878765

CS,1.2398589150000043,2.0525043863694923

CYTBO3_4pp,8.410403675000031,9.64046500976762

ENO,3.220598515000006,1.9083694698041795

FBA,1.2128609358333324,0.6264668691833951

FUM,1.2333893150000121,2.046034786369943

G6PDA,0.0,1.397685411266352

G6PDH2r,0.7056018925000015,0.2992791568112516

GAPD,3.5642330149999975,2.252003969804171

GF6PTA,0.018897600000000007,2.2958622503121973e-10

GLCptspp,2.1404179895833364,0.0

GLCtex_copy1,2.1404179895833364,0.0

GLNS,0.36212300000000835,0.34322540022957426

GND,0.7056018925000014,0.2992791568112516

H2Otex,-9.914783537500057,-5.571773666240884

H2Otpp,-9.914783537500059,-5.571773666240884

Htex,-1.8376463999999728,0.2872281165590599

ICDHyr,1.2398589150000043,2.0525043863694923

LDH_D,0.0,0.7082915055176736

MDH,1.233197915000013,2.0458433863699437

NADH16pp,7.319156560000027,7.734572423397665

NH4tex,2.160164800000022,0.7435817889596479

NH4tpp,2.160164800000022,0.7435817889596479

O2tex,4.206049537500015,4.82108020488381

O2tpp,4.206049537500015,4.82108020488381

PDH,1.8733075150000043,1.269369975326768

PFK,1.2128609358333324,0.6264668691833951

PGI,1.434816097083335,-0.2992791568112516

PGK,-3.564233014999997,-2.252003969804171

PGL,0.7056018925000015,0.2992791568112516

PGM,-3.2205985149999905,-1.9083694698041795

PTAr,-0.11651519999999993,-1.532098211032087

PYK,0.0,0.8291889443905649

RPE,0.31519592833333127,0.044314104541214636

RPI,-0.38244516416667035,-0.24700425227049538

SUCDi,1.024954315000004,1.839599786369954

SUCOAS,-0.9198485150000041,-1.7324939863704096

TALA,0.0,-0.13444091189616478

TKT1,0.1957321641666656,0.06029125227050086

TKT2,0.11946376416666554,-0.01597714772928623

TPI,1.7028532254166657,1.1144591587667283

54 reacctions require a flux change for the evolution of the anhgm consumer

.......................................................

.......................................................

arg__L

.......................................................

ACGK,0.05915840000000002,0.0

ACGS,0.05915840000000002,0.0

ACODA,0.059158400000000014,0.0

ACONTa,1.2398589150000043,-0.23089110146521324

ACONTb,1.2398589150000041,-0.23089110146521324

ACOTA,-0.05915840000000002,-0.0

ACS,0.0,0.17273290315937356

ADK1,0.524992600000009,0.638031808579162

AGPR,-0.05915840000000003,0.0

AKGDH,1.024820515000004,2.1259851470964537

ARGORNt7pp,0.0,2.572603648559686

ARGSL,0.05915840000000001,0.0

ARGSS,0.05915840000000001,0.0

ARGtex,0.0,2.572603648559686

ASPT,0.0,0.05925581995139595

AST,0.0,2.5133994485616666

ATPS4rpp,13.370320970000053,18.40997501622611

CBMKr,0.12531739999999997,0.066159

CITL,0.0,0.23089110146521324

CO2tex,-4.632830737500008,-7.228962991155601

CO2tpp,-4.632830737500008,-7.228962991155601

CS,1.2398589150000043,0.0

CYTBO3_4pp,8.410403675000031,11.02982437404631

DHAPT,0.32252132541666795,0.0

ENO,3.220598515000006,-0.8261913517325201

F6PA,0.32252132541666795,-0.001

FBA,1.2128609358333324,-0.17304046001470974

FBA3,0.19573216416666564,0.0

FBP,0.0,0.17304046001470974

FUM,1.2333893150000121,2.335555547098444

G6PDH2r,0.7056018925000015,0.0

GAPD,3.5642330149999975,-0.4811235500368962

GLCptspp,2.1404179895833364,0.0

GLCtex_copy1,2.1404179895833364,0.0

GLUDy,-1.7103415000000157,3.4311309971258694

GND,0.7056018925000014,0.0

H2Otex,-9.914783537500057,0.3539153713533878

H2Otpp,-9.914783537500059,0.35391537135387097

Htex,-1.8376463999999728,5.88081972201266

ICDHyr,1.2398589150000043,-0.23189110146521325

MDH,1.233197915000013,2.3362303470984003

MTHFC,0.1977522000000081,0.1964341099768157

MTHFD,0.1977522000000081,0.1964341099768157

NADH16pp,7.319156560000027,8.830503026949899

NADTRHD,0.0,2.301111200748566

NH4tex,2.160164800000022,-8.130488144123023

NH4tpp,2.160164800000022,-8.130488144123023

O2tex,4.206049537500015,5.515955588557423

O2tpp,4.206049537500015,5.515955588557423

OCBT,0.05915840000000002,0.0

PDH,1.8733075150000043,0.4016558284543564

PFK,1.2128609358333324,0.0

PFK_3,0.19573216416666564,0.0

PFL,0.0,0.0012222700737789943

PGCD,0.34363450000000595,0.34563470169562316

PGI,1.434816097083335,0.0

PGK,-3.564233014999997,0.4811235500368962

PGL,0.7056018925000015,0.0

PGM,-3.2205985149999905,0.8261913517325201

PPC,0.5918716000000012,0.0

PPCK,0.0,1.979910848563648

PPK,-0.7007176000000049,-0.8135621168564094

PSERT,0.343634500000006,0.34563470169562316

PSP_L,0.343634500000006,0.34563470169562316

PTRCORNt7pp,0.0,-2.572603648559686

PTRCt2pp,0.0,2.572603648559686

PYK,0.0,0.9879318968324923

RPE,0.31519592833333127,-0.1551428600151894

RPI,-0.38244516416667035,-0.14718206001566614

SADH,0.0,2.5133994485616666

SGDS,0.0,2.5133994485616666

SGSAD,0.0,2.5133994485616666

SOTA,0.0,2.5133994485616666

SUCDi,1.024954315000004,2.1291189470964538

SUCOAS,-0.9198485150000041,0.49238670146323216

TALA,0.0,-0.0394372300077066

TKT1,0.1957321641666656,-0.0394372300077066

TKT2,0.11946376416666554,-0.1157056300074828

TPI,1.7028532254166657,-0.20230006001471068

79 reacctions require a flux change for the evolution of the arg__L consumer

.......................................................

.......................................................

asn__L

.......................................................

ACONTa,1.2398589150000043,1.6980137734848313

ACONTb,1.2398589150000041,1.6980137734848313

ADK1,0.524992600000009,0.47577880000005734

AKGDH,1.024820515000004,1.482975373484778

ASNN,0.0,4.455409506818169

ASNS2,0.04821100000000001,0.0

ASNt2rpp,0.0,4.503620506818152

ASNtex,0.0,4.503620506818152

ASPT,0.0,4.504621706818183

ATPS4rpp,13.370320970000053,16.49190259318175

CO2tex,-4.632830737500008,-9.804819227272674

CO2tpp,-4.632830737500008,-9.804819227272674

CS,1.2398589150000043,1.698013773484778

CYTBO3_4pp,8.410403675000031,9.747142040909049

DHAPT,0.32252132541666795,0.0

ENO,3.220598515000006,-0.8248683333333702

F6PA,0.32252132541666795,0.0

FBA,1.2128609358333324,-0.17410213333334923

FBA3,0.19573216416666564,0.0

FBP,0.0,0.17410213333333194

FUM,1.2333893150000121,6.19616508030299

G6PDH2r,0.7056018925000015,0.0

GAPD,3.5642330149999975,-0.4814003333333403

GLCptspp,2.1404179895833364,0.0

GLCtex_copy1,2.1404179895833364,-0.0009999999999763531

GND,0.7056018925000014,0.0

H2Otex,-9.914783537500057,-1.5759029068181007

H2Otpp,-9.914783537500059,-1.5759029068181007

Htex,-1.8376463999999728,7.169596213636282

ICDHyr,1.2398589150000043,1.6980137734848313

MDH,1.233197915000013,3.2770245068180657

ME1,0.0,1.9607198469696656

ME2,0.0,0.9582397265151124

NADH16pp,7.319156560000027,8.19774006742427

NH4tex,2.160164800000022,-6.847079013636403

NH4tpp,2.160164800000022,-6.847079013636403

O2tex,4.206049537500015,4.874418720454514

O2tpp,4.206049537500015,4.874418720454514

PDH,1.8733075150000043,2.332131773484907

PFK,1.2128609358333324,0.0

PFK_3,0.19573216416666564,0.0

PGI,1.434816097083335,0.0

PGK,-3.564233014999997,0.4814003333333403

PGL,0.7056018925000015,0.0

PGM,-3.2205985149999905,0.8249352333333491

PPC,0.5918716000000012,0.0

PPCK,0.0,0.9916559333333915

PPK,-0.7007176000000049,-0.6545038000000432

RPE,0.31519592833333127,-0.15520453333328987

RPI,-0.38244516416667035,-0.14724373333331187

SUCDi,1.024954315000004,1.484109173484778

SUCOAS,-0.9198485150000041,-1.3780037734848065

TALA,0.0,-0.0394680666667

TKT1,0.1957321641666656,-0.0394680666667

TKT2,0.11946376416666554,-0.11573646666670356

TPI,1.7028532254166657,-0.20236333333332368

56 reacctions require a flux change for the evolution of the asn__L consumer

.......................................................

.......................................................

asp__L

.......................................................

ACONTa,1.2398589150000043,1.702293791681579

ACONTb,1.2398589150000041,1.702293791681579

AKGDH,1.024820515000004,1.487255391682128

ASPT,0.0,4.507003325010398

ASPt2pp_copy1,0.0,4.5070033250000625

ASPtex,0.0,4.508003325000039

ATPS4rpp,13.370320970000053,16.53575277514432

CO2tex,-4.632830737500008,-9.822336100036171

CO2tpp,-4.632830737500008,-9.822336100036171

CS,1.2398589150000043,1.702293791681579

CYTBO3_4pp,8.410403675000031,9.773407750088527

DHAPT,0.32252132541666795,0.0

ENO,3.220598515000006,-0.8249699333306136

F6PA,0.32252132541666795,-0.0010000000001751021

FBA,1.2128609358333324,-0.17310293351210956

FBA3,0.19573216416666564,1.7990942069445737e-10

FBP,0.0,0.17310293351210956

FUM,1.2333893150000121,6.2028275166917695

G6PDH2r,0.7056018925000015,0.0

GAPD,3.5642330149999975,-0.4814023333308857

GLCptspp,2.1404179895833364,0.0

GLCtex_copy1,2.1404179895833364,0.0

GND,0.7056018925000014,0.0

H2Otex,-9.914783537500057,-6.08828225002749

H2Otpp,-9.914783537500059,-6.088282250027215

Htex,-1.8376463999999728,7.178360250002877

ICDHyr,1.2398589150000043,1.702293791681579

MDH,1.233197915000013,3.278261925019683

ME1,0.0,1.9724714833574932

ME2,0.0,0.9527689083145927

NADH16pp,7.319156560000027,8.2197257584064

NH4tex,2.160164800000022,-2.3478385250046143

NH4tpp,2.160164800000022,-2.3478385250046143

O2tex,4.206049537500015,4.887551575044263

O2tpp,4.206049537500015,4.887551575044263

PDH,1.8733075150000043,2.3356085916721696

PFK,1.2128609358333324,0.0

PFK_3,0.19573216416666564,1.7990942069445737e-10

PGI,1.434816097083335,0.0

PGK,-3.564233014999997,0.4814023333308857

PGL,0.7056018925000015,0.0

PGM,-3.2205985149999905,0.8249699333306136

PPC,0.5918716000000012,0.0

PPCK,0.0,0.9907575333292494

RPE,0.31519592833333127,-0.15520533333283357

RPI,-0.38244516416667035,-0.14724453333329193

SUCDi,1.024954315000004,1.4883891916821281

SUCOAS,-0.9198485150000041,-1.3822833916821036

TALA,0.0,-0.03946846684643266

TKT1,0.1957321641666656,-0.03946846666652323

TKT2,0.11946376416666554,-0.11573686666631033

TPI,1.7028532254166657,-0.20236413333220016

52 reacctions require a flux change for the evolution of the asp__L consumer

.......................................................

.......................................................

cgly

.......................................................

ACKr,0.11651519999999993,0.06594080017694992

ACONTa,1.2398589150000043,2.5994796230914563

ACONTb,1.2398589150000041,2.5994796230914563

ADK1,0.524992600000009,1.0750337333285256

ADK3,0.0,0.04857440000000679

ADSK,0.04957439999999991,0.0

AKGDH,1.024820515000004,1.7925696230921662

AMPTASECG,0.0,3.6036675709426853

ATPS4rpp,13.370320970000053,19.938873348009224

BPNT,0.049574399999999914,0.0

CGLYabcpp,0.0,3.6036675709426853

CGLYtex,0.0,3.6036675709426853

CO2tex,-4.632830737500008,-9.808660654739583

CO2tpp,-4.632830737500008,-9.808170054739662

CS,1.2398589150000043,2.5994796230914563

CYSDS,0.0,3.5540931711210306

CYSS,0.04957439999999994,1.7834485277479217e-10

CYTBO3_4pp,8.410403675000031,11.753025967603861

DHAPT,0.32252132541666795,0.0

ENO,3.220598515000006,-0.4814023333314026

F6PA,0.32252132541666795,0.0

FBA,1.2128609358333324,-0.17410293333250593

FBA3,0.19573216416666564,0.0

FBP,0.0,0.17410293333249552

FUM,1.2333893150000121,2.5930100230908617

G6PDH2r,0.7056018925000015,0.0

GAPD,3.5642330149999975,-0.4814023333314026

GHMT2r,0.22309610000000604,-1.578492385471349

GLCptspp,2.1404179895833364,0.0

GLCtex_copy1,2.1404179895833364,0.0

GLUDy,-1.7103415000000157,-1.3667069999980868

GLYCL,0.010920900000001992,1.8129999854713403

GND,0.7056018925000014,0.0

H2Otex,-9.914783537500057,-0.6759431709627961

H2Otpp,-9.914783537500059,-0.6759431709622277

H2St1pp,0.0,3.5540931709426857

H2Stex,0.0,-3.5540931709426857

Htex,-1.8376463999999728,5.270539941888046

ICDHyr,1.2398589150000043,2.007608023092189

ICL,0.0,0.5918715999992713

MALS,0.0001338000000000684,0.5920053999992713

MDH,1.233197915000013,3.184734823090133

NADH16pp,7.319156560000027,9.300801344512449

NH4tex,2.160164800000022,-5.047170341888091

NH4tpp,2.160164800000022,-5.047170341888091

O2tex,4.206049537500015,5.87736068380193

O2tpp,4.206049537500015,5.87736068380193

PAPSR,0.049574399999999914,0.0

PDH,1.8733075150000043,3.8247998230841205

PFK,1.2128609358333324,0.0

PFK_3,0.19573216416666564,0.0

PGCD,0.34363450000000595,0.0

PGI,1.434816097083335,0.0

PGK,-3.564233014999997,0.4814023333314026

PGL,0.7056018925000015,0.0

PGM,-3.2205985149999905,0.4814023333314026

PPC,0.5918716000000012,0.0

PPK,-0.7007176000000049,-0.653143199996407

PPS,0.0,0.6471899333299971

PSERT,0.343634500000006,0.0

PSP_L,0.343634500000006,0.0

PTAr,-0.11651519999999993,-0.06594080017694992

RPE,0.31519592833333127,-0.15520533333290132

RPI,-0.38244516416667035,-0.14724453333337806

SADT2,0.04957439999999991,0.0

SERAT,0.04957439999999993,1.7834485277479217e-10

SERD_L,0.0,1.5065283852930043

SO4t2pp,0.05044199999999991,0.0008676000000000001

SO4tex,0.05044199999999991,0.0008676000000000001

SUCDi,1.024954315000004,2.3865750230913902

SUCOAS,-0.9198485150000041,-1.6885976230921642

SULR,0.049574399999999914,0.0

TALA,0.0,-0.03946846666656256

THD2pp,0.0,0.10103197690246946

TKT1,0.1957321641666656,-0.03946846666656256

TKT2,0.11946376416666554,-0.11573686666633876

TPI,1.7028532254166657,-0.20236413333248038

TRDR,0.049574399999999914,0.0

78 reacctions require a flux change for the evolution of the cgly consumer

.......................................................

.......................................................

cit

.......................................................

ACONTa,1.2398589150000043,3.204961566682045

ACONTb,1.2398589150000041,3.204961566682045

AKGDH,1.024820515000004,2.9899231666825936

ATPS4rpp,13.370320970000053,15.033085000144865

CITt7pp,0.0,3.0053355500004053

CITtex,0.0,3.0053355500004053

CO2tex,-4.632830737500008,-9.822336100036182

CO2tpp,-4.632830737500008,-9.822336100036182

CS,1.2398589150000043,0.19962601668163948

CYTBO3_4pp,8.410403675000031,9.77340775008928

DHAPT,0.32252132541666795,0.0

ENO,3.220598515000006,-0.8249699333306353

F6PA,0.32252132541666795,-0.0010000000002037268

FBA,1.2128609358333324,-0.17310373350653663

FBA3,0.19573216416666564,8.001743445618104e-07

FBP,0.0,0.17310373350653213

FUM,1.2333893150000121,3.198491966682468

G6PDH2r,0.7056018925000015,0.0

GAPD,3.5642330149999975,-0.48140233333083415

GLCptspp,2.1404179895833364,0.0

GLCtex_copy1,2.1404179895833364,0.0

GND,0.7056018925000014,0.0

H2Otex,-9.914783537500057,-9.094117800028698

H2Otpp,-9.914783537500059,-9.09361780002871

Htex,-1.8376463999999728,7.179360250002219

ICDHyr,1.2398589150000043,3.204961566682045

MDH,1.233197915000013,1.7755941500109884

ME1,0.0,1.422572616671424

NADH16pp,7.319156560000027,6.717057983406688

NADTRHD,0.0,0.5478943060917838

O2tex,4.206049537500015,4.88780157504464

O2tpp,4.206049537500015,4.88755157504464

PDH,1.8733075150000043,0.8339408166719691

PFK,1.2128609358333324,0.0

PFK_3,0.19573216416666564,8.001743445618104e-07

PGI,1.434816097083335,0.0

PGK,-3.564233014999997,0.48140233333083415

PGL,0.7056018925000015,0.0

PGM,-3.2205985149999905,0.8249699333306353

PPC,0.5918716000000012,0.0

PPCK,0.0,0.9907575333297114

RPE,0.31519592833333127,-0.15520533333290132

RPI,-0.38244516416667035,-0.14724453333326437

SUCCt2_2pp,0.0,3.0053355500004053

SUCDi,1.024954315000004,2.9900569666825936

SUCOAS,-0.9198485150000041,-2.8849511666825265

TALA,0.0,-0.03946926684091068

TKT1,0.1957321641666656,-0.03946846666656256

TKT2,0.11946376416666554,-0.11573686666633876

TPI,1.7028532254166657,-0.20236413333213932

50 reacctions require a flux change for the evolution of the cit consumer

.......................................................

.......................................................

cys__L

.......................................................

ACKr,0.11651519999999993,0.0669407999980649

ACONTa,1.2398589150000043,3.3307136183831796

ACONTb,1.2398589150000041,3.3307136183831796

ADK1,0.524992600000009,1.031442514928699

ADK3,0.0,0.05057440000018687

ADSK,0.04957439999999991,0.0

AKGDH,1.024820515000004,2.201073199160089

ATPS4rpp,13.370320970000053,22.31815173411769

BPNT,0.049574399999999914,0.0

CO2tex,-4.632830737500008,-10.16703025761592

CO2tpp,-4.632830737500008,-10.16703025761592

CS,1.2398589150000043,3.3307136183831796

CYSDS,0.0,6.075994752525625

CYSS,0.04957439999999994,0.0

CYSabcpp,0.0,6.125569152525624

CYStex,0.0,6.125569152525624

CYTBO3_4pp,8.410403675000031,13.551531162727668

DHAPT,0.32252132541666795,0.0

ENO,3.220598515000006,-0.7645415341564785

F6PA,0.32252132541666795,-0.0009999999999763531

FBA,1.2128609358333324,-0.1731029333324159

FBA3,0.19573216416666564,0.0

FBP,0.0,0.17310293333237572

FTHFLi,0.0,0.020841798347461804

FUM,1.2333893150000121,3.3242440183835242

G6PDH2r,0.7056018925000015,0.0

GAPD,3.5642330149999975,-0.48140233333072047

GLCptspp,2.1404179895833364,0.0

GLCtex_copy1,2.1404179895833364,0.0

GLUDy,-1.7103415000000157,-1.6508462008149536

GLYCL,0.010920900000001992,8.261765716000014e-10

GND,0.7056018925000014,0.0

H2Otex,-9.914783537500057,-3.19784475255733

H2Otpp,-9.914783537500059,-3.1978447525568754

H2St1pp,0.0,6.075994752525625

H2Stex,0.0,-6.075994752525625

Htex,-1.8376463999999728,4.188773952527072

ICDHyr,1.2398589150000043,2.416111599159535

ICL,0.0,0.9146020192236393

MALS,0.0001338000000000684,0.9147358192236392

MDH,1.233197915000013,4.238654637607169

MTHFC,0.1977522000000081,0.17591040165234517

MTHFD,0.1977522000000081,0.17591040165234517

NADH16pp,7.319156560000027,10.36742934434394

NH4tex,2.160164800000022,-3.965404352527912

NH4tpp,2.160164800000022,-3.965404352527912

O2tex,4.206049537500015,6.776613281363834

O2tpp,4.206049537500015,6.776613281363834

PAPSR,0.049574399999999914,0.0

PDH,1.8733075150000043,4.856922439247094

PFK,1.2128609358333324,0.0

PFK_3,0.19573216416666564,0.0

PFL,0.0,0.02184179834746184

PGCD,0.34363450000000595,0.2831392008258071

PGI,1.434816097083335,0.0

PGK,-3.564233014999997,0.48140233333072047

PGL,0.7056018925000015,0.0

PGM,-3.2205985149999905,0.7645415341564785

PPC,0.5918716000000012,0.0

PPCK,0.0,0.3227304192243694

PPK,-0.7007176000000049,-0.6521431999959759

PPS,0.0,0.6075987149307709

PSERT,0.343634500000006,0.2831392008258071

PSP_L,0.343634500000006,0.2831392008258071

PTAr,-0.11651519999999993,-0.0669407999980649

RPE,0.31519592833333127,-0.15520533333290132

RPI,-0.38244516416667035,-0.14724453333326437

SADT2,0.04957439999999991,0.0

SERAT,0.04957439999999993,0.0

SO4t2pp,0.05044199999999991,0.0008676000000000001

SO4tex,0.05044199999999991,0.0008676000000000001

SUCDi,1.024954315000004,3.1178090183837286

SUCOAS,-0.9198485150000041,-2.0961011991601026

SULR,0.049574399999999914,0.0

TALA,0.0,-0.03946846666656256

TKT1,0.1957321641666656,-0.03946846666656256

TKT2,0.11946376416666554,-0.11573686666633876

TPI,1.7028532254166657,-0.20236413333202563

TRDR,0.049574399999999914,0.0

79 reacctions require a flux change for the evolution of the cys__L consumer

.......................................................

.......................................................

cytd

.......................................................

ACONTa,1.2398589150000043,0.9462844365909859

ACONTb,1.2398589150000041,0.9462844365909859

ADK3,0.0,-0.06702760000007402

AKGDH,1.024820515000004,0.7312460365910154

ASPCT,0.06615899999999995,0.0

ASPTA,-0.5855358000000094,-0.5193755999999894

ATPS4rpp,13.370320970000053,11.451594033719289

CBMKr,0.12531739999999997,0.059158400000001166

CO2tex,-4.632830737500008,-3.9324410237610437

CO2tpp,-4.632830737500008,-3.9324410237610437

CS,1.2398589150000043,0.9462844365910154

CTPS2,0.03210499999999995,0.0

CYTBO3_4pp,8.410403675000031,6.811225694766066

CYTD,0.0,2.3433695529411827

CYTDK2,0.0,0.032105

CYTDt2pp_copy2,0.0,2.3744745529411375

CYTDtex,0.0,2.3754745529412276

CYTK1,0.036180999999999956,0.06826698818895238

DHORD2,0.06615899999999995,0.0

DHORTS,-0.06615899999999995,0.0

ENO,3.220598515000006,2.8608448247799743

FBA,1.2128609358333324,0.6200286704444125

FUM,1.2333893150000121,0.939814036590974

G6PDH2r,0.7056018925000015,0.8197962257989957

GAPD,3.5642330149999975,3.2044775247800317

GLCptspp,2.1404179895833364,0.0

GLCtex_copy1,2.1404179895833364,-0.0009999999999763531

GLNS,0.36212300000000835,0.3300171999999915

GLUDy,-1.7103415000000157,-1.6441794999999502

GND,0.7056018925000014,0.8197962257989826

H2Otex,-9.914783537500057,-4.13262137772108

H2Otpp,-9.914783537500059,-4.132623106067626

Htex,-1.8376463999999728,0.6701442962482815

ICDHyr,1.2398589150000043,0.9462844365909859

MDH,1.233197915000013,0.939667236591049

NADH16pp,7.319156560000027,6.079712058175051

NDPK2,0.08505659999999997,0.05195160000005217

NDPK3,0.03618099999999997,0.06826698818895238

NH4tex,2.160164800000022,-0.34763055294115475

NH4tpp,2.160164800000022,-0.34763055294115475

O2tex,4.206049537500015,3.4064605473830625

O2tpp,4.206049537500015,3.4064605473830625

OMPDC,0.06615899999999997,0.0

ORPT,-0.06615899999999995,0.0

PDH,1.8733075150000043,1.5797508484020284

PFK,1.2128609358333324,0.6200286704444632

PGI,1.434816097083335,-0.8197962257989957

PGK,-3.564233014999997,-3.2044775247800317

PGL,0.7056018925000015,0.8197962257989826

PGM,-3.2205985149999905,-2.8608448247799743

PPC,0.5918716000000012,0.5257121999999999

PPK,-0.7007176000000049,-0.6356537047244046

PPM,-0.00013460000000000268,2.3093155529411433

PRPPS,0.1865784000000047,0.12055280000004132

PYK,0.0,1.8478236993633348

PYNP2r,0.0,2.3103155529412334

RPE,0.31519592833333127,1.9749759858267453

RPI,-0.38244516416667035,1.1631405600278413

SUCDi,1.024954315000004,0.7315136365910153

SUCOAS,-0.9198485150000041,-0.6262744365909612

TALA,0.0,0.830890028746694

TKT1,0.1957321641666656,1.0256221929133744

TKT2,0.11946376416666554,0.9493537929133709

TPI,1.7028532254166657,1.1080209600278295

UMPK,0.07560779999999996,0.042502799999965646

URAt2pp_copy2,0.0,-2.3093155529411433

URAtex,0.0,-2.3093155529411433

URIK2,0.0,0.033054

68 reacctions require a flux change for the evolution of the cytd consumer

.......................................................

.......................................................

dha

.......................................................

DHAPT,0.32252132541666795,2.461939524986299

DHAtex,0.0,4.280835954142101

DHAtpp,0.0,4.280835954142101

F6PA,0.32252132541666795,-1.8188964291558019

GLCptspp,2.1404179895833364,0.0

GLCtex_copy1,2.1404179895833364,0.0

PGI,1.434816097083335,-0.7056011874933714

TPI,1.7028532254166657,3.843271424979925

8 reacctions require a flux change for the evolution of the dha consumer

.......................................................

.......................................................

etha

.......................................................

ACALD,0.0,5.94436030450495

ACONTa,1.2398589150000043,3.138583871195225

ACONTb,1.2398589150000041,3.138583871195225

AKGDH,1.024820515000004,1.340849437871082

ATPS4rpp,13.370320970000053,16.682158729079667

CO2tex,-4.632830737500008,-3.679043409069114

CO2tpp,-4.632830737500008,-3.679043409069114

CS,1.2398589150000043,3.138583871195225

CYTBO3_4pp,8.410403675000031,12.447189322662702

DHAPT,0.32252132541666795,0.0

ENO,3.220598515000006,-0.8250368333278646

ETHAAL,0.0,5.94436030450495

ETHAt2pp,5.655907058495112e-35,5.94436030450495

ETHAtex,0.0,5.94436030450495

F6PA,0.32252132541666795,0.0

FBA,1.2128609358333324,-0.1741041336409706

FBA3,0.19573216416666564,1.200309693416557e-06

FBP,0.0,0.1741041336409706

FLDR2,0.0,0.587631799998136

FUM,1.2333893150000121,3.132114271195497

G6PDH2r,0.7056018925000015,0.0

GAPD,3.5642330149999975,-0.48140233332843035

GLCptspp,2.1404179895833364,0.0

GLCtex_copy1,2.1404179895833364,0.0

GND,0.7056018925000014,0.0

H2Otex,-9.914783537500057,-8.961496209047466

H2Otpp,-9.914783537500059,-8.960996209050581

ICDHyr,1.2398589150000043,1.555887837870621

ICL,0.0,1.582696033324604

MALS,0.0001338000000000684,1.582829833324604

MDH,1.233197915000013,4.714618904520101

NADH16pp,7.319156560000027,9.455217251467223

NH4tex,2.160164800000022,-3.784195504511268

NH4tpp,2.160164800000022,-3.784195504511268

O2tex,4.206049537500015,6.224692361329793

O2tpp,4.206049537500015,6.22444236133135

PDH,1.8733075150000043,0.0

PFK,1.2128609358333324,0.0

PFK_3,0.19573216416666564,1.200309693416557e-06

PGI,1.434816097083335,0.0

PGK,-3.564233014999997,0.48140233332843035

PGL,0.7056018925000015,0.0

PGM,-3.2205985149999905,0.8250368333278646

POR5,0.021750999999999996,-0.5678807999983428

PPC,0.5918716000000012,0.0

PPCK,0.0,0.9908244333265002

RPE,0.31519592833333127,-0.15520533333269576

RPI,-0.38244516416667035,-0.14724453333315407

SUCDi,1.024954315000004,2.925679271195686

SUCOAS,-0.9198485150000041,-1.2358774378715376

TALA,0.0,-0.03946966697614775

THD2pp,0.0,1.684806662118403

TKT1,0.1957321641666656,-0.03946846666645433

TKT2,0.11946376416666554,-0.11573686666624142

TPI,1.7028532254166657,-0.20236413333091183

55 reacctions require a flux change for the evolution of the etha consumer

.......................................................

.......................................................

etoh

.......................................................

ACALD,0.0,4.88758510369189

ACONTa,1.2398589150000043,2.0818086703688348

ACONTb,1.2398589150000041,2.0818086703688348

ADK1,0.524992600000009,1.2647396779600366

ALCD2x,0.0,4.88758510369189

ATPS4rpp,13.370320970000053,17.738933929612585

CO2tex,-4.632830737500008,-1.56549300740744

CO2tpp,-4.632830737500008,-1.56549300740744

CS,1.2398589150000043,2.0818086703688348

CYTBO3_4pp,8.410403675000031,12.050898422209832

DHAPT,0.32252132541666795,0.0

ENO,3.220598515000006,-0.82503683333141

ETOHtex,0.0,4.88758510369189

ETOHtrpp,0.0,4.88758510369189

F6PA,0.32252132541666795,0.0

FBA,1.2128609358333324,-0.17410293333250593

FBA3,0.19573216416666564,0.0

FBP,0.0,0.17410293333249557

FLDR2,0.0,1.3273780779609015

FUM,1.2333893150000121,2.0753390703688264

G6PDH2r,0.7056018925000015,0.0

GAPD,3.5642330149999975,-0.4814023333314026

GLCptspp,2.1404179895833364,0.0

GLCtex_copy1,2.1404179895833364,0.0

GND,0.7056018925000014,0.0

H2Otex,-9.914783537500057,-11.735030911094782

H2Otpp,-9.914783537500059,-11.735030911094327

ICL,0.0,0.842949755368799

MALS,0.0001338000000000684,0.843083555368799

MDH,1.233197915000013,2.9180974257376255

NADH16pp,7.319156560000027,10.117701551840996

O2tex,4.206049537500015,6.026296911104916

O2tpp,4.206049537500015,6.026296911104916

PDH,1.8733075150000043,0.0

PFK,1.2128609358333324,0.0

PFK_3,0.19573216416666564,0.0

PGI,1.434816097083335,0.0

PGK,-3.564233014999997,0.4814023333314026

PGL,0.7056018925000015,0.0

PGM,-3.2205985149999905,0.82503683333141

POR5,0.021750999999999996,-1.3076270779608876

PPC,0.5918716000000012,0.0

PPCK,0.0,0.25107815536900535

PPS,0.0,0.7397462779609856

RPE,0.31519592833333127,-0.15520533333290132

RPI,-0.38244516416667035,-0.14724453333337806

SUCDi,1.024954315000004,1.866904070368835

TALA,0.0,-0.03946846666656256

THD2pp,0.0,2.7415810629570325

TKT1,0.1957321641666656,-0.03946846666656256

TKT2,0.11946376416666554,-0.11573686666633876

TPI,1.7028532254166657,-0.20236413333248038

52 reacctions require a flux change for the evolution of the etoh consumer

.......................................................

.......................................................

for

.......................................................

ACONTa,1.2398589150000043,0.21503847268144227

ACONTb,1.2398589150000041,0.21503847268144227

ADK1,0.524992600000009,11.928186874307945

ADSL2r,0.08960300000000408,7.243841807658064

AICART,0.10855060000000409,7.2627894076580635

AIRC2,0.08960300000000408,7.243841807658064

AIRC3,-0.08960300000000408,-7.243841807658064

AKGDH,1.024820515000004,0.0

ALLTAMH,0.0,7.154238807658068

ALLTN,0.0,7.154238807658068

ASAD,-0.21381520000000004,-10.377948013660168

ASPK,0.2138152,10.377948013660168

ASPTA,-0.5855358000000094,-17.90390742131823

ATPS4rpp,13.370320970000053,120.43413504972683

CO2tex,-4.632830737500008,-104.70629206113874

CO2tpp,-4.632830737500008,-9.13053622134615

CS,1.2398589150000043,0.21503847268144227

CYTBO3_4pp,8.410403675000031,95.64218251247401

DHAPT,0.32252132541666795,0.0

ENO,3.220598515000006,-0.48140233333136145

F6PA,0.32252132541666795,0.0

FBA,1.2128609358333324,-0.1741029333324955

FBA3,0.19573216416666564,0.0

FBP,0.0,0.1741029333324955

FDH4pp,0.0,95.57475583979259

FE2tpp,0.0032121999999999997,0.0016506000000000001

FE3abcpp,0.0,0.0015616000000000002

FESR,0.0015616,0.0

FLDR2,0.0,2.1604068606487394

FORt2pp,0.0,17.340213421318204

FORtex,0.0,112.91596926111079

FTHFLi,0.0,10.184974613660147

FUM,1.2333893150000121,7.362807680339482

G6PDH2r,0.7056018925000015,0.0

GAPD,3.5642330149999975,-0.48140233333136145

GART,0.0004906000000000834,7.155729407658059

GHMT2r,0.22309610000000604,-2.79771880600208

GLCptspp,2.1404179895833364,0.0

GLCtex_copy1,2.1404179895833364,0.0

GLNS,0.36212300000000835,14.670600615315669

GLUDy,-1.7103415000000157,-18.68507862131787

GLUPRT,0.08964760000000407,7.243886407658064

GLYAT,0.0,-10.164132813660142

GLYCL,0.010920900000001992,0.0

GND,0.7056018925000014,0.0

H2Otex,-9.914783537500057,-109.98774486113436

H2Otpp,-9.914783537500059,-109.98746406113399

HCO3E,0.10516900000000409,7.259407807657147

HSDy,-0.13962539999999996,-10.303758213660144

HSK,0.10884319999999999,10.272976013660143

Htex,-1.8376463999999728,111.07732286111238

ICDHyr,1.2398589150000043,0.2150384000000185

IMPC,-0.10855060000000409,-7.2627894076580635

IMPD,0.048743200000000084,7.2029820076580675

MALS,0.0001338000000000684,7.154372680339492

MDH,1.233197915000013,21.671919393987192

MOX,0.00032519999999967104,-7.154739033308219

MTHFC,0.1977522000000081,-2.83398360600208

MTHFD,0.1977522000000081,-2.83398360600208

NADH16pp,7.319156560000027,0.0

NTD10,0.0,7.154238807658068

O2tex,4.206049537500015,47.82127624341193

O2tpp,4.206049537500015,47.82113584341193

PDH,1.8733075150000043,0.0

PFK,1.2128609358333324,0.0

PFK_3,0.19573216416666564,0.0

PGCD,0.34363450000000595,0.0

PGI,1.434816097083335,0.0

PGK,-3.564233014999997,0.48140233333136145

PGL,0.7056018925000015,0.0

PGM,-3.2205985149999905,0.48140233333136145

POR5,0.021750999999999996,-2.1396558606487392

PPC,0.5918716000000012,3.6017655333206995

PPK,-0.7007176000000049,-7.854956407655865

PPM,-0.00013460000000000268,7.154104207658068

PPS,0.0,4.248955466651177

PRAGSr,0.08964760000000409,7.243886407658064

PRAIS,0.08964760000000409,7.243886407658064

PRASCSi,0.0896030000000041,7.243841807658064

PRFGS,0.08964760000000407,7.243886407658064

PRPPS,0.1865784000000047,7.340817207658064

PSERT,0.343634500000006,0.0

PSP_L,0.343634500000006,0.0

PUNP7,0.0,7.154238807658068

RPE,0.31519592833333127,-0.15520533333295383

RPI,-0.38244516416667035,-0.14724453333341217

SERD_L,0.0,2.67618040600208

SUCDi,1.024954315000004,0.0022676726814239156

SUCOAS,-0.9198485150000041,0.1039720000000482

TALA,0.0,-0.039468466666583366

THD2pp,0.0,44.93216971528773

THRD,0.0,10.164132813660142

THRS,0.1088432,10.272976013660143

TKT1,0.1957321641666656,-0.039468466666583366

TKT2,0.11946376416666554,-0.11573686666637047

TPI,1.7028532254166657,-0.20236413333249548

UGLYCH,0.0,7.154238807658068

URIC,0.0,7.154238807658068

XAND,0.0,7.154238807658068

99 reacctions require a flux change for the evolution of the for consumer

.......................................................

.......................................................

g3pe

.......................................................

ACALD,0.0,2.158276339784949

ACONTa,1.2398589150000043,1.5107759572970632

ACONTb,1.2398589150000041,1.5107759572970632

ATPS4rpp,13.370320970000053,13.002587690329973

CO2tex,-4.632830737500008,-2.581702841603844

CO2tpp,-4.632830737500008,-2.581702841603844

CS,1.2398589150000043,1.5107759572970634

CYTBO3_4pp,8.410403675000031,8.624702305247725

DHAPT,0.32252132541666795,0.0

ENO,3.220598515000006,1.3332400819948325

ETHAAL,0.0,2.158276339784949

ETHAt2pp,5.655907058495112e-35,2.158276339784949

F6PA,0.32252132541666795,0.0

FBA,1.2128609358333324,-0.1741029238437913

FBA3,0.19573216416666564,0.0

FBP,0.0,0.1741029238437913

FUM,1.2333893150000121,1.50430580535141

G3PD2,-0.027805600000000014,2.1304707397851392

G3PEtex,0.0,2.158276339784949

G6PDH2r,0.7056018925000015,0.0

GAPD,3.5642330149999975,1.6768738605385956

GLCptspp,2.1404179895833364,0.0

GLCtex_copy1,2.1404179895833364,0.0

GLYC3Pt6pp,1.6967721175485333e-34,2.158276339784949

GND,0.7056018925000014,0.0

GPDDA2pp,5.655907058495111e-35,2.158276339784949

H2Otex,-9.914783537500057,-5.705879924969581

H2Otpp,-9.914783537500059,-7.8636556969088565

Htex,-1.8376463999999728,-3.994919625652308

ICL,0.0,0.26991661865901106

MALS,0.0001338000000000684,0.270050418659011

MDH,1.233197915000013,1.774031025975991

NADH16pp,7.319156560000027,7.2606723640900075

NADTRHD,0.0,0.7470743744501651

NH4tex,2.160164800000022,0.0018880093819763296

NH4tpp,2.160164800000022,0.0018880093819763296

O2tex,4.206049537500015,4.313449136546699

O2tpp,4.206049537500015,4.313198852623862

PDH,1.8733075150000043,0.2568655104917823

PFK,1.2128609358333324,0.0

PFK_3,0.19573216416666564,0.0

PGI,1.434816097083335,0.0

PGK,-3.564233014999997,-1.6768738605385956

PGL,0.7056018925000015,0.0

PGM,-3.2205985149999905,-1.3332400819948325

PItex,0.19292620000000563,-1.9653502098531088

PPC,0.5918716000000012,0.32195530245321424

PYK,0.0,0.8454971795429825

RPE,0.31519592833333127,-0.155205361894516

RPI,-0.38244516416667035,-0.14724456189497434

SUCDi,1.024954315000004,1.2988709411577182

TALA,0.0,-0.039468480947364465

TKT1,0.1957321641666656,-0.039468480947364465

TKT2,0.11946376416666554,-0.11573688094715154

TPI,1.7028532254166657,1.9559122139757779

55 reacctions require a flux change for the evolution of the g3pe consumer

.......................................................

.......................................................

g3pg

.......................................................

ACONTa,1.2398589150000043,0.7660679928678956

ACONTb,1.2398589150000041,0.7660679928678956

AKGDH,1.024820515000004,0.5510295928678773

ATPS4rpp,13.370320970000053,12.53198060116206

CO2tex,-4.632830737500008,-2.5058560785970134

CO2tpp,-4.632830737500008,-2.5058560785970134

CS,1.2398589150000043,0.7660679928678956

CYTBO3_4pp,8.410403675000031,7.728298783395689

DHAPT,0.32252132541666795,0.1061536867725095

ENO,3.220598515000006,2.746807592859062

F6PA,0.32252132541666795,-1.5806960333324973

FUM,1.2333893150000121,0.7595983928673122

G3PD2,-0.027805600000000014,1.8571891060854269

G3PGtex,0.0,1.7859222130952168

G6PDH2r,0.7056018925000015,0.0

GAPD,3.5642330149999975,3.090442092859063

GLCptspp,2.1404179895833364,0.0

GLCtex_copy1,2.1404179895833364,0.0

GLYC3Pt6pp,1.6967721175485333e-34,1.78592221361555

GLYCDx,0.0,1.6868497201050068

GLYCtpp,1.9304563662165405e-18,-1.7859222130952168

GLYK,-1.93045636621654e-18,0.09907249246987691

GND,0.7056018925000014,0.0

GPDDA4pp,0.0,1.7859222130952168

H2Otex,-9.914783537500057,-7.788308878592038

H2Otpp,-9.914783537500059,-9.57373109272745

Htex,-1.8376463999999728,-3.622568613093802

ICDHyr,1.2398589150000043,0.7660679928678956

MDH,1.233197915000013,0.7594069928673122

NADH16pp,7.319156560000027,7.110842590527811

O2tex,4.206049537500015,3.8652470916978405

O2tpp,4.206049537500015,3.8649970916978447

PDH,1.8733075150000043,1.3995165928612883

PGI,1.434816097083335,0.0

PGK,-3.564233014999997,-3.090442092859063

PGL,0.7056018925000015,0.0

PGM,-3.2205985149999905,-2.746807592859062

PItex,0.19292620000000563,-1.5929960130956804

PYK,0.0,1.8829947060886707

RPE,0.31519592833333127,-0.15520533333295738

RPI,-0.38244516416667035,-0.14724453333341575

SUCDi,1.024954315000004,0.5511633928678775

SUCOAS,-0.9198485150000041,-0.4470575928684211

TALA,0.0,-0.2342006308332508

TKT1,0.1957321641666656,-0.039468466666585156

TKT2,0.11946376416666554,-0.11573686666637223

TPI,1.7028532254166657,3.3694802928579346

47 reacctions require a flux change for the evolution of the g3pg consumer

.......................................................

.......................................................

glcn

.......................................................

ACONTa,1.2398589150000043,1.1009276396645182

ACONTb,1.2398589150000041,1.1009276396645182

AKGDH,1.024820515000004,0.8848892396652298

ATPS4rpp,13.370320970000053,13.648182560673057

CO2tex,-4.632830737500008,-5.854460228168239

CO2tpp,-4.632830737500008,-5.854460228168239

CS,1.2398589150000043,1.1009276396645182

CYTBO3_4pp,8.410403675000031,8.509639751668875

ENO,3.220598515000006,3.0816672396498883

FBA,1.2128609358333324,0.8723247453765127

FUM,1.2333893150000121,1.0944580396637646

G6PDH2r,0.7056018925000015,2.3044991683471493e-06

GAPD,3.5642330149999975,3.4253017396494423

GLCNt2rpp,0.0,2.3440229046879275

GLCNtex,0.0,2.3440229046879275

GLCptspp,2.1404179895833364,0.0

GLCtex_copy1,2.1404179895833364,0.0

GND,0.7056018925000014,2.344025209187096

GNK,0.0,2.3440229046879275

H2Otex,-9.914783537500057,-11.136413028159762

H2Otpp,-9.914783537500059,-11.136413028159762

Htex,-1.8376463999999728,0.5063765046904823

ICDHyr,1.2398589150000043,1.0999276396652482

MDH,1.233197915000013,1.0953112396630347

NADH16pp,7.319156560000027,7.557323912004374

NADTRHD,0.0,0.7908936533667374

O2tex,4.206049537500015,4.255667575834437

O2tpp,4.206049537500015,4.255667575834437

PDH,1.8733075150000043,1.7363762396513163

PFK,1.2128609358333324,0.8723247453765127

PGI,1.434816097083335,-2.3044991683471493e-06

PGK,-3.564233014999997,-3.4253017396494423

PGL,0.7056018925000015,2.3044991683471493e-06

PGM,-3.2205985149999905,-3.0816672396498883

PYK,0.0,2.0034867142341035

RPE,0.31519592833333127,1.4074781394585565

RPI,-0.38244516416667035,-0.9285862697289976

SUCDi,1.024954315000004,0.8871568396644761

SUCOAS,-0.9198485150000041,-0.7799172396652052

TALA,0.0,0.5471411055625062

TKT1,0.1957321641666656,0.7418732697291718

TKT2,0.11946376416666554,0.6656048697293847

TPI,1.7028532254166657,1.3603170349602118

43 reacctions require a flux change for the evolution of the glcn consumer

.......................................................

.......................................................

glu__L

.......................................................

ACONTa,1.2398589150000043,0.19962601667838697

ACONTb,1.2398589150000041,0.19962601667838697

AKGDH,1.024820515000004,2.9899231666789357

ATPS4rpp,13.370320970000053,15.03308500011508

CO2tex,-4.632830737500008,-6.817000550029547

CO2tpp,-4.632830737500008,-6.817000550029547

CS,1.2398589150000043,0.19962601667838697

CYTBO3_4pp,8.410403675000031,9.773407750070476

DHAPT,0.32252132541666795,0.0

ENO,3.220598515000006,-0.8249699333309763

F6PA,0.32252132541666795,-0.0009999999999763531

FBA,1.2128609358333324,-0.1731029334783898

FBA3,0.19573216416666564,1.4596324149351858e-10

FBP,0.0,0.17310293347835076

FUM,1.2333893150000121,3.198491966678148

G6PDH2r,0.7056018925000015,0.0

GAPD,3.5642330149999975,-0.4814023333310615

GLCptspp,2.1404179895833364,0.0

GLCtex_copy1,2.1404179895833364,0.0

GLUDy,-1.7103415000000157,1.2939940500080969

GLUt2rpp,0.0,3.0053355500000407

GLUtex,0.0,3.00533555

GND,0.7056018925000014,0.0

H2Otex,-9.914783537500057,-6.088282250021848

H2Otpp,-9.914783537500059,-6.088282250021393

Htex,-1.8376463999999728,4.173024700002998

ICDHyr,1.2398589150000043,0.19962601667838697

MDH,1.233197915000013,1.7755941500065546

ME1,0.0,1.422572616671672

NADH16pp,7.319156560000027,6.7170579833915225

NADTRHD,0.0,0.5478988666903755

NH4tex,2.160164800000022,-0.8451707500043995

NH4tpp,2.160164800000022,-0.8451707500043995

O2tex,4.206049537500015,4.887551575035237

O2tpp,4.206049537500015,4.887551575035237

PDH,1.8733075150000043,0.8339408166717612

PFK,1.2128609358333324,0.0

PFK_3,0.19573216416666564,1.4596324149351858e-10

PGI,1.434816097083335,0.0

PGK,-3.564233014999997,0.4814023333310615

PGL,0.7056018925000015,0.0

PGM,-3.2205985149999905,0.8249699333309763

PPC,0.5918716000000012,0.0

PPCK,0.0,0.9907575333296306

RPE,0.31519592833333127,-0.15520533333290132

RPI,-0.38244516416667035,-0.14724453333326437

SUCDi,1.024954315000004,2.9900569666789356

SUCOAS,-0.9198485150000041,-2.8849511666788885

TALA,0.0,-0.03946846681253646

TKT1,0.1957321641666656,-0.03946846666656256

TKT2,0.11946376416666554,-0.11573686666633876

TPI,1.7028532254166657,-0.2023641333323667

52 reacctions require a flux change for the evolution of the glu__L consumer

.......................................................

.......................................................

gly

.......................................................

ACONTa,1.2398589150000043,1.9349304288897915

ACONTb,1.2398589150000041,1.9349304288897915

ADK1,0.524992600000009,1.171182533340978

AKGDH,1.024820515000004,1.1280204288910693

ATPS4rpp,13.370320970000053,17.19805257112239

CO2tex,-4.632830737500008,-10.754131448887392

CO2tpp,-4.632830737500008,-10.753640848887471

CS,1.2398589150000043,1.9349304288897915

CYTBO3_4pp,8.410403675000031,11.171100773339939

DHAPT,0.32252132541666795,0.0

ENO,3.220598515000006,-0.4814023333364048

F6PA,0.32252132541666795,0.0

FBA,1.2128609358333324,-0.17410293333375648

FBA3,0.19573216416666564,0.0

FBP,0.0,0.17410293333374566

FUM,1.2333893150000121,1.9284608288890168

G6PDH2r,0.7056018925000015,0.0

GAPD,3.5642330149999975,-0.4814023333364048

GHMT2r,0.22309610000000604,-4.51761076222292

GLCptspp,2.1404179895833364,0.0

GLCtex_copy1,2.1404179895833364,0.0

GLUDy,-1.7103415000000157,-1.3667070000020658

GLYCL,0.010920900000001992,4.75211836222273

GLYt2pp_copy1,0.0,9.480904324445646

GLYtex,0.0,9.481904324445646

GND,0.7056018925000014,0.0

H2Otex,-9.914783537500057,-6.554679924454831

H2Otpp,-9.914783537500059,-6.554179924454274

Htex,-1.8376463999999728,7.645257924444309

ICDHyr,1.2398589150000043,1.3430588288905483

ICL,0.0,0.591871599999271

MALS,0.0001338000000000684,0.5920053999992709

MDH,1.233197915000013,2.520185628888288

NADH16pp,7.319156560000027,9.383425344450167

NH4tex,2.160164800000022,-7.321739524444752

NH4tpp,2.160164800000022,-7.321739524444752

O2tex,4.206049537500015,5.586648086669965

O2tpp,4.206049537500015,5.586398086669969

PDH,1.8733075150000043,3.1602506288796013

PFK,1.2128609358333324,0.0

PFK_3,0.19573216416666564,0.0

PGCD,0.34363450000000595,0.0

PGI,1.434816097083335,0.0

PGK,-3.564233014999997,0.4814023333364048

PGL,0.7056018925000015,0.0

PGM,-3.2205985149999905,0.4814023333364048

PPC,0.5918716000000012,0.0

PPS,0.0,0.6471899333432105

PSERT,0.343634500000006,0.0

PSP_L,0.343634500000006,0.0

RPE,0.31519592833333127,-0.15520533333426556

RPI,-0.38244516416667035,-0.1472445333346286

SERD_L,0.0,4.39707236222311

SUCDi,1.024954315000004,1.722516428889773

SUCOAS,-0.9198485150000041,-1.0240484288909784

TALA,0.0,-0.03946846666519832

THD2pp,0.0,0.9638787711123664

TKT1,0.1957321641666656,-0.03946846666519832

TKT2,0.11946376416666554,-0.11573686666906724

TPI,1.7028532254166657,-0.20236413333350356

60 reacctions require a flux change for the evolution of the gly consumer

.......................................................

.......................................................

glyald

.......................................................

ACONTa,1.2398589150000043,1.4500382019991627

ACONTb,1.2398589150000041,1.4500382019991627

AKGDH,1.024820515000004,1.233999802001008

ALCD19,0.0,4.255814635460752

ATPS4rpp,13.370320970000053,12.949962395996938

CO2tex,-4.632830737500008,-4.557766706418354

CO2tpp,-4.632830737500008,-4.557766706418354

CS,1.2398589150000043,1.4500382019991627

CYTBO3_4pp,8.410403675000031,8.26027561285486

ENO,3.220598515000006,3.430777801986768

F6PA,0.32252132541666795,-1.580696033475797

FUM,1.2333893150000121,1.4435686019995027

G3PD2,-0.027805600000000014,2.325791676568477

G6PDH2r,0.7056018925000015,4.302898870545135e-10

GAPD,3.5642330149999975,3.774412301986467

GLCptspp,2.1404179895833364,0.0

GLCtex_copy1,2.1404179895833364,0.0

GLYALDtex,0.0,4.255814635460752

GLYALDtpp,0.0,4.255814635460752

GLYCDx,0.0,1.902217358892465

GLYK,-1.93045636621654e-18,2.3535972765682867

GND,0.7056018925000014,4.302898870545135e-10

H2Otex,-9.914783537500057,-9.840219506411026

H2Otpp,-9.914783537500059,-9.839719506410555

ICDHyr,1.2398589150000043,1.4490382020004593

MDH,1.233197915000013,1.4443772019982064

NADH16pp,7.319156560000027,6.958849210855149

NADTRHD,0.0,1.1515727794364763

O2tex,4.206049537500015,4.131235506427426

O2tpp,4.206049537500015,4.130985506427431

PDH,1.8733075150000043,2.084486801988214

PGI,1.434816097083335,-4.302898870545135e-10

PGK,-3.564233014999997,-3.774412301986467

PGL,0.7056018925000015,4.302898870545135e-10

PGM,-3.2205985149999905,-3.430777801986768

PYK,0.0,2.3525972765714633

RPE,0.31519592833333127,-0.1552053330459674

RPI,-0.38244516416667035,-0.14724453347671565

SUCDi,1.024954315000004,1.2351336019997115

SUCOAS,-0.9198485150000041,-1.1290278020009834

TALA,0.0,-0.2342006306897558

TKT1,0.1957321641666656,-0.03946846652309016

TKT2,0.11946376416666554,-0.11573686652287725

TPI,1.7028532254166657,4.053450501985143

44 reacctions require a flux change for the evolution of the glyald consumer

.......................................................

.......................................................

glyc

.......................................................

ACONTa,1.2398589150000043,0.898358527005894

ACONTb,1.2398589150000041,0.898358527005894

AKGDH,1.024820515000004,0.6823201270077699

ATPS4rpp,13.370320970000053,14.053321745985418

CO2tex,-4.632830737500008,-2.902727681524242

CO2tpp,-4.632830737500008,-2.902727681524242

CS,1.2398589150000043,0.898358527005894

CYTBO3_4pp,8.410403675000031,8.654332523565449

DHAPT,0.32252132541666795,0.35873475504725505

ENO,3.220598515000006,2.879098126990643

F6PA,0.32252132541666795,-1.5846960335053533

FUM,1.2333893150000121,0.8928889270096095

G3PD2,-0.027805600000000014,1.7328985719416

G6PDH2r,0.7056018925000015,5.189572660491137e-10

GAPD,3.5642330149999975,3.2227326269901755

GLCptspp,2.1404179895833364,0.0

GLCtex_copy1,2.1404179895833364,0.0

GLYCDx,0.0,1.9434307885526083

GLYCtex,0.0,3.704134960493843

GLYCtpp,1.9304563662165405e-18,-3.704134960493843

GLYK,-1.93045636621654e-18,1.7607041719412346

GND,0.7056018925000014,5.189572660491137e-10

H2Otex,-9.914783537500057,-11.889315442009133

H2Otpp,-9.914783537500059,-11.888815442009632

ICDHyr,1.2398589150000043,0.8973585270072211

MDH,1.233197915000013,0.893697527008283

NADH16pp,7.319156560000027,7.902585796559059

O2tex,4.206049537500015,4.328263961782355

O2tpp,4.206049537500015,4.328013961782724

PDH,1.8733075150000043,1.5338071269920954

PGI,1.434816097083335,-5.189572660491137e-10

PGK,-3.564233014999997,-3.2227326269901755

PGL,0.7056018925000015,5.189572660491137e-10

PGM,-3.2205985149999905,-2.879098126990643

PYK,0.0,1.7637041719447513

RPE,0.31519592833333127,-0.15520533298685663

RPI,-0.38244516416667035,-0.14724453350627215

SUCDi,1.024954315000004,0.6864539270063905

SUCOAS,-0.9198485150000041,-0.5773481270077699

TALA,0.0,-0.23620063066020042

TKT1,0.1957321641666656,-0.039468466493534776

TKT2,0.11946376416666554,-0.11573686649332186

TPI,1.7028532254166657,3.5017708269888526

43 reacctions require a flux change for the evolution of the glyc consumer

.......................................................

.......................................................

glyc__R

.......................................................

ACONTa,1.2398589150000043,2.7697707777988168

ACONTb,1.2398589150000041,2.7697707777988168

AKGDH,1.024820515000004,2.5537323778015626

ATPS4rpp,13.370320970000053,16.06014738909667

CO2tex,-4.632830737500008,-8.516964433385182

CO2tpp,-4.632830737500008,-8.516964433385182

CS,1.2398589150000043,2.7697707777988168

CYTBO3_4pp,8.410403675000031,10.603123855682616

DHAPT,0.32252132541666795,0.0

ENO,3.220598515000006,4.750510377779816

F6PA,0.32252132541666795,-0.001

FBA,1.2128609358333324,-0.17310353358661817

FBA3,0.19573216416666564,6.002550811956553e-07

FBP,0.0,0.17310353358661817

FUM,1.2333893150000121,2.763301177799275

G6PDH2r,0.7056018925000015,0.0

GAPD,3.5642330149999975,-0.4814023333290797

GLCptspp,2.1404179895833364,0.0

GLCtex_copy1,2.1404179895833364,0.0

GLYCAt2rpp,1.4997824761187406e-14,5.575547211108528

GLYCAtex,1.4997824761187406e-14,5.575547211108528

GLYCK,0.0,4.044635348328703

GLYCK2,1.4997824761187406e-14,1.530911862779825

GND,0.7056018925000014,0.0

H2Otex,-9.914783537500057,-13.798917233366877

H2Otpp,-9.914783537500059,-13.798917233366877

Htex,-1.8376463999999728,3.737900811115612

ICDHyr,1.2398589150000043,2.7687707778005333

MDH,1.233197915000013,2.7641097777975583

NADH16pp,7.319156560000027,7.97996487788277

NADTRHD,0.0,0.1127092778199037

O2tex,4.206049537500015,5.302409627841308

O2tpp,4.206049537500015,5.302409627841308

PDH,1.8733075150000043,3.4042193777836864

PFK,1.2128609358333324,0.0

PFK_3,0.19573216416666564,6.002550811956553e-07

PGI,1.434816097083335,0.0

PGK,-3.564233014999997,0.4814023333290797

PGL,0.7056018925000015,0.0

PYK,0.0,3.9938511777811785

RPE,0.31519592833333127,-0.15520533333295555

RPI,-0.38244516416667035,-0.1472445333334139

SUCDi,1.024954315000004,2.556866177799846

SUCOAS,-0.9198485150000041,-2.4487603778020426

TALA,0.0,-0.03946906692166544

TKT1,0.1957321641666656,-0.03946846666658425

TKT2,0.11946376416666554,-0.11573686666637131

TPI,1.7028532254166657,-0.2023641333311714

48 reacctions require a flux change for the evolution of the glyc__R consumer

.......................................................

.......................................................

glyc3p

.......................................................

ACONTa,1.2398589150000043,0.654907618274633

ACONTb,1.2398589150000041,0.654907618274633

AKGDH,1.024820515000004,0.4388689138994429

ATPS4rpp,13.370320970000053,11.253640406222342

CO2tex,-4.632830737500008,-2.172374732633925

CO2tpp,-4.632830737500008,-2.172374730681183

CS,1.2398589150000043,0.654907618274633

CYTBO3_4pp,8.410403675000031,6.950178116452395

DHAPT,0.32252132541666795,0.0

ENO,3.220598515000006,2.6356479673620408

F6PA,0.32252132541666795,-6.102397859445653e-10

FBA,1.2128609358333324,-0.17410296985220794

FBA3,0.19573216416666564,0.0

FBP,0.0,0.17410296985220794

FUM,1.2333893150000121,0.6484379187253034

G3PD2,-0.027805600000000014,3.4328788756992923

G6PDH2r,0.7056018925000015,0.0

GAPD,3.5642330149999975,2.979281999139448

GLCptspp,2.1404179895833364,0.0

GLCtex_copy1,2.1404179895833364,0.0

GLYC3Pt6pp,1.6967721175485333e-34,3.460684475699292

GLYC3Ptex,0.0,3.460684475862037

GND,0.7056018925000014,0.0

H2Otex,-9.914783537500057,-7.454328381642861

H2Otpp,-9.914783537500059,-7.454328382253101

ICDHyr,1.2398589150000043,0.6539074575777385

MDH,1.233197915000013,0.6492912800736151

NADH16pp,7.319156560000027,6.443882442954759

NADTRHD,0.0,1.460530885898836

O2tex,4.206049537500015,3.4759367582261973

O2tpp,4.206049537500015,3.4759367582261973

PDH,1.8733075150000043,1.2893563378801445

PFK,1.2128609358333324,0.0

PFK_3,0.19573216416666564,0.0

PGI,1.434816097083335,0.0

PGK,-3.564233014999997,-2.979281999139448

PGL,0.7056018925000015,0.0

PGM,-3.2205985149999905,-2.6356479673620408

PItex,0.19292620000000563,-3.2677581736314263

PYK,0.0,1.878988767363405

RPE,0.31519592833333127,-0.15520537266089135

RPI,-0.38244516416667035,-0.1472445726613497

SUCDi,1.024954315000004,0.4401366745963369

SUCOAS,-0.9198485150000041,-0.33489689871135775

TALA,0.0,-0.03946848633055214

TKT1,0.1957321641666656,-0.03946848633055214

TKT2,0.11946376416666554,-0.11573688633033921

TPI,1.7028532254166657,3.258320305195667

48 reacctions require a flux change for the evolution of the glyc3p consumer

.......................................................

.......................................................

glyclt

.......................................................

ACONTa,1.2398589150000043,1.9034599338888185

ACONTb,1.2398589150000041,1.9034599338888185

AKGDH,1.024820515000004,1.6884215338888184

ATPS4rpp,13.370320970000053,16.334587567188237

CO2tex,-4.632830737500008,-13.30620953001619

CO2tpp,-4.632830737500008,-13.30620953001619

CS,1.2398589150000043,1.9034599338888183

CYTBO3_4pp,8.410403675000031,14.99921789505585

DHAPT,0.32252132541666795,0.0

ENO,3.220598515000006,3.8841995338773714

F6PA,0.32252132541666795,0.0

FBA,1.2128609358333324,-0.17410366721446735

FBA3,0.19573216416666564,0.0

FBP,0.0,0.1741036672145101

FUM,1.2333893150000121,1.896990333888084

G6PDH2r,0.7056018925000015,2.2016464020224724e-06

GAPD,3.5642330149999975,-0.48140306721302295

GLCptspp,2.1404179895833364,0.0

GLCtex_copy1,2.1404179895833364,0.0

GLXCL,0.0,4.709237101090123

GLYCK,0.0,4.044636082212742

GLYCK2,1.4997824761187406e-14,0.6646010188773797

GLYCLTt2rpp,0.0,10.756943364991571

GLYCLTtex,0.0,10.757943364991547

GLYCTO2,0.0001338000000000684,10.75807716499157

GND,0.7056018925000014,2.2016464020224724e-06

H2Otex,-9.914783537500057,-18.58816233000914

H2Otpp,-9.914783537500059,-18.588162330008686

Htex,-1.8376463999999728,8.92029696499344

ICDHyr,1.2398589150000043,1.9034599338888185

MALS,0.0001338000000000684,1.3396029628113257

MDH,1.233197915000013,2.48867053388733

ME2,0.0,0.7475975628120973

NADH16pp,7.319156560000027,2.4864263961754594

O2tex,4.206049537500015,7.500456647527924

O2tpp,4.206049537500015,7.500456647527924

PDH,1.8733075150000043,3.876377696690735

PFK,1.2128609358333324,0.0

PFK_3,0.19573216416666564,0.0

PGI,1.434816097083335,-2.2016464527041535e-06

PGK,-3.564233014999997,0.48140306721302295

PGL,0.7056018925000015,2.2016464020224724e-06

PPC,0.5918716000000012,0.0

PYK,0.0,3.718411933878735

RPE,0.31519592833333127,-0.15520386556852372

RPI,-0.38244516416667035,-0.14724526721545317

SUCDi,1.024954315000004,1.6885553338888184

SUCOAS,-0.9198485150000041,-1.583449533889393

TALA,0.0,-0.039467732784373766

TKT1,0.1957321641666656,-0.039467732784373766

TKT2,0.11946376416666554,-0.11573613278414996

TPI,1.7028532254166657,-0.20236486721432811

TRSARr,0.0,4.7082371010901625

53 reacctions require a flux change for the evolution of the glyclt consumer

.......................................................

.......................................................

gthrd

.......................................................

ACKr,0.11651519999999993,0.016458766208643283

ACONTa,1.2398589150000043,1.2032518396921432

ACONTb,1.2398589150000041,1.2032518396921432

ACS,0.0,0.05048203379140857

ADSK,0.04957439999999991,0.0

AKGDH,1.024820515000004,2.5918774166468816

AMPTASECG,0.0,1.6036639769547392

ATPS4rpp,13.370320970000053,16.49229553416933

BPNT,0.049574399999999914,0.0

CGLYabcpp,0.0,1.6036639769547392

CO2tex,-4.632830737500008,-7.8270812961440015

CO2tpp,-4.632830737500008,-7.826590696143967

CS,1.2398589150000043,1.2032518396921426

CYSDS,0.0,1.5540903769547394

CYSS,0.04957439999999994,0.0

CYTBO3_4pp,8.410403675000031,10.18629312270091

DHAPT,0.32252132541666795,0.0

ENO,3.220598515000006,-0.4813125680140047

F6PA,0.32252132541666795,0.0

FBA,1.2128609358333324,-0.17410213333334923

FBA3,0.19573216416666564,0.0

FBP,0.0,0.17410213333332877

FUM,1.2333893150000121,2.8014454166468568

G6PDH2r,0.7056018925000015,0.0

GAPD,3.5642330149999975,-0.48137946801398357

GHMT2r,0.22309610000000604,-0.5784915884773909

GLCptspp,2.1404179895833364,0.0

GLCtex_copy1,2.1404179895833364,-0.0009999999999763531

GLUDy,-1.7103415000000157,0.23495817695470578

GLUt2rpp,0.0,1.6036639769547492

GLYCL,0.010920900000001992,0.8129975884773659

GND,0.7056018925000014,0.0

GTHRDHpp,0.0,1.6036639769547392

GTHRDtex,0.0,1.6036639769547492

H2Otex,-9.914783537500057,-1.8833558952941303

H2Otpp,-9.914783537500059,-3.4870217690961454

H2St1pp,0.0,1.5540903769547394

H2Stex,0.0,-1.5540903769547185

Htex,-1.8376463999999728,4.477860114124496

ICDHyr,1.2398589150000043,1.2032518396921432

MDH,1.233197915000013,2.4375614077061982

ME1,0.0,0.36455880894071946

NADH16pp,7.319156560000027,7.526632306054029

NADTRHD,0.0,0.6933203472854877

NDPK1,0.158125000000004,0.1095501999999442

NH4tex,2.160164800000022,-2.6508299308642336

NH4tpp,2.160164800000022,-2.6508299308642336

O2tex,4.206049537500015,5.093994261350417

O2tpp,4.206049537500015,5.093994261350417

PAPSR,0.049574399999999914,0.0

PDH,1.8733075150000043,1.8375654396921426

PFK,1.2128609358333324,0.0

PFK_3,0.19573216416666564,0.0

PGCD,0.34363450000000595,0.0

PGI,1.434816097083335,0.0

PGK,-3.564233014999997,0.48137946801398357

PGL,0.7056018925000015,0.0

PGM,-3.2205985149999905,0.48137946801398357

PPC,0.5918716000000012,0.0

PPCK,0.0,0.6471001680140189

PSERT,0.343634500000006,0.0

PSP_L,0.343634500000006,0.0

PTAr,-0.11651519999999993,-0.016458766208643283

RPE,0.31519592833333127,-0.15520453333328987

RPI,-0.38244516416667035,-0.14724373333331187

SADT2,0.04957439999999991,0.0

SERAT,0.04957439999999993,0.0

SERD_L,0.0,0.5075275884773804

SO4t2pp,0.05044199999999991,0.0008676000000000001

SO4tex,0.05044199999999991,0.0008675999999923079

SUCDi,1.024954315000004,2.594011216646882

SUCOAS,-0.9198485150000041,-2.4869058166468676

SULR,0.049574399999999914,0.0

TALA,0.0,-0.0394680666667

TKT1,0.1957321641666656,-0.0394680666667

TKT2,0.11946376416666554,-0.11573646666670356

TPI,1.7028532254166657,-0.20236333333332368

TRDR,0.049574399999999914,0.0009791346806966848

78 reacctions require a flux change for the evolution of the gthrd consumer

.......................................................

.......................................................

gua

.......................................................

ACONTa,1.2398589150000043,0.21503839999921104

ACONTb,1.2398589150000041,0.21503839999921104

ADK1,0.524992600000009,0.47838400000072856

ADSL2r,0.08960300000000408,0.0

AICART,0.10855060000000409,0.01894759999981943

AIRC2,0.08960300000000408,0.0

AIRC3,-0.08960300000000408,0.0

AKGDH,1.024820515000004,0.0

ALLTAMH,0.0,5.93873924414369

ALLTN,0.0,5.93873924414369

ASPTA,-0.5855358000000094,-0.49593279999930906

ATPS4rpp,13.370320970000053,17.691000489321596

CO2tex,-4.632830737500008,-21.932034020757953

CO2tpp,-4.632830737500008,-21.864272820636234

CS,1.2398589150000043,0.21503839999921104

CYTBO3_4pp,8.410403675000031,6.838756776667558

DHAPT,0.32252132541666795,0.0

ENO,3.220598515000006,2.1957779999852205

F6PA,0.32252132541666795,0.0

FBA,1.2128609358333324,-0.17410293333259363

FBA3,0.19573216416666564,0.0

FBP,0.0,0.17410293333259363

FDH4pp,0.0,0.06776120012172009

FORtppi,0.0,0.06776120012172009

FTHFD,0.0,0.06727060012172016

FUM,1.2333893150000121,0.11896580000029644

G6PDH2r,0.7056018925000015,0.0

GAPD,3.5642330149999975,-0.4814023333316718

GARFT,0.08915700000000398,4.46e-05

GHMT2r,0.22309610000000604,0.1225722000607698

GLCptspp,2.1404179895833364,0.0

GLCtex_copy1,2.1404179895833364,0.0

GLNS,0.36212300000000835,0.134173799998881

GLUDy,-1.7103415000000157,-1.5202146000576287

GLUPRT,0.08964760000000407,4.46e-05

GLXCL,0.0,2.920290933377487

GLYCK,0.0,2.920290933377487

GLYCL,0.010920900000001992,6.076977809854611e-11

GMPR,0.0,0.04085980000104428

GMPS2,0.048743200000000084,0.0

GND,0.7056018925000014,0.0

GUAD,0.0,5.93873924414369

GUAPRT,0.0,0.08960300000104393

GUAtex,0.0,6.028342244144734

GUAtpp,0.0,6.028342244144734

H2Otex,-9.914783537500057,33.069435620688246

H2Otpp,-9.914783537500059,33.06943562068873

HCO3E,0.10516900000000409,0.015565999999083334

Htex,-1.8376463999999728,28.304064820724818

ICDHyr,1.2398589150000043,0.21503839999921104

IMPC,-0.10855060000000409,-0.01894759999981943

IMPD,0.048743200000000084,0.0

MALS,0.0001338000000000684,0.09829117738877358

MDH,1.233197915000013,6.15567102153276

MOX,0.00032519999999967104,-5.93841404414369

MTHFC,0.1977522000000081,0.08630740012153959

MTHFD,0.1977522000000081,0.08630740012153959

NADH16pp,7.319156560000027,6.704568976545838

NH4tex,2.160164800000022,-27.981546420724115

NH4tpp,2.160164800000022,-27.981546420724115

O2tex,4.206049537500015,3.420226088333779

O2tpp,4.206049537500015,3.420226088333779

PDH,1.8733075150000043,0.9466443773780956

PFK,1.2128609358333324,0.0

PFK_3,0.19573216416666564,0.0

PGCD,0.34363450000000595,0.2431106000605947

PGI,1.434816097083335,0.0

PGK,-3.564233014999997,0.4814023333316718

PGL,0.7056018925000015,0.0

PGM,-3.2205985149999905,-2.1957779999852205

PPC,0.5918716000000012,0.4937142226094501

PPK,-0.7007176000000049,-0.6541089999965422

PRAGSr,0.08964760000000409,4.46e-05

PRAIS,0.08964760000000409,4.46e-05

PRASCSi,0.0896030000000041,0.0

PRFGS,0.08964760000000407,4.46e-05

PSERT,0.343634500000006,0.2431106000605947

PSP_L,0.343634500000006,0.2431106000605947

PYK,0.0,1.536276177377052

RPE,0.31519592833333127,-0.15520533333353212

RPI,-0.38244516416667035,-0.14724453333399048

SUCDi,1.024954315000004,0.00013380000000000336

SUCOAS,-0.9198485150000041,0.1049719999997845

TALA,0.0,-0.039468466666872516

THD2pp,0.0,2.398201999933235

TKT1,0.1957321641666656,-0.039468466666872516

TKT2,0.11946376416666554,-0.1157368666666596

TPI,1.7028532254166657,-0.20236413333241854

TRSARr,0.0,2.920290933377487

UGLYCH,0.0,5.93873924414369

URIC,0.0,5.93873924414369

XAND,0.0,5.93873924414369

92 reacctions require a flux change for the evolution of the gua consumer

.......................................................

.......................................................

hxa

.......................................................

ACACT1r,0.07117599999999999,-2.331305340076673

ACACT2r,0.07117599999999998,-2.331305340076673

ACOAD1f,-0.07117599999999998,2.331305340076673

ACOAD2f,-0.07117599999999999,2.331305340076673

ACONTa,1.2398589150000043,4.4016675306561455

ACONTb,1.2398589150000041,4.4016675306561455

ADK1,0.524992600000009,2.927473940074186

AKGDH,1.024820515000004,2.604933041077698

ATPS4rpp,13.370320970000053,17.821555761272407

CO2tex,-4.632830737500008,-6.205210688607508

CO2tpp,-4.632830737500008,-6.205210688607508

CS,1.2398589150000043,4.4016675306561455

CYTBO3_4pp,8.410403675000031,12.429519560342255

DHAPT,0.32252132541666795,0.0

ECOAH1,0.07117599999999999,-2.331305340076673

ECOAH2,0.07117599999999998,-2.331305340076673

ENO,3.220598515000006,-0.825036855830831

F6PA,0.32252132541666795,0.0

FACOAL60t2pp,0.0,2.4014813400754065

FADRx,0.437178,0.0

FBA,1.2128609358333324,-0.17410293895750328

FBA3,0.19573216416666564,0.0

FBP,0.0,0.17410293895745232

FE3Ri,0.0,4.367784680157497

FESD1s,0.0007808,4.368565480157497

FESR,0.0015616,8.737130960314994

FLDR2,0.0,0.5906318000002762

FUM,1.2333893150000121,4.39519793065616

G6PDH2r,0.7056018925000015,0.0

GAPD,3.5642330149999975,-0.48140235583105095

GLCptspp,2.1404179895833364,0.0

GLCtex_copy1,2.1404179895833364,0.0

GND,0.7056018925000014,0.0

H2Otex,-9.914783537500057,-11.487663572976658

H2Otpp,-9.914783537500059,-11.487163572976215

HACD1,0.07117599999999999,-2.331305340076673

HACD2,0.07117599999999998,-2.331305340076673

HXAtex,0.0,2.4024813400754064

Htex,-1.8376463999999728,0.565834923201237

ICDHyr,1.2398589150000043,2.8199714410771306

ICL,0.0,1.5816960895790149

MALS,0.0001338000000000684,1.5818298895790148

MDH,1.233197915000013,1.6089625400776981

MOX,0.00032519999999967104,4.368065280157497

NADH16pp,7.319156560000027,8.176463829686108

O2tex,4.206049537500015,10.58364216032862

O2tpp,4.206049537500015,10.583392160328625

PDH,1.8733075150000043,0.0

PFK,1.2128609358333324,0.0

PFK_3,0.19573216416666564,0.0

PGI,1.434816097083335,0.0

PGK,-3.564233014999997,0.48140235583105095

PGL,0.7056018925000015,0.0

PGM,-3.2205985149999905,0.825036855830831

POR5,0.021750999999999996,-0.5688808000003291

PPC,0.5918716000000012,0.0

PPCK,0.0,0.9898244895791973

PPK,-0.7007176000000049,-3.1021989400720953

RPE,0.31519592833333127,-0.15520533895789868

RPI,-0.38244516416667035,-0.14724453895837541

SUCDi,1.024954315000004,4.186762930656146

SUCOAS,-0.9198485150000041,-2.499961041077654

TALA,0.0,-0.03946846104167889

THD2pp,0.0,0.4217230926697533

TKT1,0.1957321641666656,-0.03946846104167889

TKT2,0.11946376416666554,-0.11573687791621978

TPI,1.7028532254166657,-0.20236413895725036

67 reacctions require a flux change for the evolution of the hxa consumer

.......................................................

.......................................................

hxan

.......................................................

ACALD,0.0,0.32741740430492033

ACONTa,1.2398589150000043,0.21503839999943258

ACONTb,1.2398589150000041,0.21503839999943258

ADSL2r,0.08960300000000408,0.0

AICART,0.10855060000000409,0.01894759999981943

AIRC2,0.08960300000000408,0.0

AIRC3,-0.08960300000000408,0.0

AKGDH,1.024820515000004,0.0

ALLTAMH,0.0,5.183613258170378

ALLTN,0.0,5.183613258170378

ASAD,-0.21381520000000004,-0.542232604304921

ASPK,0.2138152,0.542232604304921

ASPTA,-0.5855358000000094,-0.8243502043041744

ATPS4rpp,13.370320970000053,18.89006648373254

CO2tex,-4.632830737500008,-18.156404090878

CO2tpp,-4.632830737500008,-18.156404090878

CS,1.2398589150000043,0.21503839999943258

CYTBO3_4pp,8.410403675000031,9.091469090914876

DHAPT,0.32252132541666795,0.0

ENO,3.220598515000006,2.110471195754127

F6PA,0.32252132541666795,0.0

FBA,1.2128609358333324,-0.17410293333236382

FBA3,0.19573216416666564,0.0

FBP,0.0,0.17410293333237567

FTHFLi,0.0,0.25965620416874924

FUM,1.2333893150000121,0.11896579999924967

G6PDH2r,0.7056018925000015,0.0

GAPD,3.5642330149999975,-0.4814023333310622

GARFT,0.08915700000000398,-0.0014460000000000835

GHMT2r,0.22309610000000604,-0.20584520423691544

GLCptspp,2.1404179895833364,0.0

GLCtex_copy1,2.1404179895833364,0.0

GLNS,0.36212300000000835,0.1829169999993604

GLUDy,-1.7103415000000157,-1.605521404302479

GLUPRT,0.08964760000000407,4.46e-05

GLXCL,0.0,2.591873529085189

GLYCK2,1.4997824761187406e-14,2.5908735290852127

GLYCL,0.010920900000001992,6.798339668989684e-11

GND,0.7056018925000014,0.0

H2Otex,-9.914783537500057,24.020589432662405

H2Otpp,-9.914783537500059,24.02058943266286

HCO3E,0.10516900000000409,0.015565999999083334

HSDy,-0.13962539999999996,-0.4680428043048965

HSK,0.10884319999999999,0.4372606043048967

HXAND,0.0,5.183613258170378

HXPRT,0.0,0.08960299999999677

HYXNtex,0.0,5.2732162581703745

HYXNtpp,0.0,5.2732162581703745

Htex,-1.8376463999999728,19.255218632683977

ICDHyr,1.2398589150000043,0.21503839999943258

IMPC,-0.10855060000000409,-0.01894759999981943

MDH,1.233197915000013,5.302253858169626

MOX,0.00032519999999967104,-5.183288058170377

MTHFC,0.1977522000000081,-0.24211000416892858

MTHFD,0.1977522000000081,-0.24211000416892858

NADH16pp,7.319156560000027,9.025042490914874

NH4tex,2.160164800000022,-18.932700232685136

NH4tpp,2.160164800000022,-18.932700232685136

O2tex,4.206049537500015,4.546582245457438

O2tpp,4.206049537500015,4.546582245457438

PDH,1.8733075150000043,0.2592795915191791

PFK,1.2128609358333324,0.0

PFK_3,0.19573216416666564,0.0

PFL,0.0,0.26065620416874935

PGCD,0.34363450000000595,0.0

PGI,1.434816097083335,0.0

PGK,-3.564233014999997,0.4814023333310622

PGL,0.7056018925000015,0.0

PGM,-3.2205985149999905,0.4804023333310856

PPC,0.5918716000000012,0.920422804304354

PRAGSr,0.08964760000000409,4.46e-05

PRAIS,0.08964760000000409,4.46e-05

PRASCSi,0.0896030000000041,0.0

PRFGS,0.08964760000000407,4.46e-05

PSERT,0.343634500000006,0.0

PSP_L,0.343634500000006,0.0

PYK,0.0,1.0242607914511384

RPE,0.31519592833333127,-0.15520533333290132

RPI,-0.38244516416667035,-0.14724453333326437

SERD_L,0.0,0.08430680423691322

SUCDi,1.024954315000004,0.00013380000000000336

SUCOAS,-0.9198485150000041,0.10497200000002456

TALA,0.0,-0.03946846666656256

THD2pp,0.0,3.427901217079449

THRA,0.0,0.3274174043049204

THRS,0.1088432,0.4372606043048967

TKT1,0.1957321641666656,-0.03946846666656256

TKT2,0.11946376416666554,-0.11573686666633876

TPI,1.7028532254166657,-0.2023641333323667

TRSARr,0.0,2.591873529085189

UGLYCH,0.0,5.183613258170378

URIC,0.0,5.183613258170378

XAND,0.0,5.183613258170378

93 reacctions require a flux change for the evolution of the hxan consumer

.......................................................

.......................................................

idon__L

.......................................................

5DGLCNR,0.0,2.3618405402476133

ACONTa,1.2398589150000043,0.8265368205224016

ACONTb,1.2398589150000041,0.8265368205224016

AKGDH,1.024820515000004,0.6114984205234305

ATPS4rpp,13.370320970000053,14.196965158949052

CO2tex,-4.632830737500008,-5.961366041540295

CO2tpp,-4.632830737500008,-5.961366041540295

CS,1.2398589150000043,0.8265368205224016

CYTBO3_4pp,8.410403675000031,8.705633742848562

ENO,3.220598515000006,2.8072764205066325

FBA,1.2128609358333324,0.580116290672638

FUM,1.2333893150000121,0.8200672205232276

G6PDH2r,0.7056018925000015,0.9122629397334927

GAPD,3.5642330149999975,3.150910920506247

GLCptspp,2.1404179895833364,0.0

GLCtex_copy1,2.1404179895833364,0.0

GND,0.7056018925000014,3.2741034799811057

GNK,0.0,2.3618405402476133

H2Otex,-9.914783537500057,-11.24331884152254

H2Otpp,-9.914783537500059,-11.24331884152254

Htex,-1.8376463999999728,0.5241941402537369

ICDHyr,1.2398589150000043,0.8265368205224016

IDOND,0.0,-2.361840540247613

IDONt2rpp,0.0,2.361840540247613

IDONtex,0.0,2.361840540247613

MDH,1.233197915000013,0.8199204196627403

NADH16pp,7.319156560000027,8.025708721464898

O2tex,4.206049537500015,4.3536645714242805

O2tpp,4.206049537500015,4.3536645714242805

PDH,1.8733075150000043,1.4599854205122393

PFK,1.2128609358333324,0.580116290672638

PGI,1.434816097083335,-0.9122629397334927

PGK,-3.564233014999997,-3.150910920506247

PGL,0.7056018925000015,0.9122629397334927

PGM,-3.2205985149999905,-2.8072764205066325

PYK,0.0,1.7280958950938166

RPE,0.31519592833333127,2.0275303199880463

RPI,-0.38244516416667035,-1.2386123599935184

SUCDi,1.024954315000004,0.6146322213836628

SUCOAS,-0.9198485150000041,-0.5075264205239106

TALA,0.0,0.8571671958272509

TKT1,0.1957321641666656,1.0518993599939166

TKT2,0.11946376416666554,0.9756309599941295

TPI,1.7028532254166657,1.0681085802561463

44 reacctions require a flux change for the evolution of the idon__L consumer

.......................................................

.......................................................

ins

.......................................................

ACONTa,1.2398589150000043,0.21472739999803792

ACONTb,1.2398589150000041,0.21472739999803792

ADSL2r,0.08960300000000408,0.00011762131848438732

AICART,0.10855060000000409,0.019065221318303818

AIRC2,0.08960300000000408,0.00011762131848438732

AIRC3,-0.08960300000000408,-0.00011762131848438732

AKGDH,1.024820515000004,0.0

ALLTAMH,0.0,1.5477710132307934

ALLTN,0.0,1.5477710132307934

ASPTA,-0.5855358000000094,-0.4970014213176819

CO2tex,-4.632830737500008,-8.165471719177903

CO2tpp,-4.632830737500008,-8.098219434424951

CS,1.2398589150000043,0.21472739999803792

CYTBO3_4pp,8.410403675000031,7.289750165533659

ENO,3.220598515000006,2.1940310716657603

FBA,1.2128609358333324,0.002253053383577397

FDH4pp,0.0,0.06725228475295254

FORtppi,0.0,0.06725228475295254

FTHFD,0.0,0.0667616847529527

FUM,1.2333893150000121,0.1190364213177304

G6PDH2r,0.7056018925000015,1.182775154934876

GAPD,3.5642330149999975,1.8526319348826767

GARFT,0.08915700000000398,0.00016222131848438732

GHMT2r,0.22309610000000604,0.12194957435205356

GLCptspp,2.1404179895833364,0.0

GLCtex_copy1,2.1404179895833364,0.0

GLNS,0.36212300000000835,0.1831522426370062

GLUDy,-1.7103415000000157,-1.5213949239803939

GLUPRT,0.08964760000000407,0.00016222131848438732

GLXCL,0.0,0.5838866394558202

GLYCK,0.0,0.5828866394558438

GLYCL,0.010920900000001992,0.0002593530357070106

GND,0.7056018925000014,1.1817751549348996

H2Otex,-9.914783537500057,2.926062999958671

H2Otpp,-9.914783537500059,2.926562999959196

HCO3E,0.10516900000000409,0.01568242131756772

HXAND,0.0,1.5477710132307934

HXPRT,0.0,0.08848697868177395

Htex,-1.8376463999999728,4.712933054357283

ICDHyr,1.2398589150000043,0.21472739999803792

IMPC,-0.10855060000000409,-0.019065221318303818

INSt2pp_copy1,0.0,1.6362093919118714

INStex,0.0,1.6372093919123025

MALS,0.0001338000000000684,0.38013153431915225

MDH,1.233197915000013,2.0456121688676996

MOX,0.00032519999999967104,-1.5474442132307933

MTHFC,0.1977522000000081,0.08603372738974091

MTHFD,0.1977522000000081,0.08603372738974091

NADH16pp,7.319156560000027,7.15607128078073

NH4tex,2.160164800000022,-4.388998167658084

NH4tpp,2.160164800000022,-4.388998167658084

O2tex,4.206049537500015,3.6459504827668296

O2tpp,4.206049537500015,3.64570048276683

PDH,1.8733075150000043,1.2281725343091872

PFK,1.2128609358333324,0.002253053383577397

PGCD,0.34363450000000595,0.2424875026727369

PGI,1.434816097083335,-1.182775154934876

PGK,-3.564233014999997,-1.8526319348826767

PGL,0.7056018925000015,1.182775154934876

PGM,-3.2205985149999905,-2.193031071665784

PPC,0.5918716000000012,0.21456086567878918

PPM,-0.00013460000000000268,1.6351671919726571

PRAGSr,0.08964760000000409,0.00016222131848438732

PRAIS,0.08964760000000409,0.00016222131848438732

PRASCSi,0.0896030000000041,0.00011762131848438732

PRFGS,0.08964760000000407,0.00016222131848438732

PSERT,0.343634500000006,0.2424875026727369

PSP_L,0.343634500000006,0.2424875026727369

PUNP5,0.0,1.6362579919125673

PYK,0.0,1.4901612805716673

RPE,0.31519592833333127,1.7241792979012962

RPI,-0.38244516416667035,0.5503649429659383

SUCDi,1.024954315000004,0.00026759999999999284

SUCOAS,-0.9198485150000041,0.10497239999804414

TALA,0.0,0.7034916847839086

TKT1,0.1957321641666656,0.9002238489505416

TKT2,0.11946376416666554,0.8239554489507546

TPI,1.7028532254166657,0.4942469429670536

TRSARr,0.0,0.5838866394558202

UGLYCH,0.0,1.5477710132307934

URIC,0.0,1.5477710132307934

XAND,0.0,1.5477710132307934

82 reacctions require a flux change for the evolution of the ins consumer

.......................................................

.......................................................

lac__D

.......................................................

ACONTa,1.2398589150000043,2.022244419476178

ACONTb,1.2398589150000041,2.022244419476178

ADK1,0.524992600000009,1.5028961338616351

AKGDH,1.024820515000004,1.2153344194769136

ATPS4rpp,13.370320970000053,17.79849818050132

CO2tex,-4.632830737500008,-6.241622660020868

CO2tpp,-4.632830737500008,-6.241622660020868

CS,1.2398589150000043,2.022244419476178

CYTBO3_4pp,8.410403675000031,11.627987520056742

D_LACt2pp,0.0,4.817099953329375

D_LACtex,0.0,4.817099953329375

DHAPT,0.32252132541666795,0.0

ENO,3.220598515000006,-0.8141159338648309

F6PA,0.32252132541666795,0.0

FBA,1.2128609358333324,-0.17410293333249977

FBA3,0.19573216416666564,0.0

FBP,0.0,0.17410293333249977

FTHFLi,0.0,0.02084179893238838

FUM,1.2333893150000121,2.015774819475597

G6PDH2r,0.7056018925000015,0.0

GAPD,3.5642330149999975,-0.4814023333311967

GLCptspp,2.1404179895833364,0.0

GLCtex_copy1,2.1404179895833364,0.0

GLYCL,0.010920900000001992,5.338069153371094e-10

GND,0.7056018925000014,0.0

H2Otex,-9.914783537500057,-11.523575460013921

H2Otpp,-9.914783537500059,-11.523575460013442

Htex,-1.8376463999999728,2.9794535533317763

ICDHyr,1.2398589150000043,1.4303728194769318

ICL,0.0,0.5918715999992461

LDH_D,0.0,4.817099953328895

MALS,0.0001338000000000684,0.5920053999992461

MDH,1.233197915000013,2.607455019474843

MTHFC,0.1977522000000081,0.17591040106761582

MTHFD,0.1977522000000081,0.17591040106761582

NADH16pp,7.319156560000027,9.752354900580581

O2tex,4.206049537500015,5.81484146002837

O2tpp,4.206049537500015,5.81484146002837

PDH,1.8733075150000043,3.225722820533627

PFK,1.2128609358333324,0.0

PFK_3,0.19573216416666564,0.0

PFL,0.0,0.021841798932388457

PGCD,0.34363450000000595,0.3327136005336342

PGI,1.434816097083335,0.0

PGK,-3.564233014999997,0.4814023333311967

PGL,0.7056018925000015,0.0

PGM,-3.2205985149999905,0.8141159338648309

PPC,0.5918716000000012,0.0

PPS,0.0,0.9799035338634665

PSERT,0.343634500000006,0.3327136005336342

PSP_L,0.343634500000006,0.3327136005336342

RPE,0.31519592833333127,-0.15520533333295808

RPI,-0.38244516416667035,-0.14724453333341642

SUCDi,1.024954315000004,1.8093398194761596

SUCOAS,-0.9198485150000041,-1.1103624194769135

TALA,0.0,-0.03946846666658551

THD2pp,0.0,1.2316107799834723

TKT1,0.1957321641666656,-0.03946846666658551

TKT2,0.11946376416666554,-0.11573686666637258

TPI,1.7028532254166657,-0.20236413333232445

60 reacctions require a flux change for the evolution of the lac__D consumer

.......................................................

.......................................................

lac__L

.......................................................

ACONTa,1.2398589150000043,2.3559462574728993

ACONTb,1.2398589150000041,2.3559462574728993

ADK1,0.524992600000009,1.5120410821018129

AKGDH,1.024820515000004,1.549659857479575

ATPS4rpp,13.370320970000053,17.462026451777504

CO2tex,-4.632830737500008,-7.27340961965621

CO2tpp,-4.632830737500008,-7.27340961965621

CS,1.2398589150000043,2.3559462574728993

CYTBO3_4pp,8.410403675000031,13.691622615685322

DHAPT,0.32252132541666795,0.0

ENO,3.220598515000006,-0.8237584533502655

F6PA,0.32252132541666795,0.0

FBA,1.2128609358333324,-0.17404059369912994

FBA3,0.19573216416666564,0.0

FBP,0.0,0.17404059369912994

FUM,1.2333893150000121,2.3496922385783297

G6PDH2r,0.7056018925000015,0.0

GAPD,3.5642330149999975,-0.4811238842479475

GLCptspp,2.1404179895833364,0.0

GLCtex_copy1,2.1404179895833364,0.0

GLYCL,0.010920900000001992,0.009968609447897764

GND,0.7056018925000014,0.0

H2Otex,-9.914783537500057,-12.55396030626838

H2Otpp,-9.914783537500059,-12.55346030626814

Htex,-1.8376463999999728,3.3241074773560584

ICDHyr,1.2398589150000043,1.7643872574776132

ICL,0.0,0.5915589999952859

L_LACD2,0.0,5.159008241708061

L_LACt2rpp,0.0,5.160008241708061

L_LACtex,0.0,5.160008241708061

MALS,0.0001338000000000684,0.591692799995286

MDH,1.233197915000013,2.941103053822794

MTHFC,0.1977522000000081,0.19588950944988398

MTHFD,0.1977522000000081,0.19588950944988398

NADH16pp,7.319156560000027,6.323968916502402

O2tex,4.206049537500015,6.8469091152563815

O2tpp,4.206049537500015,6.846659115256381

PDH,1.8733075150000043,3.579306385807426

PFK,1.2128609358333324,0.0

PFK_3,0.19573216416666564,0.0

PFL,0.0,0.001767271654152025

PGCD,0.34363450000000595,0.342634569102318

PGI,1.434816097083335,0.0

PGK,-3.564233014999997,0.4811238842479475

PGL,0.7056018925000015,0.0

PGM,-3.2205985149999905,0.8237584533502655

PPC,0.5918716000000012,0.0

PPS,0.0,0.9895460533489012

PSERT,0.343634500000006,0.342634569102318

PSP_L,0.343634500000006,0.342634569102318

RPE,0.31519592833333127,-0.15514299369958828

RPI,-0.38244516416667035,-0.14718219370004665

SUCDi,1.024954315000004,2.1424864574748606

SUCOAS,-0.9198485150000041,-1.4456874574815317

TALA,0.0,-0.03943729684990059

THD2pp,0.0,0.8867425905663059

TKT1,0.1957321641666656,-0.03943729684990059

TKT2,0.11946376416666554,-0.11570569684968768

TPI,1.7028532254166657,-0.20230019369912988

59 reacctions require a flux change for the evolution of the lac__L consumer

.......................................................

.......................................................

mal__L

.......................................................

ACONTa,1.2398589150000043,1.8071310783126593

ACONTb,1.2398589150000041,1.8071310783126593

AKGDH,1.024820515000004,1.5920926783136882

ATPS4rpp,13.370320970000053,16.430915488551868

CO2tex,-4.632830737500008,-10.24168524656188

CO2tpp,-4.632830737500008,-10.24168524656188

CS,1.2398589150000043,1.8071310783126593

CYTBO3_4pp,8.410403675000031,10.402431469877161

DHAPT,0.32252132541666795,0.0

ENO,3.220598515000006,-0.8249699333294573

F6PA,0.32252132541666795,-0.001

FBA,1.2128609358333324,-0.17310293355245848

FBA3,0.19573216416666564,2.2091839468885155e-10

FBP,0.0,0.17310293355245848

FUM,1.2333893150000121,1.8006614783131267

G6PDH2r,0.7056018925000015,0.0

GAPD,3.5642330149999975,-0.48140233332945304

GLCptspp,2.1404179895833364,0.0

GLCtex_copy1,2.1404179895833364,0.0

GND,0.7056018925000014,0.0

H2Otex,-9.914783537500057,-10.91079743491611

H2Otpp,-9.914783537500059,-10.91079743491611

Htex,-1.8376463999999728,7.388034823264729

ICDHyr,1.2398589150000043,1.8071310783126593

MALt2_2pp,0.0,4.612840611628395

MALtex,0.0,4.612840611628395

MDH,1.233197915000013,3.3830992116389385

ME1,0.0,2.181146056630809

ME2,0.0,0.848931621671774

NADH16pp,7.319156560000027,8.741912191563472

O2tex,4.206049537500015,5.20206343493858

O2tpp,4.206049537500015,5.20206343493858

PDH,1.8733075150000043,2.4404458783050913

PFK,1.2128609358333324,0.0

PFK_3,0.19573216416666564,2.2091839468885155e-10

PGI,1.434816097083335,0.0

PGK,-3.564233014999997,0.48140233332945304

PGL,0.7056018925000015,0.0

PGM,-3.2205985149999905,0.8249699333294573

PPC,0.5918716000000012,0.0

PPCK,0.0,0.9907575333280931

RPE,0.31519592833333127,-0.15520533333295866

RPI,-0.38244516416667035,-0.14724453333341705

SUCDi,1.024954315000004,1.5942264783136884

SUCOAS,-0.9198485150000041,-1.4871206783141684

TALA,0.0,-0.03946846688750418

TKT1,0.1957321641666656,-0.039468466666585794

TKT2,0.11946376416666554,-0.11573686666637287

TPI,1.7028532254166657,-0.20236413333154007

49 reacctions require a flux change for the evolution of the mal__L consumer

.......................................................

.......................................................

orn

.......................................................

ABTA,0.0,2.6023529784577035

ABUTD,0.0,2.6023529784577035

ACALD,0.0,0.29490447277125376

ACGK,0.05915840000000002,0.0

ACGS,0.05915840000000002,0.0

ACKr,0.11651519999999993,0.057359081779850385

ACODA,0.059158400000000014,0.0

ACONTa,1.2398589150000043,0.15452320000000097

ACONTb,1.2398589150000041,0.15452320000000097

ACOTA,-0.05915840000000002,-0.0

AGPR,-0.05915840000000003,0.0

AKGDH,1.024820515000004,0.0

ASAD,-0.21381520000000004,-0.5107200727691179

ASPK,0.2138152,0.5107200727691179

ASPT,0.0,5.321533146347608

ASPTA,-0.5855358000000094,-6.204869707549619

ATPS4rpp,13.370320970000053,18.5030200632242

CO2tex,-4.632830737500008,-5.106204250162023

CO2tpp,-4.632830737500008,-5.106204250162023

CS,1.2398589150000043,0.15452320000000097

CYTBO3_4pp,8.410403675000031,12.01974347275982

DHAPT,0.32252132541666795,0.0

ENO,3.220598515000006,-0.5277326602487165

F6PA,0.32252132541666795,-0.001

FBA,1.2128609358333324,-0.17303836280877777

FBA3,0.19573216416666564,0.0

FBP,0.0,0.17303836280877777

FTHFLi,0.0,0.2968148727690927

FUM,1.2333893150000121,8.13435281323829

G6PDH2r,0.7056018925000015,0.0

GAPD,3.5642330149999975,-0.4811183070220295

GHMT2r,0.22309610000000604,-0.0738594285546722

GLCptspp,2.1404179895833364,0.0

GLCtex_copy1,2.1404179895833364,0.0

GND,0.7056018925000014,0.0

H2Otex,-9.914783537500057,-5.061098974697757

H2Otpp,-9.914783537500059,-5.061923645541356

HSDy,-0.13962539999999996,-0.43653027276911793

HSK,0.10884319999999999,0.4057476727710476

Htex,-1.8376463999999728,0.8256718173458921

ICDHyr,1.2398589150000043,0.15452320000000097

MDH,1.233197915000013,7.054587967796957

ME2,0.0,1.0804396454413339

MTHFC,0.1977522000000081,-0.10011372855268963

MTHFD,0.1977522000000081,-0.10011372855268963

NADH16pp,7.319156560000027,9.349963894302116

NH4tex,2.160164800000022,-3.165295235835856

NH4tpp,2.160164800000022,-3.165295235835856

O2tex,4.206049537500015,6.010718344865329

O2tpp,4.206049537500015,6.01071943637991

ORNDC,0.0,2.602398778455723

ORNtex,0.0,2.662557178455723

PDH,1.8733075150000043,0.1952895102366622

PFK,1.2128609358333324,0.0

PFK_3,0.19573216416666564,0.0

PFL,0.0,0.29676381698566834

PGCD,0.34363450000000595,0.046681253226598246

PGI,1.434816097083335,0.0

PGK,-3.564233014999997,0.4811183070220295

PGL,0.7056018925000015,0.0

PGM,-3.2205985149999905,0.5277995602487164

PPC,0.5918716000000012,0.0

PPCK,0.0,0.6935202602473014

PSERT,0.343634500000006,0.046681253226598246

PSP_L,0.343634500000006,0.046681253226598246

PTAr,-0.11651519999999993,-0.057359081779850385

PTRCORNt7pp,0.0,-2.662557178455723

PTRCTA,0.0,2.6023529784577035

PTRCt2pp,0.0,2.662557178455723

RPE,0.31519592833333127,-0.15514076280931022

RPI,-0.38244516416667035,-0.14717996280967327

SSALx,0.0,0.3542069226848974

SSALy,0.0,2.248146055772806

SUCDi,1.024954315000004,2.6044867784577037

SUCOAS,-0.9198485150000041,0.10597239999799513

TALA,0.0,-0.03943618140476701

THRA,0.0,0.29490447277125376

THRS,0.1088432,0.4057476727710476

TKT1,0.1957321641666656,-0.03943618140476701

TKT2,0.11946376416666554,-0.1157045814045432

TPI,1.7028532254166657,-0.2022979628087569

81 reacctions require a flux change for the evolution of the orn consumer

.......................................................

.......................................................

pro__L

.......................................................

ACONTa,1.2398589150000043,0.13923473509032647

ACONTb,1.2398589150000041,0.13923473509032647

AKGDH,1.024820515000004,2.869363355090272

ATPS4rpp,13.370320970000053,15.109278191285398

CO2tex,-4.632830737500008,-6.516216171059

CO2tpp,-4.632830737500008,-6.515725571058965

CS,1.2398589150000043,0.13923473509027248

CYTBO3_4pp,8.410403675000031,10.194823018965229

DHAPT,0.32252132541666795,0.0

ENO,3.220598515000006,-0.8252026591214872

F6PA,0.32252132541666795,-0.0009999999999763531

FADRx,0.437178,0.0

FBA,1.2128609358333324,-0.17310213333337288

FBA3,0.19573216416666564,0.0

FBP,0.0,0.17310213333333221

FE3Ri,0.0,2.4637780200000003

FESD1s,0.0007808,2.46455882

FESR,0.0015616,4.92911764

FUM,1.2333893150000121,3.0789325550903186

G5SADs,0.044211,0.0

G5SD,0.044211,0.0

G6PDH2r,0.7056018925000015,0.0

GAPD,3.5642330149999975,-0.48139155912156184

GLCptspp,2.1404179895833364,0.0

GLCtex_copy1,2.1404179895833364,-0.0009999999999763531

GLU5K,0.04421099999999999,0.0

GLUDy,-1.7103415000000157,1.2325820199999953

GND,0.7056018925000014,0.0

H2Otex,-9.914783537500057,-5.907817360363765

H2Otpp,-9.914783537500059,-5.907818556847246

Htex,-1.8376463999999728,1.1075202258609806

ICDHyr,1.2398589150000043,0.13923473509032647

MDH,1.233197915000013,-0.746342425788157

ME1,0.0,1.3621717608784718

MOX,0.00032519999999967104,2.4631032199999936

NADH16pp,7.319156560000027,7.258542463874957

NADTRHD,0.0,0.5160305035138643

NH4tex,2.160164800000022,-0.7850050199999714

NH4tpp,2.160164800000022,-0.7850050199999714

O2tex,4.206049537500015,7.562037229482598

O2tpp,4.206049537500015,7.562037229482598

P5CD,0.0,2.9009560200000006

P5CR,0.044211,0.0

PDH,1.8733075150000043,0.7735483350902725

PFK,1.2128609358333324,0.0

PFK_3,0.19573216416666564,0.0

PGI,1.434816097083335,0.0

PGK,-3.564233014999997,0.48139155912156184

PGL,0.7056018925000015,0.0

PGM,-3.2205985149999905,0.8252026591214872

PPC,0.5918716000000012,0.0

PPCK,0.0,0.9909902591215277

PROD2,0.0,2.9009560200000006

PROt2rpp,0.0,2.9451670199999853

PROtex,0.0,2.9451670199999853

RPE,0.31519592833333127,-0.15520453333328987

RPI,-0.38244516416667035,-0.14724373333331187

SUCDi,1.024954315000004,2.8706309550902724

SUCOAS,-0.9198485150000041,-2.764391755090287

TALA,0.0,-0.0394680666667

TKT1,0.1957321641666656,-0.0394680666667

TKT2,0.11946376416666554,-0.11573646666670356

TPI,1.7028532254166657,-0.20236333333332368

63 reacctions require a flux change for the evolution of the pro__L consumer

.......................................................

.......................................................

ptrc

.......................................................

ABTA,0.0,2.709259116463296

ABUTD,0.0,2.7082591164632963

ACALD,0.0,0.30780226407822864

ACONTa,1.2398589150000043,0.21468160000000136

ACONTb,1.2398589150000041,0.21468160000000136

AKGDH,1.024820515000004,0.0

ASAD,-0.21381520000000004,-0.5236178640762482

ASPK,0.2138152,0.5236178640762482

ASPT,0.0,5.4160281464785225

ASPTA,-0.5855358000000094,-6.311263880401244

ATPS4rpp,13.370320970000053,18.64564202889576

CO2tex,-4.632830737500008,-2.6306311166535625

CO2tpp,-4.632830737500008,-2.6306311166535625

CS,1.2398589150000043,0.21468160000000136

CYTBO3_4pp,8.410403675000031,12.533960057058826

DHAPT,0.32252132541666795,0.0

ENO,3.220598515000006,-0.5143215415418028

F6PA,0.32252132541666795,0.0

FBA,1.2128609358333324,-0.17452411070653376

FBA3,0.19573216416666564,0.0

FBP,0.0,0.17452411070653376

FTHFLi,0.0,0.3107126640762485

FUM,1.2333893150000121,8.333755332788291

G6PDH2r,0.7056018925000015,0.0014558622781172075

GAPD,3.5642330149999975,-0.48160474562739847

GHMT2r,0.22309610000000604,-0.08775652915425178

GLCptspp,2.1404179895833364,0.0

GLCtex_copy1,2.1404179895833364,0.0

GND,0.7056018925000014,0.0014558622781172075

H2Otex,-9.914783537500057,-5.20146464826237

H2Otpp,-9.914783537500059,-5.201345019269787

HSDy,-0.13962539999999996,-0.44942806407624825

HSK,0.10884319999999999,0.41864546407822867

ICDHyr,1.2398589150000043,0.21468160000000136

MDH,1.233197915000013,7.206727821942164

ME2,0.0,1.1287007108461276

MTHFC,0.1977522000000081,-0.11401082915226955

MTHFD,0.1977522000000081,-0.11401082915226955

NADH16pp,7.319156560000027,9.756274340595528

NH4tex,2.160164800000022,-3.2587872583095594

NH4tpp,2.160164800000022,-3.2587872583095594

O2tex,4.206049537500015,6.2678875430257035

O2tpp,4.206049537500015,6.267827728529412

PDH,1.8733075150000043,0.22665163691494483

PFK,1.2128609358333324,0.0

PFK_3,0.19573216416666564,0.0

PFL,0.0,0.3116622990002196

PGCD,0.34363450000000595,0.03278369591440447

PGI,1.434816097083335,-0.0014558622781172075

PGK,-3.564233014999997,0.48160474562739847

PGL,0.7056018925000015,0.0014558622781172075

PGM,-3.2205985149999905,0.5143884415418027

PPC,0.5918716000000012,0.0

PPCK,0.0,0.6791091415409187

PSERT,0.343634500000006,0.03278369591440447

PSP_L,0.343634500000006,0.03278369591440447

PTRCTA,0.0,2.7082591164632963

PTRCt2pp,0.0,2.7093049164613157

PTRCtex,0.0,2.7093049164613157

RPE,0.31519592833333127,-0.15417064842887487

RPI,-0.38244516416667035,-0.14766571070745055

SSALx,0.0,0.47759499428901486

SSALy,0.0,2.2316641221742812

SUCDi,1.024954315000004,2.7123929164632963

SUCOAS,-0.9198485150000041,0.10497239999654281

TALA,0.0,-0.03895112421454391

THRA,0.0,0.30880226407822864

THRS,0.1088432,0.41864546407822867

TKT1,0.1957321641666656,-0.03895112421454391

TKT2,0.11946376416666554,-0.11521952421433097

TPI,1.7028532254166657,-0.20278371070653373

71 reacctions require a flux change for the evolution of the ptrc consumer

.......................................................

.......................................................

pyr

.......................................................

ACONTa,1.2398589150000043,2.909604936934877

ACONTb,1.2398589150000041,2.909604936934877

ADK1,0.524992600000009,1.5028961341265514

AKGDH,1.024820515000004,2.102694936936722

ATPS4rpp,13.370320970000053,16.91113766303899

CO2tex,-4.632830737500008,-8.90370421319498

CO2tpp,-4.632830737500008,-8.90370421319498

CS,1.2398589150000043,2.909604936934877

CYTBO3_4pp,8.410403675000031,11.247690155353096

DHAPT,0.32252132541666795,0.0

ENO,3.220598515000006,-0.8141159341303298

F6PA,0.32252132541666795,0.0

FBA,1.2128609358333324,-0.1741029333323772

FBA3,0.19573216416666564,0.0

FBP,0.0,0.1741029333323772

FTHFLi,0.0,0.020841798400526055

FUM,1.2333893150000121,2.9031353369346755

G6PDH2r,0.7056018925000015,0.0

GAPD,3.5642330149999975,-0.48140233333087534

GLCptspp,2.1404179895833364,0.0

GLCtex_copy1,2.1404179895833364,0.0

GLYCL,0.010920900000001992,7.996459605585926e-10

GND,0.7056018925000014,0.0

H2Otex,-9.914783537500057,-8.481196542133878

H2Otpp,-9.914783537500059,-8.481196542133638

Htex,-1.8376463999999728,3.8668140710561505

ICDHyr,1.2398589150000043,2.3177333369361732

ICL,0.0,0.5918715999987039

MALS,0.0001338000000000684,0.5920053999987038

MDH,1.233197915000013,3.4948155369333795

MTHFC,0.1977522000000081,0.17591040159929064

MTHFD,0.1977522000000081,0.17591040159929064

NADH16pp,7.319156560000027,8.48469701841767

O2tex,4.206049537500015,5.624692777676548

O2tpp,4.206049537500015,5.624692777676548

PDH,1.8733075150000043,4.113083338523403

PFK,1.2128609358333324,0.0

PFK_3,0.19573216416666564,0.0

PFL,0.0,0.021841798400526188

PGCD,0.34363450000000595,0.33271360079945445

PGI,1.434816097083335,0.0

PGK,-3.564233014999997,0.48140233333087534

PGL,0.7056018925000015,0.0

PGM,-3.2205985149999905,0.8141159341303298

PPC,0.5918716000000012,0.0

PPS,0.0,0.9799035341289656

PSERT,0.343634500000006,0.33271360079945445

PSP_L,0.343634500000006,0.33271360079945445

PYRt2rpp,0.0,5.704460471052812

PYRtex,0.0,5.704460471052812

RPE,0.31519592833333127,-0.1552053333328355

RPI,-0.38244516416667035,-0.1472445333332939

SUCDi,1.024954315000004,2.696700336935426

SUCOAS,-0.9198485150000041,-1.9977229369366976

TALA,0.0,-0.039468466666524225

THD2pp,0.0,0.3442502622569563

TKT1,0.1957321641666656,-0.039468466666524225

TKT2,0.11946376416666554,-0.11573686666631129

TPI,1.7028532254166657,-0.20236413333218692

59 reacctions require a flux change for the evolution of the pyr consumer

.......................................................

.......................................................

ser__L

.......................................................

ACONTa,1.2398589150000043,2.8082647340319005

ACONTb,1.2398589150000041,2.8082647340319005

ADK1,0.524992600000009,1.1741820699858068

AKGDH,1.024820515000004,2.001355116279014

ATPS4rpp,13.370320970000053,16.324804834132898

CO2tex,-4.632830737500008,-8.633182584055081

CO2tpp,-4.632830737500008,-8.632691984055082

CS,1.2398589150000043,2.8082647340319005

CYTBO3_4pp,8.410403675000031,10.796864396471976

DHAPT,0.32252132541666795,0.0

ENO,3.220598515000006,-0.48140188901353265

F6PA,0.32252132541666795,-2.767430729022635e-10

FBA,1.2128609358333324,-0.17410278005307805

FBA3,0.19573216416666564,0.0

FBP,0.0,0.17410278005307805

FUM,1.2333893150000121,2.801794947417823

G6PDH2r,0.7056018925000015,0.0

GAPD,3.5642330149999975,-0.48140188901353265

GLCptspp,2.1404179895833364,0.0

GLCtex_copy1,2.1404179895833364,0.0

GLUDy,-1.7103415000000157,-1.3667060313204407

GND,0.7056018925000014,0.0

H2Otex,-9.914783537500057,-8.301346511284692

H2Otpp,-9.914783537500059,-8.30084651110019

Htex,-1.8376463999999728,3.7776382088860307

ICDHyr,1.2398589150000043,2.216393336390066

ICL,0.0,0.5918713976418345

MALS,0.0001338000000000684,0.5920051976418345

MDH,1.233197915000013,3.393474944252985

NADH16pp,7.319156560000027,8.13572068282811

NH4tex,2.160164800000022,-3.4541216865112645

NH4tpp,2.160164800000022,-3.4541216865112645

O2tex,4.206049537500015,5.399507598328238

O2tpp,4.206049537500015,5.399257598235987

PDH,1.8733075150000043,4.033584792594216

PFK,1.2128609358333324,0.0

PFK_3,0.19573216416666564,0.0

PGCD,0.34363450000000595,0.0

PGI,1.434816097083335,5.534901162008197e-10

PGK,-3.564233014999997,0.48140188901353265

PGL,0.7056018925000015,0.0

PGM,-3.2205985149999905,0.48140188901353265

PPC,0.5918716000000012,2.767421319106106e-10

PPS,0.0,0.6471894890121683

PSERT,0.343634500000006,0.0

PSP_L,0.343634500000006,0.0

RPE,0.31519592833333127,-0.15520518088472982

RPI,-0.38244516416667035,-0.14724438088518815

SERD_L,0.0,5.269405585401825

SERt2rpp,0.0,5.614285455264304

SERtex,0.0,5.614285455263622

SUCDi,1.024954315000004,2.5953603136438654

SUCOAS,-0.9198485150000041,-1.8973831391687952

TALA,0.0,-0.03946839044247137

THD2pp,0.0,0.09054488980787817

TKT1,0.1957321641666656,-0.03946839044247137

TKT2,0.11946376416666554,-0.11573679044225844

TPI,1.7028532254166657,-0.2023639792460405

58 reacctions require a flux change for the evolution of the ser__L consumer

.......................................................

.......................................................

succ

.......................................................

ACONTa,1.2398589150000043,1.4145488986024426

ACONTb,1.2398589150000041,1.4145488986024426

AKGDH,1.024820515000004,1.1995104986034715

ATPS4rpp,13.370320970000053,16.823497668292184

CO2tex,-4.632830737500008,-8.671356527715538

CO2tpp,-4.632830737500008,-8.671356527715538

CS,1.2398589150000043,1.4145488986024426

CYTBO3_4pp,8.410403675000031,12.267196823530973

DHAPT,0.32252132541666795,0.0

ENO,3.220598515000006,-0.8249699333290657

F6PA,0.32252132541666795,-0.001

FBA,1.2128609358333324,-0.17310333358388813

FBA3,0.19573216416666564,4.002523503743305e-07

FBP,0.0,0.17310333358388813

FUM,1.2333893150000121,5.628337730518267

G6PDH2r,0.7056018925000015,0.0

GAPD,3.5642330149999975,-0.48140233332925697

GLCptspp,2.1404179895833364,0.0

GLCtex_copy1,2.1404179895833364,0.0

GND,0.7056018925000014,0.0

H2Otex,-9.914783537500057,-9.733050895782425

H2Otpp,-9.914783537500059,-9.733050895782425

Htex,-1.8376463999999728,6.602870463838503

ICDHyr,1.2398589150000043,1.4145488986024426

MDH,1.233197915000013,2.9905170319283476

ME1,0.0,1.3969824972104978

ME2,0.0,1.2405130013794212

NADH16pp,7.319156560000027,6.781001293012137

O2tex,4.206049537500015,6.134446111765486

O2tpp,4.206049537500015,6.134446111765486

PDH,1.8733075150000043,2.047863698592021

PFK,1.2128609358333324,0.0

PFK_3,0.19573216416666564,4.002523503743305e-07

PGI,1.434816097083335,0.0

PGK,-3.564233014999997,0.48140233332925697

PGL,0.7056018925000015,0.0

PGM,-3.2205985149999905,0.8249699333290657

PPC,0.5918716000000012,0.0

PPCK,0.0,0.9907575333277012

RPE,0.31519592833333127,-0.15520533333295633

RPI,-0.38244516416667035,-0.14724453333341464

SUCCt2_2pp,0.0,4.220258431915364

SUCCtex,0.0,4.220258431915364

SUCDi,1.024954315000004,5.419902730518836

SUCOAS,-0.9198485150000041,-1.094538498603927

TALA,0.0,-0.03946886691893501

TKT1,0.1957321641666656,-0.03946846666658462

TKT2,0.11946376416666554,-0.1157368666663717

TPI,1.7028532254166657,-0.20236413333134748

49 reacctions require a flux change for the evolution of the succ consumer

.......................................................

.......................................................

thr__L

.......................................................

ACALD,0.0,3.3822022231406828

ACONTa,1.2398589150000043,2.3634597740291285

ACONTb,1.2398589150000041,2.3634597740291285

ADK1,0.524992600000009,1.187135317956809

AKGDH,1.024820515000004,1.6660165740350077

ASAD,-0.21381520000000004,-0.10497239999804414

ASPK,0.2138152,0.10497239999804414

ASPTA,-0.5855358000000094,-0.47659699999785937

ATPS4rpp,13.370320970000053,16.492181776712915

CO2tex,-4.632830737500008,-5.765559492612061

CO2tpp,-4.632830737500008,-5.765068892612061

CS,1.2398589150000043,2.3634597740291285

CYTBO3_4pp,8.410403675000031,10.676427731557919

DHAPT,0.32252132541666795,0.0

ENO,3.220598515000006,-0.4976186606949514

F6PA,0.32252132541666795,-0.0010000000000000009

FBA,1.2128609358333324,-0.1895346606982437

FBA3,0.19573216416666564,0.0

FBP,0.0,0.1895346606982437

FUM,1.2333893150000121,2.357207174030905

G6PDH2r,0.7056018925000015,0.04948078210156204

GAPD,3.5642330149999975,-0.4976186606949514

GHMT2r,0.22309610000000604,-1.4688750115714249

GLCptspp,2.1404179895833364,0.0

GLCtex_copy1,2.1404179895833364,0.0

GLUDy,-1.7103415000000157,-1.2585029999885675

GLYCL,0.010920900000001992,1.7031990115692595

GND,0.7056018925000014,0.04948078210156204

H2Otex,-9.914783537500057,-7.552073784894282

H2Otpp,-9.914783537500059,-7.552073784894282

HSDy,-0.13962539999999996,-0.030782599998019577

HSK,0.10884319999999999,0.0

Htex,-1.8376463999999728,1.6551421157438138

ICDHyr,1.2398589150000043,1.8807439740330458

ICL,0.0,0.4827157999960827

MALS,0.0001338000000000684,0.48284959999608273

MDH,1.233197915000013,2.840229458592896

NADH16pp,7.319156560000027,8.460778157526828

NH4tex,2.160164800000022,-1.3332060231479703

NH4tpp,2.160164800000022,-1.3332060231479703

O2tex,4.206049537500015,5.338562081213052

O2tpp,4.206049537500015,5.338562081213052

PDH,1.8733075150000043,0.09554175087792088

PFK,1.2128609358333324,0.0

PFK_3,0.19573216416666564,0.0

PGCD,0.34363450000000595,0.0

PGI,1.434816097083335,-0.04948078210156204

PGK,-3.564233014999997,0.4976186606949514

PGL,0.7056018925000015,0.04948078210156204

PGM,-3.2205985149999905,0.4976186606949514

PPC,0.5918716000000012,0.0

PPS,0.0,0.6634062606940672

PSERT,0.343634500000006,0.0

PSP_L,0.343634500000006,0.0

RPE,0.31519592833333127,-0.12215627859711731

RPI,-0.38244516416667035,-0.1636762606991624

SERD_L,0.0,1.3473371270077246

SUCDi,1.024954315000004,2.1488661740310904

SUCOAS,-0.9198485150000041,-1.56204417403694

TALA,0.0,-0.022943939298670557

THRA,0.0,3.3832022231406826

THRS,0.1088432,0.0

THRt2rpp,0.0,3.4930454231404764

THRtex,0.0,3.4930454231404764

TKT1,0.1957321641666656,-0.022943939298670557

TKT2,0.11946376416666554,-0.09921233929844675

TPI,1.7028532254166657,-0.21879426069824381

67 reacctions require a flux change for the evolution of the thr__L consumer

.......................................................

.......................................................

thymd

.......................................................

ACALD,0.0,2.3658247223481097

ACONTa,1.2398589150000043,1.8033365524805791

ACONTb,1.2398589150000041,1.8033365524805791

AKGDH,1.024820515000004,1.5882981524816082

ASPCT,0.06615899999999995,0.060925800000000016

ATPS4rpp,13.370320970000053,12.537384163722589

CO2tex,-4.632830737500008,-3.6717784117982872

CO2tpp,-4.632830737500008,-3.671775529429731

CS,1.2398589150000043,1.8033365524805791

CYTBO3_4pp,8.410403675000031,8.848890545965967

DHAPT,0.32252132541666795,0.0

DHFR,0.005367000000000067,0.00013379999999996173

DHORD2,0.06615899999999995,0.060925800000000016

DHORTS,-0.06615899999999995,-0.060925800000000016

DRPA,0.0,2.3658247223481097

DUTPDP,0.0052332,0.0

ENO,3.220598515000006,1.4130182301140914

F6PA,0.32252132541666795,0.0

FBA,1.2128609358333324,-0.30972095105256525

FBA3,0.19573216416666564,0.0

FBP,0.0,0.30972095105256525

FUM,1.2333893150000121,1.7968669524816032

G6PDH2r,0.7056018925000015,0.4173204531630851

GAPD,3.5642330149999975,1.7540375712980012

GLCptspp,2.1404179895833364,0.0

GLCtex_copy1,2.1404179895833364,0.0

GLYCL,0.010920900000001992,0.008305741184277071

GND,0.7056018925000014,0.4173204531630851

H2Otex,-9.914783537500057,-6.556507289433805

H2Otpp,-9.914783537500059,-6.556507289433805

ICDHyr,1.2398589150000043,1.8033365524805791

MDH,1.233197915000013,1.7966755524816036

NADH16pp,7.319156560000027,7.199396111115803

NDPK2,0.08505659999999997,0.07982339999858144

O2tex,4.206049537500015,4.425292972982984

O2tpp,4.206049537500015,4.425292972982984

OMPDC,0.06615899999999997,0.060925800000000016

ORPT,-0.06615899999999995,-0.060925800000000016

PDH,1.8733075150000043,0.07619363011924718

PFK,1.2128609358333324,0.0

PFK_3,0.19573216416666564,0.0

PGCD,0.34363450000000595,0.34101934118390964

PGI,1.434816097083335,-0.4173204531630851

PGK,-3.564233014999997,-1.7540375712980012

PGL,0.7056018925000015,0.4173204531630851

PGM,-3.2205985149999905,-1.4130182301140914

PPC,0.5918716000000012,0.5866383999983101

PPM2,0.0,2.3658247223481097

PSERT,0.343634500000006,0.34101934118390964

PSP_L,0.343634500000006,0.34101934118390964

PYK,0.0,0.6605922301171459

RNTR4c2,0.0052331999999999995,0.0

RPE,0.31519592833333127,0.12649710210910128

RPI,-0.38244516416667035,-0.28286255105444214

SUCDi,1.024954315000004,1.5884319524816082

SUCOAS,-0.9198485150000041,-1.4833261524820638

TALA,0.0,0.10138275105444416

THMDt2pp_copy1,0.0,2.37005792234811

THMDtex,0.0,2.37105792234811

THYMt3pp,0.0,2.3658247223481097

THYMtex,0.0,-2.3658247223481097

TKT1,0.1957321641666656,0.10138275105444416

TKT2,0.11946376416666554,0.02511435105465713

TMDPP,0.0,2.3658247223481097

TMDS,0.0052331999999999995,0.0

TPI,1.7028532254166657,-0.3379821510522003

66 reacctions require a flux change for the evolution of the thymd consumer

.......................................................

.......................................................

trp__L

.......................................................

ACONTa,1.2398589150000043,2.7423485352071753

ACONTb,1.2398589150000041,2.7423485352071753

ADK1,0.524992600000009,1.446187734031918

AKGDH,1.024820515000004,1.9354385352083092

ANPRT,0.011368599999999998,0.0

ANS,0.011368599999999998,0.0

ATPS4rpp,13.370320970000053,16.93060226477118

CHORS,0.07622380000000006,0.06485519999978703

CO2tex,-4.632830737500008,-8.390566407724647

CO2tpp,-4.632830737500008,-8.390566407724647

CS,1.2398589150000043,2.7423485352071753

CYTBO3_4pp,8.410403675000031,10.445513946234394

DDPA,0.07622380000000009,0.06485519999978703

DHAPT,0.32252132541666795,0.0

DHQS,0.07622380000000006,0.06485519999978703

DHQTi,0.07622380000000006,0.06485519999978703

ENO,3.220598515000006,-0.7913787340350984

F6PA,0.32252132541666795,0.0

FBA,1.2128609358333324,-0.1627343333325033

FBA3,0.19573216416666564,0.0

FBP,0.0,0.1627343333325033

FTHFLi,0.0,0.020841798591886673

FUM,1.2333893150000121,2.73587893520719

G6PDH2r,0.7056018925000015,0.0

GAPD,3.5642330149999975,-0.4586651333312059

GLCptspp,2.1404179895833364,0.0

GLCtex_copy1,2.1404179895833364,0.0

GLNS,0.36212300000000835,0.35075439999955615

GLYCL,0.010920900000001992,7.040602545416164e-10

GND,0.7056018925000014,0.0

H2Otex,-9.914783537500057,-2.598111069255869

H2Otpp,-9.914783537500059,-2.5981110692556286

Htex,-1.8376463999999728,3.676820469232861

ICDHyr,1.2398589150000043,2.150476935207742

ICL,0.0,0.5918715999994331

IGPS,0.0113686,0.0

INDOLEt2rpp,0.0,-5.4907296692310315

INDOLEtex,0.0,-5.491729669231032

MALS,0.0001338000000000684,0.592005399999433

MDH,1.233197915000013,3.327559135206623

MTHFC,0.1977522000000081,0.17591040140812783

MTHFD,0.1977522000000081,0.17591040140812783

NADH16pp,7.319156560000027,7.84977721102665

NH4tex,2.160164800000022,-3.3543020692343006

NH4tpp,2.160164800000022,-3.3543020692343006

O2tex,4.206049537500015,5.223604673117197

O2tpp,4.206049537500015,5.223604673117197

PDH,1.8733075150000043,3.9458269366053123

PFK,1.2128609358333324,0.0

PFK_3,0.19573216416666564,0.0

PFL,0.0,0.021841798591886844

PGCD,0.34363450000000595,0.33271360070389255

PGI,1.434816097083335,0.0

PGK,-3.564233014999997,0.4586651333312059

PGL,0.7056018925000015,0.0

PGM,-3.2205985149999905,0.7913787340350984

PPC,0.5918716000000012,0.0

PPS,0.0,0.934429134033734

PRAIi,0.011368599999999998,0.0

PRPPS,0.1865784000000047,0.1743444000000073

PSCVT,0.07622380000000008,0.06485519999978703

PSERT,0.343634500000006,0.33271360070389255

PSP_L,0.343634500000006,0.33271360070389255

RPE,0.31519592833333127,-0.14383673333296165

RPI,-0.38244516416667035,-0.13587593333342002

SHK3Dr,0.07622380000000008,0.06485519999978703

SHKK,0.07622380000000009,0.06485519999978703

SUCDi,1.024954315000004,2.529443935207742

SUCOAS,-0.9198485150000041,-1.8304665352083092

TALA,0.0,-0.03946846666658731

THD2pp,0.0,0.5001380640833895

TKT1,0.1957321641666656,-0.03946846666658731

TKT2,0.11946376416666554,-0.10436826666637433

TPI,1.7028532254166657,-0.19099553333232822

TRPAS2,-0.0113686,5.491729669231032

TRPS3,0.0113686,0.0

TRPt2rpp,0.0,5.503098269231032

TRPtex,0.0,5.503098269231032

78 reacctions require a flux change for the evolution of the trp__L consumer

.......................................................

.......................................................

uri

.......................................................

ACONTa,1.2398589150000043,1.009109161904803

ACONTb,1.2398589150000041,1.009109161904803

AKGDH,1.024820515000004,0.7940707619047891

ASPCT,0.06615899999999995,0.0

ASPTA,-0.5855358000000094,-0.5193755999999894

ATPS4rpp,13.370320970000053,11.358838245238076

CBMKr,0.12531739999999997,0.059158400000001166

CO2tex,-4.632830737500008,-4.089031354762028

CO2tpp,-4.632830737500008,-4.089031354762028

CS,1.2398589150000043,1.0091091619047892

CYTBO3_4pp,8.410403675000031,7.124330309523962

DHORD2,0.06615899999999995,0.0

DHORTS,-0.06615899999999995,0.0

ENO,3.220598515000006,2.9236885619047825

FBA,1.2128609358333324,0.647554341369073

FUM,1.2333893150000121,1.002638761904791

G6PDH2r,0.7056018925000015,0.7878933690476515

GAPD,3.5642330149999975,3.26732126190484

GLCptspp,2.1404179895833364,0.0

GLCtex_copy1,2.1404179895833364,-0.0009999999999763531

GLUDy,-1.7103415000000157,-1.6441795000000639

GND,0.7056018925000014,0.7878933690476071

H2Otex,-9.914783537500057,-6.633369923809596

H2Otpp,-9.914783537500059,-6.633369923809596

Htex,-1.8376463999999728,-1.7053267999999662

ICDHyr,1.2398589150000043,1.009109161904803

MDH,1.233197915000013,1.0024919619047523

NADH16pp,7.319156560000027,6.328991947619173

NH4tex,2.160164800000022,2.027843999999959

NH4tpp,2.160164800000022,2.027843999999959

O2tex,4.206049537500015,3.5630128547619506

O2tpp,4.206049537500015,3.5630128547619506

OMPDC,0.06615899999999997,0.0

ORPT,-0.06615899999999995,0.0

PDH,1.8733075150000043,1.6425565619047893

PFK,1.2128609358333324,0.6475543413690621

PGI,1.434816097083335,-0.7878933690476515

PGK,-3.564233014999997,-3.26732126190484

PGL,0.7056018925000015,0.7878933690476071

PGM,-3.2205985149999905,-2.9236885619047825

PPC,0.5918716000000012,0.5257121999999999

PPM,-0.00013460000000000268,2.4068116309523475

PYK,0.0,1.908667436488121

PYNP2r,0.0,2.4068116309523475

RPE,0.31519592833333127,1.974598799999967

RPI,-0.38244516416667035,1.1946662309524072

SUCDi,1.024954315000004,0.795338361904789

SUCOAS,-0.9198485150000041,-0.6890991619047782

TALA,0.0,0.8287014358332954

TKT1,0.1957321641666656,1.0254336000000421

TKT2,0.11946376416666554,0.9491652000000386

TPI,1.7028532254166657,1.1395466309523954

UPPRT,0.0,0.066159

URAt2pp_copy2,0.0,-2.340652630952377

URAtex,0.0,-2.340652630952377

URIt2pp_copy2,0.0,2.4068116309523475

URItex,0.0,2.4068116309523475

57 reacctions require a flux change for the evolution of the uri consumer

.......................................................

.......................................................

xan

.......................................................

ACONTa,1.2398589150000043,0.21503840000002583

ACONTb,1.2398589150000041,0.21503840000002583

ADK1,0.524992600000009,0.5668484000000262

ADSL2r,0.08960300000000408,0.0

AICART,0.10855060000000409,0.018947600000046805

AIRC2,0.08960300000000408,0.0

AIRC3,-0.08960300000000408,0.0

AKGDH,1.024820515000004,0.0

ALLTAMH,0.0,6.126105373148276

ALLTN,0.0,6.126105373148276

ASPTA,-0.5855358000000094,-0.4959320000000389

ATPS4rpp,13.370320970000053,17.682725530461312

CO2tex,-4.632830737500008,-22.86894224054663

CO2tpp,-4.632830737500008,-22.801180240546614

CS,1.2398589150000043,0.21503839999999996

CYTBO3_4pp,8.410403675000031,7.587433382047673

DHAPT,0.32252132541666795,0.0

ENO,3.220598515000006,2.195763765038919

F6PA,0.32252132541666795,0.0

FBA,1.2128609358333324,-0.17410213333334923

FBA3,0.19573216416666564,0.0

FBP,0.0,0.17410213333333613

FDH4pp,0.0,0.06776200000000006

FORtppi,0.0,0.06776200000000006

FTHFD,0.0,0.06727140000000004

FUM,1.2333893150000121,0.11896539999997913

G6PDH2r,0.7056018925000015,0.0

GAPD,3.5642330149999975,-0.4813872983722831

GARFT,0.08915700000000398,4.460000002382003e-05

GHMT2r,0.22309610000000604,0.12257220000003599

GLCptspp,2.1404179895833364,0.0

GLCtex_copy1,2.1404179895833364,-0.0009999999999763531

GLNS,0.36212300000000835,0.22377640000000196

GLUDy,-1.7103415000000157,-1.5202130000000125

GLUPRT,0.08964760000000407,4.46e-05

GLXCL,0.0,2.920260863411253

GLYCK,0.0,2.920260863411253

GLYCL,0.010920900000001992,0.0

GMPR,0.0,0.04085940000000193

GMPS2,0.048743200000000084,0.08960260000000196

GND,0.7056018925000014,0.0

H2Otex,-9.914783537500057,27.79049913024369

H2Otpp,-9.914783537500059,27.790497352748844

HCO3E,0.10516900000000409,0.015564799999992829

Htex,-1.8376463999999728,23.025184130101934

ICDHyr,1.2398589150000043,0.21503840000002583

IMPC,-0.10855060000000409,-0.018947600000046805

IMPD,0.048743200000000084,0.0

MALS,0.0001338000000000684,0.28571744632577

MDH,1.233197915000013,6.530507619474065

MOX,0.00032519999999967104,-6.125824773148224

MTHFC,0.1977522000000081,0.0863081999999622

MTHFD,0.1977522000000081,0.0863081999999622

NADH16pp,7.319156560000027,7.452244782047673

NH4tex,2.160164800000022,-22.70266989259312

NH4tpp,2.160164800000022,-22.70266989259312

O2tex,4.206049537500015,3.795064391023857

O2tpp,4.206049537500015,3.795064391023857

PDH,1.8733075150000043,1.134083281286816

PFK,1.2128609358333324,0.0

PFK_3,0.19573216416666564,0.0

PGCD,0.34363450000000595,0.24310980000000004

PGI,1.434816097083335,0.0

PGK,-3.564233014999997,0.4813872983722831

PGL,0.7056018925000015,0.0

PGM,-3.2205985149999905,-2.195763765038919

PPC,0.5918716000000012,0.3062875536742322

PPK,-0.7007176000000049,-0.7415497150743704

PRAGSr,0.08964760000000409,4.460000002382003e-05

PRAIS,0.08964760000000409,4.46e-05

PRASCSi,0.0896030000000041,0.0

PRFGS,0.08964760000000407,4.46e-05

PSERT,0.343634500000006,0.24310980000000004

PSP_L,0.343634500000006,0.24310980000000004

PYK,0.0,1.7236886113647238

RPE,0.31519592833333127,-0.15520453333328987

RPI,-0.38244516416667035,-0.14724373333331187

SUCDi,1.024954315000004,0.0021337999999999913

SUCOAS,-0.9198485150000041,0.10397160000002259

TALA,0.0,-0.0394680666667

THD2pp,0.0,2.39818136503896

TKT1,0.1957321641666656,-0.0394680666667

TKT2,0.11946376416666554,-0.11573646666670356

TPI,1.7028532254166657,-0.20236333333332368

TRSARr,0.0,2.9192608634112958

UGLYCH,0.0,6.126105373148276

URIC,0.0,6.126105373148276

XAND,0.0,6.126105373148276

XANtex,0.0,6.215707973148255

XANtpp,0.0,6.215707973148255

XPPT,0.0,0.08960260000000196

91 reacctions require a flux change for the evolution of the xan consumer

.......................................................

.......................................................

xtsn

.......................................................

ACONTa,1.2398589150000043,0.3296903053447977

ACONTb,1.2398589150000041,0.3296903053447977

ADK1,0.524992600000009,0.5649833999999601

ADSL2r,0.08960300000000408,0.0

AICART,0.10855060000000409,0.018947600000046805

AIRC2,0.08960300000000408,0.0

AIRC3,-0.08960300000000408,0.0

AKGDH,1.024820515000004,0.11465190534485176

ALLTAMH,0.0,1.6440586405172528

ALLTN,0.0,1.6440586405172528

ASPTA,-0.5855358000000094,-0.4959320000000389

ATPS4rpp,13.370320970000053,12.845290169051964

CO2tex,-4.632830737500008,-9.126949605172513

CO2tpp,-4.632830737500008,-9.059187605172497

CS,1.2398589150000043,0.329690305344852

CYTBO3_4pp,8.410403675000031,6.996676367241515

ENO,3.220598515000006,2.3104287053448616

FBA,1.2128609358333324,0.08082825498559032

FDH4pp,0.0,0.06776199999999995

FORtppi,0.0,0.06776199999999995

FTHFD,0.0,0.06727139999999993

FUM,1.2333893150000121,0.2336173053448647

G6PDH2r,0.7056018925000015,1.1417708473276207

GAPD,3.5642330149999975,2.0274447850862316

GARFT,0.08915700000000398,4.460000002382003e-05

GHMT2r,0.22309610000000604,0.12257220000003599

GLCptspp,2.1404179895833364,0.0

GLCtex_copy1,2.1404179895833364,-0.0009999999999763531

GLNS,0.36212300000000835,0.2237763999999979

GLUDy,-1.7103415000000157,-1.5202130000000125

GLUPRT,0.08964760000000407,4.46e-05

GLXCL,0.0,0.5260937202586258

GLYCK2,1.4997824761187406e-14,0.5260937202586258

GLYCL,0.010920900000001992,0.0

GMPR,0.0,0.04085939999999784

GMPS2,0.048743200000000084,0.08960259999999787

GND,0.7056018925000014,1.1417708473275732

H2Otex,-9.914783537500057,2.927717600000051

H2Otpp,-9.914783537500059,2.927717600000051

HCO3E,0.10516900000000409,0.015564799999992829

Htex,-1.8376463999999728,5.09700016206898

ICDHyr,1.2398589150000043,0.3296903053447977

IMPC,-0.10855060000000409,-0.018947600000046805

IMPD,0.048743200000000084,0.0

MALS,0.0001338000000000684,0.5920050000000012

MDH,1.233197915000013,2.469355745862117

MOX,0.00032519999999967104,-1.6437334405172805

MTHFC,0.1977522000000081,0.0863081999999622

MTHFD,0.1977522000000081,0.0863081999999622

NADH16pp,7.319156560000027,6.745835861896664

NH4tex,2.160164800000022,-4.774482962068987

NH4tpp,2.160164800000022,-4.774482962068987

O2tex,4.206049537500015,3.4991858836207257

O2tpp,4.206049537500015,3.4991858836207257

PDH,1.8733075150000043,1.5550089053448533

PFK,1.2128609358333324,0.0808282549856433

PGCD,0.34363450000000595,0.2431098000000001

PGI,1.434816097083335,-1.1417708473276207

PGK,-3.564233014999997,-2.0274447850862316

PGL,0.7056018925000015,1.1417708473275732

PGM,-3.2205985149999905,-1.784334985086275

PPC,0.5918716000000012,0.0

PPK,-0.7007176000000049,-0.7417807000002767

PPM,-0.00013460000000000268,1.7336612405172218

PRAGSr,0.08964760000000409,4.460000002382003e-05

PRAIS,0.08964760000000409,4.46e-05

PRASCSi,0.0896030000000041,0.0

PRFGS,0.08964760000000407,4.46e-05

PSERT,0.343634500000006,0.2431098000000001

PSP_L,0.343634500000006,0.2431098000000001

PUNP7,0.0,1.7336612405172218

PYK,0.0,1.8211197799281857

RPE,0.31519592833333127,1.7617501918965672

RPI,-0.38244516416667035,0.6279401445689246

SUCDi,1.024954315000004,0.1177857053448518

SUCOAS,-0.9198485150000041,-0.00968030534488662

TALA,0.0,0.7222771317816523

TKT1,0.1957321641666656,0.9190092959482854

TKT2,0.11946376416666554,0.8427408959482818

TPI,1.7028532254166657,0.5728205445690264

TRSARr,0.0,0.5260937202585865

UGLYCH,0.0,1.6440586405172528

URIC,0.0,1.6440586405172528

XAND,0.0,1.6440586405172528

XPPT,0.0,0.08960259999999787

XTSNt2rpp,0.0,1.7336612405172218

XTSNtex,0.0,1.7336612405172218

87 reacctions require a flux change for the evolution of the xtsn consumer

.......................................................

.......................................................
