# Supplementary material for: Acetate and glycerol are not uniquely suited for the evolution of cross-feeding in E. coli
Source: PLoS Comput Biol. 2020 Nov 30;16(11):e1008433. doi: 10.1371/journal.pcbi.1008433 (PMC7728234; doi:10.1371/journal.pcbi.1008433)
Supplement: S2 Text — (DOCX) [file pcbi.1008433.s002.docx]

**S2_text**

**The following nine reactions require a flux change for the evolution of every producer strain, i.e., for all 58 carbon sources we consider.** (Each line below shows, from left to right, the reaction acronym, the reaction name, and the metabolic subsystem to which the reaction belongs.)

DHAPT, Dihydroxyacetone phosphotransferase, Alternate Carbon Metabolism

F6PA, Fructose 6-phosphate aldolase, Glycolysis/Gluconeogenesis

G6PDH2r, Glucose 6-phosphate dehydrogenase, Pentose Phosphate Pathway

GLCptspp, D-glucose transport via PEP:Pyr PTS (periplasm), Transport, Inner Membrane

GND,Phosphogluconate dehydrogenase, Pentose Phosphate Pathway

H2Otex, H2O transport via diffusion (extracellular to periplasm, Transport, Outer Membrane Porin

H2Otpp, H2O transport via diffusion (periplasm, Transport, Inner Membrane

PGL, 6-phosphogluconolactonase, Pentose Phosphate Pathway

TPI, Triose-phosphate isomerase, Glycolysis/Gluconeogenesis

**The following four reactions require a flux change for the evolution of every consumer strain, i.e., for all 58 carbon sources we consider.** (Each line below shows, from left to right, the reaction acronym, the reaction name, and the metabolic subsystem to which the reaction belongs.)

GLCptspp, D-glucose transport via PEP:Pyr PTS (periplasm), Transport, Inner Membrane

GLCtex_copy1, None, Transport, Outer Membrane Porin

PGI, Glucose-6-phosphate isomerase, Glycolysis/Gluconeogenesis

TPI, Triose-phosphate isomerase, Glycolysis/Gluconeogenesis
