# Supplementary material for: Acetate and glycerol are not uniquely suited for the evolution of cross-feeding in E. coli
Source: PLoS Comput Biol. 2020 Nov 30;16(11):e1008433. doi: 10.1371/journal.pcbi.1008433 (PMC7728234; doi:10.1371/journal.pcbi.1008433)
Supplement: S3 Text — (DOCX) [file pcbi.1008433.s003.docx]

**S3_text.**

**Comparing computational predictions and experimental data on genomic mutations and transcriptional changes**

Thorough experimental analysis performed on the ancestral and acetate/glycerol cross-feeding *E. coli* strains provided extensive information on mutations [1] and gene expression differences among strains [2]. Here we compare these experimentally obtained data to our computational predictions.

A total of 535 different genes had undergone mutations in evolved cross-feeding strains relative to the ancestral (JA122) strain (data from supplementary Table S2 of [1]). 168 out of those 535 mutated genes are genes included in the metabolic model of *E. coli* [3] that we used. In a first analysis, we explored the agreement between the mutated genes, and those genes associated with reactions that we computationally predict to require a flux change for the evolution of cross-feeding.

To this end, we used the Gene-Protein-Reaction association (GPR) map available for the metabolic model of *E. coli i*JO1366 [3]. The relationship between genes and enzymatic reactions is only one-to-one in the simplest case, where one gene product catalyzes one reaction. Alternatively, a gene product may catalyze more than one reaction; the products of multiple genes may be needed to catalyze one reaction; or the products of different genes may catalyze the same reaction.

We mapped every reaction that we predict (with RooM) to require a flux change for the evolution of a cross-feeding strain to its corresponding gene or genes (see supplementary S1 File). Then, for each cross-feeding strain, we determined whether a gene associated with a flux-changing reaction underwent one or more mutations in the experiment. If so, we reported the number of mutations found in the gene (supplementary S1 File).

A few genes associated with flux-changing reactions were mutated in the chemostat experiments [1]. We observed the largest agreement between computational predictions and experimental data for the acetate producer strain, where 16 of 118 genes (approximately 7%) associated with flux-changing reactions were mutated in strain CV103 (supplementary S1 File).

Transcriptional profiling of the evolved strains (acetate and glycerol cross-feeding strains) in monoculture revealed 181 genes whose expression was significantly altered in the evolved strains relative to the ancestral strain [2]. Approximately half (93) of these genes were classified as metabolic genes (using MultiFun [4]). Forty-nine genes were up- or down-regulated relative to the ancestral strain, and showed similar expression changes in the acetate and glycerol cross-feeding strains. Forty-four genes showed different expression changes among the cross-feeding strains.

Although our analysis is based on metabolic fluxes and not gene expression, and although the two quantities may show very limited association, for example because of post-translational regulation and differences in enzyme half-lives [5–11], we wished to analyze to what extent computationally predicted metabolic changes agree with published experimental gene expression data. To this end, we again used the GPR map available for the model of *E. coli* to compare flux predictions with gene expression data. Gene expression data is available for 72 genes included in the genome scale metabolic reconstruction we used for this analysis (iJO1366)[1,2]. (See supplementary S2 File for each gene’s Blattner id and expression change). The products of most of these genes are associated with few reactions. Exceptions include ompF (Blattner id b0929), tesA (b0494), and ptsH (b2415), which are associated with 271, 21, and 18 reactions. Gene ompF expresses an outer membrane porin which is involved in many transport reactions; tesA expresses acyl-CoA thioesterase I, an enzyme involved in the biosynthesis of unsaturated fatty acids; and ptsH expresses the phosphocarrier protein HPr involved in the phosphotranspherase system (PTS). In total, these 72 genes are associated with 457 reactions (column G in supplementary S2 File).

Four strains evolved and coexisted for many generations in the glucose minimal chemostat experiment [12]: an acetate consumer (strain CV101), an acetate and glycerol producer (strain CV103), and two glycerol consumers (strains CV115 and CV116). With the aim to disentangle the metabolic changes required for acetate and glycerol production and consumption, we computationally predicted four different flux distributions, i.e., a distribution for an acetate producer, an acetate consumer, a glycerol producer, and a glycerol consumer. We then compared the experimentally observed gene expression changes with the computationally identified flux changes relative to the ancestor. Specifically, we compared (i) the computationally predicted flux changes for the acetate producer strain with the gene expression changes observed experimentally for strain CV103 (an acetate and glycerol producer); (ii) the computationally predicted flux changes for the acetate consumer strain with the gene expression changes observed experimentally for strain CV101 (an acetate consumer strain); (iii) the computationally predicted flux changes for the glycerol producer strain with the gene expression changes observed experimentally for strain CV101 (which produces both acetate and glycerol); and (iv) the computationally predicted flux changes for the glycerol consumer strain with the gene expression changes observed experimentally for strains CV115 and CV116 (two glycerol consumer strains).

For each pair of computationally predicted and experimentally observed changes we performed the following analyses. First, we classified all 72 metabolic genes in the metabolic network of *E. coli* as up-regulated, down-regulated or unchanged relative to the ancestor, using available gene expression data from [1,2] for the strains listed above. Then, we classified all (1295) remaining genes included in the model of *E. coli* which were not part of these 72 genes as genes with unchanged expression. Supplementary S3 Fig shows in the form of grey bars the number of genes that were classified as up-regulated, down-regulated or unchanged for every experimentally observed strain.

Second, for every gene classified as up-regulated, we examined if at least one of the associated reactions showed a flux increase in the evolved strain (flux$e_{i}$)relative to the ancestral strain (flux$a_{i}$, $\left| e_{i} \right|>\left| a_{i} \right|+0.001)$. If so, we considered that the experimentally observed gene expression change agreed with the computationally predicted flux change. We applied the same procedure to genes classified as down-regulated, requiring that at least one of the associated reactions showed a flux decrease ($\left| e_{i} \right|<\left| a_{i} \right|-0.001$) to consider that experimental data agreed with the computational prediction. For genes classified as unchanged, we only assumed agreement between experiment and computational prediction when all reactions associated with a gene showed the same flux in the evolved and the ancestral strain ($\left| a_{i} \right|-0.001<\left| e_{i} \right|<\left| a_{i} \right|+0.001$).

We repeated the analysis just described for computational predictions based on RooM, MoMA, minimization of changed reaction subsets, and RooM-het. The results are shown in supplementary S3 Fig. Green bars in supplementary S3 Fig show the number of genes showing agreement between the experiment and computation (true positives). Orange bars show the number of false positives, i.e., genes computationally predicted to be up-regulated, down-regulated, or unchanged, but where this prediction was not supported by experimental data.

The same data is summarized in panel (F) of supplementary S3 Fig, a table whose entries show the number of true positives and false positives, respectively, separated by a slash (‘/’). We performed a Fisher exact test to test the null hypothesis that the number of genes correctly predicted to be up-regulated, down-regulated or unchanged cannot be attributed to chance alone. We found predictions to be significant (p-value<0.05, denoted with *) and very significant (p-value<0.01, denoted with **) for all consumer strains, irrespective of the computational method used to identify the strain.

Taken together, these analyses show that the agreement between experimental data and computational predictions is limited, which is unsurprising. Little agreement with mutant data may exist because mutations not directly affecting metabolic genes can have large effects on the rate at which metabolic reactions proceed. This is the case, for example, with regulatory mutations. Little agreement with transcriptomic data can arise from the fact that metabolic flux is affected by multiple factors besides mRNA expression levels, including post-translational and allosteric regulation. An additional source of disagreement may come from the fact that the strain found experimentally to excrete acetate (CV103) also excretes glycerol, while we computationally modeled two separate producer strains excreting either acetate or glycerol.

**References**

1. Kinnersley MA, Wenger J, Kroll E, Adams J, Sherlock G, Rosenzweig F. Ex Uno Plures: Clonal Reinforcement Drives Evolution of a Simple Microbial Community. PLoS Genet. 2014;10: e1004430–e1004430. doi:10.1371/journal.pgen.1004430

2. Kinnersley MA, Holben WE, Rosenzweig F. E Unibus Plurum: Genomic Analysis of an Experimentally Evolved Polymorphism in Escherichia coli. PLOS Genetics. 2009;5: e1000713. doi:10.1371/journal.pgen.1000713

3. Orth JD, Conrad TM, Na J, Lerman JA, Nam H, Feist AM, et al. A comprehensive genome-scale reconstruction of Escherichia coli metabolism. Mol Syst Biol. 2011. doi:10.1038/msb.2011.65

4. Serres M, Riley M. MultiFun, a Multifunctional Classification Scheme for Escherichia coli K-12 Gene Products. Microbial & Comparative Genomics. 2000;5: 205–222. doi:10.1089/omi.1.2000.5.205

5. Futcher B, Latter GI, Monardo P, McLaughlin CS, Garrels JI. A Sampling of the Yeast Proteome. Mol Cell Biol. 1999;19: 7357. doi:10.1128/MCB.19.11.7357

6. Greenbaum D, Colangelo C, Williams K, Gerstein M. Comparing protein abundance and mRNA expression levels on a genomic scale. Genome Biology. 2003;4: 117. doi:10.1186/gb-2003-4-9-117

7. Ideker T, Thorsson V, Ranish JA, Christmas R, Buhler J, Eng JK, et al. Integrated Genomic and Proteomic Analyses of a Systematically Perturbed Metabolic Network. Science. 2001;292: 929. doi:10.1126/science.292.5518.929

8. Washburn MP, Koller A, Oshiro G, Ulaszek RR, Plouffe D, Deciu C, et al. Protein pathway and complex clustering of correlated mRNA and protein expression analyses in Saccharomyces cerevisiae. Proc Natl Acad Sci U S A. 2003;100: 3107–3112. doi:10.1073/pnas.0634629100

9. Vogel C, Marcotte EM. Insights into the regulation of protein abundance from proteomic and transcriptomic analyses. Nature Reviews Genetics. 2012;13: 227.

10. Guimaraes JC, Rocha M, Arkin AP. Transcript level and sequence determinants of protein abundance and noise in Escherichia coli. Nucleic Acids Res. 2014;42: 4791–4799. doi:10.1093/nar/gku126

11. Gygi SP, Rochon Y, Franza BR, Aebersold R. Correlation between Protein and mRNA Abundance in Yeast. Mol Cell Biol. 1999;19: 1720. doi:10.1128/MCB.19.3.1720

12. Helling RB, Vargas CN, Adams J. Evolution of Escherichia coli during growth in a constant environment. Genetics. 1987.
