# Supplementary material for: Acetate and glycerol are not uniquely suited for the evolution of cross-feeding in E. coli
Source: PLoS Comput Biol. 2020 Nov 30;16(11):e1008433. doi: 10.1371/journal.pcbi.1008433 (PMC7728234; doi:10.1371/journal.pcbi.1008433)
Supplement: S4 Text — (DOCX) [file pcbi.1008433.s004.docx]

**S4_text**

**Alternative methods used to identify the cross-feeding strains.**

In this supplementary file we introduce alternative methods that we used to identify the flux distribution of the cross-feeding strains. We also present a comparison of the results obtained with these methods and the results presented in the main text obtained with RooM.

**Minimization of Metabolic Adjustments (MoMA)**

MoMA, like RooM, was first described [1] to predict metabolic changes after genetic perturbations such as gene deletion. It is based on the assumption that after a genetic perturbation, the flux change in a metabolic network relative to a wild-type will be minimal. It identifies a flux distribution that satisfies a set of constraints while minimizing the distance to a flux distribution, just as RooM does. The difference between RooM and MoMA consists in the distance metric used: While in RooM the *number* of reactions with a significant flux change relative to a reference flux distribution is minimized, in MoMA the *Euclidean distance* to the reference flux distribution is minimized. The quadratic programming problem solved in MoMA can be written as:

Min $\sqrt{\sum{{(a}_{i}-e_{i})}^{2}}$

$$s.t. Se=0$$

$$l_{i}\leq e_{i}\leq u_{i}$$

Here, $S$ is the $m\times r$stoichiometry matrix and $l_{i}$ and $u_{i}$ are lower and upper bounds, respectively, that constrain the flux through each reaction $i$ in the network. These bounds reflect thermodynamic and capacity constraints on a reaction. The flux distribution $a$ corresponds to the reference flux distribution, which for our analysis equals the flux distribution of the ancestral strain obtained with pFBA, as explained in the main text.

We repeated the analysis presented in the main text, identifying every producer and consumer strain with MoMA instead of RooM. We found that the mean ancestor-producer distance was 10.3±7.7 mmol gDW^-1^ h^-1^ (supplementary S4 Fig). The distances between the ancestor and the acetate and glycerol producer strains were 3.3 mmol gDW^-1^ h^-1^ and 2.9 mmol gDW^-1^ h^-1^, respectively. When ranking producer strains according to increasing distance to the ancestor, the acetate producer had rank eleven, while the glycerol had rank six. The ancestor-consumer mean distance was 27.8±41.6mmol gDW^-1^ h^-1^ (supplementary S4 Fig). The acetate consumer strain occupied rank 47, with a distance of 26.5mmol gDW^-1^ h^-1^. The glycerol consumer strain ranked third, with a distance of 8.6 mmol gDW^-1^ h^-1^.

We used the sum of the ancestor-producer and ancestor-consumer distances as a proxy for the likelihood of cross-feeding interactions to evolve (supplementary S4 Fig). We observed that 34 cross-feeding interactions can evolve more easily than the one involving acetate. In contrast, only two cross-feeding interactions can evolve more easily than glycerol cross-feeding, those involving the metabolites dihydroxyacetone (dha) and D-gluconate (glcn). Even though MoMA and RooM perform different optimizations to identify the flux distributions of the cross-feeding strains and even though the distances obtained differ in absolute values, we observed a high statistical association (Spearman’s r= 0.86, P=6.1e-18, n=58) between the predictions of both methods with respect to metabolite rank, i.e., the likelihood with which metabolite cross-feeding arises.

**Minimizing the number ofreaction subsets with a significant change in flux**

Regulatory mutations may be important for causing the flux changes that allow cross-feeding, and any one regulatory mutation may affect the flux through multiple reactions simultaneously. Bearing this in mind, we wanted to quantify the number of flux changes not in terms of individual reactions (as in RooM) but in terms of subsets of reactions which are co-regulated and whose flux may be simultaneously affected by a single regulatory change. To this end, we first identified all subsets of fully coupled reactions. (A set of reactions is fully coupled if the flux through one reaction constrains the flux through all reactions in the set to a scalar multiple of the specified flux [2].These fully coupled reactions tend to be co-regulated [3]. We used the tool F2C2 [2] to identify such reactions. We included all reactions that were fully coupled in one subset of reactions. With this approach, we partitioned the 2583 reactions from the *E. coli* metabolic model *i*JO1366 into 1118 subsets of fully coupled reactions.

We then performed an optimization similar to RooM, in which a flux distribution that fulfills a set of constraints is identified, while the number of subsets of reactions that change their flux with respect to the ancestral flux distribution is minimized. The optimization problem can be written as:

Min $\sum f_{i}^{subset}$

$$s.t. Se=0$$

$$f_{i}^{subset}\in\{0,1\}$$

$$l_{i}\leq e_{i}\leq u_{i}$$

$$e_{i}-f_{i}^{subset}(u_{i}-(a_{i}+\beta))\leq a_{i}+\beta$$

$$e_{i}-f_{i}^{subset}(l_{i}-(a_{i}-\beta))\geq a_{i}-\beta$$

Once again, here $S$ is the $m\times r$ stoichiometry matrix and $l_{i}$ and $u_{i}$ are lower and upper bounds respectively, that constrain the flux through each reaction in the network according to thermodynamic and capacity constraints. $f_{i}^{subset}$ is a binary variable. It takes a value of 1 if reaction subset$i$ shows a substantial change in flux $e_{i}$ relative to the reference flux $a_{i}$ and zero otherwise.$\beta$ specifies the amount of flux change that is considered substantial, and as in RooM, we used a value of $\beta=0.001$.

We used this method to identify all potential cross-feeding strains. We found that producers differed from the ancestor on average in 50±10 reaction subsets while consumers differed from the ancestor on average in 55±13 reaction subsets (supplementary S4 Fig). We also used the sum of the ancestor-producer and ancestor-consumer distances obtained with this method to estimate the likelihood of the various cross-feeding interactions to evolve. For acetate and glycerol cross-feeding 92 and 83 subsets of reactions require a flux change, respectively. Cross-feeding of 17 metabolites is easier to evolve than acetate cross-feeding, whereas cross-feeding of only 6 metabolites is easier to evolve than glycerol cross-feeding.

Although the results presented in the main text considered all reactions, whereas the method used here considered only reactions subset, the methods produced highly concordant predictions about metabolite rank,i.e., about the likelihood that metabolite cross-feeding arises (Spearman’s r= 0.98, P=2.4e-44, n=58).

1. Segrè D, Vitkup D, Church GM. Analysis of optimality in natural and perturbed metabolic networks. Proc Natl Acad Sci USA. 2002;99: 15112. doi:10.1073/pnas.232349399

2. Larhlimi A, David L, Selbig J, Bockmayr A. F2C2: a fast tool for the computation of flux coupling in genome-scale metabolic networks. BMC Bioinformatics. 2012;13: 57–57. doi:10.1186/1471-2105-13-57

3. Notebaart RA, Teusink B, Siezen RJ, Papp B. Co-Regulation of Metabolic Genes Is Better Explained by Flux Coupling Than by Network Distance. PLOS Computational Biology. 2008;4: e26. doi:10.1371/journal.pcbi.0040026
