# Supplementary material for: Acetate and glycerol are not uniquely suited for the evolution of cross-feeding in E. coli
Source: PLoS Comput Biol. 2020 Nov 30;16(11):e1008433. doi: 10.1371/journal.pcbi.1008433 (PMC7728234; doi:10.1371/journal.pcbi.1008433)
Supplement: S5 Text — (DOCX) [file pcbi.1008433.s005.docx]

**S5_text**

**Model to quantify the probability of cross-feeding evolution**

The evolution of the consumer strain may not be independent of that of the producer. For example, if the producer is not present in the population by the time the consumer evolves, the consumer will go extinct because it lacks a carbon source on which to thrive. Therefore, the sum of ancestor-producer and ancestor-consumer distances may not be the most accurate proxy for the likelihood that cross-feeding evolves. In this section we develop a more adequate model that does not consider producer and consumer origins as independent. We also show that the predictions of this more complex model are virtually identical to those of the simpler model of the main text.

One fundamental assumption of our work is that metabolic ancestor-producer and ancestor-consumer distances are proportional to the amount of metabolic or genetic change that is necessary to give rise to a producer or a consumer strain for any one carbon source. In addition, we assume that these distances are inversely proportional to the likelihood that a producer or consumer strain emerges. In a reflection of these assumptions, we transformed metabolic distances ($d$) into probabilities that producers (consumers) emerge in the population after one generation ($P$). To this end, we took a phenomenological approach and assumed that the probability that an evolved (producer or consumer) strain originates in the population from the ancestor would have a maximal value of $P=1$ if the metabolic distance between ancestor and the evolved strain had a minimal value of zero. This probability would monotonically approach the smallest value of zero as the metabolic distance became larger. We used the following simple function to transform metabolic distances into probabilities of evolution: $P=\frac{1}{d+1}$ (1)

Equation (1) predicts, for example, that the producer of dihydroxyacetone (dha), which requires the fewest flux changes (19 reactions, Fig 2A), is the one with the highest probability to emerge in the population ($P=0.050$). In addition, the dihydroxyacetone (dha) consumer strain is also the consumer strain with the highest probability to emerge ($P=0.11$), because it requires only 8 reactions (Fig 2A) to change their flux relative to the ancestral strain. Conversely, the strain producing L-alanine-D-glutamate-meso-2,6-diaminoheptanedioate-D-alanine (LalaDgluMdapDala) has the lowest probability of emerging among all producer strains (90 flux changes, P=0.011), and the strain consuming formate (for) has the lowest probability among all consumer strains (99 reaction changes, P= 0.010).

We used these probabilities to model the evolution of cross-feeding. For simplicity, we assumed that generations are non-overlapping and that at every generation four scenarios are possible: the producer and consumer strains arise simultaneously, which occurs with probability $P_{Producer˄Consumer}$; only the producer arises (probability$P_{Producer˄(\neg Consumer)}$); only the consumer arises (probability $P_{(\neg Producer)˄Consumer}$); or neither producer nor consumer arises (probability $P_{(\neg Producer)˄(\neg Consumer)}$). For cross-feeding to evolve after the first generation, producer and consumer need to arise simultaneously. Therefore the probability of observing cross-feeding after the first generation is given by $P(cf,t_{1})$ =$P_{Producer˄Consumer}$. From the second generation onwards cross-feeding may arise by simultaneous evolution of producer and consumer (as in the first generation) or by evolution of a consumer after the evolution of a producer. The probability of evolution of cross-feeding in generation $t$ (with $t>t_{1}$) can be written as:

$P\left( cf,t \right)= {P_{(\neg Producer)}}^{t-1}P_{Producer˄Consumer}+\sum_{m=2}^{t} \sum_{j=0}^{t-m} {P_{(\neg Producer)}}^{m-2}{P_{Producer˄(\neg Consumer)}}^{j+1}{P_{(\neg Producer)˄(\neg Consumer)}}^{t-m-j}\frac{\left( t-m \right)!}{j!\left( t-m-j \right)!}P_{Consumer}$ (2)

Where $P_{(\neg Producer)}$ (with $P_{(\neg Producer)}=P_{(\neg Producer)˄Consumer}+P_{\left( \neg Producer \right)˄(\neg Consumer)}$) is the probability that the producer does not emerge in a given generation. $P_{Consumer}$(with $P_{(Consumer)}=P_{Producer˄Consumer}+P_{\left( \neg Producer \right)˄Consumer}$) is the probability of the consumer to emerge in a given generation.

Defining $p$ and $c$as the probabilities that producer and consumer evolve in the population, then for a population composed of N cells (N=2x10^10^ in [1]) we can write the following probabilities:

$$P_{(\neg Producer)}= \left( 1-p \right)^{N}$$

$$P_{(Producer)}=1-P_{(\neg Producer)}= 1-\left( 1-p \right)^{N}$$

$$P_{(\neg Consumer)}= \left( 1-c \right)^{N}$$

$$P_{(Consumer)}=1-P_{(\neg Consumer)}= 1-\left( 1-c \right)^{N}$$

$$P_{\left( \neg Producer \right)˄(\neg Consumer)}= {(1-(p+c))}^{N}$$

$$P_{Producer˄Consumer}= \sum_{r_{P}=1}^{N-1} \sum_{r_{C}=1}^{N-r_{P}} \frac{N!p^{r_{P}}c^{r_{C}}\left( 1-p-c \right)^{N-r_{P}-r_{C}}}{r_{P}!r_{C}!\left( N-r_{P}-r_{C} \right)!}=1+{(1-p-c)}^{N}-{(p+(1-p-c))}^{N}-{(c+(1-p-c))}^{N}$$

$$P_{Producer˄\left( \neg Consumer \right)}{=P}_{Producer}-P_{Producer˄Consumer}$$

$$P_{\left( \neg Producer \right)˄Consumer}= P_{Consumer}-P_{Producer˄Consumer}$$

We used equation (1) to estimate the probabilities of the producer $P_{Producer}$ and consumer $P_{Consumer}$ strains to evolve in a population. With these probabilities, we were able to calculate the probabilities of producer and consumer to evolve per cell in the population ($p$ and $c$). With $p$ and $c$ in hand, we determined the remaining probabilities needed to calculate the probability of cross-feeding: $P_{\left( \neg Producer \right)˄(\neg Consumer)}$, $P_{Producer˄Consumer}$, $P_{\left( Producer \right)˄(\neg Consumer)}$ and $P_{\left( \neg Producer \right)˄Consumer}$.

Supplementary S5 Fig (A) plots the predicted cumulative probability ($\sum_{t} P\left( cf,t \right)$)

that cross-feeding emerges against time (in generations) for the evolution of each of the 58 possible cross-feeding interactions. Orange and green lines show the predictions for acetate and glycerol respectively. Supplementary S5 Fig (B) compares this prediction to that of the simpler model of the main text, where the sum of the ancestor-producer and ancestor-consumer distances are used as proxies for these probabilities. The two predictions are highly concordant (Spearman’s r=0.99, P=9.7e-74, n=58).

References

1. Helling RB, Vargas CN, Adams J. Evolution of Escherichia coli during growth in a constant environment. Genetics. 1987;
