# Supplementary material for: Acetate and glycerol are not uniquely suited for the evolution of cross-feeding in E. coli
Source: PLoS Comput Biol. 2020 Nov 30;16(11):e1008433. doi: 10.1371/journal.pcbi.1008433 (PMC7728234; doi:10.1371/journal.pcbi.1008433)
Supplement: S6 Text — (DOCX) [file pcbi.1008433.s006.docx]

**S6_text**

**Which novel cross-feeding interactions are likely to be found experimentally?**

In this section we offer some informed speculations on still unknown cross-feeding interactions that are most likely to be found, based on their likelihood to evolve and to be discovered experimentally.

In previous work [1] we simulated the dynamics of a producer and consumer strain in a chemostat. We observed that coexistence of the producer and consumer strains was possible for all 58 predicted metabolites in a wide range of conditions. However, we also observed that the cross-fed metabolite affected the community biomass (the sum of the biomass values of producer and consumer strains) considerably. We proved that the community biomass was explained by the product of two quantities: the maximal flux of the cross-fed metabolite that can be excreted by the producer strain (which is related to the cost of producing this metabolite), and the biomass yield of the metabolite (which is the growth rate that can be achieved per unit of metabolite consumed).We also showed that approximately half of the predicted cross-feeding interactions (29 out of 58) support higher community biomass than acetate cross-feeding [1] (supplementary S9 Fig). Cross-feeding interactions resulting in larger community biomass may be easiest to detect experimentally.

Alpha-ketoglutarate (akg), citrate (cit), pyruvate (pyr) and aspartate (asp-L) are the four metabolites that (i) support highest community biomass [1], (ii) can be excreted by E. coli in glucose medium [2], and (iii) require few metabolic changes for their cross-feeding interaction to evolve (Fig 2 of the present work and supplementary S9 Fig). Additionally, to our knowledge none of these metabolites is toxic for *E. coli*. *E. coli* is normally unable to grow aerobically on citrate, due to the inability to express a citrate transporter in the presence of oxygen, which would hinder citrate cross-feeding evolution. Taken together, these considerations suggest that alpha-ketoglutarate, pyruvate and aspartate may be the best candidates for involvement in cross-feeding interactions that can both readily evolve and be detected experimentally.

1. San Roman M, Wagner A. An enormous potential for niche construction through bacterial cross-feeding in a homogeneous environment. PLOS Computational Biology. 2018;14: e1006340. doi:10.1371/journal.pcbi.1006340

2. Paczia N, Nilgen A, Lehmann T, Gätgens J, Wiechert W, Noack S. Extensive exometabolome analysis reveals extended overflow metabolism in various microorganisms. Microbial Cell Factories. 2012;11: 122. doi:10.1186/1475-2859-11-122
