# Supplementary figures and images for: Acetate and glycerol are not uniquely suited for the evolution of cross-feeding in E. coli
Source: PLoS Comput Biol. 2020 Nov 30;16(11):e1008433. doi: 10.1371/journal.pcbi.1008433 (PMC7728234; doi:10.1371/journal.pcbi.1008433)

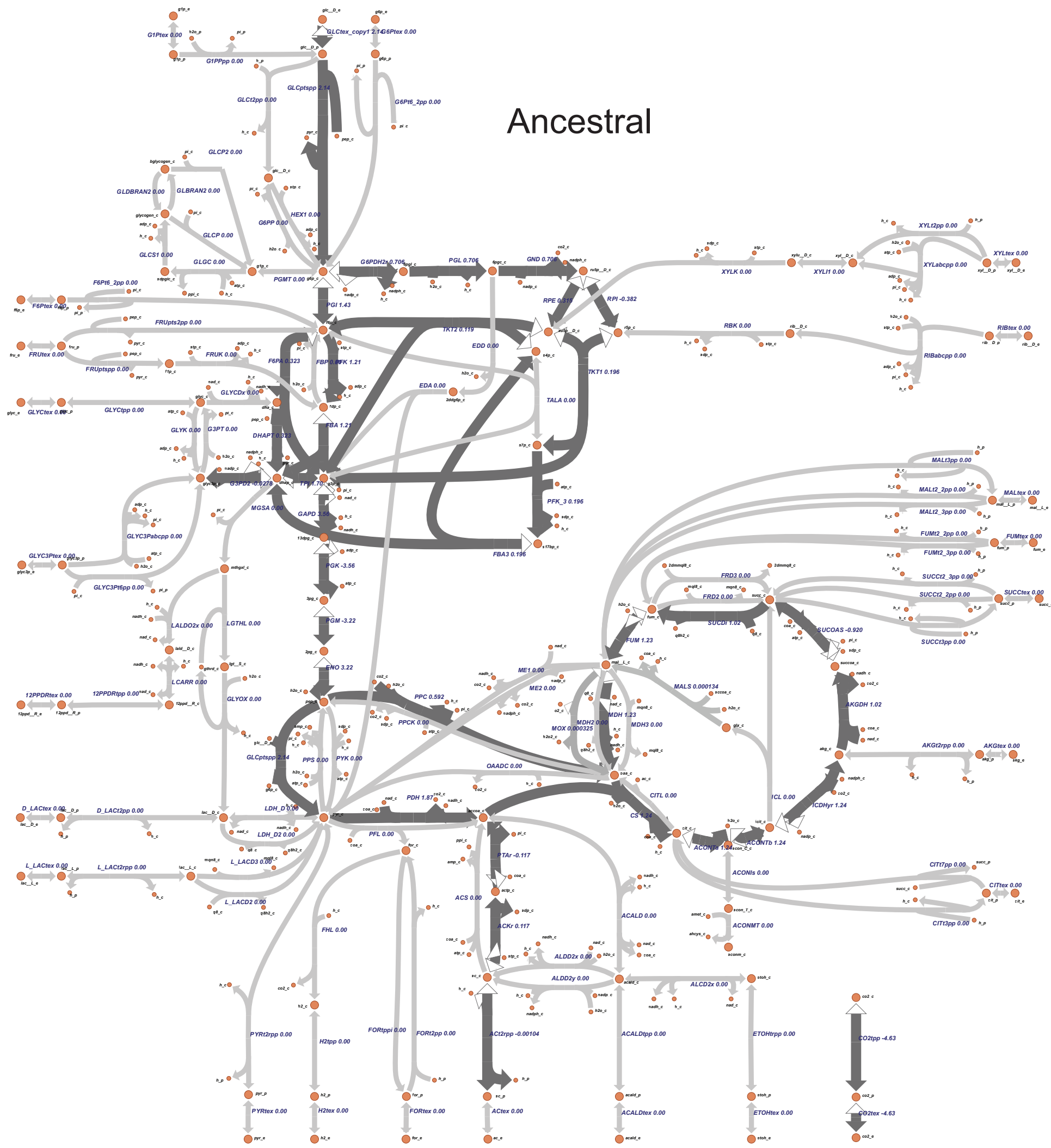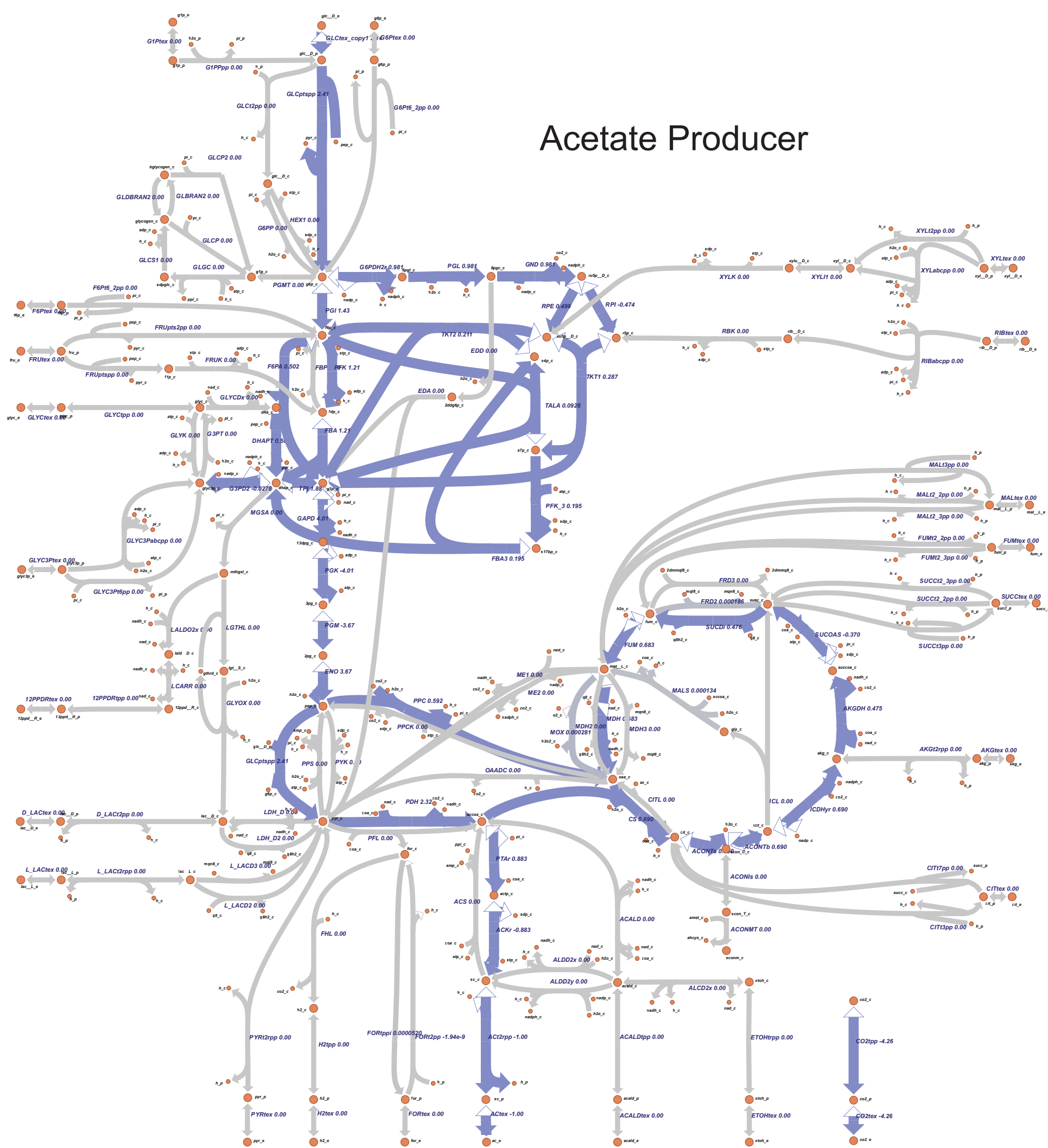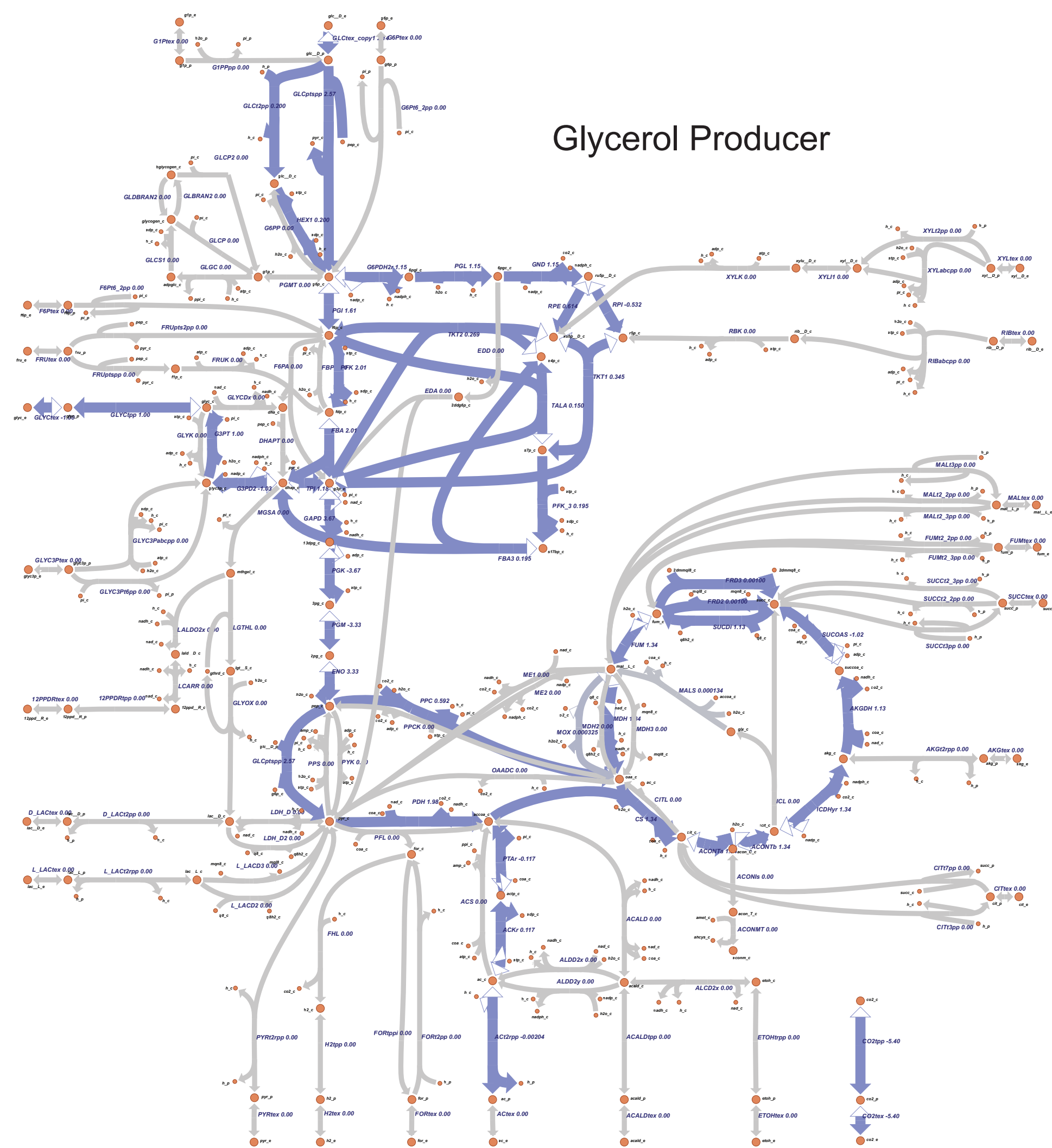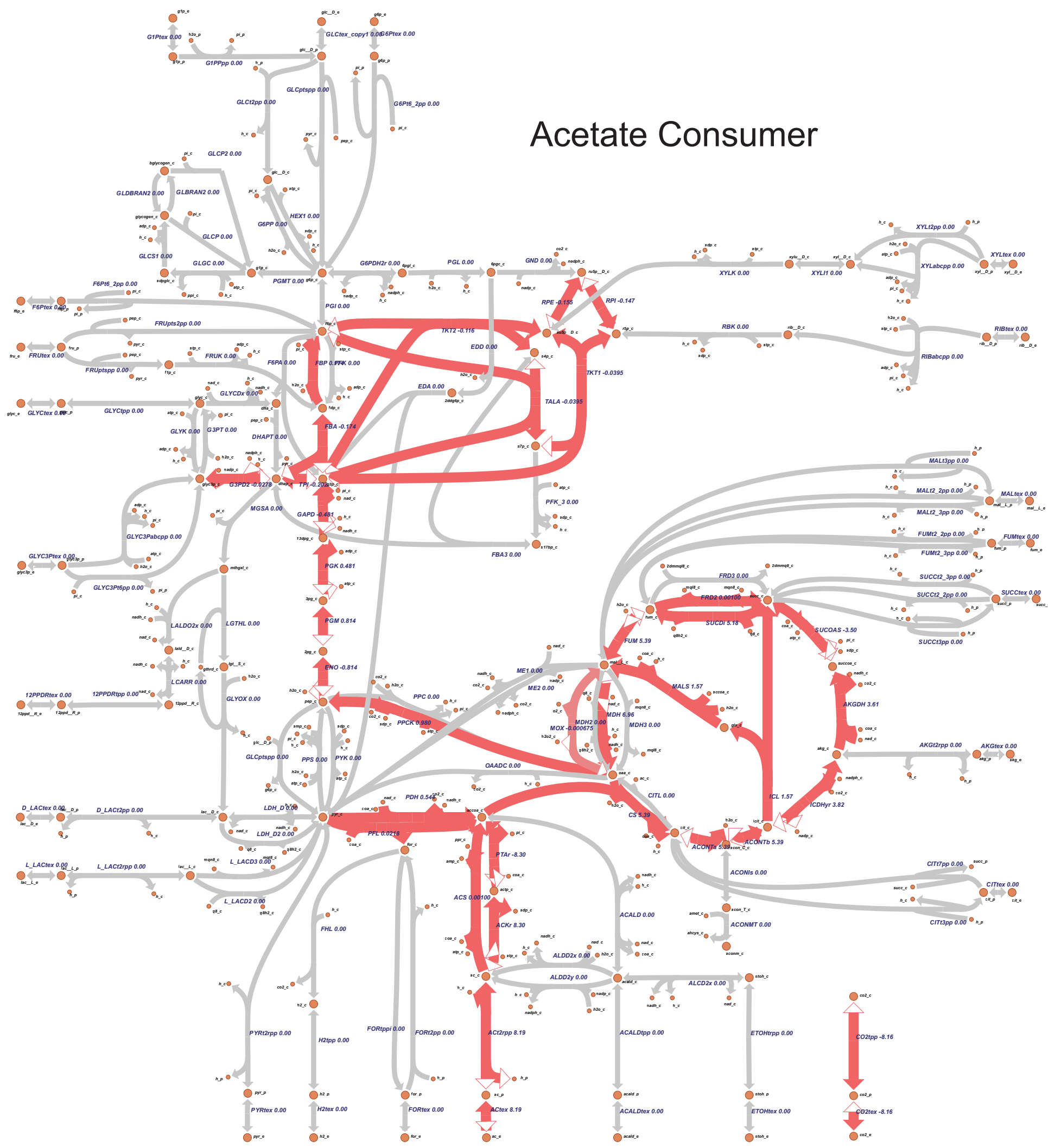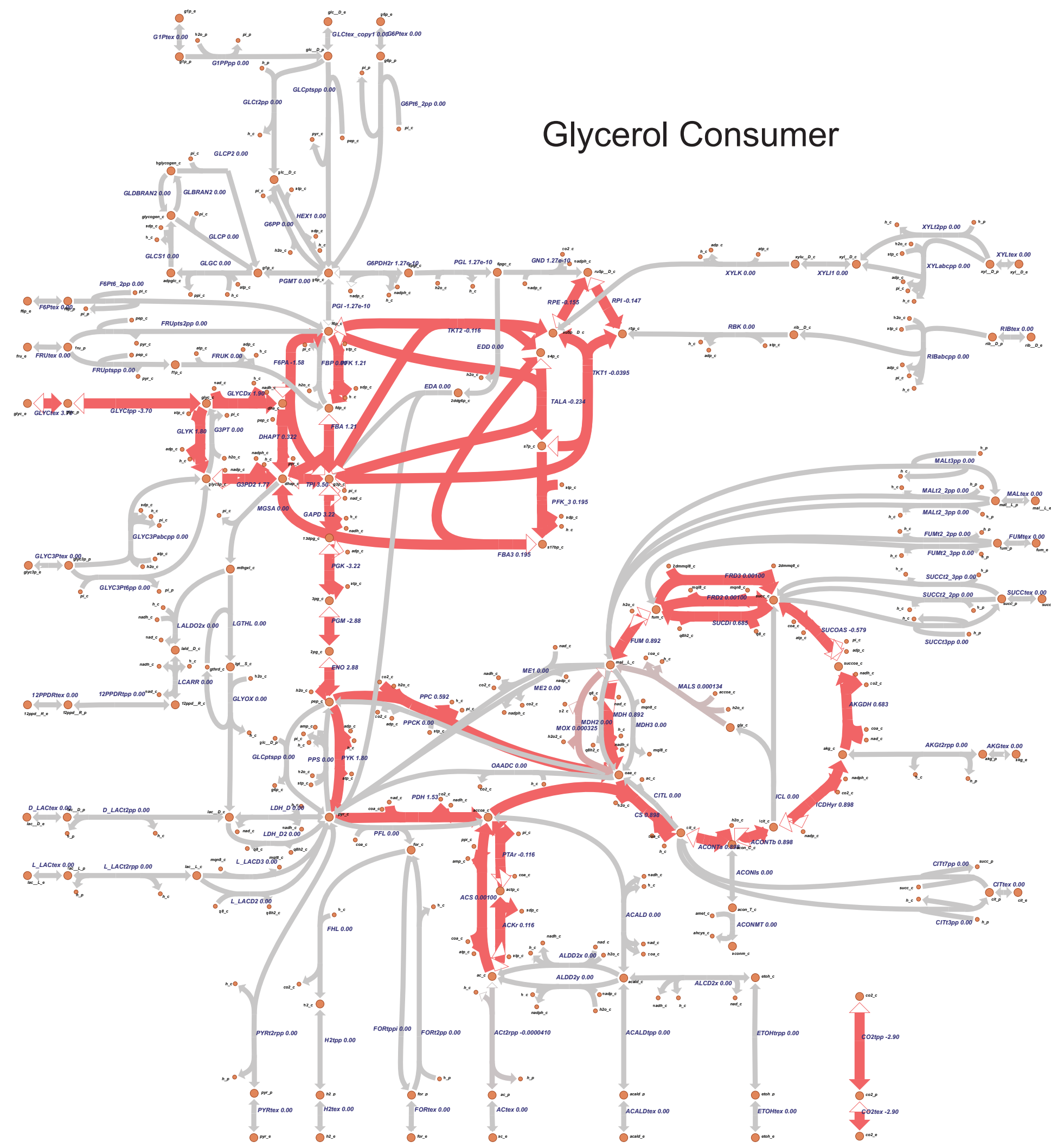

Supplement: S1 Fig — Like Fig 1, the network corresponds to the central carbon metabolism of E. coli. Every orange circle represents a metabolite, and every line a reaction. Thick grey, blue and red lines indicate a non-zero flux in the ancestral, producer and consumer strains respectively. Unlike Fig 1, one can zoom into this figure to see metabolite names, reaction names, and flux values. (PDF) [file pcbi.1008433.s009.pdf]

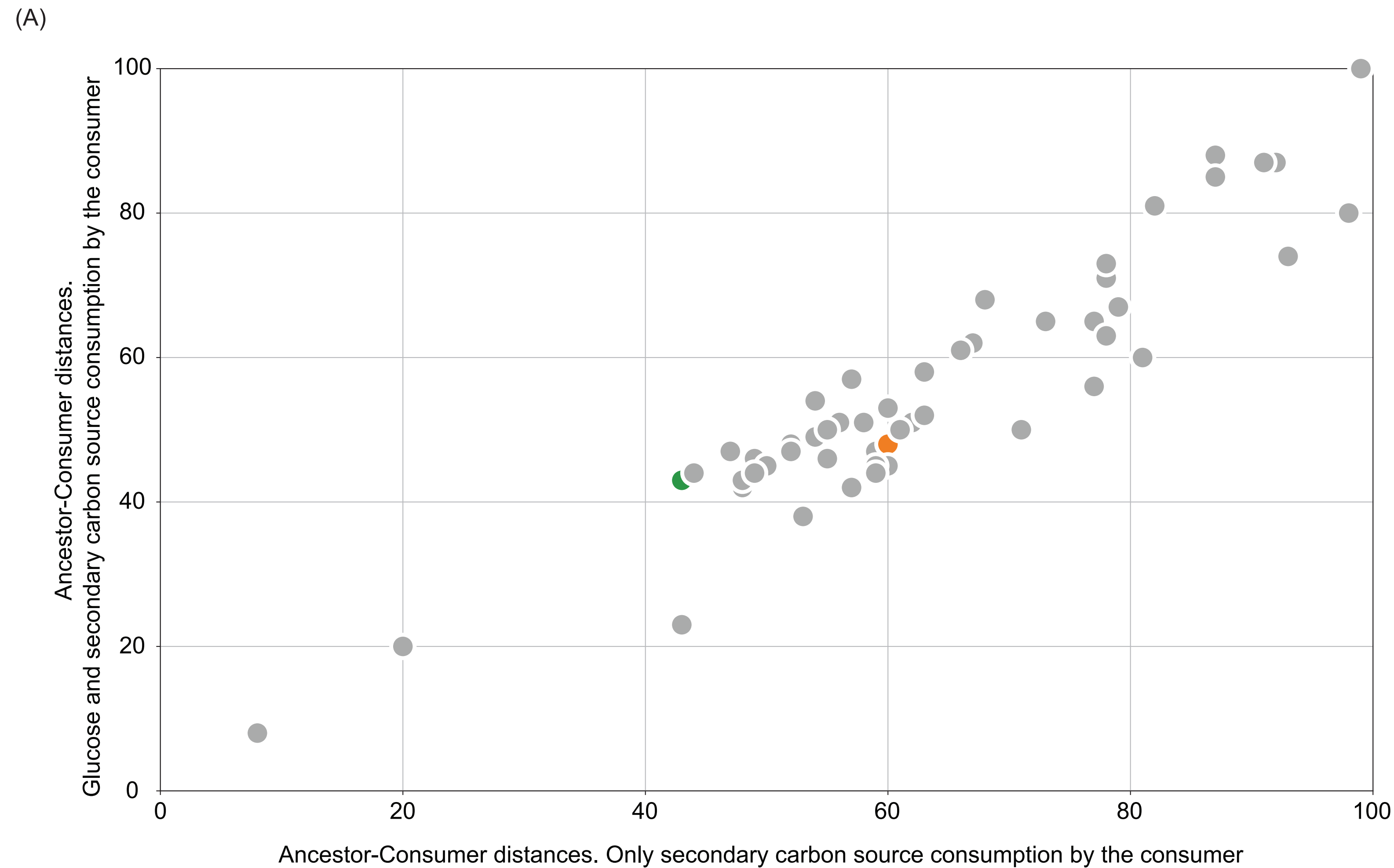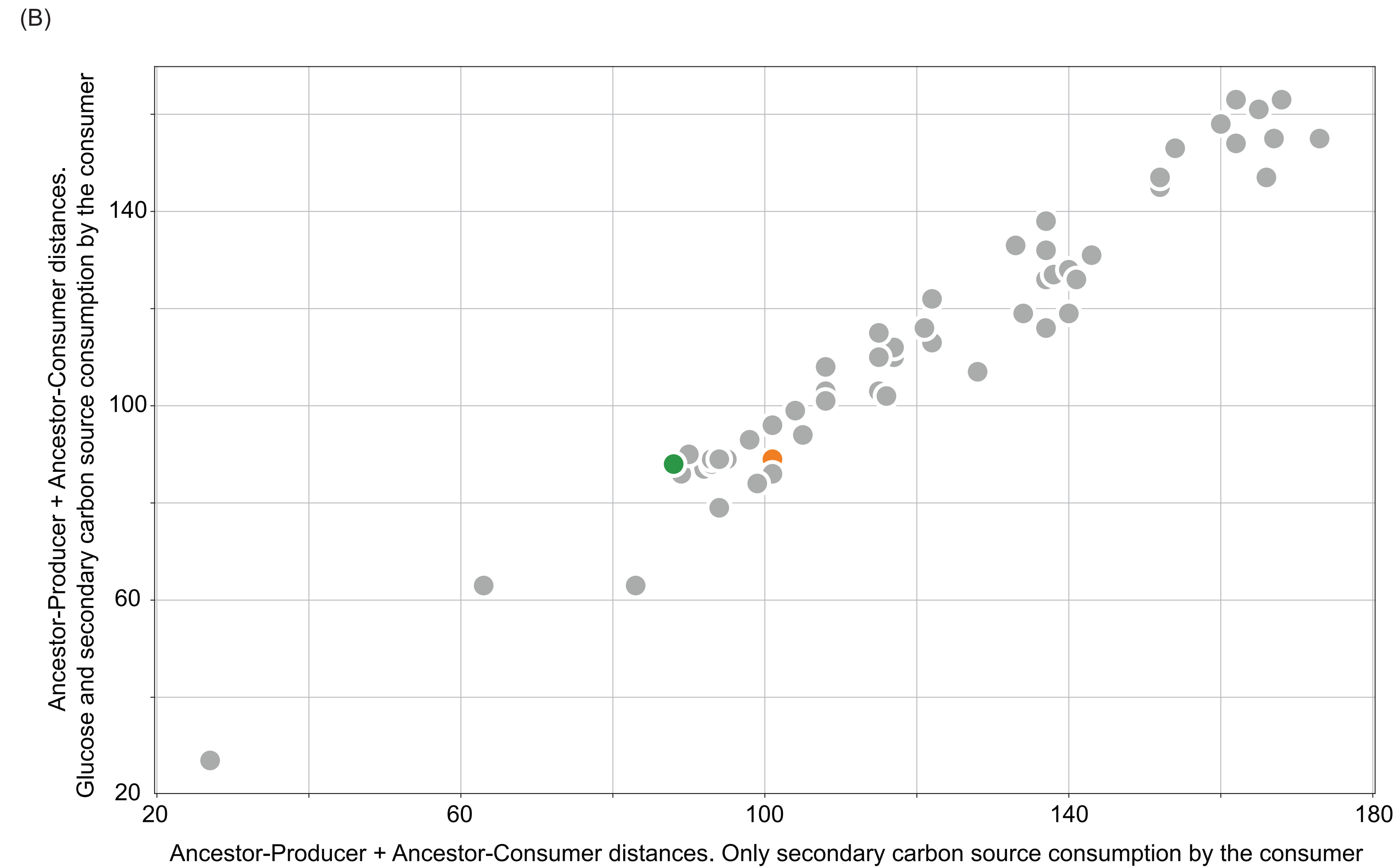

Supplement: S2 Fig — Comparison of ancestor-consumer distances and B) the sum of ancestor-producer and ancestor-consumer distances when the consumer strains consume (A) only the specific secondary carbon source or (B) both glucose and the secondary carbon source. The x-axes show the ancestor-consumer and total distances obtained when the consumer strains cannot consume glucose but only the specific secondary carbon source (see also Fig 2A and 2B). The y-axes show the same distances but obtained when the consumer strain consumes 1 mmol gDW-1 h-1of glucose and the respective secondary carbon source in amounts that allow growth at 0.2 h-1. The ancestor-producer distances used to calculate the total distances shown in (B) are those shown on the x-axis of Fig 2A. Every grey circle represents one of 56 metabolites that can be cross-fed. Acetate and glycerol are shown as orange and green circles, respectively. Even though the ancestor-consumer distances change when the consumer strains consume glucose in addition to their specific secondary carbon source, the main conclusion of this work do not change: Multiple changes are required for any cross-feeding interaction to evolve, and cross-feeding of multiple metabolites may evolve with higher likelihood than acetate and glycerol cross-feeding. (PDF) [file pcbi.1008433.s010.pdf]

(A)

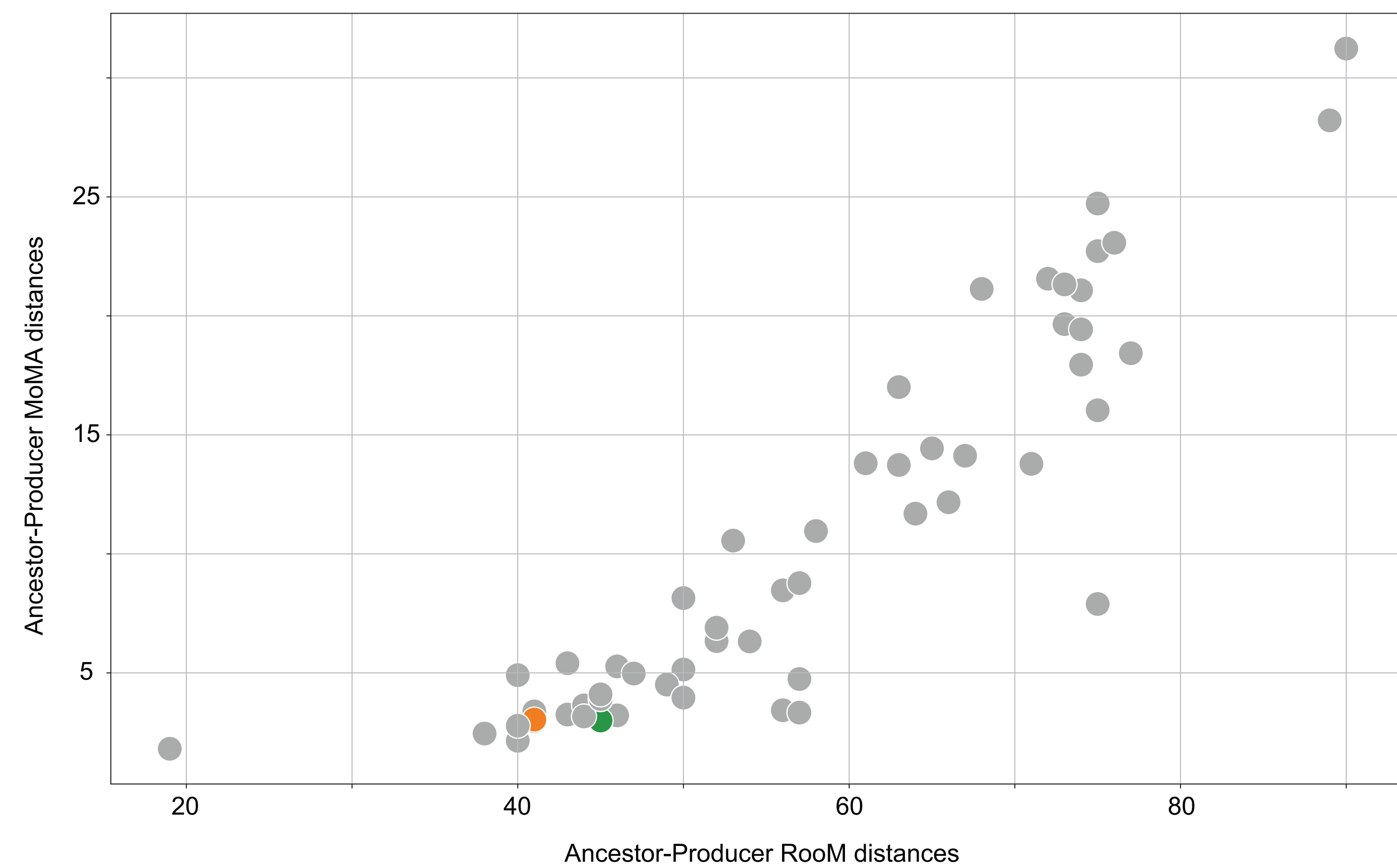

(B)

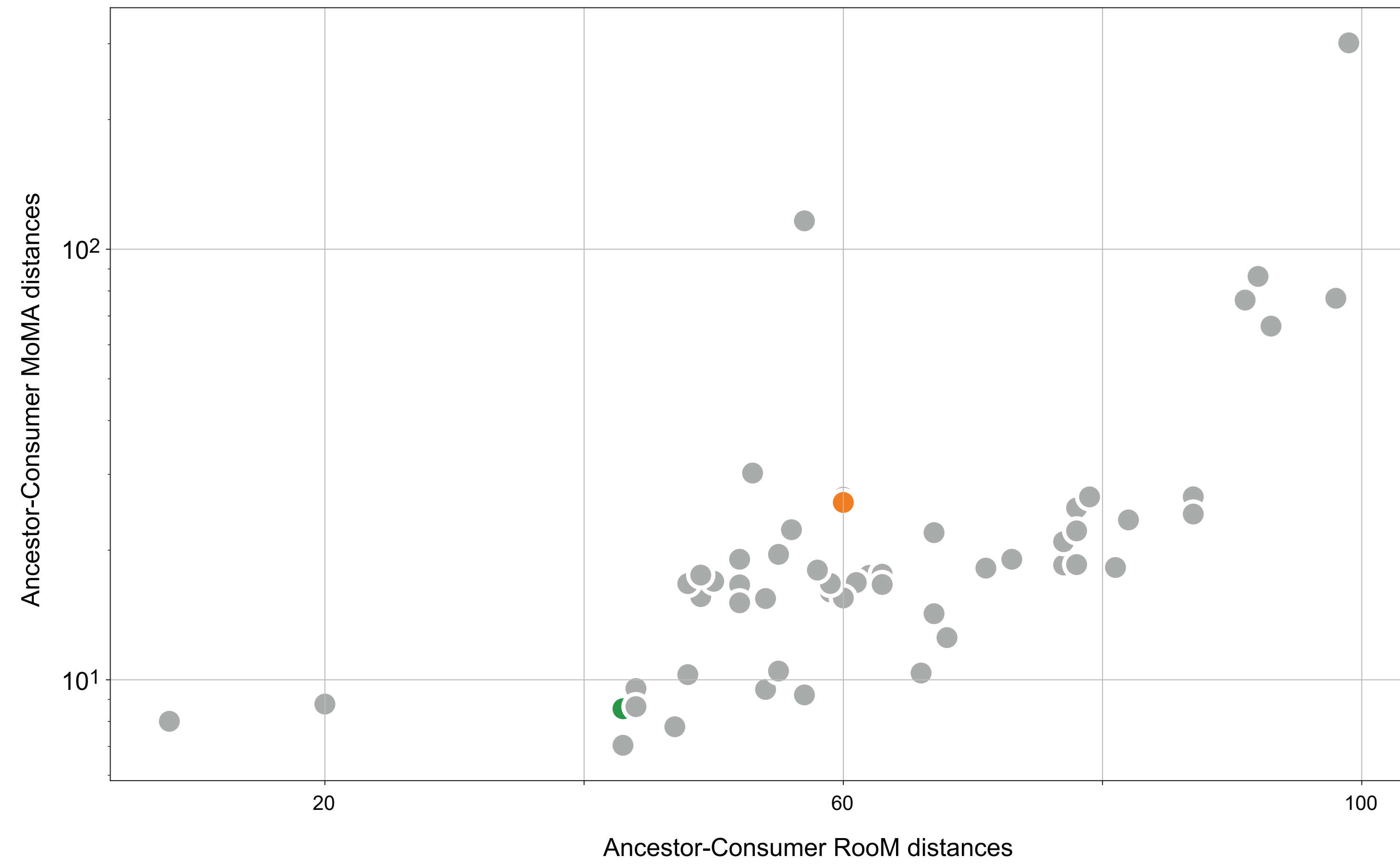

(C)

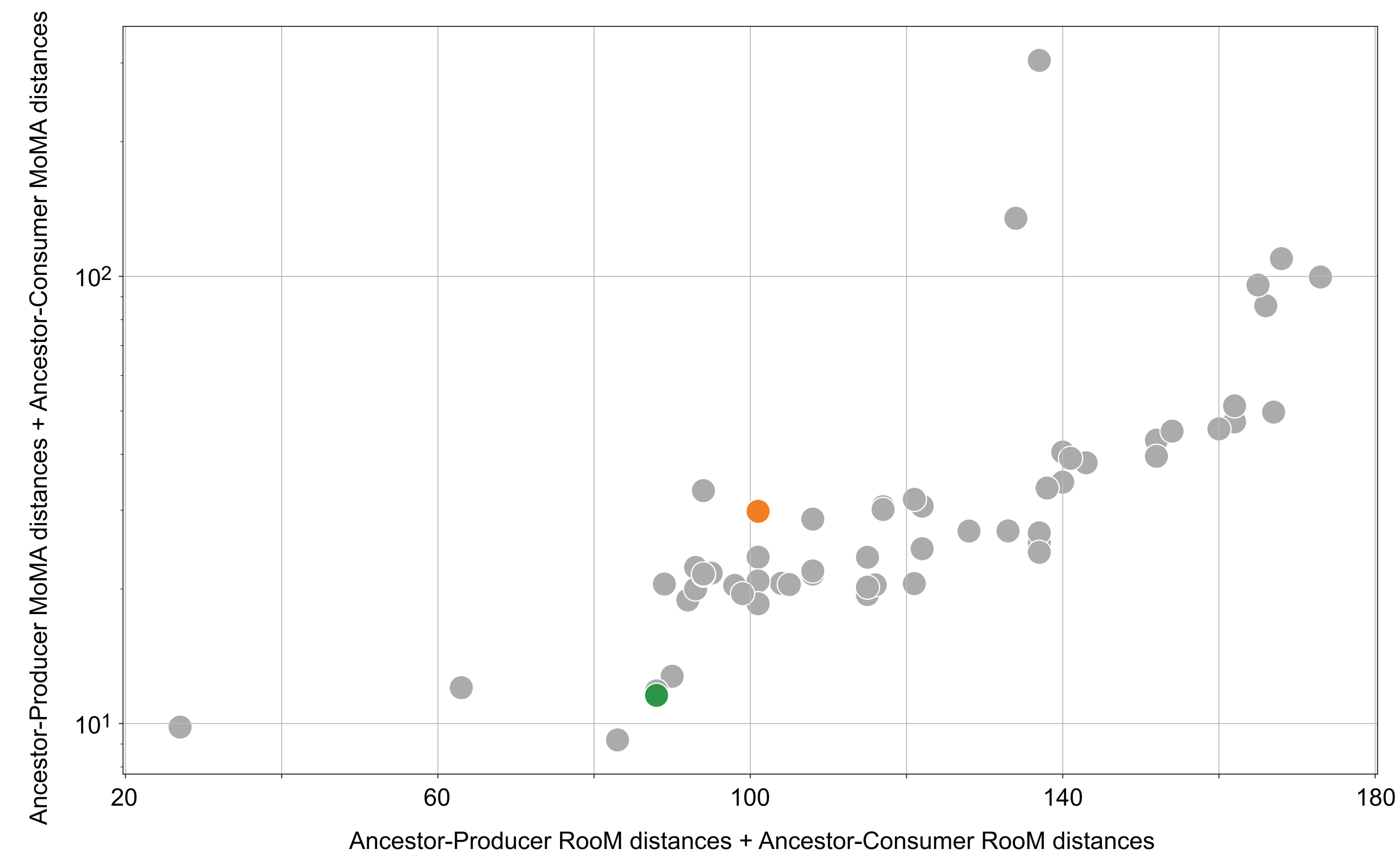

(D)

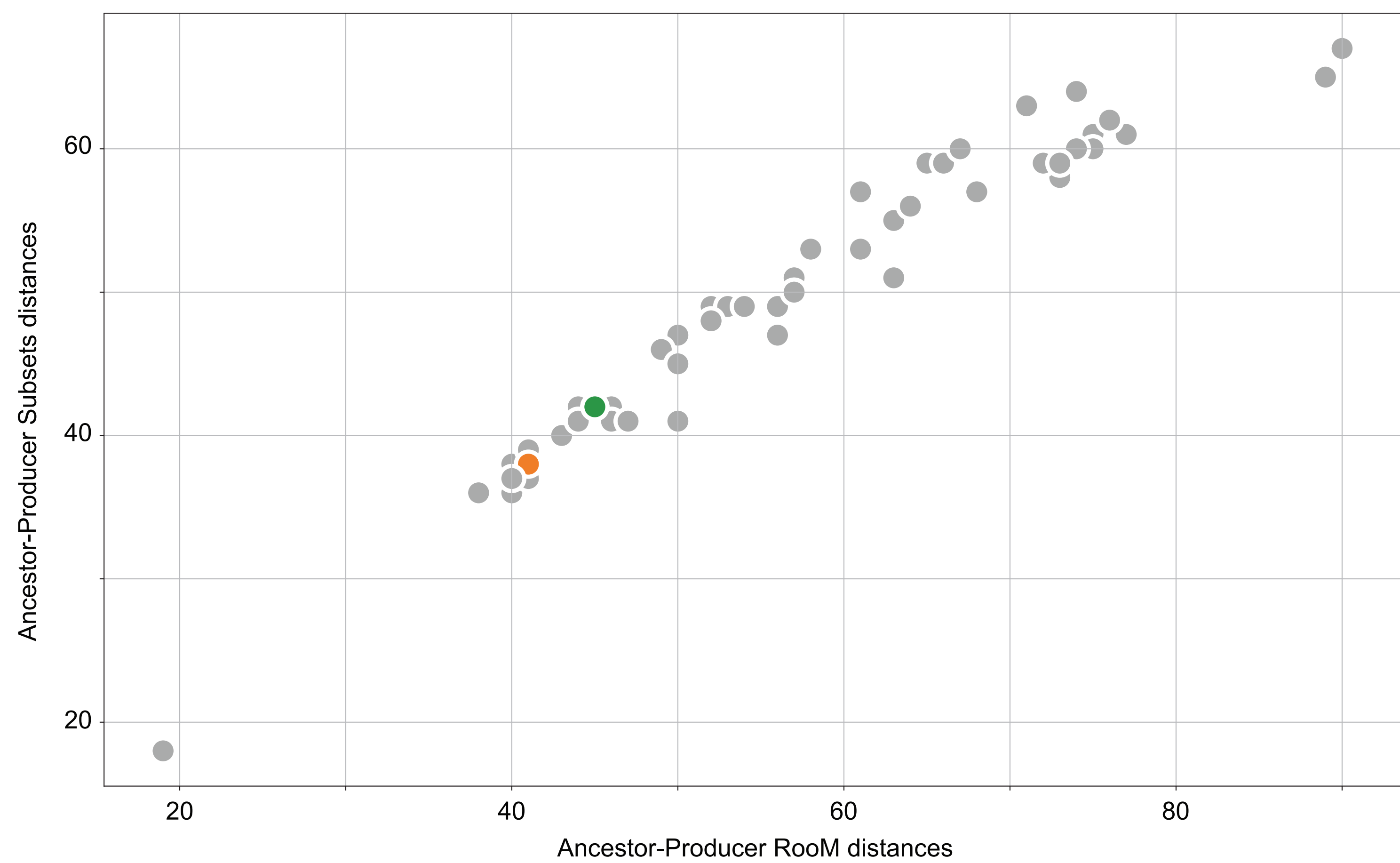

(E)

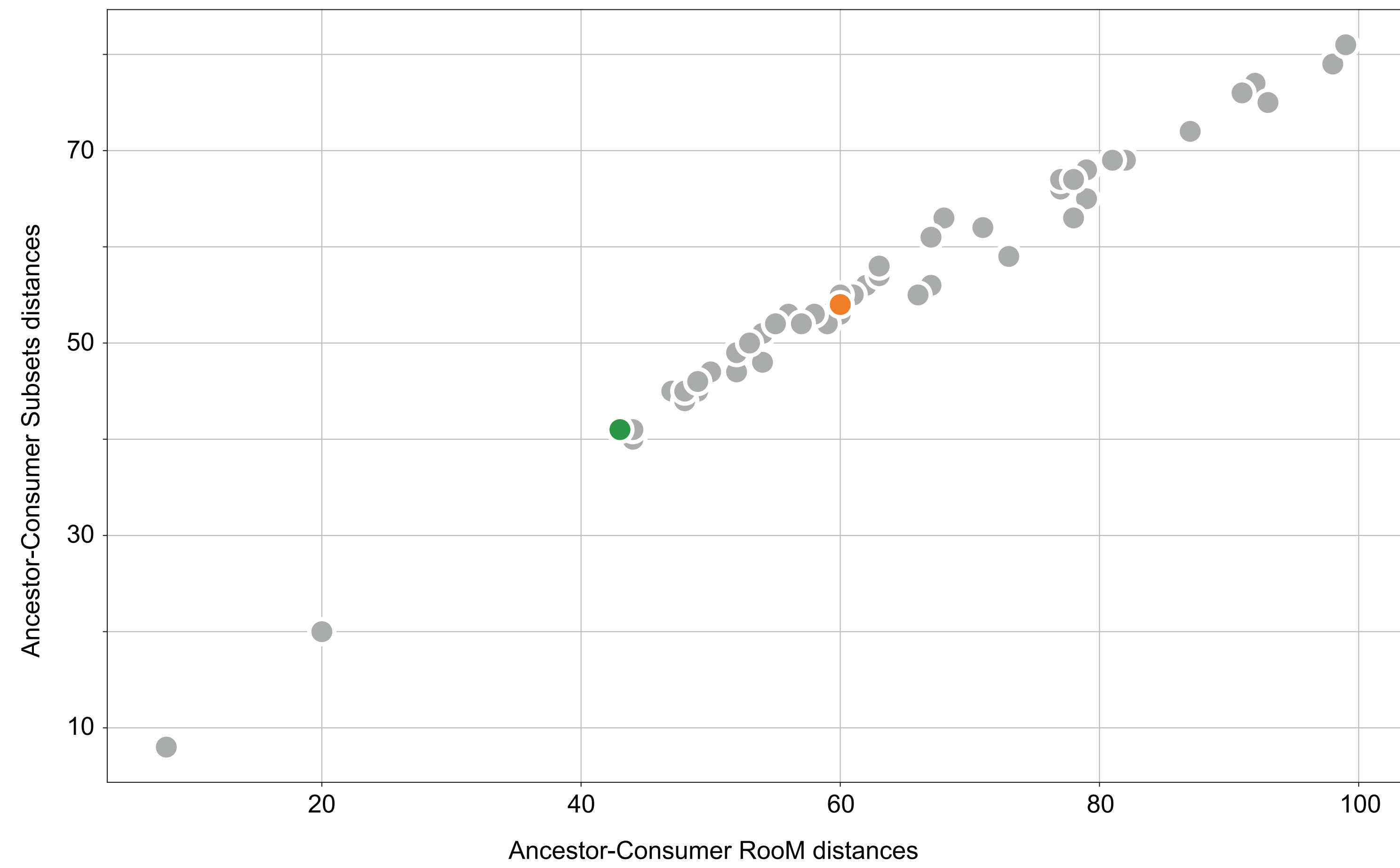

(F)

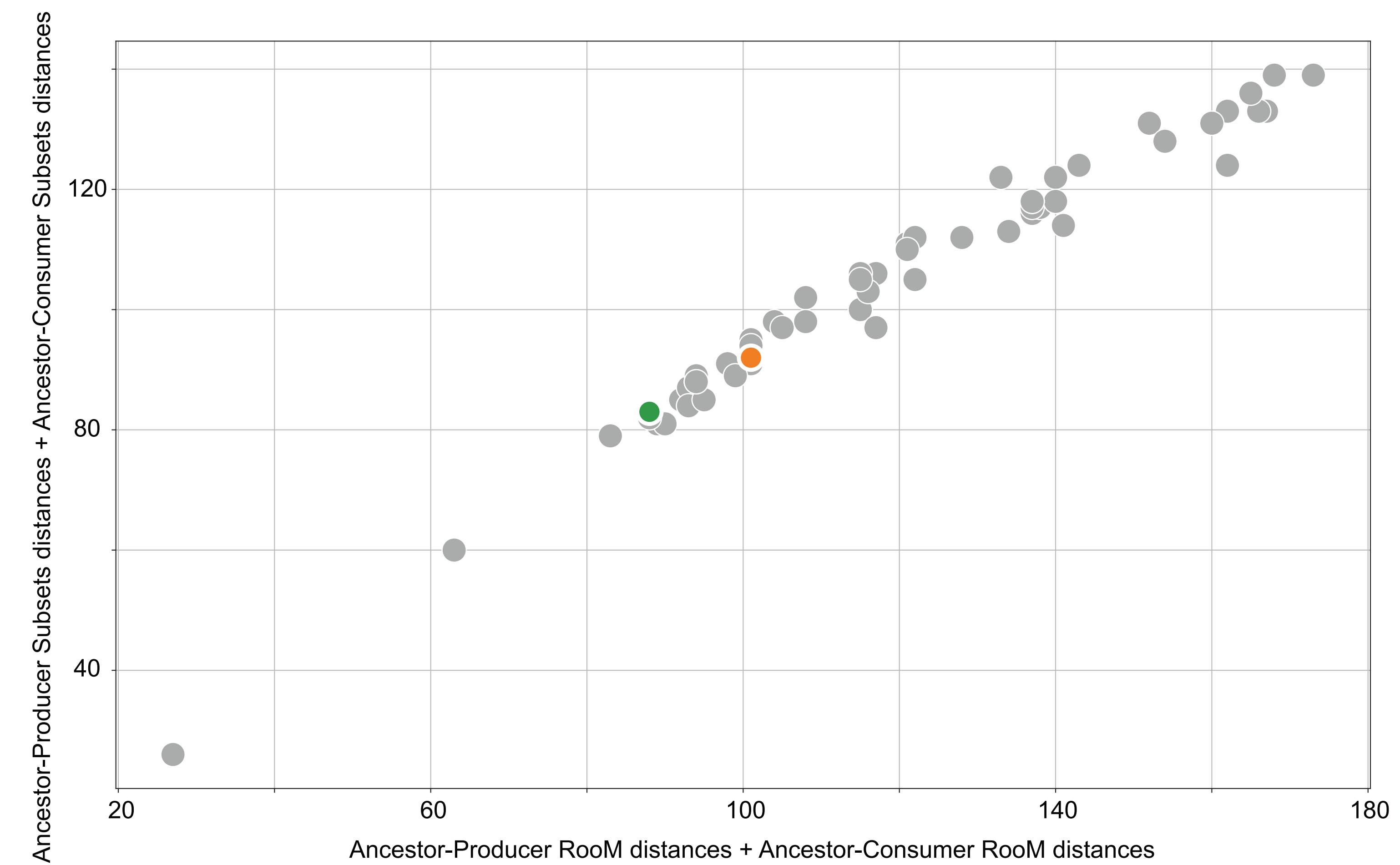

Supplement: S4 Fig — In figures (A) to (C) flux distribution distances predicted by MoMA (on the y-axis) are compared with distances predicted by RooM (on the x-axis). The ancestor-producer distance, the ancestor-consumer distance, and the total distance (sum of ancestor-producer and ancestor-consumer distances) are shown in panels (A) to (C), respectively. Every grey circle represents one of 56 metabolites that can be cross-fed. Acetate and glycerol are shown as orange and green circles. Panels (D) to (F) are analogous to (A) to (C), but their y-axes show the distances predicted when minimizing the number of co-regulated reaction subsets that change expression. (PDF) [file pcbi.1008433.s012.pdf]

(A)

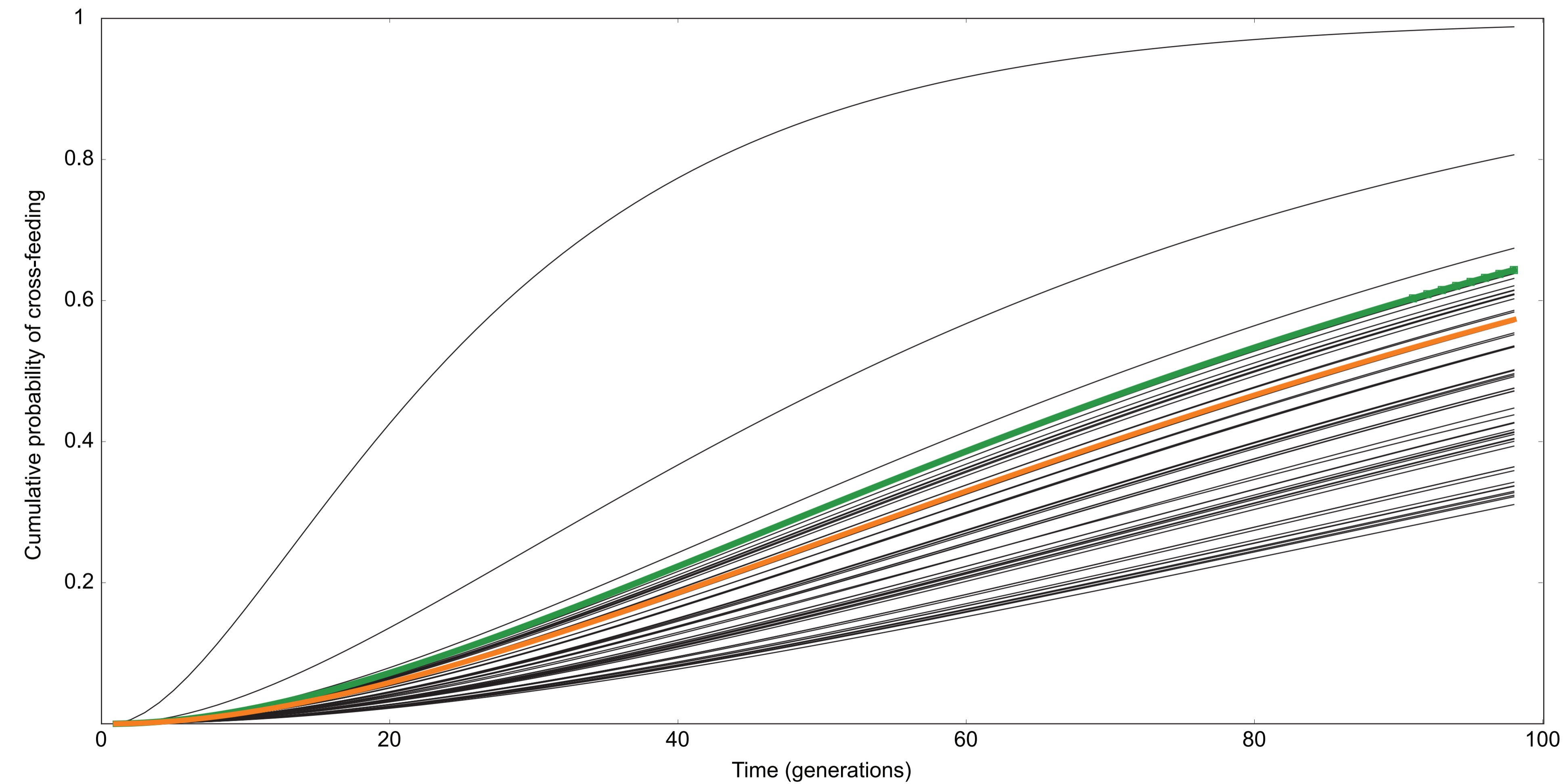

(B)

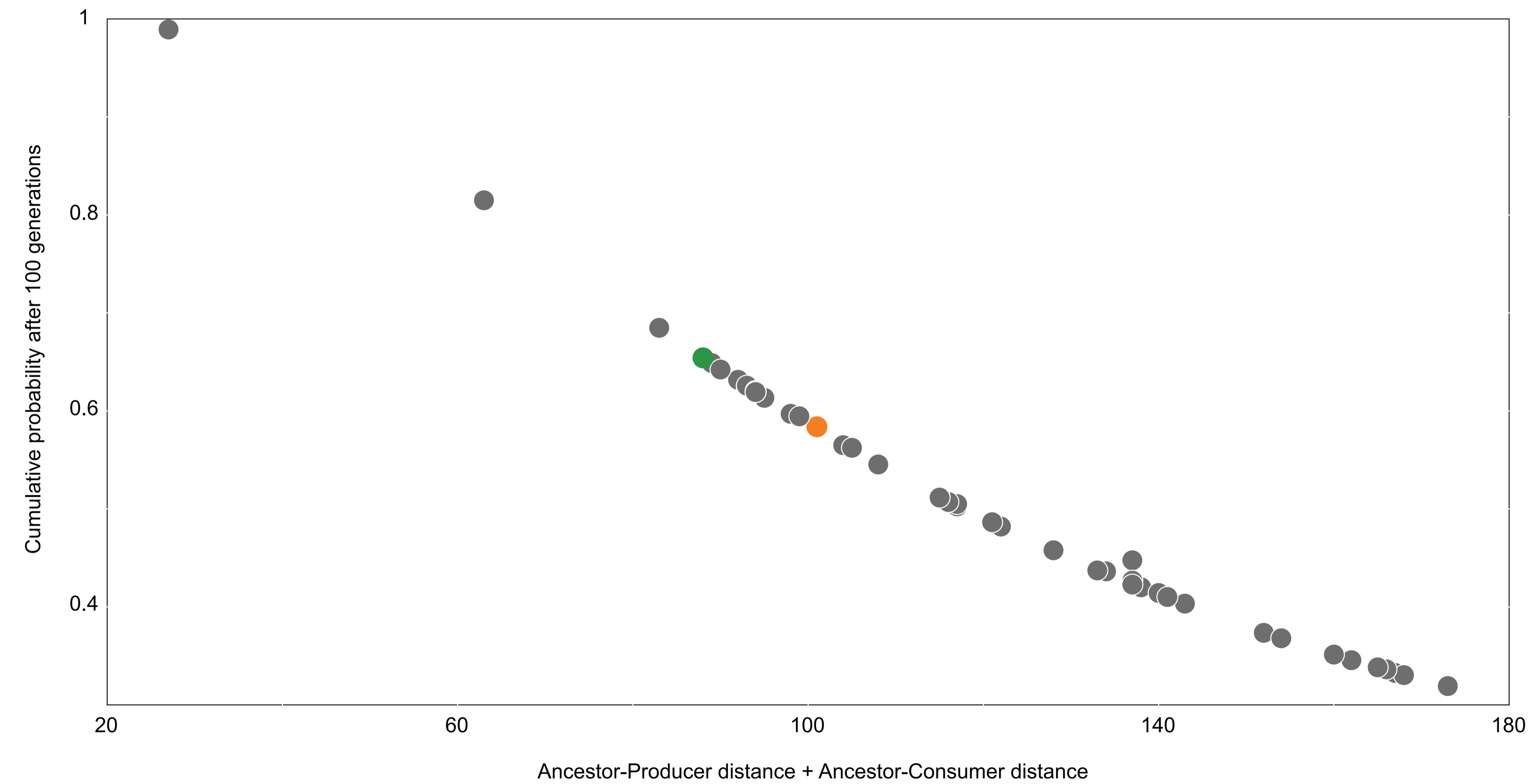

Supplement: S5 Fig — (A) The cumulative probability for the evolution of cross-feeding interactions as calculated with Eq (2) from S5 Text is plotted against time (in generations). Every grey line corresponds to a prediction for a different metabolite subject to cross-feeding. Predictions for acetate and glycerol are shown in orange and green, respectively. (B) Comparison of two proxies of the likelihood that cross-feeding evolves. The x-axis shows the sum of the producer-ancestor and consumer-ancestor distances, which is the proxy used in the main text. The y-axis shows the cumulative probability of cross-feeding to evolve after 100 generations, according to the model from S5 Text, which takes into consideration that the evolution of producer and consumer may not be independent events. Every grey circle represents a prediction for a different metabolite subject to cross-feeding. Orange and green circles correspond to predictions for acetate and glycerol, respectively. The two proxies for the likelihood to evolve cross-feeding are highly correlated (Spearman’s r = 0.99, P = 9.7e-74, n = 58). (PDF) [file pcbi.1008433.s013.pdf]

(A)

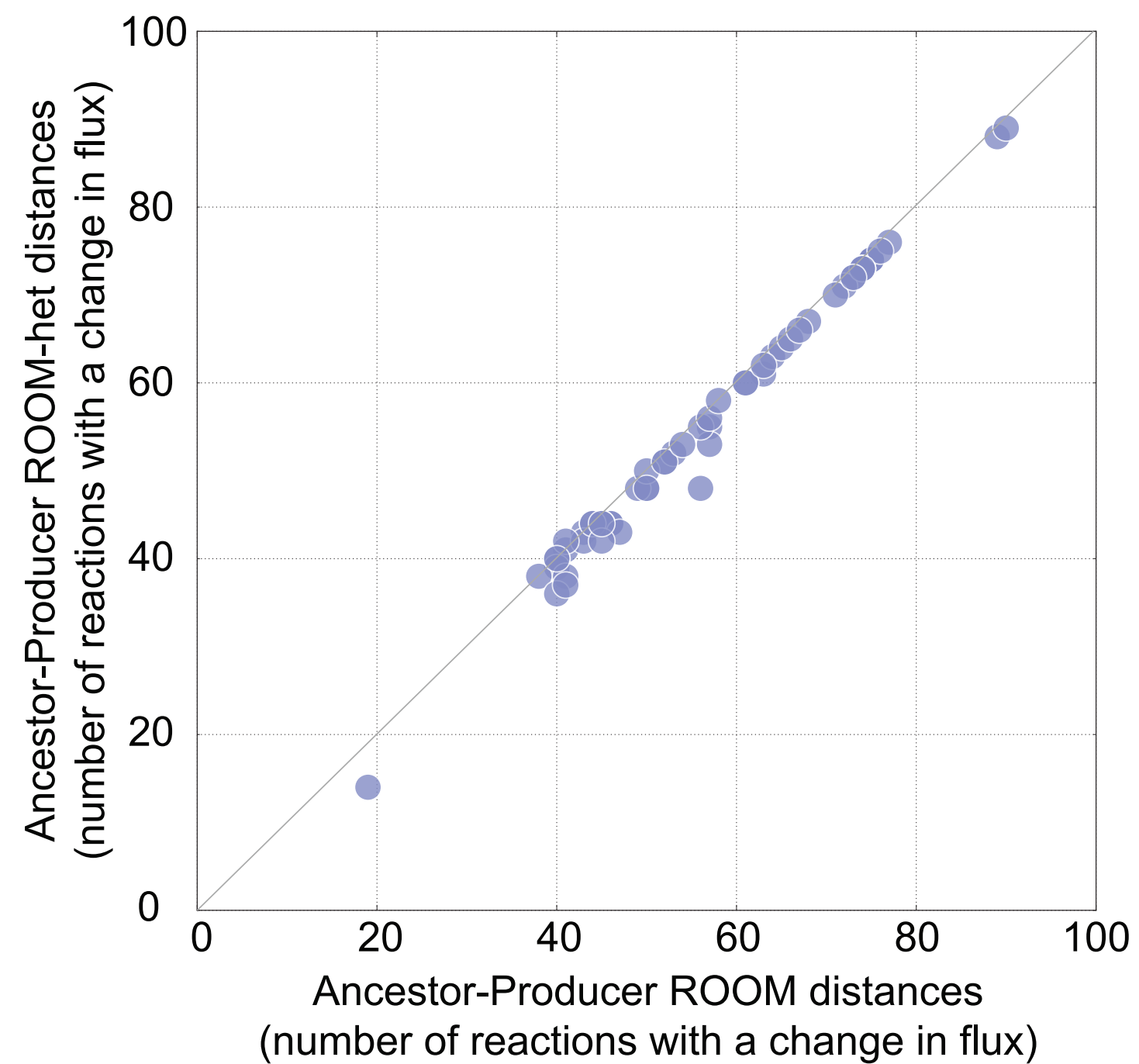

(B)

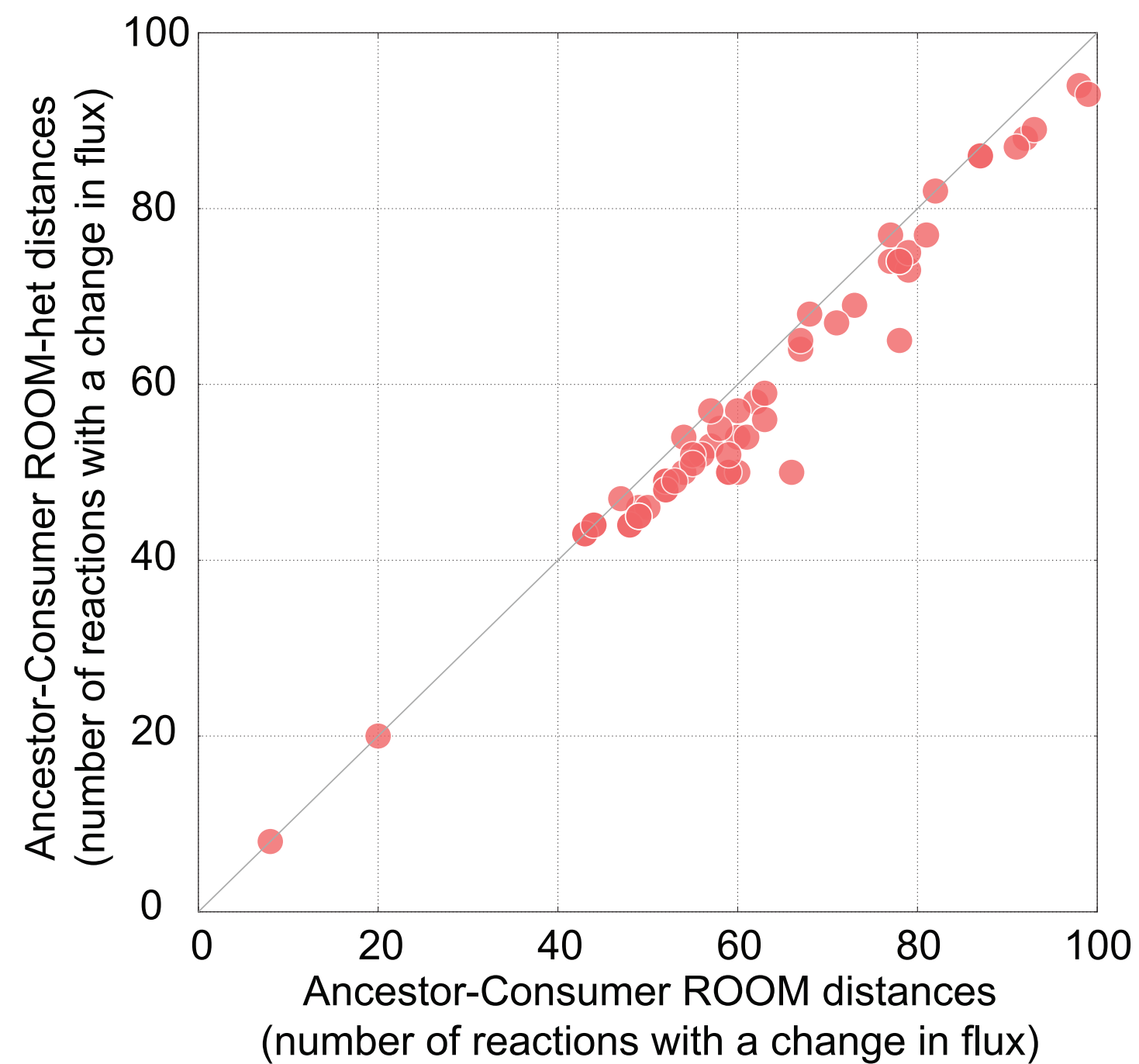

Supplement: S6 Fig — (A) Ancestor-producer distances predicted with RooM and RooM-het are shown on the x and y-axes respectively. Every circle corresponds to one produced metabolite. The diagonal line indicates equal distances. (B) As in (A) but for predicted ancestor-consumer distances and for consumed metabolites. (PDF) [file pcbi.1008433.s014.pdf]

(A)

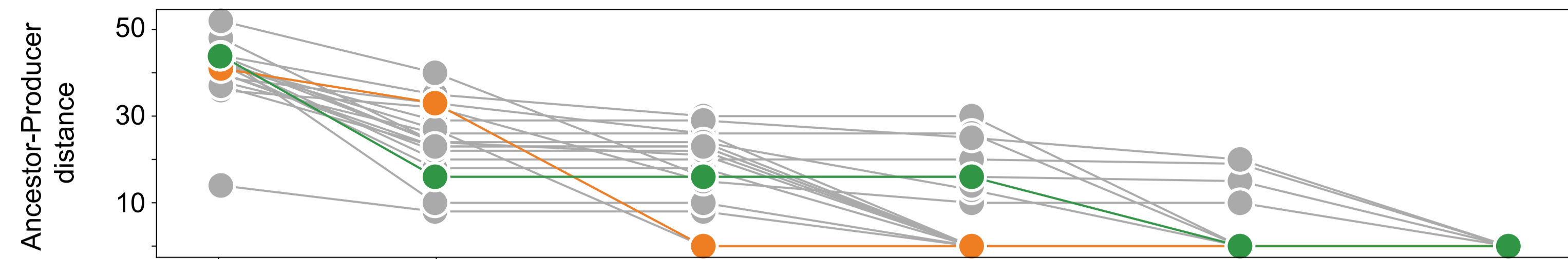

(B)

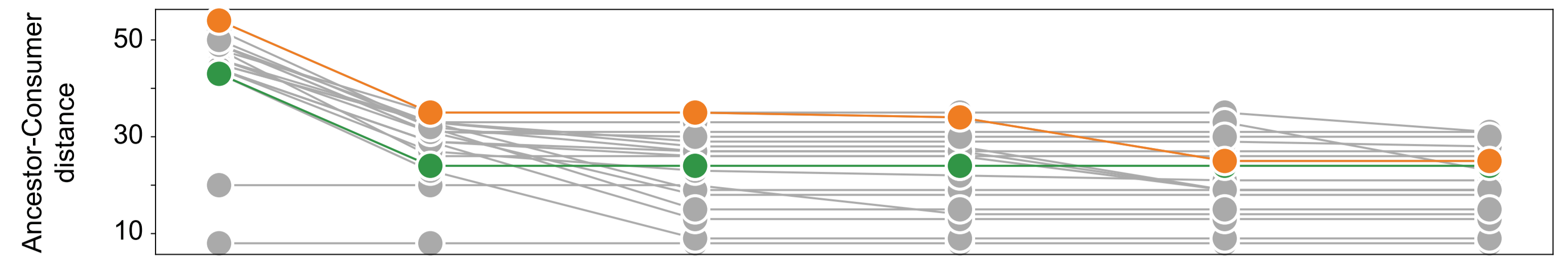

(C)

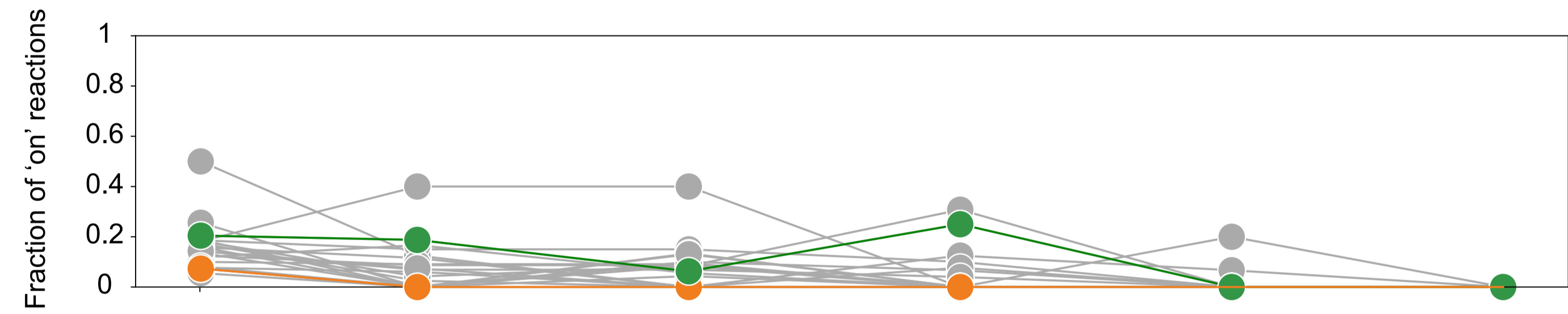

(D)

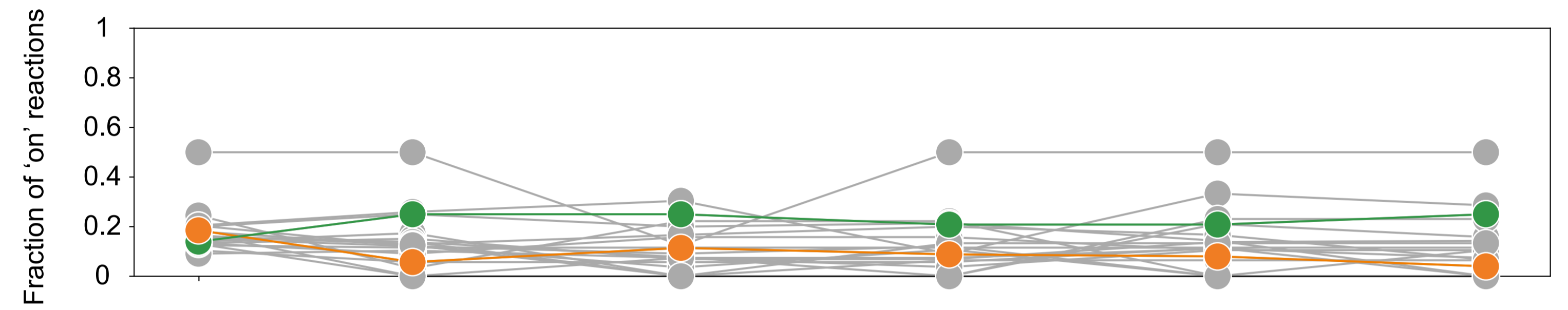

(E)

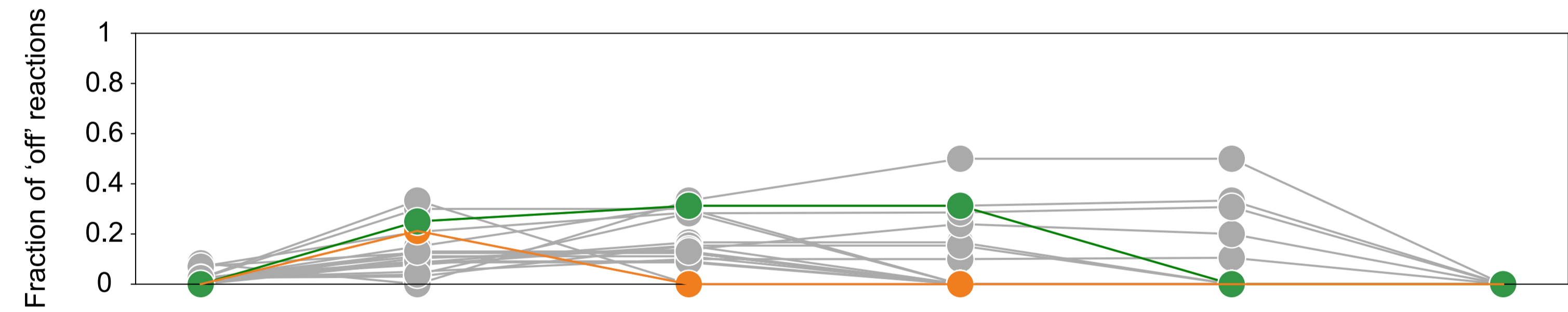

(F)

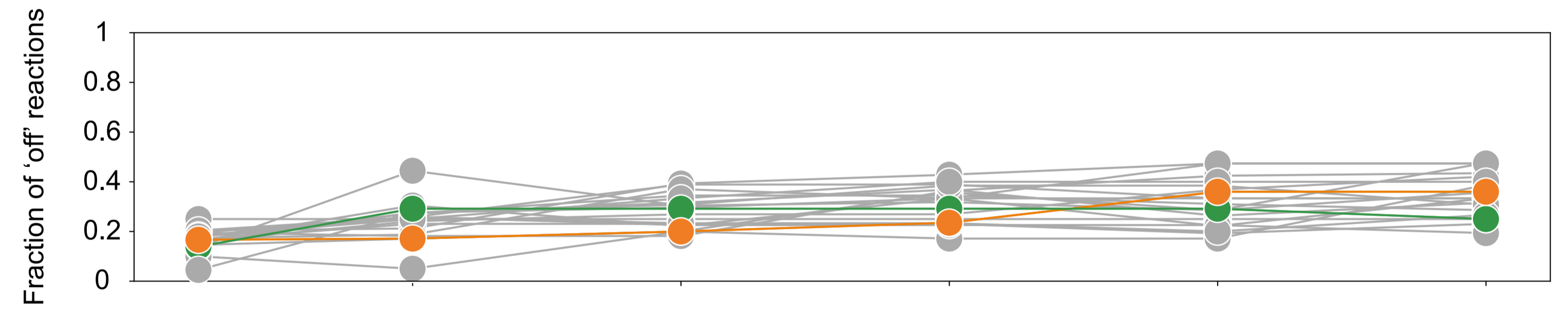

(G)

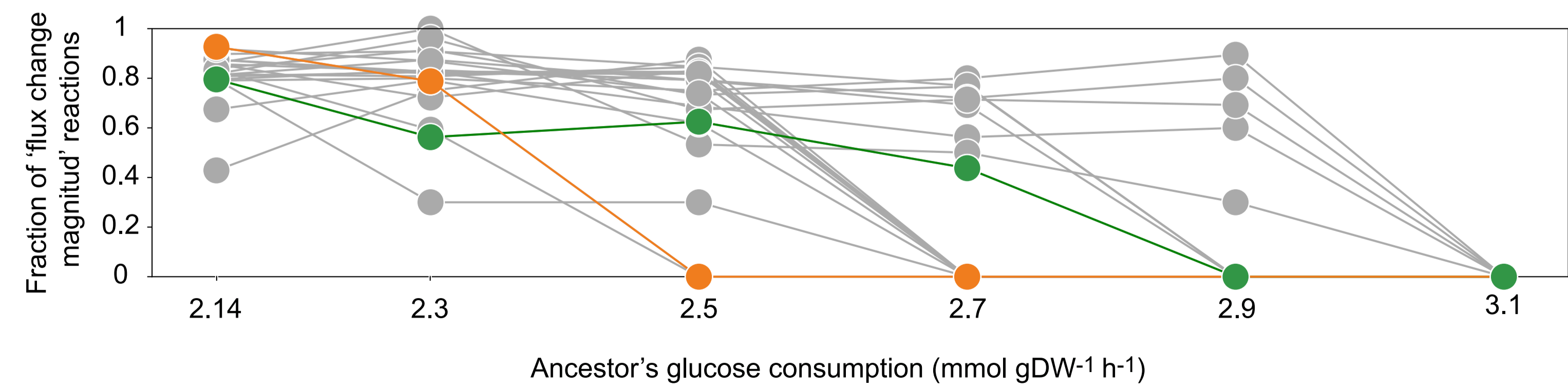

(H)

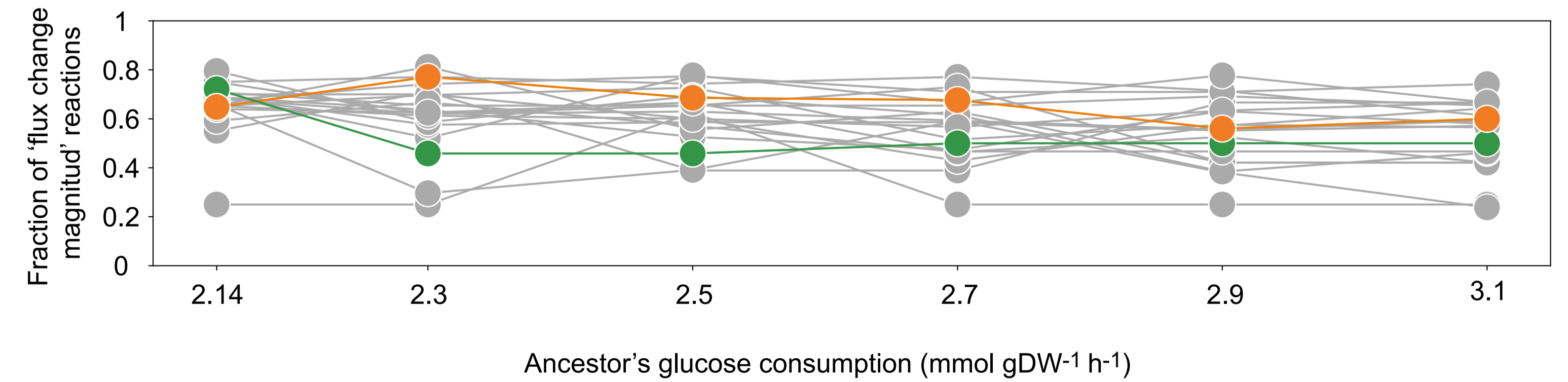

Supplement: S7 Fig — As a function of this quantity, (A) and (B) show the predicted distance of the ancestor to the producer and consumer, respectively. Among all reactions with a change in flux, (C), (E) and (G) show the fraction of reactions that are turned ‘on’, ‘off’ and that change the flux quantitatively in the producer relative to the ancestor, respectively; (D), (F) and (H) are analogous to (C), (E) and (G), but for the reactions that require a flux change in the consumer relative to the ancestor. Each set of six grey circles connected by a grey line corresponds to simulation data for one of the twenty metabolites with a predicted likelihood of being subject to evolve cross-feeding greater than that for acetate when either RooM or RooM-het are performed at the minimal glucose consumption rate of 2.14 mmol gDW-1 h-1. Predictions for acetate and glycerol are shown as orange and green circles, respectively. (PDF) [file pcbi.1008433.s015.pdf]

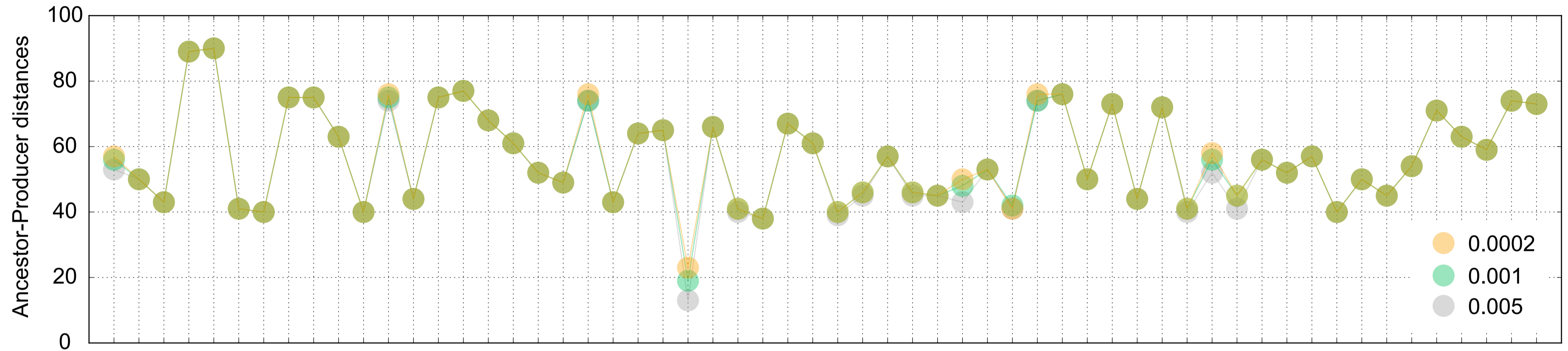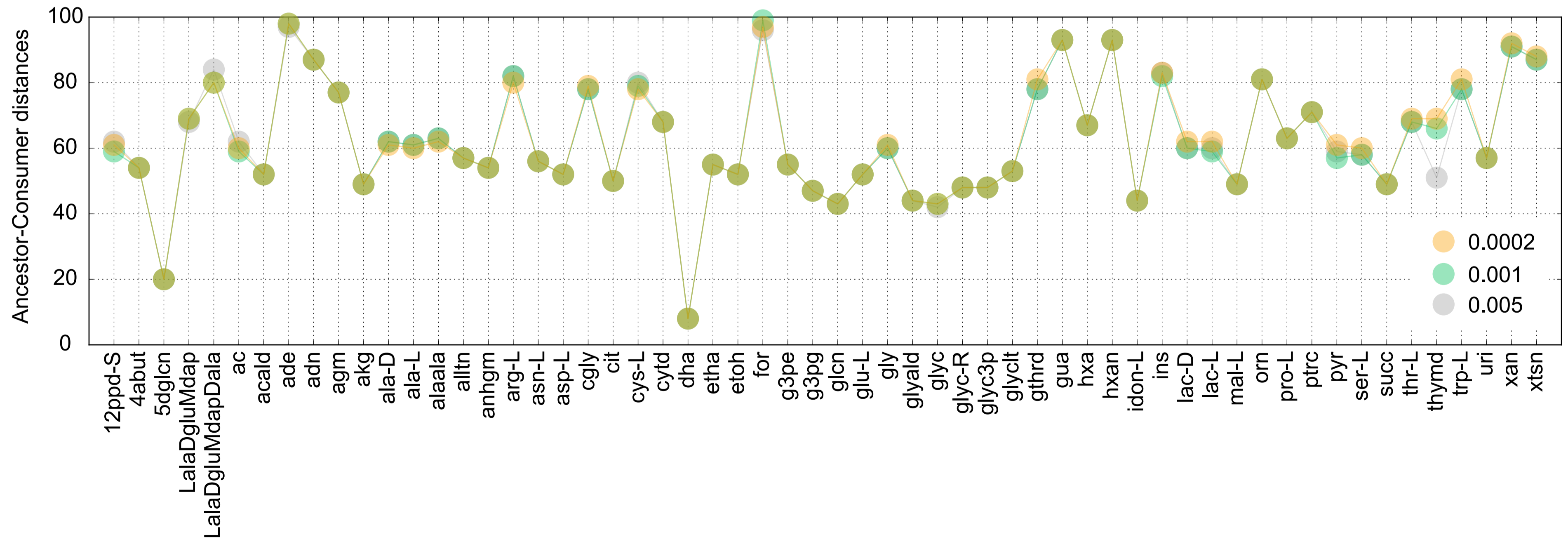

Supplement: S8 Fig — The data shows that changing beta from its default value (0.001 mmol gDW-1 h-1, green circles) to a fifth of this value (0.0002 mmol gDW-1 h-1, yellow circles), or to five times this value (0.005 mmol gDW-1 h-1, grey circles) has very little effect on the predicted distances. (PDF) [file pcbi.1008433.s016.pdf]
