# Supplementary material for: Acetate and glycerol are not uniquely suited for the evolution of cross-feeding in E. coli
Source: PLoS Comput Biol. 2020 Nov 30;16(11):e1008433. doi: 10.1371/journal.pcbi.1008433 (PMC7728234; doi:10.1371/journal.pcbi.1008433)
Supplement: S3 Fig — The light grey areas in figures (A) to (E) show the number of genes found to be up-regulated, down-regulated, or unchanged in expression for the experimentally observed acetate producer CV103, the acetate consumer CV101, the glycerol producer CV103, and the two glycerol consumer strains CV115 and CV116. The numbers above the grey bars add up to the total number of genes included in the metabolic model of E. coli iJO1366 (1367). Green and orange bars show the genes predicted to be up-regulated, down-regulated or unchanged, based on flux changes for reactions associated with these genes, as predicted by (from left to right) RooM, MoMA, minimizing reaction subsets, and RooM-het. Green bars (overlapping the grey area) indicate the number of genes correctly predicted to be up-regulated, down-regulated, or unchanged (true positives). Orange bars indicate the number of genes computationally predicted but not experimentally observed to be up-regulated, down-regulated, and unchanged (false positives). (F) Summarizes the data shown in (A) to (E). For each strain (rows) and each gene category and prediction method (columns), the two numbers separated by a dash indicate the number of true positives and false positives. ‘*’ indicates p<0.05, and ‘**’p<0.01, based on a Fisher’s exact test of the null-hypothesis that the number of genes correctly predicted to be up-regulated, down-regulated, or unchanged can be attributed to chance alone. (PDF) [file pcbi.1008433.s011.pdf]

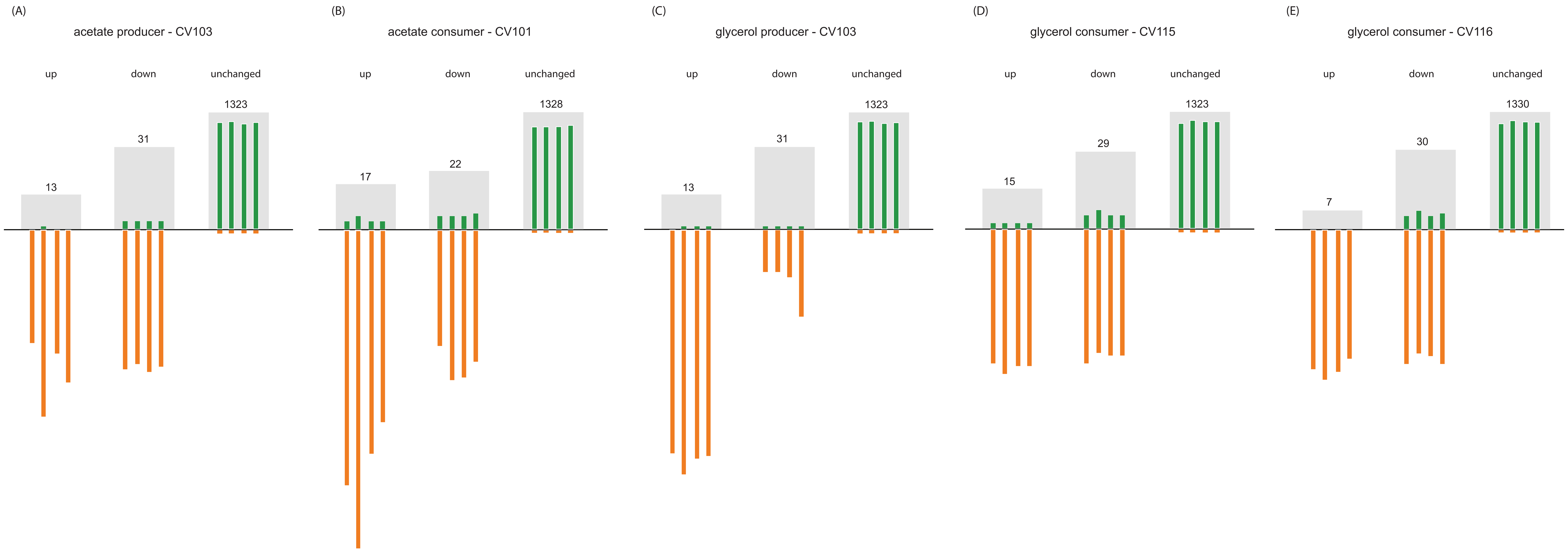

(F)

	up-regulated				down-regulated				unchanged			
	RooM	MoMA	subsets	RooM-het	RooM	MoMA	subsets	RooM-het	ROOM	MoMA	subsets	ROOM-het
acetate producer - CV103	0/43	1/71	0/47	0/58	3/53	3/51	3/54	3/52	1212/39	1223/37	1196/36	1213/38
acetate consumer - CV101	3/97	* 5/121	3/85	3/73	** 5/44	** 5/57	** 5/56	** 6/50	** 1164/27	** 1165/25	1167/38	** 1182/28
glycerol producer - CV103	0/85	1/93	1/87	1/86	1/16	1/16	1/18	1/33	1219/39	1226/37	1201/37	* 1210/36
glycerol consumer - CV115	2/51	2/55	2/52	2/47	** 5/51	** 7/47	** 5/48	** 7/50	** 1195/32	** 1230/33	** 1215/34	** 1212/32
glycerol consumer - CV116	0/53	0/57	0/54	9/49	** 5/51	** 7/47	** 5/48	** 6/51	** 1200/27	** 1235/28	** 1221/28	** 1217/27
