# Supplementary material for: Acetate and glycerol are not uniquely suited for the evolution of cross-feeding in E. coli
Source: PLoS Comput Biol. 2020 Nov 30;16(11):e1008433. doi: 10.1371/journal.pcbi.1008433 (PMC7728234; doi:10.1371/journal.pcbi.1008433)
Supplement: S9 Fig — On the vertical axis, colored circles next to each metabolite’s acronym indicate the product of maximal metabolite production and biomass yield, where the same color code as in Fig 2 from [28] is used. Specifically, community biomass increases from yellow to green to blue. The figure shows that cross-feeding interactions whose evolution requires few metabolic changes (i.e., low ancestor-producer plus ancestor-consumer distances) usually result in high community biomass (blue circles). Pink circles indicate carbon sources that E. coli can excrete when growing in glucose minimal medium [50]. (PDF) [file pcbi.1008433.s017.pdf]

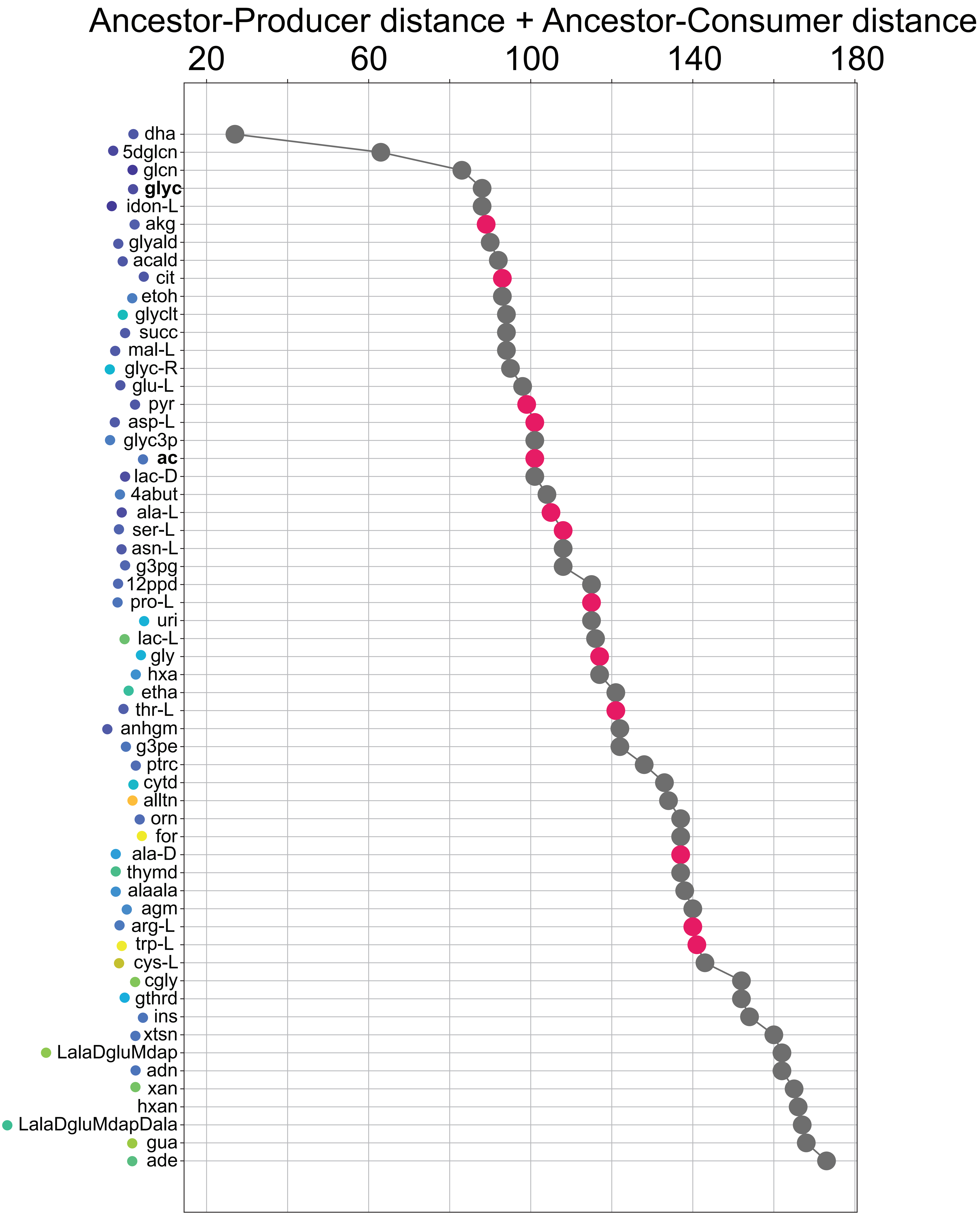

Ancestor-Producer distance + Ancestor-Consumer distance
20
60
100
140
180
dha
5dglcn
glcn
glyc
idon-L
akg
glyald
acald
cit
etoh
glyclt
succ
mal-L
glyc-R
glu-L
pyr
asp-L
glyc3p
ac
lac-D
4abut
ala-L
ser-L
asn-L
g3pg
12ppd
pro-L
uri
lac-L
gly
hxa
etha
thr-L
anhgm
g3pe
ptrc
cytd
alltn
orn
for
ala-D
thymd
alaala
agm
arg-L
trp-L
cys-L
cgly
gthrd
ins
xtsn
LalaDgluMdap
adn
xan
hxan
LalaDgluMdapDala
gua
ade
