# Supplementary material for: Acetate and glycerol are not uniquely suited for the evolution of cross-feeding in E. coli
Source: PLoS Comput Biol. 2020 Nov 30;16(11):e1008433. doi: 10.1371/journal.pcbi.1008433 (PMC7728234; doi:10.1371/journal.pcbi.1008433)
Supplement: Attachment — Submitted filename: reply_reviewers.docx [file pcbi.1008433.s018.docx]

Subject: Resubmission of manuscript number (PCOMPBIOL-D-20-00163) - [EMID:4340f5a301afa525]: **"Acetate and glycerol are not uniquely suited for the evolution of cross-feeding in E. coli"**

Dear editors and reviewers,

Thank you for allowing us to resubmit our manuscript entitled "Acetate and glycerol are not uniquely suited for the evolution of cross-feeding in *E. coli*".

We would also like to thank you and the referees for your time and your constructive comments and suggestions. We have addressed the comments made by the reviewers which have helped us prepare a much improved version of the manuscript that also communicates our observations more clearly.

Below is our point-by-point reply to the comments and suggestions of the reviewers.

Kind Regards,

Magdalena San Roman

Andreas Wagner

**------------------**

**Reviewer #1:**

**------------------**

**The authors use flux balance analysis to explore which cross-feeding interactions are most likely to evolve in E. coli growing in a minimal medium containing glucose as the sole carbon source. They show that - according to their models and assumptions - the two that have been found to evolve in previously published experiments, glycerol and acetate, do not seem to be the two that are most likely to evolve. They determine this by calculating the number of metabolic changes (on/off/quantitative change) that are needed for each metabolite to be produced and consumed by mutants. They find that there are other metabolites that require fewer changes to the ancestor compared to the two that were found. Assuming parsimony, those should be more likely to evolve.

I found the analysis to be very thorough and convincing. The assumptions of the approach and its short-comings are very clearly stated, and seem reasonable to me. I find it hard to imagine how to do this otherwise. The paper is also very well written and a pleasure to read, with many interesting findings.**

Thank you for these encouraging words**.**

**Regarding the conclusion: the authors hypothesize that cross-feeding on many other metabolites may have evolved, but has just gone undetected. You write: “In previous work [27] we showed that about half of the metabolites we study here would support lower community biomass than acetate cross-feeding. Thus, the frequencies of some cross-feeding strains in a population may be low and hard to detect”. These short sentences seem crucial to answering the question. If this is really something that could explain the findings, then I think it merits additional explanation. It was not clear to me why this would be the case.**

Thank you for raising this issue. In response, we now added a new supplementary section (Text S6) and a supplementary figure (Fig S9), where we explain this issue further. In this new section, we also speculate on which novel cross-feeding interactions we expect to detect experimentally based on community biomass considerations. We emphasize that these considerations are speculative, which is also why we feel that they should go into the supplement and not into the main text.

Supplementary Text S6 reads:

**Which novel cross-feeding interactions are likely to be found experimentally?**

In this section we offer some informed speculations on still unknown cross-feeding interactions that are most likely to be found, based on their likelihood to evolve and to be discovered experimentally.

In previous work [1] we simulated the dynamics of a producer and consumer strain in a chemostat. We observed that coexistence of the producer and consumer strains was possible for all 58 predicted metabolites in a wide range of conditions. However, we also observed that the cross-fed metabolite affected the community biomass (the sum of the biomass values of producer and consumer strains) considerably. We proved that the community biomass was explained by the product of two quantities: the maximal flux of the cross-fed metabolite that can be excreted by the producer strain (which is related to the cost of producing this metabolite), and the biomass yield of the metabolite (which is the growth rate that can be achieved per unit of metabolite consumed).We also showed that approximately half of the predicted cross-feeding interactions (29 out of 58) support higher community biomass than acetate cross-feeding [1] (supplementary figure S9). Cross-feeding interactions resulting in larger community biomass may be easiest to detect experimentally.

Alpha-ketoglutarate (akg), citrate (cit), pyruvate (pyr) and aspartate (asp-L) are the four metabolites that (i) support highest community biomass [1], (ii) can be excreted by E. coli in glucose medium [2], and (iii) require few metabolic changes for their cross-feeding interaction to evolve (Fig 2 of the present work and supplementary figure S9). Additionally, to our knowledge none of these metabolites is toxic for *E. coli*. *E. coli* is normally unable to grow aerobically on citrate, due to the inability to express a citrate transporter in the presence of oxygen, which would hinder citrate cross-feeding evolution. Taken together, these considerations suggest that alpha-ketoglutarate, pyruvate and aspartate may be the best candidates for involvement in cross-feeding interactions that can both readily evolve and be detected experimentally.

1.	San Roman M, Wagner A. An enormous potential for niche construction through bacterial cross-feeding in a homogeneous environment. PLOS Computational Biology. 2018;14: e1006340. doi:10.1371/journal.pcbi.1006340

2.	Paczia N, Nilgen A, Lehmann T, Gätgens J, Wiechert W, Noack S. Extensive exometabolome analysis reveals extended overflow metabolism in various microorganisms. Microbial Cell Factories. 2012;11: 122. doi:10.1186/1475-2859-11-122

The following is the new supplementary figure S9.

**Fig S9:** The horizontal axis shows the total metabolic distance to the ancestor found with RooM (as in Fig 2B). On the vertical axis, colored circles next to each metabolite’s acronym indicate the product of maximal metabolite production and biomass yield, where the same color code as in figure 2 from [29] is used. Specifically, community biomass increases from yellow to green to blue. The figure shows that cross-feeding interactions whose evolution requires few metabolic changes (i.e., low ancestor-producer plus ancestor-consumer distances) usually result in high community biomass (blue circles). Pink circles indicate carbon sources that *E. coli* can excrete when growing in glucose minimal medium [50].

**A further point that merits discussion is that each metabolite may have different effects on growth, if, for example they change the pH of the environment in an unfavorable way to the ancestor.**

Very good point, and one that we had clearly failed to discuss. We revised a section in the discussion accordingly, which now reads (line 509):

“There may be several reasons why only acetate and glycerol cross-feeding have been observed experimentally, even though multiple other carbon sources may be just as likely to evolve cross-feeding. First, we cannot exclude that other cross-feeding interactions did evolve but went undetected, because detecting cross-feeding requires extensive genetic and biochemical analysis. In addition, cross-feeding strains must reach a sufficiently high population frequency to become detectable. In previous work[29] we showed that about half of the metabolites we study here would support lower community biomass than acetate cross-feeding. Thus, the frequency of some cross-feeding strains in a population may be low and hard to detect (see supplementary Text S6 and supplementary figure S9). Second, the excretion of some metabolites may have negative effects on growth if the metabolite is toxic or if it changes the pH of the environment in unfavorable ways. Third, cross-feeding interactions may be transient. Given sufficient time, a generalist strain that combines maximum glucose uptake with the ability to recover a secondary metabolite could evolve[40,41]. Evolution experiments in environments alternating between pairs of carbon sources showed that such generalists evolve when both carbon sources are metabolized in similar ways, whereas specialists evolved when carbon sources are metabolized differently[42]. Based on this observation, one would expect that a generalist might replace a cross-feeding polymorphism if glucose and the cross-fed metabolite are metabolically similar. Fourth, the evolution of acetate and glycerol cross-feeding in the chemostat experiments may have been facilitated by the initial genotype. The reason is that the ancestral genotype in these experiments harbored regulatory mutations that prevented cells from recovering excreted acetate and overexpressed the glycerol regulon [15,36]. “

**The other thing that would be nice to see is a bit more of a detailed analysis of how these results correspond to what has been seen in the genomic analysis (mutations) of evolved cross-feeding E. coli populations from previous work. The authors state that there is some overlap in the results (“Multiple repeatedly mutated genes [14] were directly involved in glycolysis and gluconeogenesis, the TCA cycle, and transport, which are three of the subsystems where we also observe most of the reactions changes”), and then focus on transcriptomic data. It’s true that the transcriptomic data is a better place to start, but it’s only looking at 72 genes, and there might be something interesting looking at mutations (perhaps in a supplementary figure). If some correspondence is found (even though one would not necessarily expect a lot of overlap, for reasons that the authors already state), it would provide support for the method.**

As the reviewer suggests, we now use mutation data (from supplementary Table 2 of Kinnersley et al. 2014) to perform a detailed genome-level analysis.

We revised supplementary S3 Text to include this analysis, whose outcome is also shown in S1 File. We added the following text in supplementary S3 Text:

“**Comparing computational predictions and experimental data on genomic mutations and transcriptional changes**

Thorough experimental analysis performed on the ancestral and acetate/glycerol cross-feeding *E. coli* strains provided extensive information on mutations [1]and gene expression differences among strains [2]. Here we compare these experimentally obtained data to our computational predictions.

A total of 535 different genes had undergone mutations in evolved cross-feeding strains relative to the ancestral (JA122) strain (data from supplementary Table S2 of [1]). 168 out of those 535 mutated genes are genes included in the metabolic model of *E. coli* [3] that we used. In a first analysis, we explored the agreement between the mutated genes, and those genes associated with reactions that we computationally predict to require a flux change for the evolution of cross-feeding.

To this end, we used the Gene-Protein-Reaction association (GPR) map available for the metabolic model of *E. coli i*JO1366 [3]. The relationship between genes and enzymatic reactions is only one-to-one in the simplest case, where one gene product catalyzes one reaction. Alternatively, a gene product may catalyze more than one reaction; the products of multiple genes may be needed to catalyze one reaction; or the products of different genes may catalyze the same reaction.

We mapped every reaction that we predict (with RooM) to require a flux change for the evolution of a cross-feeding strain to its corresponding gene or genes (see supplementary S1 File). Then, for each cross-feeding strain, we determined whether a gene associated with a flux-changing reaction underwent one or more mutations in the experiment. If so, we reported the number of mutations found in the gene (supplementary S1 File).

A few genes associated to reactions that we predict to require a flux change for the evolution of the cross-feeding strains, appeared mutated is the chemostat experiments [1]. The largest agreement between computational predictions and experimental data is observed for the acetate producer strain. In this case, out of the 118 genes associated to reactions requiring a flux change for the evolution of the acetate producer strain, 16 (approximately 7%) appeared mutated in strain CV103 (supplementary S1 File). ”

And:

“The analysis show that the agreement between experimental data and computational predictions is limited, which is unsurprising. At genome level we expected little agreement given that mutations not directly affecting metabolic genes can have large impacts on the rate at which metabolic reactions proceed. That is the case, for example, with regulatory mutations. Little agreement between experimental data and computational predictions at the level of transcripts could arise from the fact that metabolic flux is affected by multiple factors besides mRNA expression level. An extra source of disagreement may come from the fact that the strain found experimentally to excrete acetate (CV103) also excretes glycerol, while we computationally modeled two separate producer strains excreting either acetate or glycerol.”

In addition, we added the following text on line 254 of the main text:

“Consistent with the prediction that one or few mutations could not bring about all these changes, experimentally evolved cross-feeding strains harbored hundreds of mutations. Multiple repeatedly mutated genes [14] were involved in glycolysis and gluconeogenesis, the TCA cycle, and transport, which are three of the subsystems where we also observe most of the reactions changes. Only a limited number of mutated genes [14] directly map onto metabolic reactions that we predict to require a flux change (supplementary S3 Text and supplementaryS1 File), and transcriptomic changes [14,15] also show very limited agreement with computational predictions (see supplementary S3 Text, supplementary Fig S3 and supplementary File S2). This is unsurprising, because of the ambiguity of gene-reaction associations, and because gene expression change poorly reflects metabolic flux change for several reasons, for example because mRNA and enzyme abundance correlate poorly[23–28].”

**Minor comments:
- You mention that parsimonious FBA is not the standard. Can you say (perhaps in the discussion) what would happen if you used regular FBA? Is parsimonious more conservative? Do you expect to see the same patterns?**

In a metabolic system as complex as that of *E. coli*, multiple alternative flux distributions fulfill all constrains required by FBA and permit maximal growth in any one environment. When performing Flux Balance Analysis (FBA) without any further restrictions on these solutions, an arbitrary one of these solutions is returned. Parsimonious FBA (pFBA) identifies, from the set of alternative solutions, the one that minimizes cost to the cell. This solution has been found to better reproduce experimental data. This motivated us to initially use pFBA to identify the flux distribution of the ancestral strain.

We also wondered how our results would be affected by the flux distribution used to describe the ancestral strain. In fact, it was with that question in mind that we originally developed RooM-het. In RooM-het, the flux distribution of the ancestral strain is not set to that of pFBA, but it can be any solution that fulfils all constraints. In other words, the ancestral flux distribution identified with RooM-het can be any solution compatible with plain FBA. Figure 2b shows that allowing this flexibility in the ancestral flux distribution has little effect on the distances found to producer and consumers. We mention this issue briefly in two sections of the paper. In line 169 we included:

“(Later, we determine the ancestral flux distribution with an alternative method and show that results are robust to the method used to find the ancestral flux distribution.)”

And in line 395 we state:

“This result also suggests that our initial results (Fig 1) are insensitive to the ancestral flux distribution used.”

**- Fig. 1: I find the very small text on the networks a bit annoying. Do you expect the reader to zoom in to read them? I would just remove the text and make the orange circles larger. The point after all is to show large changes.**

As the reviewer suggested, we removed the text from the networks in Fig 1, and enlarged the orange circles. In addition, we added supplementary figure (S1) for those readers who might be interested in zooming in to see details about metabolite names, reaction names, and fluxes.

**- Caption of Fig. 2: RooM-het appears but is only explained later in the text. Maybe just warn the reader.**

Clarified. We changed the last sentence from the caption of Fig 2 (line 303) to:

“Black circles show the total distances obtained when we used RooM-het (explained in the following section) to minimize strain distances.”

**- L. 307: “cross-feeding of 18 metabolites is easier to evolve” than what? I was confused how these 18 compare to the 7 listed in the previous paragraph.**

We apologize for causing confusion, and have aimed to clarify the paragraph in question, where we had first mentioned that there are 7 metabolites for which both producer and consumer strains can evolve with fewer metabolic changes than the acetate producer and consumer strains respectively. Then, we used the sum of the ancestor-producer and ancestor-consumer distances as proxies for the likelihood that cross-feeding interactions evolve. We found that 18 cross-feeding interactions can evolve more easily than acetate cross-feeding.

To clarify, we removed the passage mentioning the 7 metabolites, because they distract from the main message. We now state instead (line 306):

“Fig 2B shows 58 carbon sources ranked by the likelihood that cross-feeding evolves for them. Based on our minimal change criterion, cross-feeding of 18 carbon sources can evolve more easily than acetate cross-feeding – it requires fewer reaction changes in producer and consumer. In contrast, cross-feeding of only three carbon sources is easier to evolve than glycerol cross-feeding. In sum, acetate and glycerol are not exceptional in their potential to evolve in cross-feeding.”

**- L. 328: “acetate and glycerol cross-feeding would not be exceptional in their metabolic distance to the ancestor”. No, but they are on the low end, so in the set that is more likely to evolve. Possibly one additional (yet unknown) factor may explain why they preferentially evolve.**

True. In this work we explore a few of all imaginable hypotheses. For example, another hypothesis that remains unexplored is that there might be more alternative ways (more sets of mutations) that would give rise to acetate and glycerol cross-feeding. Yet, even existing experiments leave open the possibility that other cross-feeding interactions have evolved in them but went undetected. The cause of multiple polymorphisms that evolved in the batch and chemostat experiments remain unknown and the authors suggest cross-feeding of other metabolites as a possible explanation. In addition, the ancestral strain used in the chemostat experiments already had mutations that predisposed acetate and glycerol cross-feeding to evolve. This might explain why only acetate and glycerol cross-feeding was observed in the chemostat experiments which only lasted a few hundred generations.

**- L. 336ff: “This overlap suggests that the mutations required to create producer and consumer strains of different metabolites may also overlap.” To what extent do glycerol and acetate overlap? Do they actually both evolve in the same experiments and if so, might that be explained by a particularly high overlap?**

The following figure (figure 1 from Kinnersley et. al (2014) *PLOS Genetics*) shows the phylogeny of the four strains that evolved in one of the experiments where *E. coli* was kept in a chemostat on a glucose minimal medium. Strain CV103 scavenges glucose best but excretes acetate and glycerol to the environment. Strain CV101 grows from acetate consumption while strains CV115 and CV116 grow from glycerol consumption. The numbers of SNPs found are shown above each branch in the phylogeny. The figure shows that some mutations are shared between the different strains. Yet there are multiple mutations which are specific to each strain. Unfortunately, we do not know which is the set of mutations responsible for the distinct metabolic phenotypes of the strains.

**- L. 424: “it is not surprising that the sum of the ancestor-producer and ancestor-consumer distances decreases as glucose consumption increases.” I found this surprising. It would be good to spell out the intuition of why this may be the case. I believe it came later in the discussion, but would be useful to explain it here.**

We now do explain this observation earlier. When more than the minimal glucose consumption is allowed, alternative ways of metabolizing carbon open up. This can be viewed as enlarging the grey area of figure 3A, which represents the solution space of the ancestral strain – the space of alternative solutions that fulfill all constraints imposed on the ancestral strain. As this space gets larger, it can only get ‘closer’ to (decrease the distance from) the blue and red regions, which correspond to the solution spaces of the producer and consumer strains.

To clarify this issue, we added the following paragraph in line 424:

“In sum, ancestor-producer and ancestor-consumer distances become smaller when the ancestor can consume more glucose than minimally necessary to persist in the chemostat. Fig 3A provides a geometric intuition for this observation. Increasing glucose consumption by the ancestral strain results in a larger allowable solution space for this strain (grey area in Fig 3A), because the carbon it consumes can be metabolized in a greater number of alternative (and possibly less efficient) ways. As the allowable solution space for the ancestor increases, the distance to the producer’s and consumer’s solution space cannot increase as well - it can only remain unchanged or decrease. ”

**- L. 430: “Acetate ranks highest (fifth)” I was confused how fifth was highest if you are testing 20 metabolites**

We meant to say that acetate ranks at most fifth-highest among all cross-fed metabolites for the glucose consumption values explored. To state this more clearly, we have changed the text to (line 432):

“The extent to which ancestor-producer and ancestor-consumer distances decrease when the ancestor consumes more glucose depends on the cross-fed metabolite (Fig 3B). This dependency may also affect the ranking of metabolites most likely to be involved in the evolution of cross-feeding as glucose consumption changes. For example, Spearman’s correlation coefficient of these ranks varies from a minimum of 0.42 (when changing glucose consumption from 2.3 to 2.5 mmol gDW^-1^ h^-1^) to a maximum of 0.89 (when changing glucose consumption from 2.7 to 2.9 mmol gDW^-1^ h^-1^). Acetate cross-feeding evolution ranks highest among all glucose consumption rates (i.e., it shows the lowest distance to the ancestor) when glucose is consumed at a rate of 2.5 mmol gDW^-1^ h^-1^ (orange circles in Fig 3B). Even then, however, cross-feeding involving dihydroxyacetone (dha), D-gluconate (glcn), glycolate (glyclt) and glyceraldehyde (glyald) are more likely to evolve.”

**- L. 486ff: “we tacitly assumed that genetic change causes the metabolic differences leading to cross-feeding. However, phenotypic plasticity may also be involved, especially for the consumer strain”. Genetic change can also occur due to drift or be due to selection for non-metabolic traits such as motility.**

Our intention was not to say that the origin of new metabolic traits always involves natural selection. Instead we wanted to acknowledge that metabolic changes may arise through phenotypic plasticity, without a need for genetic changes.

**- Some small typos here and there (s’s where they shouldn’t be, one figure label says figure_s5.pdf)**

Sorry for that and thanks for pointing this out. We have double checked for typos and corrected the figure label.

**------------------
------------------**

**Reviewer #2:**

**------------------**

**San Roman and Wagner asked why E. coli in low glucose evolved cross-feeding interactions mediated by acetate and glycerol (and not other metabolites). One possibility is that other metabolites are released and consumed, but these interactions have not been detected. To address this possibility, they used metabolic modelling to estimate the likelihood of evolving cross-feeding interactions by how small the total number of metabolic changes required in the ancestor to give rise to a producer strain and a consumer strain. They found that among the 50+ metabolites predicted to be released (including acetate and glycerol), acetate and glycerol are not really exceptional, and that these cross-feeding interactions would require changes in many metabolic reactions (twenty or so reactions need to be turned on or off). Consistently, previous published experimental work showed that repeatedly mutated genes are involved in three of the subsystems predicted to be important. Their model further predicted that although phenotypic heterogeneity only slightly helped the evolution of cross-feeding, consuming glucose more than what was required for growth in chemostats would significantly facilitate the evolution of cross-feeding. The paper proposes tantalizing hypotheses, and is easy to follow.
Main comments:
1. Why is there such a nice positive correlation between ancestor-producer distance and ancestor-consumer distance?**

Thank you for raising this issue. This is a very interesting observation that we had not paid much attention to. We performed additional analyses to help answer your question. The first and most important fact is that for any given cross-fed carbon source the reactions requiring a flux change in both the producer and consumer strains overlap greatly. Figure R1 below shows that the number of reactions shared between producer and consumer increases as the distances to the ancestor increases.

Figure R1: The y-axis shows the number of reactions that require a flux change in both producer and consumer if cross-feeding of a given metabolite is to evolve. The x-axis shows the ancestor-producer (left panel) and ancestor-consumer (right panel) distances.

At the same time, the direction of flux change in those shared reactions is often different between producers and consumers, as illustrated by Figure R2.

Figure R2: Central carbon metabolism of *E. coli* (like Fig. 1 in the main text), where reactions that require a flux change in both producer and consumer strains of acetate (left panel) and glycerol (right panel) are shown in color. Shared reactions in which flux changes in opposite directions are shown in orange.

In sum, the positive correlation observed between ancestor-producer and ancestor-consumer distances can be explained by the large overlap of reactions requiring a flux change during the evolution of producer and consumer strains, even though the magnitude and direction of the change differs between producer and consumer.

We describe this observation qualitatively in line 283:

“The correlation observed between ancestor-producer and ancestor-consumer distances can be explained by the large overlap of reactions requiring a flux change during the evolution of producer and consumer strains, even though the magnitude and direction of the change differs between producer and consumer (supplementary S1 Text).”

**2. You listed two limitations of FBA modelling in your discussions, whereas in reality, there could be more. For example, your model assumed that all excessive metabolites are released whereas in reality, transporters of metabolites might not be there to facilitate release.**

We agree that this is an important point to clarify. We are only considering the excretion of metabolites for which there are transporters or that can diffuse through the cell membrane. We thus added the following text on line 269 for clarification:

“(Note that our work only considers cross-fed metabolites that can either diffuse through the cell membrane or that can be excreted or imported by an *E. coli* transport proteins.)”

**3. A related point of “not detecting cross-feeding” is that the frequency-dependent fitness advantage of consumer over producer is very important for the consumer to rise to a sufficiently high frequency to be detected. As an example, see our preprint** [**https://www.biorxiv.org/content/10.1101/498543v2**](https://www.biorxiv.org/content/10.1101/498543v2)

We fully agree that a frequency-dependent advantage explains the increase of consumer abundance in the chemostat, and have already shown this in our previous work (San Roman and Wagner 2018). In this work, we had simulated the dynamics of a community that initially harbored only a producer strain. At a later time point we introduced the consumer strain at a very low abundance, to simulate its evolution. We found that the consumer strain initially increases its abundance due to a frequency-dependent effect. Specifically, the secondary carbon source excreted by the producer strain is at first consumed (and needs to be ‘shared’) only by a few consumer cells. This results in a high growth rate of the consumer strain, and increased consumer abundance. As the number of consumer cells increases, the available secondary carbon source has to be shared between more cells, reducing the consumer’s growth rate. Eventually, a steady-state is reached where both producer and consumer strains are abundant.
