# Supplementary material for: Acetate and glycerol are not uniquely suited for the evolution of cross-feeding in E. coli
Source: PLoS Comput Biol. 2020 Nov 30;16(11):e1008433. doi: 10.1371/journal.pcbi.1008433 (PMC7728234; doi:10.1371/journal.pcbi.1008433)
Supplement: Attachment — Submitted filename: reply_reviewers.docx [file pcbi.1008433.s019.docx]

Subject: Resubmission of manuscript number (PCOMPBIOL-D-20-00163R1) - [EMID: 4790d3056fd9256f]: **"Acetate and glycerol are not uniquely suited for the evolution of cross-feeding in *E. coli*"**

Dear editors and reviewers,

Thank you for allowing us to revise our manuscript entitled "Acetate and glycerol are not uniquely suited for the evolution of cross-feeding in *E. coli*".

Below is our reply to the remaining few comments of the reviewers.

Kind Regards,
Magdalena San Roman
Andreas Wagner

**------------------**

**Reviewer #1:**

**------------------**

**I would like to thank the authors for addressing my questions so thoroughly. I especially appreciate the additional analysis of the experimental data on mutations (now supplementary text S3 and S1 file). I also find that supplementary text S6 adds a satisfying explanation to the existing results. I have no further recommendations for improvement.**

Thank you again for your support and for all your suggestions, which helped us improve the paper substantially.

**------------------
------------------**

**Reviewer #2:**

**------------------**

**The authors misunderstood me. What I meant is that the fitness advantage part is not a given (no law states that consumers must gain an advantage over producers). This lack of advantage could contribute to "not detecting cross-feeding".**

We are sorry for the misunderstanding, and now incorporate a passage reflecting this caveat into the discussion. Before we detail how, we paraphrase from the paper you cite in your original comment, to make sure we understood you correctly this time.

Your analysis (<https://www.biorxiv.org/content/10.1101/498543v2>) of adaptation occurring through the evolution of a more “faulty” strain is fascinating. When a yeast population auxotrophic for lysine (strain *lys^-^*) is evolved in a medium with low levels of lysine you observe that a strain which lost its ability to synthesize organosulfurs evolves (strain *lys^-^ orgS*^-^). Strains *lys^-^* and *lys^-^ orgS*^-^ are able to coexist, because strain *lys^-^* releases organosulfurs when growing in a medium with low levels of lysine. If we consider the energy-saving hypothesis, we would expect strain *lys^-^ orgS*^-^ to be fitter than *lys^-^* when growing in a medium containing organosulfurs, because *lys^-^ orgS*^-^ imports organosulfurs, thus saving energy that would otherwise be used to build organosulfurs from sulfur. Yet, this is not what you observe. The fitness advantage of *lys^-^ orgS*^-^ over *lys^-^* is also observed in a medium lacking organosulfurs. The explanation is that the evolved metabolic defect restores a correct nutrient-growth regulation that is missing in strain *lys.^-^*

Applying this observation to our work, it is possible that the consumption of some metabolites (such as acetate or glycerol) offers some unexpected advantage to the consumer strain. The metabolite consumed may not only serve as a source of carbon for the consumer strain, but it may help regulate some other processes. Even though all 58 metabolites we analyzed serve as a carbon source, only some (maybe acetate) might give an extra advantage to the consumer strain.

We now include this additional caveat in lines 518-526:

“There may be several reasons why only acetate and glycerol cross-feeding have been observed experimentally, even though multiple other carbon sources may be just as likely to evolve cross-feeding. First, we cannot exclude that other cross-feeding interactions did evolve but went undetected, because detecting cross-feeding requires extensive genetic and biochemical analysis. In addition, cross-feeding strains must reach a sufficiently high population frequency to become detectable. In previous work [29] we showed that about half of the metabolites we study here would support lower community biomass than acetate cross-feeding. Thus, the frequency of some cross-feeding strains in a population may be low and hard to detect (see supplementary Text S6 and supplementary figure S9). Second, the excretion of some metabolites may have negative effects on growth if the metabolite is toxic or if it changes the pH of the environment in unfavorable ways. Third, we cannot rule out that the consumption of acetate or glycerol might give an advantage to the consumer strain that derives from more than just their role as carbon sources. A precedent for this possibility exists [40]. When a population of yeast cells auxotrophic for lysine encountered lysine limitation, coexistence of the ancestral lysine auxotroph strain and a mutant organosulfur auxotroph repeatedly evolved. The organosulfur auxotroph strain persisted in the population, because it consumed organosulfurs excreted by the ancestral strain. Organosulfur auxotrophy conferred an advantage to the mutant strain, because it recovered the proper nutrient-driven growth regulation that had been impaired in the ancestral strain [40]. Fourth, … “
